# Nomenclature, classification, and *in-silico* structural analysis of MSP1a variants of *Anaplasma marginale*

**DOI:** 10.1371/journal.pone.0360505

**Published:** 2026-10-21

**Authors:** Amam Zonaed Siddiki, Nazifa Humaira, Nanziba Sharmin Hossain, Yusra Bint Akbar, Puja Palit, Pann Ei Phwe, Rahima Akter Eva, Hala Alshaar, Marjia Islam Tia, Kethain Marma, Barsha Akter, Samira Sultana, Sabreena Alam

**Affiliations:** 1 Genomics Research Group, Department of Pathology and Parasitology, Chittagong Veterinary and Animal Sciences University (CVASU), Chittagong, Bangladesh; 2 Department of Bioinformatics and Biotechnology, Asian University of Women (AUW), Chittagong, Bangladesh; 3 Department of Biochemistry and Biotechnology, University of Science and Technology Chittagong (USTC), Chittagong, Bangladesh; National Veterinary Research Institute (NVRI), NIGERIA

## Abstract

Bovine anaplasmosis, caused by the tick-borne rickettsial pathogen *Anaplasma marginale*, is a significant threat to livestock health and productivity. The major surface protein 1a (MSP1a) is a widely used molecular marker for characterizing strain diversity, due to its variable N-terminal tandem repeats. In this study, we analyzed and updated the nomenclature classification of 157 *A. marginale* MSP1a sequences submitted to NCBI between 2013 and 2023, following the research by Cabezas-Cruz in 2013. We identified and classified 189 unique tandem repeats, reported their tick-transmissibility, predicted their secondary structures and B-cell epitopes, and analyzed amino acid variability. Following this, we conducted a comparative analysis with previously published datasets, which revealed new tandem repeat types, new positions of serine within tandem repeats, and high variation among B-cell epitope variants. We have also classified the unique R1 tandem repeats of the new MSP1a sequences into different ecoregion clusters based on the principal component analysis of NDVI values. This updated classification system enhances the molecular understanding of *A. marginale* diversity and may contribute to future developments in strain typing, epidemiological investigations, and vaccine research.

## Introduction

Bovine anaplasmosis is an infectious tick-borne disease caused by the gram-negative rickettsial bacterium *Anaplasma marginale*. The disease is transmissible among cattle, including cows, bison, and water buffalo. Anaplasma is an obligate, intraerythrocytic bacterium primarily transmitted by the bite of infected ticks, competent biological vectors of *Anaplasma marginale*. This pathogen may also be transmitted mechanically via blood-contaminated mouthparts of biting flies or via contaminated fomites such as needles and other surgical instruments. The vector-borne disease also spread through contaminated equipment used during ear tagging, tattooing, vaccination, and other procedures.

Different strains of *A. marginale* have been recorded worldwide, varying in protein sequences, morphology, antigenicity, and tick-transmissibility [1]. Among the various proteins, major surface proteins (MSPs) primarily interact with host cells. These MSP proteins include MSP1a, MSP1b, MSP2, and MSP4. Among them, the repeat sequences of the MSP1a alone can provide information on the genetic diversity of *A. marginale,* which contributes to understanding the evolutionary interactions among the vector, pathogen, and the host cells [2,3].

MSP1 is a heterodimer complex composed of two different proteins, MSP1a and MSP1b, joined together by non-covalent bonds. MSP1a is encoded by a single gene called msp1a, and the members of the msp1b multigene family encode MSP1b. The molecular mass of the MSP1a is 70–100 kDa. The molecular weight of MSP1a varies due to the variable number of tandem repeats, containing 23–31 amino acids in its tandem repeat [2,4]. The MSP1 complex induces protective immunity by eliciting antibody production when cattle are immunized or naturally infected with homologous or heterologous strains of *A. marginale*. MSP1a is considered a stable marker for identifying different *A. marginale* strains in various regions [5]. This protein is involved in the adhesion of *A. marginale* to the midgut of the tick and to the erythrocytes of cattle. In contrast, the MSP1b is engaged with the adhesion of *A. marginale* only to bovine erythrocytes. It was observed that the MSP1a protein is conserved across isolates and shows little sequence variation when extracted from cattle with persistent infections [6]. The tandem repeats of MSP1a are thought to be responsible for causing adhesion of the rickettsial parasite to the tick cells and bovine erythrocytes. The MSP1a sequence analysis also provides insights into vector-pathogen relationships, genetic diversity, the transmissibility of phenotypes, and the evolution of host-pathogen interactions. The tandem repeat of the MSP1a sequence can also be used for designing peptide vaccines. It is a stable marker of strains, which consists of an N-terminal variable number of tandem repeats and a 5’-untranslated region UTR microsatellite in its MSP1a gene. Moreover, MSP1a sequences can be used to compare strains from the same area.

The research reports a unified nomenclature for all new MSP1a sequences uploaded by 13 countries from 2013 to 2023, aiming to determine the worldwide diversity of *A. marginale* over the past decade, following Cabezas-Cruz’s 2013 study. The information generated in the current research has also been used to compare with the results of Cabezas-Cruz’s twelve-year-old study to evaluate the changes in the diversity of the outer membrane protein, MSP1a. The research provides updated insights into the diversity and variation of tandem repeats in MSP1a. The updated classification system enhances the molecular knowledge of *A. marginale* diversity and may contribute to more effective strain typing, epidemiological tracking, and vaccine formulation.

## Methodology

### Classification of available *Anaplasma marginale* strains

A total of 157 complete *A. marginale* MSP1a sequences, including tandem repeat regions, were downloaded from the NCBI database [7]. Only MSP1a sequences deposited between 2013 and 2023 were included. The geographic distribution of these MSP1a strains reflects the publicly available sequences deposited during this period. These MSP1a sequences were analyzed and categorized according to the nomenclature proposed by Allred et al. and de la Fuente et al. [2]. Similar approaches for nomenclature, using different aspects such as country/locality/microsatellite genotype – (structure of tandem repeat), proposed earlier by Kuby et al., were included. The MSP1a sequences that showed 100% amino acid identity were assigned a single strain name. When two strains had similar information, only the first, submitted to the NCBI GenBank database, was considered.

### Variability of amino acids within MSP1a tandem repeats

All the tandem repeat sequences were aligned using the ClustalW server [8], and each amino acid in the tandem repeat was numbered from 1 to 31. Following that, the variability of the amino acids was determined by using the formula of Kuby et al [9]. The variability was calculated as the number of different amino acids divided by the frequency of the most common amino acid at that position.

### Determination of selective pressures using MSP1a tandem repeats from various global regions

The analysis of the ecoregions was carried out as previously described by Cabezas Cruz, assuming (i) eco-regions could be distinguished by characters based on well-recognized and repeatable attributes, and (ii) A multivariate geographic cluster analysis was performed on *A. marginale* strains associated with each eco-region based on their environmental conditions. Based on NDVI, which measures vegetation stress and summarizes the ecological background of tick populations, eco-regions were established. A series of monthly NDVI data was obtained from the period January 2014 to December 2024 using Google Earth Engine. Subsequently, the SPSS software v30 was used to perform a Principal Component Analysis of the NDVI data. Using Principal Components Analysis (PCA), the 12 averaged monthly images were decomposed into principal axes that display the most significant, non-redundant information. Cattell’s Scree Test was used to analyze the strongest principal axes. The PCA analysis was simplified to three principal axes, accounting for 71.30% of the total variance. Hierarchical agglomerative clustering was then applied to the PCA values, which were used to classify multiple geographical areas, such as countries, into a single standard set of discrete regions. The measure of dissimilarity was deduced by Squared Euclidean distance, and the complete linkage was used as the amalgamation method. The cut-off probability was set to 0.05 for each eco-region. Hence, four eco-regions were identified through Principal Component Analysis: ecoregion 1, ecoregion 2, ecoregion 3, and ecoregion 4. The NDVI values of each ecoregion were then averaged to calculate the average NDVI value for that ecoregion. The rainfall data and land temperature data for the period from January 2014 to December 2024, for each country within each ecoregion, were then extracted using Google Earth Engine. The rainfall data for each country within each ecoregion were then averaged to calculate the annual rainfall for that ecoregion. The highest temperature was also determined for each ecoregion using the land temperature data specific to that ecoregion.

### Bioinformatics

Following the retrieval of MSP1a sequences from the GenBank of NCBI database [7], all the tandem repeats present in each of the MSP1a sequences were identified by operating the server, Tandem Repeats Finder. All parameters were set to their default values. The Tandem Repeats Finder generates results using a two-phase heuristic algorithm that screens raw nucleotide sequences for repeats by statistically analyzing the distances between matching k-tuple windows, following the stochastic Bernoulli model. It achieves 100% accuracy in detecting tandem repeats among polished, clean sequences downloaded from NCBI [10]. Each newly detected tandem repeat was then translated into its corresponding amino acid sequence using the ExPASy Translate tool. The ExPASy Translate tool generates results by applying the standard genetic code, which translates nucleotide triplets (codons) into amino acids with 100% deterministic accuracy [11]. The secondary structure of each tandem repeat was then predicted by using the PSIPRED server. The PSIPRED server predicts protein secondary structures using a two-stage neural network that processes sequences from a database and evaluates local evolutionary context via a sliding-window approach, yielding position-specific confidence scores for alpha helices and beta-strands. The results, represented as confidence scores, are generated with 80–85% accuracy [12]. Each tandem repeat was then submitted to the BCPREDS server to predict the B-cell epitopes within it. The BCPred server predicts linear B-cell epitopes using a sliding-window approach, which splits the target antigen sequence into overlapping peptides of expected lengths. These peptide sequences are then analyzed using a subsequence string kernel combined with a Support Vector Machine (SVM) classifier, which evaluates them against experimentally verified B-cell epitopes via scoring and identifies potential immunogenic regions. The BCPred server achieves 61.73% accuracy on bacterial proteins due to the presence of non-exposed regions. However, to maximize prediction accuracy and minimize false positives, our epitope mapping was restricted to the surface-exposed tandem-repeat sequences of MSP1a. This allowed the BCPred’s string-kernel pattern recognition to operate under optimal conditions and yield potential B-cell epitopes with higher 75–80% accuracy [13]. The B-cell prediction scores were determined using the BCPREDS server. Following that, we used the immunodominant B-cell epitope, SSAGGQQQESS, as our model sequence, following Cabezas-Cruz’s work to identify B-cell epitopes in our tandem repeats, which were similar in sequence to the model using the BLASTp server. The BLASTp server generates results by decomposing the query sequence into short, overlapping segments and searching the model sequence for matches. These initial sequences are extended into gapped high-scoring segment pairs via a heuristic alignment algorithm, and their statistical significance is assessed using normalized bit scores and E-values. The accuracy of the BLASTp results depends on how similar the query sequence of the predicted B-cell epitope is to the model sequence [14]. Subsequently, the Vaxijen server was used to predict the antigenicity of each B-cell epitope. The VaxiJen v2.0 server evaluates the antigenicity of an amino acid sequence by converting it into uniform vectors using Wold’s z-scale physicochemical properties, such as hydrophobicity, molecular size, steric properties and polarity and by generating antigen probability scores. The server achieves an overall accuracy of 82% for bacterial models and 91% sensitivity in detecting true antigens [15]. The Deep TMHMM server was used to determine the transmembrane topology of these epitopes. Deep TMHMM uses the amino acid sequence to predict protein topology, in which sequence embeddings are processed through a deep neural network and a conditional random field decoder to identify protein features such as transmembrane segments, signal peptides, and globular regions. The orientation of the protein segments is determined as either inside or outside based on the presence of certain amino acids, or on whether the protein adopts its entire orientation at the N-terminal or C-terminal. The server predicts results with an overall accuracy of 85%−90% [16]. The phylogenetic analysis of the MSP1a tandem repeats was conducted using MEGA11 [17]. The phylogenetic tree was constructed using Neighbor-joining and maximum likelihood methods. The bootstrapping method was adjusted to 1000 bootstrap replicates to generate reliable results. Following that, a permutation-based cluster association chi-square test was carried out to determine whether the cluster formations in the phylogenetic tree were associated with the 20^th^ amino acid position of the tandem repeats. Another permutation-based chi-square test was then carried out to determine whether the 20^th^ amino acid position in tandem repeats is associated with their secondary structures. The statistical tests were carried out using Python v3.13.0. A p-value less than 0.05 was considered significant.

## Results

### Implementation of MSP1a sequence data for the classification of *A. marginale* strains

A total of 189 distinct MSP1a tandem repeats were identified through intensive analysis. However, these tandem repeats were not previously classified in Cabezas-Cruz’s 2013 work (Fig 1). Uncommon MSP1a sequences from the years 2013–2023 were selected (120; 76.4% of all sequences found) depending on their differences in the number and the structure of the tandem repeats (S1 File). All the unique *A. marginale* strains were derived from 13 countries, reflecting the diversity of MSP1a (Fig 2; S2 File). All recently submitted MSP1a sequences from these countries were classified according to a new nomenclature (S3 File) using the formula proposed by Kuby et al. New countries included in this MSP1a sequence analysis, which were not part of Cabezas-Cruz’s study, were Colombia, Ecuador, Grenada, Turkey, Egypt, Thailand, and Russia.

**Fig 1:**
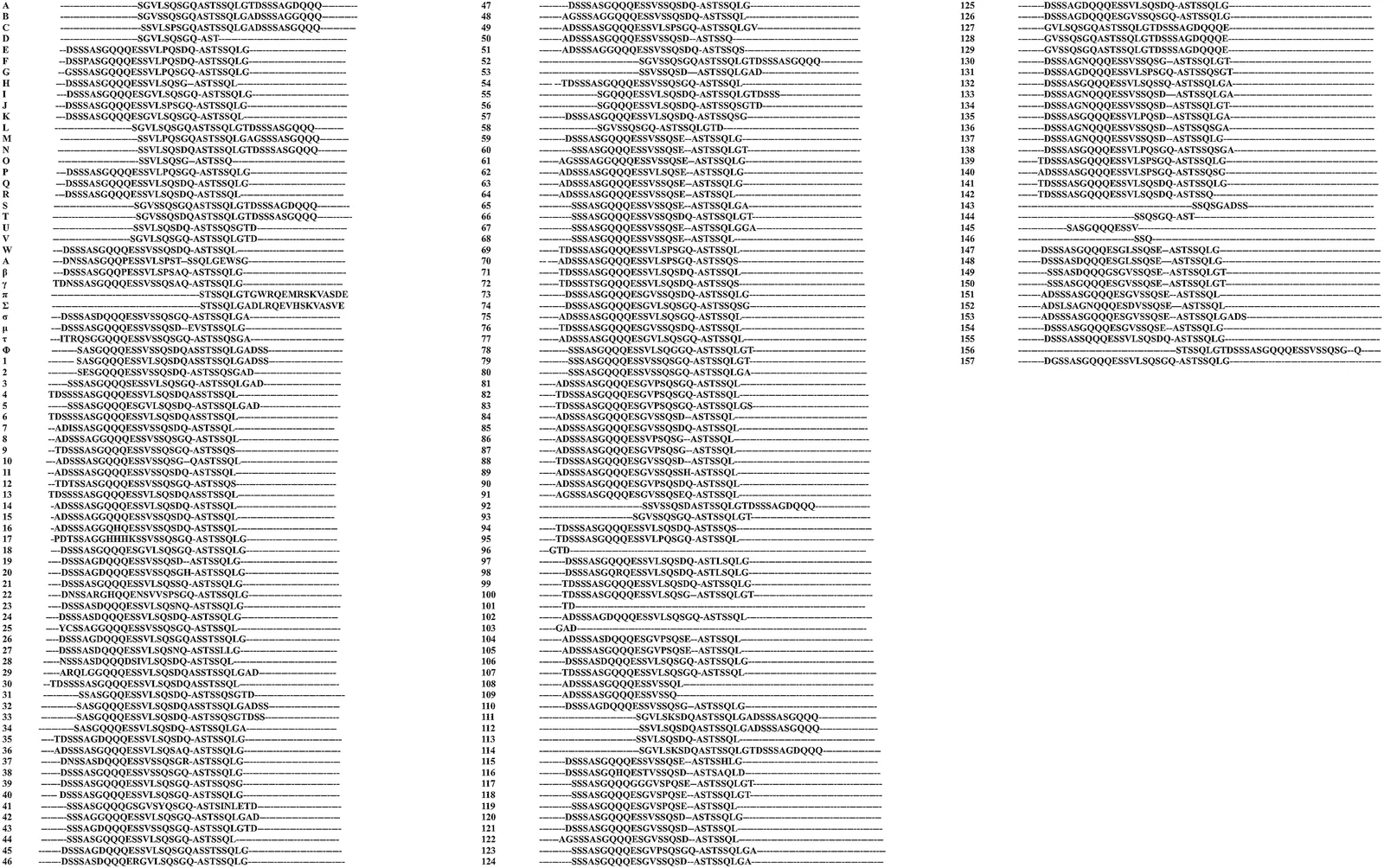
Tandem repeat sequences of MSP1a in *A. marginale* strains. All the MSP1a repeat sequences were represented using one-letter amino acid code. The dashes in the sequences depict the missing amino acids from the tandem repeats. All tandem repeats were assigned IDs according to the nomenclature proposed by Cabezas-Cruz and De la Fuente. All these tandem repeat sequences of MSP1a are newly classified.

**Fig 2:**
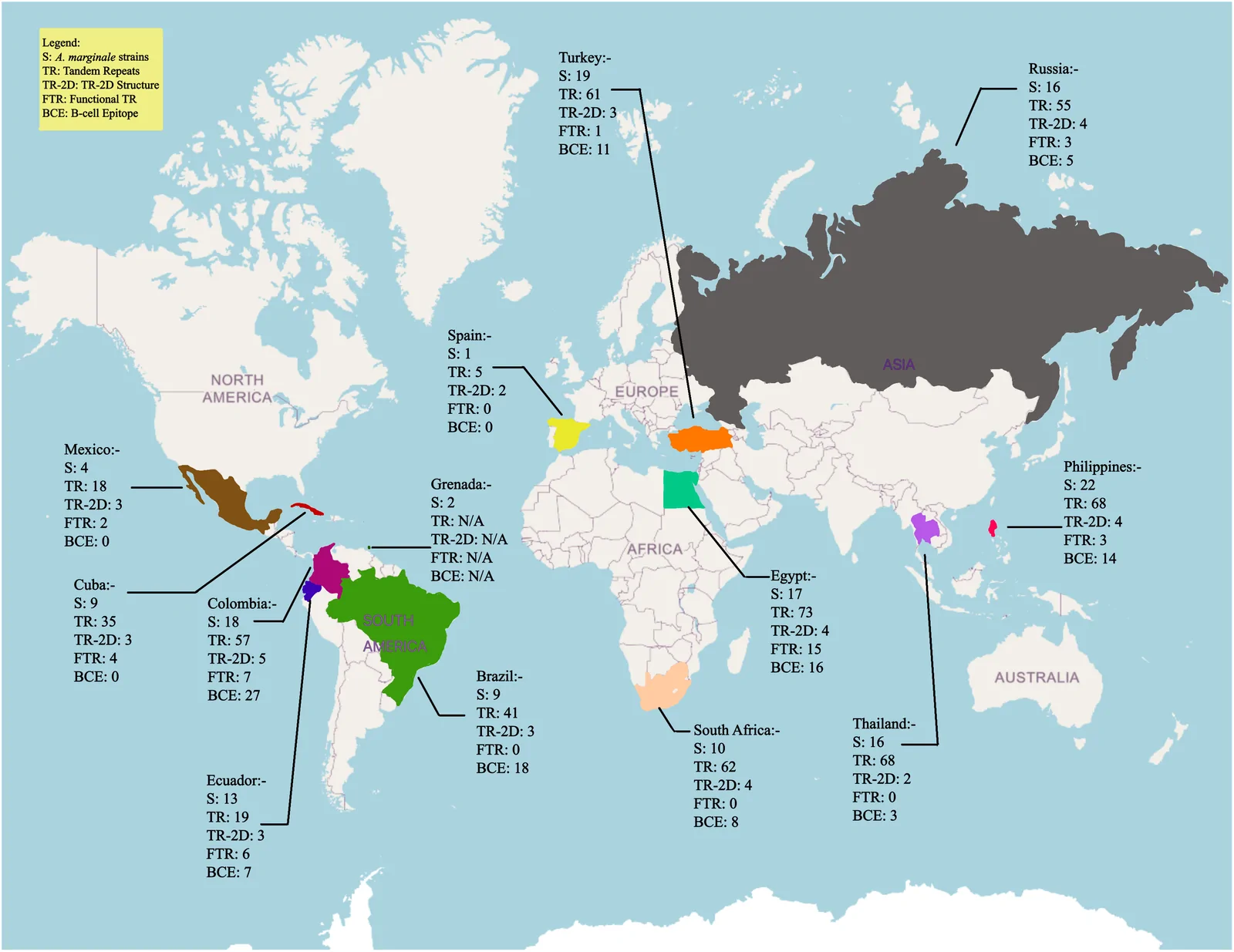
The global distribution of *A. marginale* MSP1a molecular diversity, as represented on a world map. Countries which are colour-coded are used for the indication of the geographic origin of MSP1a sequences included in the analysis. For each country, the number of analysed *A. marginale* strains (S), distinct tandem repeat types (TR), tandem repeat two-dimensional structures (TR-2D), functional tandem repeats (FTR) containing Aspartic acid (D) or Glutamic acid (E) at position 20, and B cell epitope types (BCE) are displayed. The data were compiled from MSP1a sequences retrieved from the NCBI GenBank database. Map created using The National Map Viewer (U.S. Geological Survey, National Geospatial Program) and modified by the authors.

A large number of *A. marginale* strains possessed more than one tandem repeat in their MSP1a sequence, with eight being the highest (Fig 3). The highly reported common strains with the same tandem repeats are listed in Table 1. Multiple common strains with the same tandem repeats were extracted from several countries, including Turkey; Turkey/Elazig/-(125, 126), (about eight MSP1a strains in Turkey was found with the same structure of tandem repeats), from Egypt and Cuba; Egypt/El-Wady El-Geded/-(48,15^3^,10), (about four MSP1a strains in Egypt had the same structure of tandem repeats whereas Cuba had only one strain with the same structure) from Thailand; Thailand/- (${\text{1}\text{5}\text{6}}^{\text{5}}$), (about four MSP1a strains in Thailand had the similar structure of tandem repeats) from Russia; Russia/Saratov/-$\left(\text{1}\text{5}{\text{4}}^{\text{5}}\right)$, (about four MSP1a strains in Russia had the similar structure of tandem repeats) and from the Philippines; Philippines/Cavite/- (Q, R), (about three MSP1a strains in Philippines had the similar structure of tandem repeats). In contrast, different strains with unique tandem-repeat structures in their MSP1a sequences were observed in other countries, reflecting substantial diversity. These countries included Brazil (9), Cuba (8), Ecuador (12), Egypt (13), Mexico (4), Russia (12), South Africa (7), Turkey (9), Thailand (13), with Colombia exhibiting the highest diversity (approx. 18 strains), followed by the Philippines (approx. 15 strains). Spain was the only country that showed only one type of *A. marginale* strain, i.e., Spain/Southern Spain/-($\text{5}{\text{9}}^{\text{3}}$, 115, 116) (S4 File). This may be due to fewer MSP1a sequences from Spain being uploaded to the NCBI database, although many *A. marginale*-infected cattle are found in southern regions and central Spain. The global distribution of the most common MSP1a tandem repeats is summarized in Table 2. The most common tandem repeat was the Q tandem repeat, found in 28 MSP1a strains across four countries. The tandem repeat, Q, was found to be the highest among the MSP1a strains from Ecuador (n = 10), followed by those from the Philippines (n = 7), Thailand (n = 6), and Brazil (n = 5). The second most common tandem repeat was identified as tandem repeat 15. It was found among 26 MSP1a strains across three countries. The tandem repeat 15 had the highest frequency among the MSP1a strains from Egypt (n = 15), followed by Cuba (n = 11), with a single occurrence in Colombia. Russia and Thailand were the only countries that exhibited a distinct type of tandem repeats. The tandem repeat, 154, was detected only in Russia among 24 MSP1a strains, while tandem repeats 156 and 157 were identified only in Thailand among 20 and 15 MSP1a strains, respectively. The tandem repeat 38 was predominantly found in Thailand, with minor occurrences in Cuba and Colombia. These results demonstrate that while some MSP1a tandem repeats are widely distributed, others are specifically detected in certain regions. Many tandem repeats were detected across multiple continents. The tandem repeats S and 10 were the only tandem repeats identified on four continents (i.e., the Philippines in Asia, Brazil in South America, Cuba and Mexico in North America, and Turkey in Europe, as well as Egypt in Africa).

**Table 1:** World occurrences of the most common strains of *A. marginale.*

Strains	Structure of MSP1a Tandem Repeats	Number of strains	World Occurrence
**Most Common**	125 126	8	8x Turkey
**Second Common**	48 15 15 15 10	5	4x Egypt, 1x Cuba
**Third Common**	156 156 156 156 156	4	4x Thailand
**Third Common**	154 154 154 154 154	4	4x Russia
**Fourth Common**	Q R	3	3x Philippines

**Table 2:** World occurrences of the most common tandem repeats of MSP1a strains.

Strains	Tandem Repeats	No. of times it occurs	World Occurrence
**Most Common**	Q	28	7x Philippines, 5x Brazil, 6x Thailand, 10x Ecuador
**Second Common**	15	26	14x Egypt, 11x Cuba, 1x Colombia
**Third Common**	154	24	24x Russia
**Fourth Common**	38	20	17x Thailand, 2x Cuba, 1x Colombia
**Fourth Common**	156	20	20x Thailand
**Fifth Common**	157	15	15x Thailand
	S	13	2x Philippines, 3x Brazil, 1x Cuba, 2x Mexico, 5x Turkey
	10	10	6x Philippines, 2x Egypt, 1x Cuba, 1x Colombia

**Fig 3:**
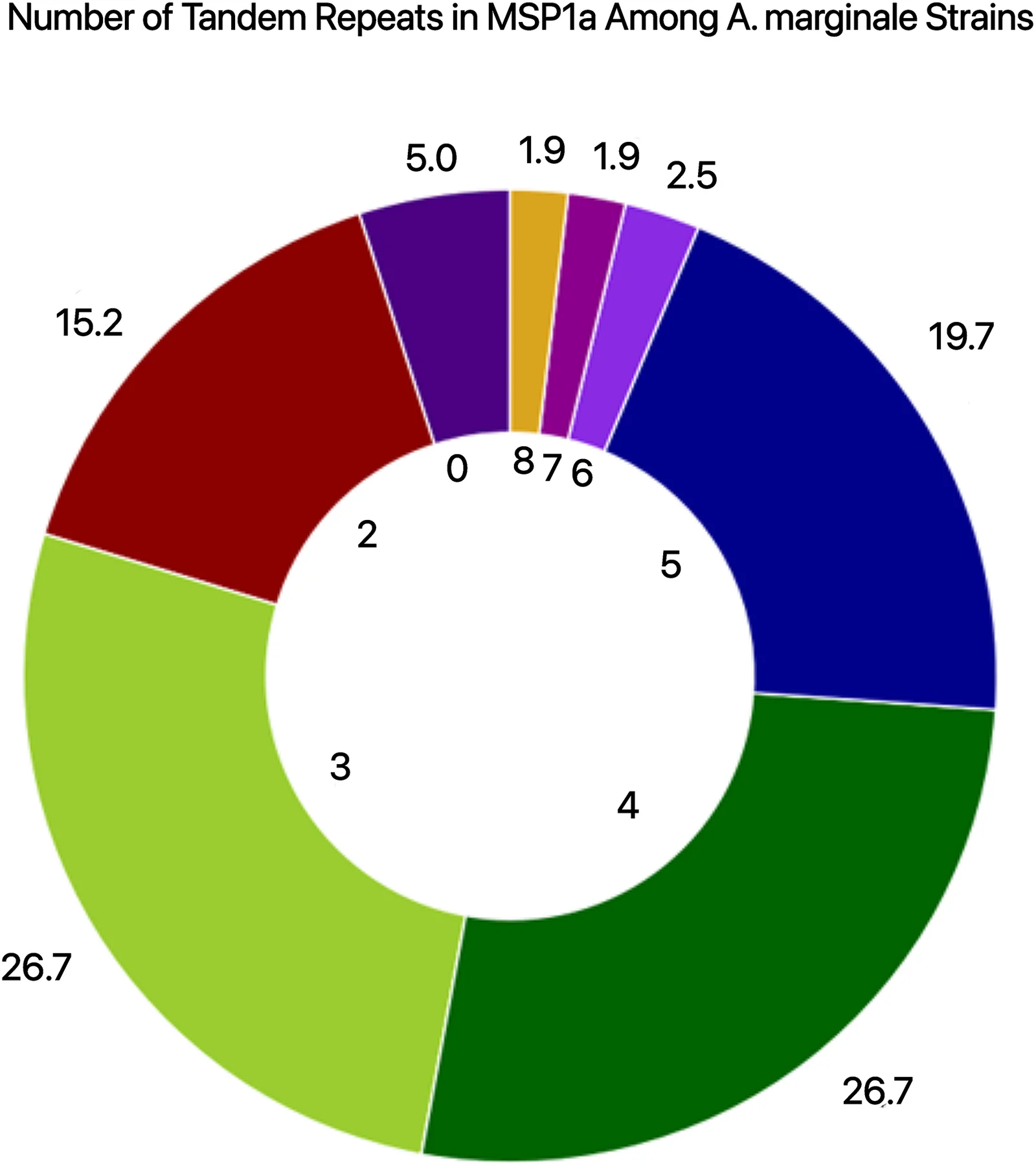
Donut diagram representing the number of tandem repeats among *A. marginale* strains. The total number of MSP1a strains classified in our study, each with a different number of tandem repeats, is illustrated in the doughnut diagram below. The percentage of the *A. marginale* strains containing different tandem repeats is shown externally, and the number of tandem repeats is shown internally. The most common MSP1a tandem repeats among strains were 2 (brown), 3 (light green), 4 (dark green), and 5 (navy blue).

### The biological effects of variations in MSP1a tandem repeat sequences

Previous studies have shown that tandem repeats at the N-terminus of MSP1a function as adhesins for tick cells and bovine erythrocytes [18–20]. Cabezas-Cruz’s research has stated that the variation in sequences of MSP1a provides insights into the multiple factors, including o-glycosylation, the ability of the *A. marginale* to bind to the ticks due to the presence of amino acids at position 20, secondary structure of proteins, relationship of *A. marginale* with the environment, and, hence, the combination of these factors contributing to its proliferation [9]. Therefore, all these factors were analyzed to gain a deeper understanding of the new MSP1a sequence dataset.

***O-glycosylation.*** From the results, it was observed that all amino acids in the MSP1a tandem repeats from positions 1–31 exhibit high variability. This suggests that the MSP1a molecule is under high evolutionary pressure. A previous study by Cabezas-Cruz found that the positions of four amino acids remained completely conserved. These included Glycine (G) in position 31, serine (S) in positions 4 and 25, and Alanine (A) in position 22. Similar variability was observed across all amino acid positions of MSP1a molecules; however, a new amino acid, Glutamine (Q), was consistently observed at positions 9 and 28 in the new dataset. The glutamine-rich regions of MSP1a may be essential for a crucial function, such as the formation of multimeric complexes between MSP1a and MSP3, thereby stabilizing MSP1a’s secondary and tertiary structure [21]. In addition, it was observed that Serine (S) has remained constant in new positions 5 (instead of position 4), 13, and in position 24 (instead of position 25). Glycine (G) has remained more constant in position 30 (instead of position 31), and Alanine in position 23 (instead of position 22).

### Presence of different amino acids at position 20 in both R1 and non-R1 tandem repeats

About 73 (38.6%) unique R1 sequences (the first tandem repeat in the MSP1a sequence) were found in all the strains. Similar to Cabezas-Cruz’s results, it was found that the non-R1 tandem repeats display more amino acid variability compared to that of R1 tandem repeats (Fig 4A). However, unlike Cabezas-Cruz’s results, it was found that only one amino acid position was conserved in both R1 and non-R1 instead of six and four, respectively, in the past datasets. The conserved amino acid is Glutamine in the 11th position of R1 tandem repeats and Glycine in the 29^th^ position of non-R1 tandem repeats. In contrast to the earlier study, these results suggest that both R1 and non-R1 tandem repeats exhibit substantially greater sequence variability than in previous datasets, and that they play equal roles in *A. marginale* infection and transmission. In addition, unlike Cabezas-Cruz’s results, the data on the non-R1 tandem repeats revealed that these repeats had many more amino acid positions, which are expected to become conserved entirely over the upcoming years due to evolution. The amino acids that become fully conserved are serine in three positions and Threonine in one position, which we learned earlier are responsible for O-glycosylation and binding to tick cells. This suggests that the function of non-R1 tandem repeats will exhibit stronger pathogenic characteristics than that of R1 tandem repeats. 92 tandem repeats consisting of 68 D20 (74.7%) and 24 E20 (26.3%) were found. 157 *A. marginale* strains contained tandem repeats with D20 or E20 at least once, and in 89 (56.6%) of these strains, D20 or E20 was found within the R1 tandem repeats. As in previous studies by Cabezas-Cruz, the highest variable amino acid position was found again at 20. This explains a higher evolutionary pressure at this amino acid position (Fig 4B). It was observed that Glycine was again the most frequent amino acid in the 20th position (Fig 4C). Also, four amino acids were found in the 20th position of the R1 tandem repeats. This included Serine [S], Glutamic acid [E], Aspartic acid [D], and Glycine [G] (lowest to highest). Seven different amino acids were found in the 20th position of the non-R1 tandem repeats (from lowest to highest frequency: Threonine [T], Asparagine [N], Alanine [A], Serine [S], Glutamic Acid [E], Aspartic acid [D], Glycine [G]). The data are available in S5 and S6 Files. Unlike Cabezas-Cruz’s results, Asparagine and Alanine are completely new amino acids to be found in the 20^th^ position. This increased variation at the 20th amino acid position, along with the overall increased variability of amino acids in non-R1 tandem repeats, explains why *A. marginale* can bind to a wide range of tick species and facilitate their transmission.

**Fig 4:**
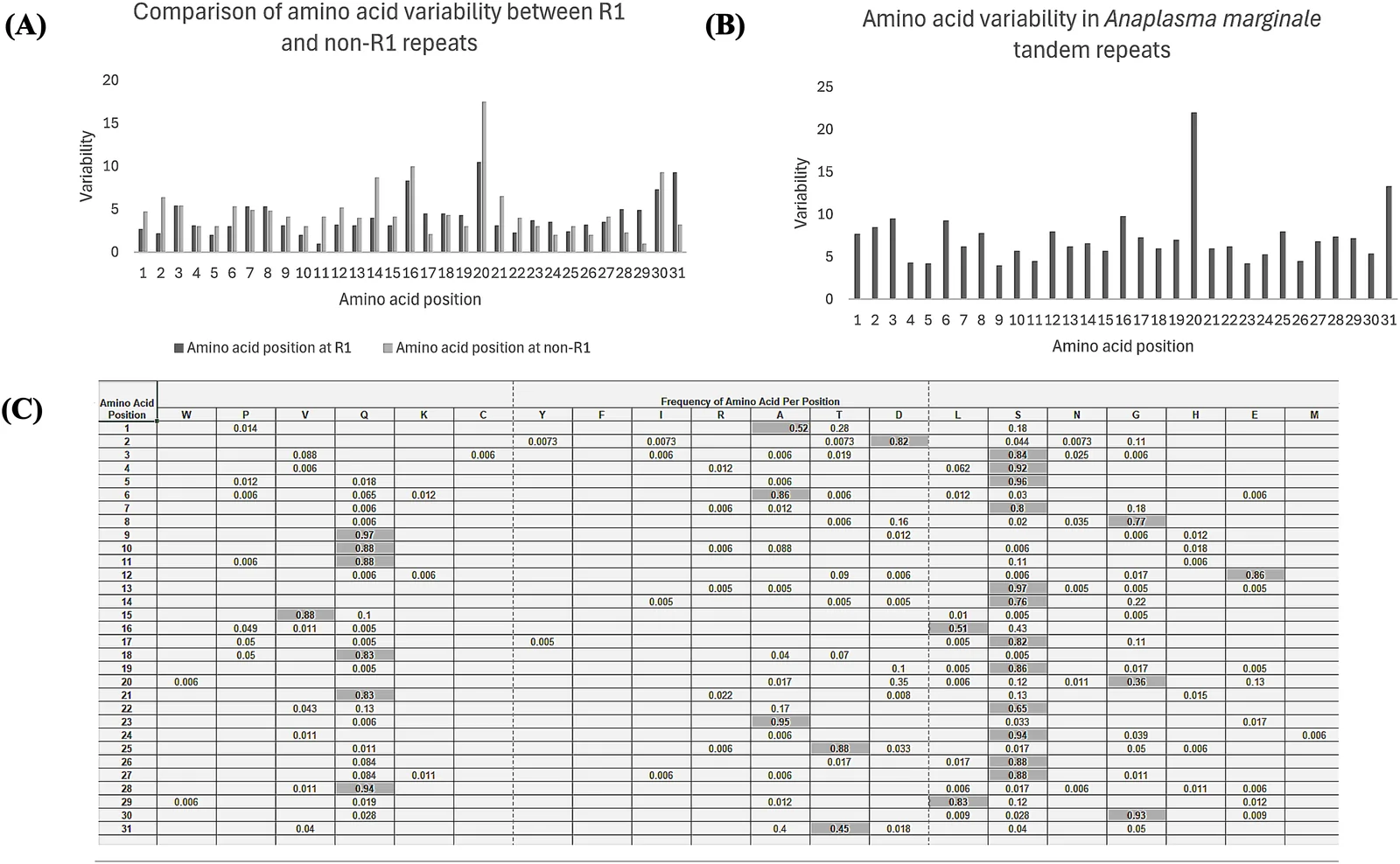
Variability and frequency demonstration of amino acids in *A. marginale* MSP1a tandem repeats. Comparison of the amino acid variability between R1 and non-R1 sequences (A), the amino acid variability of all the tandem repeat sequences (B), and Frequency (C) of the amino acid in each position were calculated. Variability was calculated using the formula, Variability = number of different amino acids at a given position divided by the frequency of the most common amino acid at that position. The one-letter amino acid code was used to identify the amino acids in (C), and the most frequent amino acids per position are coloured in grey.

**Protein conformation of MSP1a tandem repeats**. The function of the MSP1a acting as an adhesin depends on the sequence of the amino acids present in the protein as well as on the structure of that protein. Following Cabezas-Cruz’s previous study, the secondary structure of all the new MSP1a tandem repeats was also predicted (S7 File). The variability of the 189 tandem repeats in MSP1a can be demonstrated by 22 models, as indicated by the server (Fig 5). 20 alpha-helical 2D structure models with variations in the number of alpha-helices and their length were observed. The tandem repeats represented by these letters are E, F, U, Γ, π, Σ, σ, Φ, 3, 5, 19, 23, 24, 43, 57, 97, 98, 104, 125, 155. The alpha-helical 2D structures account for about 53.4% of the total 2D structure variation. Similar to Cabezas-Cruz’s study, we also observed that the amino acid at position 20 continues to determine the 2D structure of the tandem repeats. It was observed that when the amino acid is D or E at position 20, most of the tandem repeats, about 60.4%, were a-helix (Model types E, F, U, Γ, π, Σ, σ, Φ, 3, 5, 19, 23, 24, 43, 57, 97, 98, 104, 125, 155), and the rest of them were coils. When G was present in the 20th position of the tandem repeats, it was found that most of the tandem repeats, about 51.3%, were coils (Model type A), 47.2% were a-helix, and 1.4% (Model type taro) were Beta-sheet. In the previous study, four different amino acids were present at the 20th position. This included I, Y, T, and S. However, in the present study, three amino acids were present at lower frequencies at position 20 (A, N, and S). These amino acids in the 20th position demonstrated alpha-helix in their 2D structures except for the tandem repeat 89 (serine (S) in 20th position) and tandem repeat β (Alanine (A) in 20th position) displayed coils in their 2D structure.

**Fig 5:**
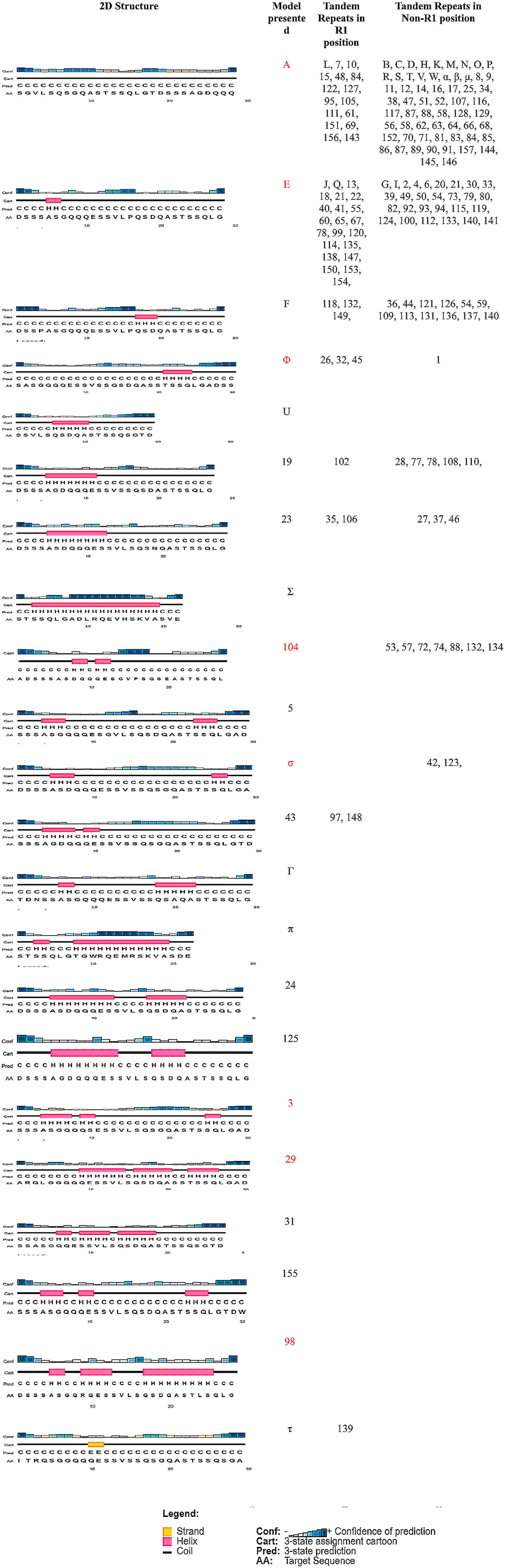
Predicted secondary structure variation among the *A. marginale* MSP1a tandem repeats. The PSIPRED server was employed to predict the secondary structures of all the MSP1a tandem repeats. Based on similarities in predicted secondary structure features, the tandem repeats were grouped into 22 distinct 2D structure models. Representative tandem repeats corresponding to each model are shown. The second column displays the IDs of the tandem repeats presented as models for each 2D structure group. The tandem repeat IDs, highlighted in red, correspond to R1 position tandem repeats (the first tandem repeat found in the MSP1a sequence).

Unlike Cabezas-Cruz’s findings, the study suggests that the 2D structures of the MSP1a tandem repeats do not consistently correlate with tick transmissibility. It was discovered that the strains transmitted by *Dermacentor sp*. contain many TCE-binding tandem repeats that form alpha-helices, beta-strands, or coiled structures in their 2D structures. However, three strains from Turkey remained transmissible by *Dermacentor sp.* despite lacking the TCE-binding tandem repeats or α-helix structures. It was observed that tick-transmissible strains have an abundance of α-helices in their 2D structures (Table 3). For example, the strains transmissible by three tick species, *R. microplus, R. annulatus*, and *H. excavatum*, possess an abundance of TCE-binding tandem repeats, also exhibiting numerous α-helices in their 2D structures. However, in contrast to Cabezas-Cruz’s results, it was found that among the tick species, *R. microplus* was able to transmit *A. marginale* strains in the absence of both TCE- binding tandem repeats and α-helix. These strains are – Brazil/Maranhao/-(A, B, C), Brazil/Maranhao/-(A, S, C, V), and Philippines/Iloilo/-(95, 54) (S8 File). The strain, which exhibited a beta strand in one of its tandem repeats, was also found to be transmissible by *R. microplus.* This opposes the view of Cabezas-Cruz, who stated earlier that the presence of the beta strand does not cause the transmission of *A. marginale* by ticks.

**Table 3:** Effect of secondary structures of the tandem repeats on tick transmission.

Strains	MSP1a tandem repeats 2D	Transmission by ticks		
		Dermacentor spp.	R. annulatus or R. microplus	H. excavatum
Brazil/Maranhao/-(A,B,C)	(c-c, c-c, c-c)	ND	Yes, (R. microplus) [22]	ND
Brazil/Maranhao/-(Q,P,**Q^2^**,R)	(a-c, c-c, a-c, a-c, c-c)	ND	Yes, (R. microplus)	ND
Brazil/Maranhao/-(A,S,C,V)	(c-c, c-c, c-c, c-c)	ND	Yes, (R. microplus)	ND
Colombia/-(σ,μ,τ)	(α-α, c-c, β-c)	ND	Yes (R. microplus)	ND
Cuba/Western Cuba/-(48^3^)	(c-c, c-c, c-c)	Yes	Yes (R. microplus) [23]	ND
Cuba/Western Cuba/-(48,**15^3^**)	(c-c, c-c, c-c, c-c)	Yes	Yes (R. microplus)	ND
Ecuador/Zamora-Chinchipe/-(99,141,57)	(**α**-c, α-c, **α**-**α**)	Yes	Yes (Rhipicephalus microplus) [24]	ND
Ecuador/Loja/-(67^6^**, 68**)	(**α****-c,** **α****-c,** **α****-c,** **α****-c,** **α****-c,** **α**-c, c-c)	ND	Yes (Rhipicephalus microplus)	ND
Egypt/El-Wady El-Geded/- (69,70,71,72)	(c-c, c-c, c-c, **β**-c)	ND	Yes (Rhipicephalus microplus & Rhipicephalus annulatus) [25]	Yes
Egypt/El-Wady El-Geded/(**15****^3^**, 8)	(**α**-**α**-**α**, **α**-**α**-**α**, **α**-**α**-**α**,α-c)	ND	Yes (Rhipicephalus microplus & Rhipicephalus annulatus)	Yes
Philippines/Iloilo/-(95, 54)	(c-c, c-c)	ND	Yes (Rhipicephalus microplus) [26]	ND
Mexico/Veracruz/-($A^{\text{2}}$, 52, 53)	(c-c, c-c, α-c, c-c)	Yes	Yes (Rhipicephalus microplus) [27]	ND
South Africa/Mpumalanga/-(135, 136, 132, 133, 136, 132, 133, 137)	(**α**-c, c-c, c-c, **β**-c, c-c, c-c, **β****-c,** **α**-c)	Yes	Yes (R. microplus) [28]	No
Turkey/Elazig/-(127, 128^2^, 129)	(c-c, c-c, c-c, c-c)	Yes	Yes (*Rhipicephalus microplus*)	ND
Turkey/Elazig/-(127,128^2^, 129)	(c-c, c-c, c-c, c-c)	Yes	Yes (*Rhipicephalus sanguineus*)	ND
Turkey/Elazig/-(A, $S^{\text{5}},\text{5}\text{8}\;$)	(c-c, c-c, c-c, c-c, c-c, c-c, c-c)	Yes	Yes (*Rhipicephalus sanguineus*) [29]	ND

TCE-binding tandem repeats are underlined—abbreviation: ND, not determined.

***Ecoregion-based pathogen-environmental relationships***. *A. marginale* was recorded in four eco-region clusters defined in our study (Table 4).

**Table 4:** Association of *A. marginale* MSP1a R1 sequences with world ecoregions.

Ecoregions	R1 sequences^(a)^	Other R1 sequences^(b)^
**Eco-region 1**: South America, involving Brazil, Southeastern Europe, and Southwestern Asia, including Turkey and Southeast Asia, containing the Philippines	E,120, 122, 125, 127,95, 97, 98, 99, 102, 104, 106, 108, 110, 111, 114, 19	A, J, L, Q, 18, 78, 84
**Eco-region 2**: Southeast Asia, involving Thailand and South Africa	155, 156, 157,130, 135, 138, 139, 143, 71, P	J, 69
**Eco-region 3**: Southwestern Europe, involving Spain, South America, containing Colombia, North America, including Cuba, Egypt in Africa, and Russia.	σ, Φ, 3, 7, 13, 21, 22, 26, 29, 32, 35, 41, 45, 81, 85, 91, 15, 48, 40,10, 75, 38	69, A, 18, 78, 84
**Eco-region 4**: North America, involving Mexico and South America, containing Ecuador	55, 60, 61, 65, 67	A, 69, Q, L

World ecoregions were constructed from a time series of NDVI values.

(a)R1 sequences were found in only one ecoregion.

(b)R1 sequences found in other ecoregions.

Eco-region Cluster 1 spans South America, encompassing Brazil, as well as Southeastern Europe and Southwestern Asia, which includes Turkey and parts of Southeastern Asia, with the Philippines being part of this region. This region exhibited the highest mean Normalized Difference Vegetation Index (NDVI) value (i.e., 42.21) with a notable seasonal decrease from June to December. All NDVI values for all countries from 2014 to 2024 are available in the S9 File. The highest recorded temperature in Eco Region 1 is 37.93 °C (S10 File), and the mean annual rainfall for Cluster 1 is 142.37 ± 104.01 mm (range: 10.38–478.71 mm), indicating rainfall heterogeneity among the countries within the cluster (S11 File). Eco-region Cluster 2 included Southeast Asia, involving Thailand and South Africa. This region also exhibited medium to high mean NDVI values (i.e., 41.34) and a distinct seasonal decrease from January to June. The highest recorded temperature in the Eco-region Cluster 2 is 43.25 °C (S12 File), and the mean annual rainfall is 87.26 ± 87.88 mm (range: 6.04–350.06 mm), indicating substantial climatic heterogeneity between the two countries in Cluster 2 (S13 File). Eco-region Cluster 3 spans Southwestern Europe, encompassing Spain, as well as South America (including Colombia), North America (including Cuba), Africa (including Egypt), and Russia. The Eco-region Cluster 3 had no distinct seasonal variation among NDVI values. The NDVI values seem to fluctuate throughout the year with minimal change compared to other clusters. This region also exhibited a moderate mean NDVI (i.e., 37.98). This region also shows higher temperatures. The highest recorded temperature in this region is 49.18 °C (S14 File), and the mean annual rainfall is 98.48 ± 100.16 mm (range: 0.05–396.8 mm), indicating substantial rainfall variability among countries in Cluster 3 (S15 File). The region’s high temperatures and variable rainfall patterns may be associated with moderate mean NDVI values of this eco-region. Finally, Eco-Region Cluster 4 spans North America, including Mexico, and South America, including Ecuador. The mean NDVI value (i.e., 34.32) for Ecoregion Cluster 4 was the lowest of all. The NDVI values of eco-region cluster 4 decreased from August to December and increased from January to August. This ecoregion cluster exhibited the lowest temperatures among all four ecoregions (S16 File) and possesses a mean annual rainfall of 122.98 ± 90.64 mm (range: 8.53–532.26 mm), also indicating considerable variability in rainfall patterns among the countries of cluster 4 (S17 File). The results of the current study showed that approximately 74.2% of the MSP1a unique R1 sequences were associated with a single eco-region cluster. Seventeen R1 unique sequences (30.8% of the total number of R1 sequences) were reported in Eco-region cluster 1. These sequences exhibited about 11 out of 31 identical amino acids (35.5% of the total number of amino acids). In eco-region cluster 2, about ten unique R1 sequences were observed (17.3% of the total number of R1 sequences). These sequences shared about 15 of 31 amino acids (48.4% of the total). In eco-region cluster 3, about 22 unique R1 sequences were detected (42.3% of the total number of R1 sequences). These sequences contained only 2 out of 31 identical amino acids (6.5% of the total number of amino acids). In eco-region cluster 4, only five unique R1 sequences (9.6% of the total number of R1 sequences) were found. These sequences contained 23 of 31 identical amino acids (74.2% of the total). Eight R1 sequences were seen multiple times among the eco-region clusters (Table 4). The analyses of the results reveal that, with increases in temperature and variability in annual rainfall (in ecoregion 1, 2, and 3 clusters), the presence of unique MSP1a R1 sequences has also increased. It was also observed that lower temperatures in the ecoregion 4 cluster were associated with fewer unique R1 sequences, despite higher mean annual rainfall and rainfall variability. Therefore, it could be concluded that the clustering of the MSP1a R1 sequences is based on abiotic (climate) factors. It was observed that higher variation of R1 sequences was found in ecoregion 3 clusters. This region involves Spain, Cuba, Colombia, Egypt and Russia. It was observed that many tick species contribute to the transmission of the numerous MSP1a strains in Colombia, Cuba, and Egypt. Similar results were obtained for the ecoregion 1 cluster, which encompasses Brazil, Turkey, and the Philippines. Exceptionally, only one MSP1a strain is reported in Spain, but no tick species has been identified in this country. All results from the Principal Component Analysis for classifying countries into different eco-region clusters are based on their NDVI values (S18 File).

Early reports by Cabezas-Cruz suggest that *A. marginale* strains are associated with factors influencing the effectiveness of tick vectors [9]. Hence, similar dN/dS index calculations were carried out on the MSP1a R1 sequences from our newly selected regions, representing the Cluster 1, Cluster 2, Cluster 3, and Cluster 4 ecoregions, to determine how ticks are performing in association with *A. marginale* in our selected regions of interest. It was discovered that the dN/dS index calculation for the MSP1a R1 sequences in Eco-region Cluster 1–4 was 0.87, 1.32, 0.844, and 2.60, respectively. The dN/dS index calculations for all the eco-region clusters are presented in S19–S22 Files.

### Phylogenetic correlation analysis of tick transmissible strains with their 2D structures and TCE binding tandem repeats

Almost all tandem repeats from the tick-transmissible MSP1a strains from different countries were extracted and included in the phylogenetic analysis. The purpose of constructing this phylogenetic tree of the tandem repeats from various countries was to explore global phylogenetic and structural patterns among MSP1a tandem repeats derived from tick-transmissible strains, after 11 years of the study carried out by Cabezas-Cruz, and to determine whether the cluster formation of the tree was based on amino acid 20 of the tandem repeats, as suggested by Cabezas-Cruz in his previous study. A phylogenetic correlation of the tick transmissible tandem repeats with their 2D structure and the TCE-binding tandem repeats (Fig 6) was explored. The tandem repeats are denoted as tick-transmissible tandem repeats, as all the tandem repeats were derived from the MSP1a strains in NCBI, and the tick transmissibility information of each of the MSP1a strains was derived from the original papers that were referenced with each of the MSP1a strains in NCBI records. These papers mentioned that all the MSP1a strains were isolated from ticks or tick-transmitted hosts. No non-tick transmissible tandem repeats could have been involved in this phylogenetic analysis because none of our MSP1a strains was found to be non-transmissible in the NCBI records. Like Cabezas-Cruz’s study, the phylogenetic tree analysis revealed that all tandem repeats formed distinct clusters based on the presence of other amino acids in the 20th position. It was observed that tandem repeats containing D (Aspartic acid) at the 20th amino acid position formed alpha helices in their 2D structures. Hence, it was named the Cluster α-1. Again, those tandem repeats containing D at the 20th amino acid position exhibited only coils as their 2D structures, such as the Cluster c displayed by Cuba 48, Cuba 51, Colombia 16, and Cuba 15. Another cluster exhibited by the Philippines 110 and Philippines 130 indicated that when the 20th amino acid was Glycine, their 2D structure also formed alpha helices, contradicting the previous studies held by Cabezas-Cruz, which claimed that the presence of Glycine in the 20th position generates mainly beta sheets and, as a result, the tandem repeats become non-transmissible. To statistically analyze whether the cluster formations in the phylogenetic tree were associated with the amino acid located at position 20, a permutation-based cluster association test was carried out. The association of the phylogenetic clusters with the amino acid at position 20 is presented in the S23 File and Table 5. The calculated value for the observed chi-square statistic was found to be χ^2^ = 6.63, and the permutation p-value was observed to be 0.681 (p > 0.05). suggesting that the clustering of the tandem repeats in the phylogenetic tree based on the 20^th^ position of the amino acid is not statistically significant. This indicates that these patterns may arise by chance. Following that, another statistical test, such as a permutation-based chi-square test, was conducted to determine whether the amino acid at position 20 was associated with the predicted secondary structures of the tandem repeats, such as alpha helices or coils. The distribution of the amino acid at position 20, along with its predicted secondary structure, can be viewed in the S24 File and Table 6. The calculated value for the observed chi-square statistic was found to be χ^2^ = 1.64, and the permutation p-value was observed to be 0.469 (p > 0.05). This indicates that the secondary structure is not significantly associated with the amino acid at position 20. Although most clusters in the phylogenetic tree exhibit distinct amino acids at position 20, which are associated with different secondary structures in the original data, the statistical tests reveal no significant associations. In the current analysis, it was observed that the tandem repeats with Glycine at position 20 did not form beta-sheets. This trend indicates that the amino acid at position 20 does not strictly determine beta-sheet formation, and that other factors likely contribute to the structural diversity of tick transmissible MSP1a tandem repeats. This may reflect evolutionary pressures throughout the decade that favored certain structural features, such as alpha helices or coils, in tick-transmissible strains; however, there is no statistical support for a correlation between the amino acid at position 20 and secondary structure.

**Table 5:** Association of the phylogenetic clusters with the amino acid at the 20^th^ position of tandem repeats.

Cluster	Aspartic Acid (D)	Glycine (G)	Serine (S)	Glutamic acid (E)
α-1	6	5	1	3
α-2	2	1	0	0
c	1	4	1	0
β-α-c	0	1	0	0

**Table 6:** Association of the amino acid at the 20^th^ position of the tandem repeat with its secondary structures.

Amino acid at the 20^th^ position	Alpha-helix	Coils
Aspartic acid (D)	23	15
Glycine (G)	17	20
Glutamic Acid (E)	5	5

**Fig 6:**
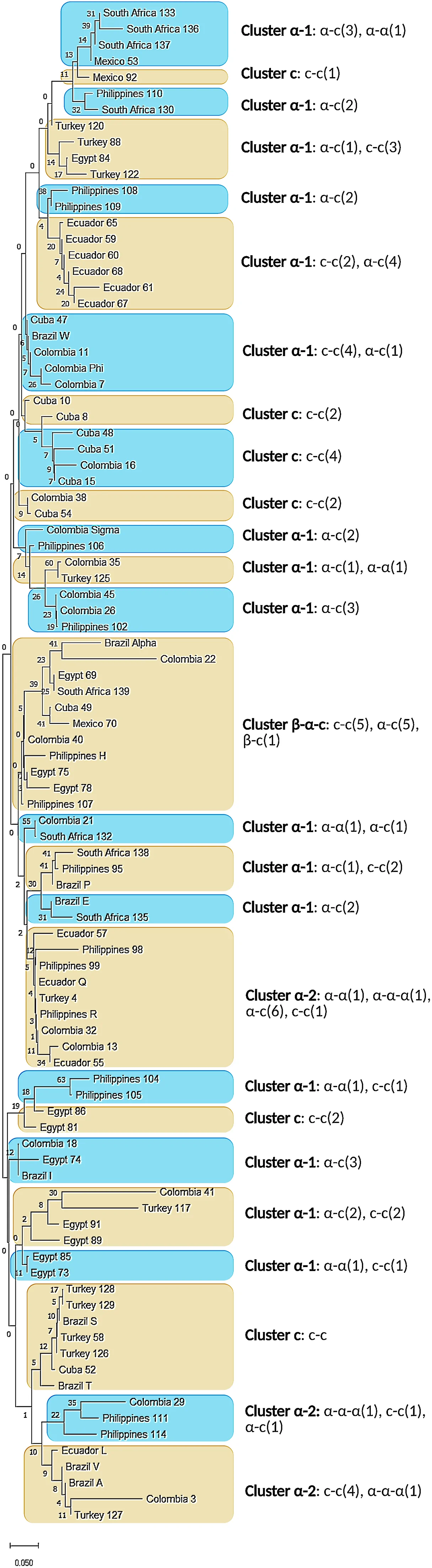
Phylogenetic tree based on MSP1a tandem repeat amino acid sequences. The tandem repeats of MSP1a sequences from tick-transmissible strains were used in the phylogenetic analysis. The phylogenetic tree was constructed using the neighbour-joining and maximum likelihood methods. The bootstrapping method was adjusted to 1000 bootstrap replicates. The bootstrap values are displayed as percentages in external branches. The tree displays 25 phylogenetic clusters, each containing distinct patterns of 2D MSP1a tandem-repeat structures.

### Analysis of B-cell epitopes in MSP1a tandem repeats

A major challenge is designing and developing vaccines that provide enhanced cross-protective immunity against the diverse *A. marginale* strains worldwide, with a focus on the highly variable outer membrane protein MSP1a. However, due to a conserved neutralization-sensitive B-cell epitope within MSP1a at positions 20–26 of the tandem repeats, MSP1a has been identified as a potential vaccine candidate by researchers. According to a 2013 paper by Cabezas-Cruz and de la Fuente, a B-cell epitope containing the linear sequence SSAGGQQQESS, at positions 4–14 in the tandem repeat of non-tick transmissible strains, was identified as immunodominant by the early researchers. Therefore, Cabezas-Cruz characterized the diversity of the specific B-cell epitope at positions 4–14 among available MSP1a strains of *A. marginale* using bioinformatic tools during that period. Following this study, the B-cell epitopes in our tandem repeats were predicted using similar bioinformatic tools to assess their diversity over the last 11 years. All predicted B-cell epitopes from the tandem repeats, along with their BCPRED scores, antigenic scores, and transmembrane location, were also assessed (S25 File).

It was observed that our B-cell epitopes exhibited high sequence variability, and none of our B-cell epitopes was found to be conserved in all of our tandem repeats. Most of the predicted B-cell epitopes in the tandem repeats also have higher prediction scores, suggesting a higher likelihood of being recognized as B-cell epitopes. All 189 tandem repeats of the MSP1a sequences were involved in the prediction of B-cell epitopes. 72 sequence variants of B-cell epitopes were identified. All 72 B-cell epitope variants, along with their corresponding country locations, are listed in the S26 File. Of these 72 variants, only 25 B-cell epitopes were found to be located inside the MSP1a molecule, and about 47 B-cell epitopes were found to be surface-exposed. Four of the B-cell epitope types that were similar in sequence to the model sequence, SSAGGQQQESS, accounted for 24.2% of the total B-cell epitope variability in the present dataset, compared to 64% in the earlier studies by Cabezas-Cruz. The type 4 B-cell epitope was found to be entirely new (Fig 7A and 7B). All five B-cell epitope variants formed two phylogenetic clusters (Fig 7C). As in the previous study, the model sequence, Type 2, and Type 4 shared the same antibody recognition sites. In contrast, Type 1 and Type 3 B-cell epitope sequences shared the same antibody recognition sites. As predicted by the new version of the Deep TMHMM server, the model B-cell epitopes, Types 1, 3, and 4, are located inside; however, in previous studies, the same server predicted these B-cell epitope types to be located outside. The new version of the Deep TMHMM server incorporates improved algorithms and more accurate training data, resulting in revised topology predictions. Only the Type 2 B-cell epitope was predicted to be surface-exposed. Similar to Cabezas-Cruz’s study, Type 1 B-cell epitopes were found to be present in greater abundance than the other B-cell epitopes in our study. However, the percentage is 20.1%, compared with Cabezas-Cruz’s study, which reported 40.83%. Upon using the Vaxijen server, it was found that the Type 2 B-cell epitope had the highest antigenic score among all B-cell epitope types and was also higher than the model sequence. However, the percentage of Type 2 B-cell epitope was only 3.2% of the total tandem repeats. The Type 1 and Type 3 B-cell epitopes displayed lower antigenic scores than those of the model sequence. In comparison, Type 2 and Type 4 B-cell epitopes exhibited antigenic scores higher than those of the model sequence. These four B-cell epitope types possessed higher antigenic scores than the average of all 72 B-cell epitope types. It is also assumed that the type 2 and type 4 B-cell epitopes have similar antibody recognition sites as the model type B-cell epitope. In contrast, the type 1 and type 3 B-cell epitopes possess similar antibody recognition sites. Unlike Cabezas-Cruz’s results, no strong correlation was found between the Vaxijen scores and the BLASTp scores (Fig 7D) in our analyzed sequences. This suggests that the similarity of B-cell epitope sequences to the model type B-cell epitope is not strongly associated with predicted antigenicity in the present datasets. All the BLASTp scores of all the B-cell epitopes predicted from each of the MSP1a tandem repeats generated against the model sequence, SSAGGQQQESS, could be observed in the S27 File. The alignment of the predicted B-cell epitope sequences with the model sequence yielded robust bit scores ranging from 28 to 34, confirming high sequence conservation across the short peptide fragments and validating the structural presence of the immunodominant epitopes in the reference model. These four types of B-cell epitopes were then used to examine the correlation between their VaxiJen and BCPREDS scores (Fig 7E). Unlike Cabezas-Cruz’s results, a weak positive linear correlation was observed between the two variables in the present dataset. The results suggest that the predicted antigenicity of B-cell epitopes tends to increase slightly with higher prediction scores; however, the relationship is not strong. The findings suggest that epitopes predicted to be B-cell epitopes may not necessarily be more antigenic. It was also observed that a weak positive linear correlation graph exists between the BLASTp scores and the BCPRED scores, which demonstrates that as the B-cell epitope prediction scores slightly increase, their BLASTp scores also increase slightly but do not have a strong relation between them (Fig 7F). The results from the present dataset suggests that the epitopes that were more similar to the model B-cell epitope sequence does not necessarily correspond to higher b-cell epitope prediction scores. A relationship between these two graphs could be suggested: Unlike Cabezas-Cruz’s result, the B-cell epitopes with greater sequence similarity to the model sequence (homology) do not necessarily exhibit similar antigenic properties across the analyzed sequences.

**Fig 7:**
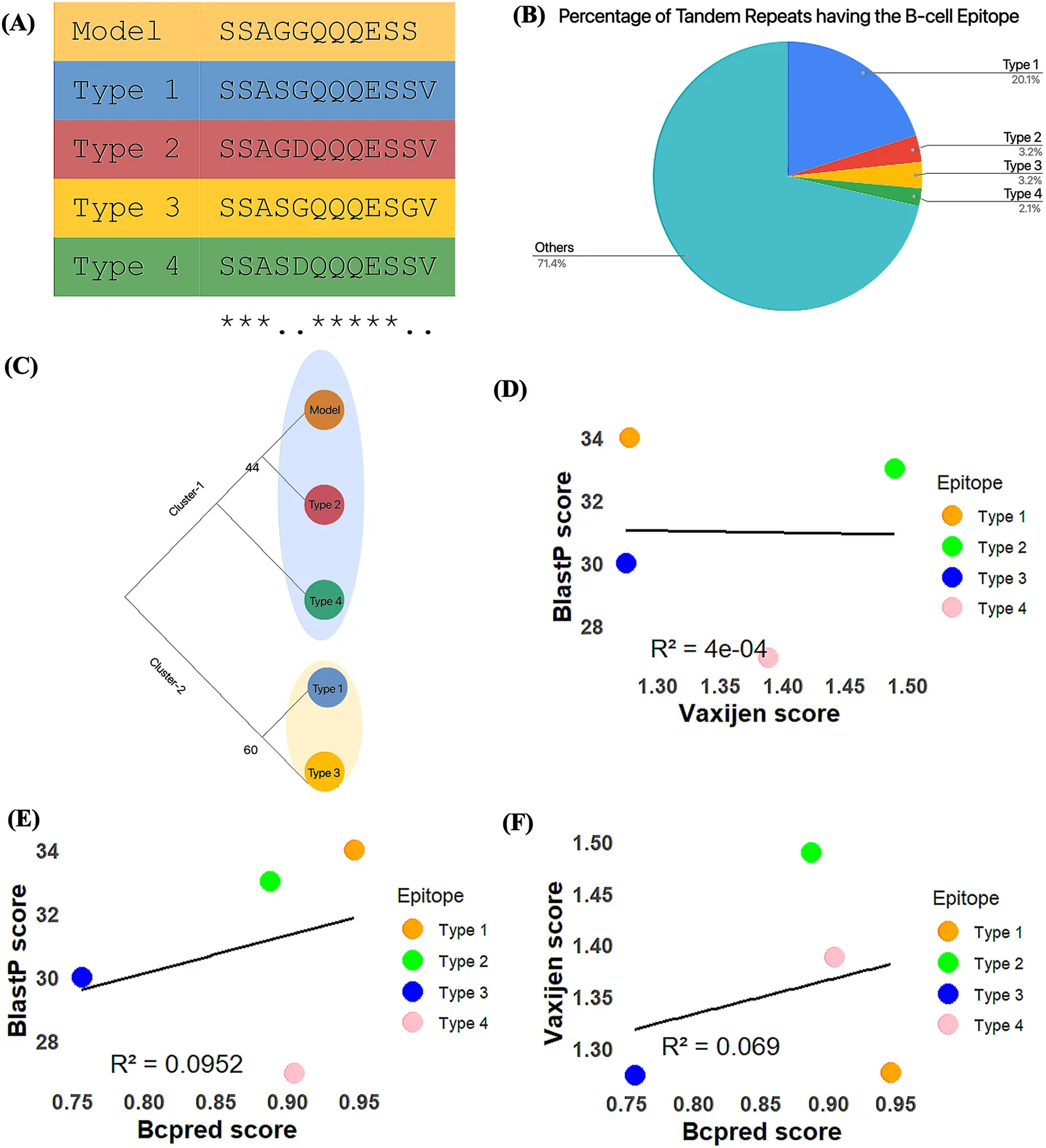
Analysis of B-cell epitopes in *A. marginale* MSP1a tandem repeats. The BCPRED server was used to predict the B-cell epitopes. The B-cell epitope SSAGGQQQESS was used as a model sequence for comparison. (A) Multiple sequence alignment and changes in amino acids within the four more illustrated MSP1a tandem repeat B-cell epitopes. The types of B-cell epitope models are Type 1 (blue), Type 2 (red), Type 3 (yellow), and Type 4 (green). (B) A pie chart illustration representing the percentage of tandem repeats containing each type of B-cell epitope. (C) Neighbour-joining phylogenetic tree created from the five represented B-cell epitopes, based on the B-cell epitope sequences. Cluster-1: Types Model, 2 and 4, and Cluster-2: Types Model, 1 and 3. VaxiJen vs BLASTp correlations (D), BCPRED vs BLASTp scores correlations (E), and VaxiJen vs BCPRED scores correlations (F). No correlation was found between the Vaxijen scores and the BLASTp scores. A weak correlation was observed between BCPRED and BLASTp scores, as well as between Vaxijen and BCPRED scores. This demonstrates that B-cell epitopes with greater sequence similarity to the model sequence (homology) are predicted to be less effective and therefore lack the same antigenic properties.

## Discussions

In this research, an updated nomenclature classification of *A. marginale* strains based on tandem repeat sequences of the outer membrane protein MSP1a has been carried out. This research was conducted following the study by Cabezas-Cruz and de la Fuente [9] in 2013. The tandem repeats were selected to decipher the nomenclature of the new *A. marginale* strains from 13 countries uploaded between 2013 and 2023, which were not included in Cabezas-Cruz’s earlier study. The current research has revealed new tandem repeats, changes in serine positions, variation in tandem repeats across different geographic locations, a lack of homogeneity among surface-exposed B-cell epitopes, and structural changes that do not consistently correlate with transmission, which is determined through bioinformatic analysis. During the analysis, some tandem repeats appeared to be unique to a single country; repeat 154 was found in 24 strains, all originating from Russia. Many southern regions of Russia, including Moscow, Kaluga, and Saratov, are experiencing bovine anaplasmosis due to the expansion of tick habitats. These regions exhibit favorable climatic conditions for tick production, such as the Moscow region, which has a humid continental climate. Moreover, Russia has strict regulations that restrict the entry of imported infected cattle from various geographic locations. All the animals are tested for anaplasmosis. The animals that test negative for the disease are vaccinated. Those animals that test positive for infection are denied importation by the Russian government. This gives rise to new MSP1a strains across different geographical regions within the nation due to their movement, resulting in unique variants found only in Russian samples. The selective pressures exerted by the host immune system and host-parasite interactions might explain the evolution of new sequences specific to the region [30]. Countries in South America, such as Brazil and Colombia, exhibited a large number of unique tandem repeat types, with Colombia having the highest number (43 TR). In Asia, the highest number was found in the Philippines, which exhibited 19 unique tandem repeat types. Russia and Spain had the fewest unique tandem repeats. As observed from the results, Colombia possessed the highest number of unique MSP1a strains and tandem repeats. This could be explained by the fact that Colombia is the third-largest livestock producer in South America, and *A. marginale* is endemic at altitudes lower than 1000m due to the wet climatic conditions of Colombia. There are several possible reasons for the origin of the new MSP1a sequences in Colombia, including abundant ticks and flies during the late or early wet season, excessive use of insecticides and acaricides, invasion of tick populations during seasonal changes, and continuous importing of cattle from various geographical locations, which caused the MSP1a strains of *A. marginale* to diversify leading to their persistence in Colombia and also mainly due to the immune selection by the different species and breeds of the host [31]. The Philippines is found to have the second-highest number of unique MSP1a strains, the highest in Asia, and a large number of uncommon tandem repeats. This may be due to the country’s unique geography, which consists of multiple islands. Also, the Philippines has a higher rate of *A. marginale*-infected water buffaloes. These water buffaloes live in close contact with the cattle, making them more susceptible to infection. Livestock trade across the different islands of the Philippines has increased the diversity of MSP1a strains [25]. These regional patterns highlight that both vector ecology and livestock management practices may play significant roles in shaping the genetic landscape of *A. marginale*.

As suggested by Cabezas-Cruz, when the glycosylation of serine in the tandem repeats of the MSP1a molecule takes place, the ability of the MSP1a molecule to bind to the tick cells increases; and, after deglycosylation, the binding capacity is decreased. Although our dataset shows that serine positions are present at the new 5th and 24th positions, a new serine position has been identified at the 13^th^ position in the tandem repeat sequences we analyzed. Three highly recurring sites, S5, S24, and S13, within the tandem repeats may indicate potential O-glycosylation sites in the analyzed sequences. Previous studies state that amino acids aspartic acid (D) and glutamic acid (E) at position 20 are necessary for MSP1a binding to the tick cell extract, a biological preparation derived from tick cells. They also note that in the presence of glycine (G) at position 20, MSP1a is unable to bind to the tick cell extract. During the analysis of the R1 and non-R1 tandem repeats, Cabezas-Cruz found only Glycine to be the most frequent 20^th^ amino acid in both the R1 and non-R1 tandem repeats. However, in our analyzed sequences, we found that two amino acids, Glycine (G) and Aspartic acid (D), were present in equal amounts at the 20th position in both R1 and non-R1 tandem repeats.

Secondary structure analysis of the 189 MSP1a tandem repeats among global *A. marginale* strains suggests that the amino acid at position 20 may be associated with secondary structure preferences. Our study demonstrates that Aspartic acid (D) and Glutamic acid (E) at position 20 are related to α-helical conformations, whereas Glycine (G) is predominantly associated with a mix of coils and, rarely, beta-sheets. Nevertheless, as the dataset does not involve any non-tick-transmissible strains, it is impossible to establish a causal relationship between MSP1a secondary structure and tick transmissibility. However, it is essential to note that some of the tick-transmissible strains lacked TCE-binding tandem repeats, or α-helices, and *Rhipicephalus microplus* could transmit strains even with beta-sheets or in the absence of helical structures. These exceptions emphasize the complexity of vector-pathogen interactions and suggest that additional factors may contribute to the efficient transmission of ticks. Moreover, the phylogenetic tree permutation-based chi-square analysis demonstrates that the clustering and structural patterns of MSP1a tandem repeats are not influenced by the presence of a specific amino acid at position 20, which alone does not determine the secondary structure.

Numerous studies have demonstrated that *A. marginale* transmission is influenced by both vector species and strain-specific pathogen properties. The research conducted by Ueti and Scoles showed that the transmission of *A. marginale* is reduced due to the presence of structural barriers in ticks, such as the midgut and salivary glands [32], and the variations in the tandem repeats of the outer membrane protein, MSP1a, are responsible for the binding of *A. marginale* to both vertebrates and ticks, thus affecting the binding affinity [33]. Therefore, the secondary structure analysis, along with their reported tick-transmission data, in the present study may reflect adaptive changes in MSP1a that enable efficient pathogen colonization of these barriers and their transmission, even in the absence of classical TCE-binding motifs. This may help explain the observed transmission of structurally divergent strains by competent vectors. Nevertheless, these assumptions are limited by the lack of functional validation, and further experimental studies are required to clarify the direct role of MSP1a structural variation in vector competence.

Although countries have distinct microclimates and microhabitats, incorporating the ecoregion classification remains highly relevant because tick populations depend strongly on environmental factors such as humidity, rainfall, vegetation cover, and temperature. In 2022, scientists conducted a study to determine the effects of climatic stress on ticks. It was observed that tick vectors die faster in warmer, drier conditions due to water loss than in cooler, more humid conditions. They lost about 50–55% of their total water content before they died [34]. In another study, scientists conducted research on 300 females of *Rhipicephalus microplus* ticks, showing that the ticks died in cold temperatures, such as 4°C, after three days and cooler temperatures, such as 20°C, with 80% humidity, reduced their egg development, while the ticks in 28 °C temperatures with 80% humidity exhibited the best reproductive capacity [35]. It is well established that NDVI values are primary indicators of tick habitats and correlate positively with tick survival. NDVI values express the greenness, density and the health of vegetation. Rainfall and high temperatures promote the growth of these vegetations. Regions with higher vegetation cover and a more humid microclimate exhibit higher NDVI values [36]. Moreover, denser vegetation creates a higher-humidity microclimate that prevents ticks from desiccating. This leads to an increase in the tick population. On the other hand, regions with lower NDVI values exhibit sparse vegetation, low humidity, and high temperatures. These conditions make them uninhabitable for tick survival because of extreme water loss under dry conditions [37]. Importantly, the *A. marginale* strains that were included in this study were originally collected from cattle of certain locations where competent tick vectors exist, and transmission was actively occurring. Ticks suck blood from cattle and other small mammals, such as rabbits, mice, and squirrels, etc., to obtain the nutrients required for their growth and development. Depending on the tick species and environmental conditions, they develop from larvae to nymphs, then to adults. The larvae and nymphs primarily feed on small mammals, whereas the adults generally feed on larger hosts, such as cattle, before falling off and returning to their favourable habitats to lay eggs [38]. Therefore, although country-level precipitation averages cannot capture the considerable microclimatic variation within countries or even within individual collection regions, the *A. marginale* strains are likely to originate from ecological settings that share the broad climatic characteristics reflected in NDVI, temperature, and rainfall data. Hence, the approach to ecoregion classification to understand MSP1a diversity in relation to large-scale environmental patterns is highly reasonable and necessary. Clustering countries into different ecoregions based on principal component analysis of NDVI values helps identify broad-scale ecological patterns associated with MSP1a diversity among *A. marginale* strains. Broad environmental clustering may reveal macroecological patterns underlying MSP1a diversity. In 2008, research was carried out to determine the distribution of tick species at a continental scale (Africa). The researchers divided the whole continent into ecoregion clusters based on NDVI values. NDVI-based clustering identified ecological regions for tick populations that correspond to climatic conditions relevant to ticks. The researchers were also able to analyze and interpret the ecology of ticks across different ecoregion clusters, as distinct tick populations occupy distinct environmental niches, unlike other analyses conducted across highly heterogeneous geographic regions. The paper also demonstrated that broad-scale environmental clustering can reveal regional ecological patterns even when local conditions vary [39]. Another study conducted in 2016 also highlighted the importance of using NDVI values at broad spatial scales to characterize tick ecological niches rather than traditional interpolated climate datasets, thereby enabling the investigation of tick-associated *A. marginale* distributions using broad-scale environmental gradients [40].Therefore, it is biologically plausible to observe the association of ecoregion clustering with R1 sequence diversity among MSP1a strains, which is consistent with the ecological requirements of tick vectors and their habitats. The study shows that both Ecoregions 1 and 2 exhibit the highest and the second-highest mean NDVI values, respectively, indicating greater vegetation in these regions. The increase in vegetation might favor stable ecological conditions in tick habitats, leading to higher tick populations and improved survival. The increased tick populations allow an increased tick transmission among hosts, which in turn provides greater chances of variation among multiple MSP1a strains through mutation, resulting in elevated MSP1a sequence diversity (17 unique MSP1a R1 tandem repeats in Ecoregion 1 and 10 unique MSP1a R1 tandem repeats in Ecoregion 2). However, the relatively stable ecological conditions likely limit diversifying selection in these ecoregions, as MSP1a diversity does not reach the levels observed in ecoregion 3. The ecoregion 3 exhibits moderate mean NDVI values. Although the overall mean NDVI was moderate, Ecoregion 3 shows fluctuations in NDVI. The fluctuations in NDVI values reflect periods during which the vegetation in these areas appears, grows, declines and reappears throughout the year. Such vegetation dynamics may create highly fragmented, heterogeneous, and temporally unstable ecological conditions within tick habitats, resulting in rapid increases and subsequent declines in tick populations. This ecological instability might then cause new MSP1a strains of *A. marginale* to undergo diversifying selection, facilitating the emergence of adaptable variants with high sequence turnover and increasing the number of unique R1 tandem repeats (i.e., 22). A similar effect was reported in another study by Estrada-Peña et al. [41], which found moderate, fluctuating NDVI values. Ecoregion 4 exhibits the lowest mean NDVI among the ecoregions. The lowest NDVI value indicates less vegetation year-round, which reduces the tick population. As tick populations decline, tick transmission and survival decline, resulting in lower MSP1a diversity. Hence, only five unique MSP1a R1 tandem repeats are observed in this ecoregion. Therefore, it could be suggested that the clustering of R1 sequences depends on both the tick vector species and the tick vector’s infectivity level in the ecoregion. In agreement with previous findings by Cabezas-Cruz et al., the distribution of MSP1a R1 sequences observed across the four eco-regions suggests a link between environmental clustering and genetic diversity.

The dN/dS index calculations were performed to understand pathogen-environment relationships across the 13 countries. It suggests that Eco-region Clusters 1 and 3 are under negative selection. In contrast, Ecoregion Clusters 2 and 4 seem to be under positive selection. Negative selection occurs when the factors in these cluster regions eliminate the deleterious genes from the *A. marginale* population. The less likely-to-survive genetic strains are being eliminated, thereby reducing the overall genetic diversity of *A. marginale* strains originating from these regions. The positive selection of the MSP1a R1 sequences in Cluster 2 and Cluster 4 regions implies that the MSP1a protein of *A. marginale* in these regions is evolving under adaptive pressures. The selective forces driving this evolution remain unclear but may include interactions with tick vectors, host immune responses, or other environmental factors.

A primary objective of this study was to investigate the diversity and the predicted antigenic properties of B-cell epitopes within the tandem repeats of the MSP1a protein of *Anaplasma marginale* over the past 10 years, with particular attention to the previously identified immunodominant epitope SSAGGQQQESS (Cabezas-Cruz and de la Fuente, 2013). Our findings indicate a significant shift in the distribution and sequence composition of B-cell epitopes, as well as a high degree of variation, in the analyzed MSP1a strains compared with previous reports. Of 47 surface-exposed B-cell epitope variants, about 29 (40.2%) different B-cell epitope variants were located in 29 different tandem repeats of different MSP1a strains. About 71.4% of the total 72 B-cell epitope variants were utterly different in sequence and were situated on different tandem repeats and did not exhibit homogeneity among themselves. This highlights a significant change, as evident in comparisons with previous studies, indicating higher diversity of MSP1a sequences in the *A. marginale* species. The lower percentage of similar sequences to the model epitope (24.2%) compared with previous reports (64%) suggests greater variation in MSP1a B-cell epitopes among the analyzed sequences. Interestingly, although some of the epitope variants (Types 1 and 3) share antibody recognition sites with the model sequence, they are now predominantly predicted to be internal (i.e., not surface-exposed). The results contrast with those of previous studies that used older TMHMM prediction tools, highlighting how updated computational models of the same prediction tool have enhanced our understanding of protein topology. Only the Type 2 B-cell epitope was predicted to have the highest antigenic score and to be present on the outer surface of MSP1a, but with a low prevalence (3.2%). However, the low prevalence of these epitopes may limit their suitability for inclusion in broadly protective *in silico* vaccine designs. It is also observed that the previously reported relationship between sequence similarity and predicted antigenicity is not evident in the present dataset. Unlike previous findings, the correlation between antigenic scores (predicted by the VaxiJen server) and BLAST similarity to the model B-cell epitope was weak. Similarly, the correlation between the antigenic scores (predicted by the VaxiJen server) and the BCPRED scores was also weak. This suggests that sequences homologous to the model epitope are not strongly associated with predicted antigenicity in the present dataset. Moreover, the weak positive correlations observed between BCPREDS scores and both VaxiJen and BLAST scores suggest only a marginal relationship between predicted B-cell epitope likelihood, antigenicity, and sequence similarity. While previous epitopes were selected based on conservation identified through bioinformatic analysis by Cabezas-Cruz, the current research demonstrates that these epitopes are less prevalent among the analyzed MSP1a sequences, as identified through the same bioinformatic analysis. Hence, this study highlights the value of using modern prediction tools for epitope mapping and of expanding datasets through sequencing and comprehensive characterization of new strains. The variability observed among the predicted B-cell epitopes of the analyzed MSP1a sequences limits the extent of broad cross-reactivity between strains. So, the MSP1a protein alone poses challenges for the design of cross-protective *in silico* vaccines. An effective and efficient *in silico* vaccine construction requires highly conserved B-cell epitopes across a larger set of *Anaplasma marginale* strains, which MSP1a alone fails to provide in the analyzed sequences due to highly variant B-cell epitopes within the tandem repeats. Other studies also report that the MSP1a protein is highly variable among *A. marginale* strains, due to a greater number and diversity of tandem repeats, leading to lower cross-protection between strains [42]. Other reasons include the fact that MSP1a proteins are subject to immediate alterations in expression to evade immune detection, thereby reducing vaccine effectiveness [43]. In another study, it was found that the MSP1a protein does not consistently elicit strong T-cell responses, which are essential for controlling intracellular pathogens. Experiments conducted on MSP1a-based vaccines yielded limited success in a field trial, resulting in inconsistent protection levels [44]. As new bioinformatic tools and technologies emerged, vaccine production has become easier and faster. In two of our recent research studies published in 2023 and 2025, we proposed two new *in silico* vaccines that contain newly predicted B-cell and T-cell epitopes derived from the conserved regions of the MSP1a protein, along with other outer membrane proteins, such as MSP2, Vir B10, Omp1, and Omp11 [45,46]. During the development of these vaccine constructs, we also observed a few conserved regions within the MSP1a protein across a smaller subset of *Anaplasma marginale* strains; therefore, we included four outer membrane proteins with highly conserved epitopes, along with MSP1a, to construct *in silico* vaccines which will improve the breadth of protection rather than relying solely on MSP1a. In line with recent research proposals, other vaccine strategies should also be considered, such as incorporating moonlighting antigens, like enolase, alongside outer membrane proteins [47].

In addition, the correlation between the number of tandem repeat 2-D structure models and the number of B-cell epitope types at the exact geographic location was R^2^ = 0.46, compared with the previous study, which reported R^2^ = 0.69. Therefore, a weaker association was observed between tandem repeat 2-D structures and B-cell epitope diversity compared to the previous study. 65.8% of tandem repeats, which contained type 1 b-cell epitopes, were found to be α-helices as their secondary structure, 88.3% of the tandem repeats with type 3 b-cell epitopes were found to be α-helices as their secondary structure, and 100% of the type 2 and type 4 b-cell epitopes were also found to be α-helices as their secondary structure. All the tandem repeats containing type 1, type 2, type 3, and type 4 B-cell epitopes could be observed in the S28–S31 Files. Therefore, like Cabezas-Cruz’s study, the results are consistent with the fact that tandem repeats containing type 1, type 2, type 3, and type 4 B-cell epitopes were present in tandem repeats of MSP1a strains, which were declared as tick-transmissible in NCBI records. None of the tandem repeats in our study was found to be non-tick transmissible, as observed in previous research by Cabezas-Cruz, which included the model type B-cell epitope. The secondary structure predictions of all the new tandem repeats also revealed only α-helices and coils, and no beta sheets. These observations are also consistent with the fact that all the tandem repeats we analyzed were derived from tick-transmissible MSP1a strains. In addition, the model B-cell epitope, SSAGGQQQESS, was not detected in any of the tick-transmissible strains analyzed in this study.

The primary limitation of the study is the lack of experimental validation of the predicted B-cell epitopes and immune recognition. However, it should be noted that the analyses carried out in the present study were performed using bioinformatic approaches comparable to those which were used in Cabezas-Cruz and de la Fuente’s (2013) research, allowing direct comparison between the two datasets. Future work should incorporate experimental assays to evaluate the biological importance of the predicted epitope variants and their antigenic properties.

In conclusion, the research has conducted an updated nomenclature classification of the new 157 MSP1a sequences, significantly expanding on the work of Cabezas-Cruz et al. (2013), which provides information on the distribution of *A. marginale* strains across 13 countries. The study identified about 189 unique tandem repeats, reported their tick transmissibility, predicted the secondary structures of all the tandem repeats demonstrating 22 distinct 2D structure models, analyzed amino acid variability of both R1 and non-R1 tandem repeats, detected new serine positions within tandem repeats, as well as documented considerable variability with limited conservation among the predicted B cell epitopes in MSP1a tandem repeats and lastly analyzed the correlation between the number of tandem repeat 2-D structure models and the number of B-cell epitope types found in the tick transmissible MSP1a strains. The study also classifies the unique R1 tandem repeats of the new MSP1a sequences into distinct ecoregion clusters using principal component analysis of NDVI values to understand MSP1a diversity among *Anaplasma marginale* strains at a large scale. In addition, the study conducted phylogenetic correlation analysis of tick-transmissible MSP1a strains with their secondary structures and TCE-binding tandem repeats, which showed that although the phylogenetic tree demonstrated apparent clustering of MSP1a tandem repeats according to the amino acid at position 20 and their predicted secondary structures, permutation-based statistical analyses demonstrated that these associations were not significant. Overall, the research provides updated insights into the diversity, distribution and structural characteristics of MSP1a sequences in *A. marginale,* which may inform future efforts in molecular typing, epidemiological investigations, diagnostics, and vaccine research.

## Supporting information

S1 File The table represents the year of deposition of each of the 157 MSP1a strains in NCBI selected between 2013 and 2023. (DOCX)

S2 File The table represents the MSP1a sequences collected from different regions of different countries, with their tandem repeat sequences and the number of tandem repeats found within each MSP1a strain. (DOCX)

S3 File The table represents the nomenclature classification of the MSP1a strains of *A. marginale.* (DOCX)

S4 File The table represents the global occurrences of common strains and common tandem repeats. (DOCX)

S5 File The table represents the frequency of amino acids per position within the R1 position tandem repeats. (XLSX)

S6 File The table represents the frequency of amino acids per position within the non-R1 position tandem repeats. (XLSX)

S7 File The table represents the secondary structure of each of the tandem repeats. (DOCX)

S8 File The table represents the MSP1a strains with the information on the secondary structures of the tandem repeats found within them and whether each of the strains is tick-transmissible or not. (DOCX)

S9 File The table represents the NDVI values of all countries from 2014 to 2024. (CSV)

S10 File The table represents the land temperature of ecoregion 1 from 2014 to 2024. (CSV)

S11 File The table presents rainfall data for ecoregion 1 from 2014 to 2024. (CSV)

S12 File The table shows land temperatures for ecoregion 2 from 2014 to 2024. (CSV)

S13 File The table presents rainfall data for ecoregion 2 from 2014 to 2024. (CSV)

S14 File The table shows land temperatures for ecoregion 3 from 2014 to 2024. (CSV)

S15 File The table presents rainfall data for ecoregion 3 from 2014 to 2024. (CSV)

S16 File The table represents the land temperature of ecoregion 4 from 2014 to 2024. (CSV)

S17 File The table presents rainfall data for ecoregion 4 from 2014 to 2024. (CSV)

S18 File The table represents the Principal Component Analysis results of all the NDVI values of all the countries. (XLSX)

S19 File The table presents the ka-ks calculations for ecoregion 1 R1 sequences. (XLS)

S20 File The table presents the ka-ks calculations for ecoregion 2 R1 sequences. (XLS)

S21 File The table represents the ka-ka calculation of ecoregion 3 R1 sequences. (XLS)

S22 File The table represents the ka-ka calculation of ecoregion 4 R1 sequences. (XLS)

S23 File The table represents the association of the phylogenetic clusters in the phylogenetic tree with the amino acid 20^th^ position. (DOCX)

S24 File The table represents the association of the secondary structures with the 20th amino acid position. (DOCX)

S25 File The table represents the predicted B-cell epitopes with their BCPRED scores, antigenicity scores and transmembrane location. (DOCX)

S26 File The table shows the specific B-cell epitopes predicted from their tandem repeat sequences, along with the number of occurrences in the stated countries. (DOCX)

S27 File The table represents the Blastp scores of all the B-cell epitopes predicted from their tandem repeats compared to the model sequence. (DOCX)

S28 File The table represents the secondary structures of the tandem repeats, which contain the Type 1 B-cell epitopes. (DOCX)

S29 File The table represents the secondary structures of the tandem repeats, which contain the Type 2 B-cell epitopes. (DOCX)

S30 File The table represents the secondary structures of the tandem repeats, which contain the Type 3 B-cell epitopes. (DOCX)

S31 File The table represents the secondary structures of the tandem repeats, which contain the Type 4 B-cell epitopes. (DOCX)
