# Supplementary material for: Nomenclature, classification, and in-silico structural analysis of MSP1a variants of Anaplasma marginale
Source: PLoS One. 2026 Oct 21;21(10):e0360505. doi: 10.1371/journal.pone.0360505 (PMC13686480; doi:10.1371/journal.pone.0360505)
Supplement: Supplementary file 1 [file pone.0360505.s001.docx]

| S.I. | Accession ID | Strain Name | Year of Publishing |
| --- | --- | --- | --- |
| 1. | OL629255.1 | Brazil/Maranhao/-(A,B,C) | 17-JAN-2022 |
| 2. | OL629249.1 | Brazil/Maranhao/-(A,S,C,V) | 17-JAN-2022 |
| 3. | OL629250.1 | Brazil/Maranhao/-(A,S²,T,U) | 17-JAN-2022 |
| 4. | OL629254.1 | Brazil/Maranhao/-(E,F,G,H) | 17-JAN-2022 |
| 5. | OL629253.1 | Brazil/Maranhao/-(J,I,J²,K) | 17-JAN-2022 |
| 6. | OL629247.1 | Brazil/Maranhao/-(J,α,β,Γ,π,Σ) | 17-JAN-2022 |
| 7. | OL629252.1 | Brazil/Maranhao/-(L,M,N,O) | 17-JAN-2022 |
| 8. | OL629248.1 | Brazil/Maranhao/-(Q,P,Q,W) | 17-JAN-2022 |
| 9. | OL629251.1 | Brazil/Maranhao/-(Q,P,Q²,R) | 17-JAN-2022 |
| 10. | MF771044.1 | Colombia/-(10,11,12) | 25-JUL-2018 |
| 11. | MF771043.1 | Colombia/-(13,14) | 25-JUL-2018 |
| 12. | MF771042.1 | Colombia/-(15,16,17) | 25-JUL-2018 |
| 13. | MF771041.1 | Colombia/-(18,19,20) | 25-JUL-2018 |
| 14. | MF771034.1 | Colombia/-(18,38,39) | 25-JUL-2018 |
| 15. | MF771040.1 | Colombia/-(21^2,R) | 25-JUL-2018 |
| 16. | MF771039.1 | Colombia/-(22,23,24,25) | 25-JUL-2018 |
| 17. | MF771038.1 | Colombia/-(26,27,28) | 25-JUL-2018 |
| 18. | MF771037.1 | Colombia/-(29,30,31) | 25-JUL-2018 |
| 19. | MF771046.1 | Colombia/-(3,4,5,6) | 25-JUL-2018 |
| 20. | MF771036.1 | Colombia/-(32,33,34) | 25-JUL-2018 |
| 21. | MF771035.1 | Colombia/-(35,36,37) | 25-JUL-2018 |
| 22. | MF771033.1 | Colombia/-(40,21,Q,R) | 25-JUL-2018 |
| 23. | MF771032.1 | Colombia/-(41,42,43,44) | 25-JUL-2018 |
| 24. | MF771031.1 | Colombia/-(45,46,38) | 25-JUL-2018 |
| 25. | MF771045.1 | Colombia/-(7,8,9) | 25-JUL-2018 |
| 26. | MF771048.1 | Colombia/-(σ,μ,τ) | 25-JUL-2018 |
| 27. | MF771047.1 | Colombia/-(Φ,1,2) | 25-JUL-2018 |
| 28. | MK809373.1 | Cuba/Western Cuba/-(15^3, 49, 50) | 17-MAY-2021 |
| 29. | MK809370.1 | Cuba/Western Cuba/-(38^2, 47) | 17-MAY-2021 |
| 30. | MK809375.1 | Cuba/Western Cuba/-(48,15,51) | 17-MAY-2021 |
| 31. | MK809378.1 | Cuba/Western Cuba/-(48, 15, 8, 54) | 17-MAY-2021 |
| 32. | MK809374.1 | Cuba/Western Cuba/-(48,15,8) | 17-MAY-2021 |
| 33. | MK809376.1 | Cuba/Western Cuba/-(48, 15^3, 10) | 17-MAY-2021 |
| 34. | MK809372.1 | Cuba/Western Cuba/-(48,15^3) | 17-MAY-2021 |
| 35. | MK809371.1 | Cuba/Western Cuba/-(48^3) | 17-MAY-2021 |
| 36. | MK809377.1 | Cuba/Western Cuba/-(A, S, 52, 53) | 17-MAY-2021 |
| 37. | MF288033.1 | Ecuador/Loja/-( 67^6, 68) | 17-MAY-2021 |
| 38. | MF288036.1 | Ecuador/Loja/-(60^2) | 17-MAY-2021 |
| 39. | MF288032.1 | Ecuador/Loja/-(61, 62, 63, 62, 64) | 17-MAY-2021 |
| 40. | MF288034.1 | Ecuador/Loja/-(65, 66, 60^2) | 17-MAY-2021 |
| 41. | MF288037.1 | Ecuador/Loja/-(Q^3) | 17-MAY-2021 |
| 42. | MF2884.1 | Ecuador/Zamora-Chinchipe/-(55^2,56) | 17-MAY-2021 |
| 43. | MF288043.1 | Ecuador/Zamora-Chinchipe/-(99,141,57) | 17-MAY-2021 |
| 44. | MF288041.1 | Ecuador/Zamora-Chinchipe/-(L, 52, 58) | 17-MAY-2021 |
| 45. | MF288039.1 | Ecuador/Zamora-Chinchipe/-(Q^2, 57) | 17-MAY-2021 |
| 46. | MF288038.1 | Ecuador/Zamora-Chinchipe/-(Q^2, 59^2) | 17-MAY-2021 |
| 47. | MF288040.1 | Ecuador/Zamora-Chinchipe/-(Q^3, 57) | 17-MAY-2021 |
| 48. | MF288035.1 | Ecuador | 17-MAY-2021 |
| 49. | MF288042.1 | Ecuador | 17-MAY-2021 |
| 50. | MN311244.1 | Egypt/-(40^2,73,74) | 13-APR-2020 |
| 51. | MN311243.1 | Egypt/-(40^3,74) | 13-APR-2020 |
| 52. | MN311245.1 | Egypt/-(75^2,76,77 ) | 13-APR-2020 |
| 53. | MN273314.1 | Egypt/-(78,79^2,80) | 13-APR-2020 |
| 54. | MN273313.1 | Egypt/-(81^2,82,83,84) | 13-APR-2020 |
| 55. | MN273312.1 | Egypt/-(85,81,76 ) | 13-APR-2020 |
| 56. | MN370071.1 | Egypt/El-Wady El-Geded/-(10,15,8) | 13-APR-2020 |
| 57. | MN370076.1 | Egypt/El-Wady El-Geded/-(15^3,8) | 13-APR-2020 |
| 58. | MN370072.1 | Egypt/El-Wady El-Geded/-(48,15^3,10) | 13-APR-2020 |
| 59. | MN370077.1 | Egypt/El-Wady El-Geded/-(69,70,71,72) | 13-APR-2020 |
| 60. | MN257057.1 | Egypt/ElMinia/-(84,86,87^3,88 ) | 13-APR-2020 |
| 61. | MN257056.1 | Egypt/ElMinia/-(85,89,90,85) | 13-APR-2020 |
| 62. | MN257055.1 | Egypt/ElMinia/-(91,85,81,76 ) | 13-APR-2020 |
| 63. | MN370075.1 | Egypt | 13-APR-2020 |
| 64. | MN370074.1 | Egypt | 13-APR-2020 |
| 65. | MN370073.1 | Egypt | 13-APR-2020 |
| 66. | MN370072.1 | Egypt | 13-APR-2020 |
| 67. | JN564635.1 | Mexico/Chiapas/-(A, S^2, 52, 53) | 25-JUL-2016 |
| 68. | EF053268.1 | Mexico/Chiapas/-(A^3, 92, 93) | 14-JUL-2016 |
| 69. | JN564642.1 | Mexico/Hidalgo/-(69, 70, 71, 94) | 25-JUL-2016 |
| 70. | AF345870.1 | Mexico/Veracruz/-(A^2, 52, 53) | 26-JUL-2016 |
| 71. | KC181903.1 | Philippines/Batangas/-(104, 103, 753, 107) | 26-FEB-2013 |
| 72. | KC181902.1 | Philippines/Batangas/-(1084, 109) | 26-FEB-2013 |
| 73. | KC181867.1 | Philippines/Batangas/-(111, 112, 113) | 26-FEB-2013 |
| 74. | KC181866.1 | Philippines/Batangas/-(114, S2, 522, 92, 53) | 26-FEB-2013 |
| 75. | KC181868.1 | Philippines/Batangas/-(Q2, R) | 26-FEB-2013 |
| 76. | KC181906.1 | Philippines/Cavite/-(104, 105, 75) | 26-FEB-2013 |
| 77. | KC181904.1 | Philippines/Cavite/-(106, 402) | 26-FEB-2013 |
| 78. | KC181897.1 | Philippines/Cavite/-(Q, R) | 26-FEB-2013 |
| 79. | KC181907.1 | Philippines/Iloilo/-(102, 102) | 26-FEB-2013 |
| 80. | KC181898.1 | Philippines/Iloilo/-(1103, 192) | 26-FEB-2013 |
| 81. | KC181914.1 | Philippines/Iloilo/-(95, 54) | 26-FEB-2013 |
| 82. | KC181915.1 | Philippines/Iloilo/-(Q2, R) | 26-FEB-2013 |
| 83. | KC181908.1 | Philippines/Negros Occidental/-(192) | 26-FEB-2013 |
| 84. | KC181910.1 | Philippines/Negros Occidental/-(98, 97, H) | 26-FEB-2013 |
| 85. | KC181911.1 | Philippines/Negros Occidental/-(99, 100) | 26-FEB-2013 |
| 86. | KC181913.1 | Philippines/Negros Oriental/-(97, H) | 26-FEB-2013 |
| 87. | KC181899.1 | Philippines | 26-FEB-2013 |
| 88. | KC181901.1 | Philippines | 26-FEB-2013 |
| 89. | KC181900.1 | Philippines | 26-FEB-2013 |
| 90. | KC181905.1 | Philippines | 26-FEB-2013 |
| 91. | KC181909.1 | Philippines | 26-FEB-2013 |
| 92. | KC181912.1 | Philippines | 26-FEB-2013 |
| 93. | MG570150 | Russia/Moscow/ - (147^2) | 01-DEC-2018 |
| 94. | MG570151 | Russia/Moscow/ - (148, 147) | 01-DEC-2018 |
| 95. | MG570152 | Russia/Moscow/ - (148, 147^5) | 01-DEC-2018 |
| 96. | MG570154 | Russia/Moscow/ - (149, 150) | 01-DEC-2018 |
| 97. | MG570155 | Russia/Moscow/ - (149, 150^2) | 01-DEC-2018 |
| 98. | MG570156 | Russia/Moscow/ - (149, 150^3) | 01-DEC-2018 |
| 99. | MG570153 | Russia/Moscow/ - (150^3) | 01-DEC-2018 |
| 100. | MG570157 | Russia/Moscow/ - (151,152) | 01-DEC-2018 |
| 101. | MG570159 | Russia/Moscow/ -(152^4) | 01-DEC-2018 |
| 102. | MG570160.1 | Russia/Saratov/-(154^5) | 01-DEC-2018 |
| 103. | MG570158 | Russia | 01-DEC-2018 |
| 104. | MG570149 | Russia | 01-DEC-2018 |
| 105. | MG570164.1 | Russia | 01-DEC-2018 |
| 106. | MG570163.1 | Russia | 01-DEC-2018 |
| 107. | MG570162.1 | Russia | 01-DEC-2018 |
| 108. | MG570161.1 | Russia | 01-DEC-2018 |
| 109. | OQ384906.1 | South Africa/Mpumalanga/-(130, 131, 132, 133, 134) | 26-MAR-2023 |
| 110. | OQ384901.1 | South Africa/Mpumalanga/-(135, 136, 132, 133, 136, 132, 133, 137) | 26-MAR-2023 |
| 111. | OQ384898.1 | South Africa/Mpumalanga/-(135, 136, 132, 133, 137) | 26-MAR-2023 |
| 112. | OQ384904.1 | South Africa/Mpumalanga/-(135, 136, 132, 137) | 26-MAR-2023 |
| 113. | OQ384903.1 | South Africa/Mpumalanga/-(138, 132, 137) | 26-MAR-2023 |
| 114. | OQ384894.1 | South Africa/Mpumalanga/-(139, 140, 141, 142) | 26-MAR-2023 |
| 115. | OQ384890.1 | South Africa/Mpumalanga/-(143, 144, 145, 143, 146) | 26-MAR-2023 |
| 116. | OQ384912.1 | South Africa | 26-MAR-2023 |
| 117. | OQ384909.1 | South Africa | 26-MAR-2023 |
| 118. | OQ384905.1 | South Africa | 26-MAR-2023 |
| 119. | OQ384906.1 | South Africa | 26-MAR-2023 |
| 120. | MT664982.1 | Spain/Southern Spain/-(59^3, 115, 116) | 23-AUG-2021 |
| 121. | ON489300.1 | Thailand/-(155,38^2,157) | 30-JAN-2023 |
| 122. | ON489292.1 | Thailand/-(156^5) | 30-JAN-2023 |
| 123. | ON489290.1 | Thailand/-(157,38) | 30-JAN-2023 |
| 124. | ON489289.1 | Thailand/-(157^2,38) | 30-JAN-2023 |
| 125. | ON489288.1 | Thailand/-(157^3,38) | 30-JAN-2023 |
| 126. | ON489287.1 | Thailand/-(157^3,38^2) | 30-JAN-2023 |
| 127. | ON489291.1 | Thailand/-(157^5,38) | 30-JAN-2023 |
| 128. | ON489301.1 | Thailand/-(38^2, Q, 69) | 30-JAN-2023 |
| 129. | ON489286.1 | Thailand/-(38^3) | 30-JAN-2023 |
| 130. | ON489302.1 | Thailand/-(69^2, 38) | 30-JAN-2023 |
| 131. | ON489299.1 | Thailand/-(69^2) | 30-JAN-2023 |
| 132. | ON489296.1 | Thailand/-(P, 38^2, Q) | 30-JAN-2023 |
| 133. | ON489298.1 | Thailand/-(Q^4, 38) | 30-JAN-2023 |
| 134. | ON489295.1 | Thailand | 30-JAN-2023 |
| 135. | ON489294.1 | Thailand | 30-JAN-2023 |
| 136. | ON489293.1 | Thailand | 30-JAN-2023 |
| 137. | MF787745.1 | Turkey/Elazig/-(120^4, 121, 84) | 29-JUL-2018 |
| 138. | MF787743.1 | Turkey/Elazig/-(122,123,124) | 29-JUL-2018 |
| 139. | MF787744.1 | Turkey/Elazig/-(122^2, 84^2) | 29-JUL-2018 |
| 140. | MF377388.1 | Turkey/Elazig/-(125, 126) | 29-JUL-2018 |
| 141. | MG983514.1 | Turkey/Elazig/-(127,128^2,,129) | 29-JUL-2018 |
| 142. | MF787746.1 | Turkey/Elazig/-(18, 47^2) | 29-JUL-2018 |
| 143. | MF787747.1 | Turkey/Elazig/-(78, 117,118,119) | 29-JUL-2018 |
| 144. | MF377394.1 | Turkey/Elazig/-(84^3,87, 88) | 29-JUL-2018 |
| 145. | MF787742.1 | Turkey/Elazig/-(A, S^5,58 ) | 29-JUL-2018 |
| 146. | MF377395.1 | Turkey | 29-JUL-2018 |
| 147. | MF377394.1 | Turkey | 29-JUL-2018 |
| 148. | MF377393.1 | Turkey | 29-JUL-2018 |
| 149. | MF377392.1 | Turkey | 29-JUL-2018 |
| 150. | MF377391.1 | Turkey | 29-JUL-2018 |
| 151. | MF377390.1 | Turkey | 29-JUL-2018 |
| 152. | MF377389.1 | Turkey | 29-JUL-2018 |
| 153. | MF377388.1 | Turkey | 29-JUL-2018 |
| 154. | MG983517.1 | Turkey | 29-JUL-2018 |
| 155. | MG983516.1 | Turkey | 29-JUL-2018 |
| 156. | MW486117.1 | Grenada | 10-MAR-2021 |
| 157. | MW486118.1 | Grenada | 10-MAR-2021 |
