# Supplementary material for: Nomenclature, classification, and in-silico structural analysis of MSP1a variants of Anaplasma marginale
Source: PLoS One. 2026 Oct 21;21(10):e0360505. doi: 10.1371/journal.pone.0360505 (PMC13686480; doi:10.1371/journal.pone.0360505)
Supplement: Supplementary file 2 [file pone.0360505.s002.docx]

| **Serial No.** | **Accession ID** | **Geographical Location** | **Region / Locality** | **Fasta Sequence** | **Tandem Repeat Nucleotide Sequence** | **Tandem Repeat Protein Sequence** | **No. of Tandem Repeats** |
| --- | --- | --- | --- | --- | --- | --- | --- |
| 1 | OL629255.1 | Brazil | Maranhao | >OL629255.1 Anaplasma marginale isolate Maranhao117 major surface protein 1a (msp1a) gene, partial cds  CTGCCGGTGTGGTAGCGTGCTGGTTGTGTGGTTGTCCTCTTTCCCGATGTTGGGTCGTTCGTTTTACGTC  TCACAAGTTTGTACGCTGTGCCCCTGGCAGTGTAGGGTTTGTTTGTGTGTGTGTGTGTTATGTCAGAGTA  TGTGTCCAGCCAGCCAGCTGATAGCTCGTCAGCGAGTGGTGTGTTATCTCAAAGTGGTCAGGCCAGTACA  TCGTCTCAATTAGGAACTGATAGCTCGTCAGCGGGTGATCAGCAGCAAGGGAGTGGTGTGTCATCTCAAA  GTGGTCAGGCCAGTACATCGTCTCAATTAGGAGCTGATAGCTCGTCAGCGGGTGGTCAGCAGCAAGAGAG  TAGTGTGTTATCTCCAAGTGGTCAGGCCAGTACATCGTCTCAATTAGGAGCTGATAGCTCGTCAGCGAGT  GGTCAGCAACAAGAGAGTGGTGTGTTATCTCAAAGTGGTCAGGCCAGTACA | GAGTGGTGTGTTATCTCAAAGTGGTCAGGCCAGTACATCGTCTCAATTAGGAACTGATAGCTCGTCAGCGGGTGATCAGCAGCAAGG  GAGTGGTGTGTCATCTCAAAGTGGTCAGGCCAGTACATCGTCTCAATTAGGAGCTGATAGCTCGTCAGCGGGTGGTCAGCAGCAAGA  GAGTAGTGTGTTATCTCCAAGTGGTCAGGCCAGTACATCGTCTCAATTAGGAGCTGATAGCTCGTCAGCGAGTGGTCAGCAACAAGA  GAGTGGTGTGTTATCTCAAAGTGGTCAGGCCAGTACA | 1. SGVLSQSGQASTSSQLGTDSSSAGDQQQ  2.  SGVSSQSGQASTSSQLGADSSSAGGQQQ  3.  SSVLSPSGQASTSSQLGADSSSASGQQQ  4.  SGVLSQSGQAST | 3.0 |
| 2 | OL629254.1 | Brazil | Maranhao | >OL629254.1 Anaplasma marginale isolate Maranhao203 major surface protein 1a (msp1a) gene, partial cds  GTTCCGTATGTTACAATCGGGCTGCCGGTGTGGTAGCGTGCTGGTTGTGTGGTTGTCCTCTTTCCCGATG  TTGGGTCGTTCGTTTTACGTCTCACAAGTTTGTACGCTGTGCCCCTGGCAGTGTAGGGTTTGTTTGTGTG  TGTGTGTGTTATGTCAGAGTTGTCCCTTCAGCCAACTGATAGCTCGTCAGCGAGTGGTCAGCAGCAAGAG  AGTAGTGTGTTACCTCAAAGTGATCAGGCCAGTACATCGTCTCAATTAGGAGCTGATAGCTCGCCAGCGA  GTGGTCAGCAGCAAGAGAGTAGTGTGTTACCTCAAAGTGATCAGGCCAGTACATCGTCTCAATTAGGAGC  TGGTAGCTCGTCAGCGAGTGGTCAGCAGCAAGAGAGTAGTGTGTTACCTCAAAGTGGTCAGGCCAGTACA  TCGTCTCAATTAGGAGCTGATAGCTCGTCAGCGAGTGGTCAGCAGCAAGAGAGTAGTGTGTTATCTCAAA  GTGGGGCCAGTACATCGTCTCAATTAGGGACTAATTGGCGGCAAGAGATGCGCTCCAAGGTTGCG | CTGATAGCTCGTCAGCGAGTGGTCAGCAGCAAGAGAGTAGTGTGTTACCTCAAAGTGATCAGGCCAGTACATCGTCTCAATTAGGAG  CTGATAGCTCGCCAGCGAGTGGTCAGCAGCAAGAGAGTAGTGTGTTACCTCAAAGTGATCAGGCCAGTACATCGTCTCAATTAGGAG  CTGGTAGCTCGTCAGCGAGTGGTCAGCAGCAAGAGAGTAGTGTGTTACCTCAAAGTGGTCAGGCCAGTACATCGTCTCAATTAGGAG  CTGATAGCTCGTCAGCGAGTGGTCAGCAGCAAGAGAGTAGTGTGTTATCTCAAAGTG---GGGCCAGTACATCGTCTCAATTAGG | 5.  DSSSASGQQQESSVLPQSDQASTSSQLG  6.  DSSPASGQQQESSVLPQSDQASTSSQLG  7.  GSSSASGQQQESSVLPQSGQASTSSQLG  8.  DSSSASGQQQESSVLSQSGASTSSQL | 4.0 |
| 3 | OL629253.1 | Brazil | Maranhao | >OL629253.1 Anaplasma marginale isolate Maranhao138 major surface protein 1a (msp1a) gene, partial cds  GGGCTGCCGGTGTGGTAGCGTGCTGGTTGTGTGGTTGTCCTCTTTCCCGATGTTGGGTCGTTCGTTTTAC  GTCTCACAAGTTTGTACGCTGTGCCCCTGGCAGTGTAGGGTTTGTTTGTGTGTGTGTTATGTCAGAGTTG  TCCCTTCAGCCAACTGATAGCTCGTCAGCGAGTGGTCAGCAGCAAGAGAGTAGTGTGTTATCTCCAAGTG  GTCAGGCCAGTACATCGTCTCAATTAGGAGCTGATAGCTCGTCAGCGAGTGGTCAGCAGCAAGAGAGTGG  TGTGTTATCTCAAAGTGGTCAGGCCAGTACATCGTCTCAATTAGGAGCTGATAGCTCGTCAGCGAGTGGT  CAGCAGCAAGAGAGTAGTGTGTTATCTCCAAGTGGTCAGGCCAGTACATCGTCTCAATTAGGAGCTGATA  GCTCGTCAGCGAGTGGTCAGCAGCAAGAGAGTAGTGTGTTATCTCCAAGTGGTCAGGCCAGTACATCGTC  TCAATTAGGAGCTGATAGCTCGTCAGCGAGTGGTCAGCAGCAAGAGAGTGGTGTGTTATCTCAAAGTGGT  CAGGCCAGTACATCGTCTCAATTAGGGACTGATTGGCGGCAAGAGATGCGCTCCAAGGT | CTGATAGCTCGTCAGCGAGTGGTCAGCAGCAAGAGAGTAGTGTGTTATCTCCAAGTGGTCAGGCCAGTACATCGTCTCAATTAGGAG  CTGATAGCTCGTCAGCGAGTGGTCAGCAGCAAGAGAGTGGTGTGTTATCTCAAAGTGGTCAGGCCAGTACATCGTCTCAATTAGGAG  CTGATAGCTCGTCAGCGAGTGGTCAGCAGCAAGAGAGTAGTGTGTTATCTCCAAGTGGTCAGGCCAGTACATCGTCTCAATTAGGAG  CTGATAGCTCGTCAGCGAGTGGTCAGCAGCAAGAGAGTAGTGTGTTATCTCCAAGTGGTCAGGCCAGTACATCGTCTCAATTAGGAG  CTGATAGCTCGTCAGCGAGTGGTCAGCAGCAAGAGAGTGGTGTGTTATCTCAAAGTGGTCAGGCCAGTACATCGTCTCAATTAGG | 9.  DSSSASGQQQESSVLSPSGQASTSSQLG  10.  DSSSASGQQQESGVLSQSGQASTSSQLG  11.  DSSSASGQQQESSVLSPSGQASTSSQLG  12.  DSSSASGQQQESSVLSPSGQASTSSQLG  13.  DSSSASGQQQESGVLSQSGQASTSSQL | 5.0 |
| 4 | OL629252.1 | Brazil | Maranhao | >OL629252.1 Anaplasma marginale isolate Maranhao257 major surface protein 1a (msp1a) gene, partial cds  CCGGTGTGGTAGCGTGCTGGTTGTGTGGTTGTCCTCTTTCCCGATGTTGGGTCGTTCGTTTTACGTCTCA  CAAGTTTGTACGCTGTGCCCCTGGCAGTGTAGGGTTTGTTTGTGTGTGTGTGTGTTATGTCAGAGTATGT  GTCCAGCCAGCCAGCTGATAGCTCGTCAGCGAGTGGTGTGTTATCTCAAAGTGGTCAGGCCAGTACATCG  TCTCAATTAGGAACTGATAGCTCGTCAGCGAGTGGTCAGCAGCAAGAGAGTAGTGTGTTACCTCAAAGTG  GTCAGGCCAGTACATCGTCTCAATTAGGAGCTGGTAGCTCGTCAGCGAGTGGTCAGCAGCAAGAGAGTAG  TGTGTTATCTCAAAGTGATCAGGCCAGTACATCGTCTCAATTAGGGACTGATAGCTCGTCAGCGAGTGGT  CAGCAGCAAGAGAGTAGTGTGTTATCTCAAAGTGGGGCCAGTACATCGTCTCAAT | GAGTGGTGTGTTATCTCAAAGTGGTCAGGCCAGTACATCGTCTCAATTAGGAACTGATAGCTCGTCAGCGAGTGGTCAGCAGCAAGA  GAGTAGTGTGTTACCTCAAAGTGGTCAGGCCAGTACATCGTCTCAATTAGGAGCTGGTAGCTCGTCAGCGAGTGGTCAGCAGCAAGA  GAGTAGTGTGTTATCTCAAAGTGATCAGGCCAGTACATCGTCTCAATTAGG-GACTGATAGCTCGTCAGCGAGTGGTCAGCAGCAAGA  GAGTAGTGTGTTATCTCAAAGTGG---GGCCAGTACATCGTCTCAAT | 14.  SGVLSQSGQASTSSQLGTDSSSASGQQQ  15.  SSVLPQSGQASTSSQLGAGSSSASGQQQ  16.  SSVLSQSDQASTSSQLGTDSSSASGQQQ  17.  SSVLSQSGASTSSQ | 4.0 |
| 5 | OL629251.1 | Brazil | Maranhao | >OL629251.1 Anaplasma marginale isolate Maranhao91 major surface protein 1a (msp1a) gene, partial cds  GTTCCGTATGTTACAATCGGGCTGCCGGTGTGGTAGCGTGCTGGTTGTGTGGTTGTCCTCTTTCCCGATG  TTGGGTCGTTCGTTTTACGTCTCACAAGTTTGTACGCTGTGCCCCTGGCAGTGTAGGGTTTGTGTGTGTG  TGTGTGTGTTATGTCAGAGTTGTCCCTTCAGTCAACTGATAGCTCGTCAGCGAGTGGTCAGCAGCAAGAG  AGTAGTGTGTTATCTCAAAGTGATCAGGCCAGTACATCGTCTCAATTAGGAGCTGATAGCTCGTCAGCGA  GTGGTCAGCAGCAAGAGAGTAGTGTGTTACCTCAAAGTGGTCAGGCCAGTACATCGTCTCAATTAGGAGC  TGATAGCTCGTCAGCGAGTGGTCAGCAGCAAGAGAGTAGTGTGTTATCTCAAAGTGATCAGGCCAGTACA  TCGTCTCAATTAGGAGCTGATAGCTCGTCAGCGAGTGGTCAGCAGCAAGAGAGTAGTGTGTTATCTCAAA  GTGATCAGGCCAGTACATCGTCTCAATTAGGAGCTGATAGCTCGTCAGCGAGTGGTCAGCAGCAAGAGAG  TAGTGTGTTATCTCAAAGTGATCAGGCCAGTACATCGTCTCAATTAGGGACTGATTGGCGGCAAGAGATG  CGCTCCCAGGTTGCGAGTGTTGAG | CTGATAGCTCGTCAGCGAGTGGTCAGCAGCAAGAGAGTAGTGTGTTATCTCAAAGTGATCAGGCCAGTACATCGTCTCAATTAGGAG  CTGATAGCTCGTCAGCGAGTGGTCAGCAGCAAGAGAGTAGTGTGTTACCTCAAAGTGGTCAGGCCAGTACATCGTCTCAATTAGGAG  CTGATAGCTCGTCAGCGAGTGGTCAGCAGCAAGAGAGTAGTGTGTTATCTCAAAGTGATCAGGCCAGTACATCGTCTCAATTAGGAG  CTGATAGCTCGTCAGCGAGTGGTCAGCAGCAAGAGAGTAGTGTGTTATCTCAAAGTGATCAGGCCAGTACATCGTCTCAATTAGGAG  CTGATAGCTCGTCAGCGAGTGGTCAGCAGCAAGAGAGTAGTGTGTTATCTCAAAGTGATCAGGCCAGTACATCGTCTCAATTAGG | 18.  DSSSASGQQQESSVLSQSDQASTSSQLG  19.  DSSSASGQQQESSVLPQSGQASTSSQLG  20.  DSSSASGQQQESSVLSQSDQASTSSQLG  21.  DSSSASGQQQESSVLSQSDQASTSSQLG  22.  DSSSASGQQQESSVLSQSDQASTSSQL | 5.0 |
| 6 | OL629250.1 | Brazil | Maranhao | >OL629250.1 Anaplasma marginale isolate Maranhao84 major surface protein 1a (msp1a) gene, partial cds  GTTCCGTATGTTACAATCGGGCTGCCGGTGTGGTAGCGTGCTGGTTGTGTGGTTGTCCTCTTTCCCGATG  TTGGGTCGTTCGTTTTACGTCTCACAAGTTTGTACGCTGTGCCCCTGGCAGTGTAGGGTTTGTTTGTGTG  TGTGTGTGTTATGTCAGAGTATGTGTCCAGCCAGCCAGCTGATAGCTCGTCAGCGAGTGGTGTGTTATCT  CAAAGTGGTCAGGCCAGTACATCGTCTCAATTAGGAACTGATAGCTCGTCAGCGGGTGATCAGCAGCAAG  GGAGTGGTGTGTCATCTCAAAGTGGTCAGGCCAGTACATCGTCTCAATTAGGAACTGATAGCTCGTCAGC  GGGTGATCAGCAGCAAGGGAGTGGTGTGTCATCTCAAAGTGGTCAGGCCAGTACATCGTCTCAATTAGGA  ACTGATAGCTCGTCAGCGGGTGATCAGCAGCAAGGGAGTGGTGTGTCATCTCAAAGTGATCAGGCCAGTA  CATCGTCTCAATTAGGAACTGATAGCTCGTCAGCGAGTGGTCAGCAGCAAGAGAGTAGTGTGTTATCTCA  AAGTGATCAGGCCAGTACATCGTCTCAATCAGGAACTGATTGGCGGCAAGAGATGCGCTCCAAGGTT | GAGTGGTGTGTTATCTCAAAGTGGTCAGGCCAGTACATCGTCTCAATTAGGAACTGATAGCTCGTCAGCGGGTGATCAGCAGCAAGG  GAGTGGTGTGTCATCTCAAAGTGGTCAGGCCAGTACATCGTCTCAATTAGGAACTGATAGCTCGTCAGCGGGTGATCAGCAGCAAGG  GAGTGGTGTGTCATCTCAAAGTGGTCAGGCCAGTACATCGTCTCAATTAGGAACTGATAGCTCGTCAGCGGGTGATCAGCAGCAAGG  GAGTGGTGTGTCATCTCAAAGTGATCAGGCCAGTACATCGTCTCAATTAGGAACTGATAGCTCGTCAGCGAGTGGTCAGCAGCAAGA  GAGTAGTGTGTTATCTCAAAGTGATCAGGCCAGTACATCGTCTCAATCAGGAACTGAT | 23.  SGVLSQSGQASTSSQLGTDSSSAGDQQQ  24.  SGVSSQSGQASTSSQLGTDSSSAGDQQQ  25.  SGVSSQSGQASTSSQLGTDSSSAGDQQQ  26.  SGVSSQSDQASTSSQLGTDSSSASGQQQ  27.  SSVLSQSDQASTSSQSGTD | 5.0 |
| 7 | OL629249.1 | Brazil | Maranhao | >OL629249.1 Anaplasma marginale isolate Maranhao78 major surface protein 1a (msp1a) gene, partial cds  GGGCTGCCGGTGTGGTAGCGTGCTGGTTGTGTGGTTGTCCTCTTTCCCGATGTTGGGTCGTTCGTTTTAC  GTCTCACAAGTTTGTACGCTGTGCCCCTGGCAGTGTAGGGTTTGTTTGTGTGTGTGTGTGTTATGTCAGA  GTATGTGTCCAGCCAGCCAGCTGATAGCTCGTCAGCGAGTGGTGTGTTATCTCAAAGTGGTCAGGCCAGT  ACATCGTCTCAATTAGGAACTGATAGCTCGTCAGCGGGTGATCAGCAGCAAGGGAGTGGTGTGTCATCTC  AAAGTGGTCAGGCCAGTACATCGTCTCAATTAGGAACTGATAGCTCGTCAGCGGGTGATCAGCAGCAAGA  GAGTAGTGTGTTATCTCCAAGTGGTCAGGCCAGTACATCGTCTCAATTAGGAGCTGATAGCTCGTCAGCG  AGTGGTCAGCAGCAAGAGAGTGGTGTGTTATCTCAAAGTGGTCAGGCCAGTACATCGTCTCAATTAGGGA  CTGATTGGCGGCAAGAGATGCGCTCCAAGGTTGCAGGTGTTGAG | GAGTGGTGTGTTATCTCAAAGTGGTCAGGCCAGTACATCGTCTCAATTAGGAACTGATAGCTCGTCAGCGGGTGATCAGCAGCAAGG  GAGTGGTGTGTCATCTCAAAGTGGTCAGGCCAGTACATCGTCTCAATTAGGAACTGATAGCTCGTCAGCGGGTGATCAGCAGCAAGA  GAGTAGTGTGTTATCTCCAAGTGGTCAGGCCAGTACATCGTCTCAATTAGGAGCTGATAGCTCGTCAGCGAGTGGTCAGCAGCAAGA  GAGTGGTGTGTTATCTCAAAGTGGTCAGGCCAGTACATCGTCTCAATTAGGGACTGAT | 28.  SGVLSQSGQASTSSQLGTDSSSAGDQQQ  29.  SGVSSQSGQASTSSQLGTDSSSAGDQQQ  30.  SSVLSPSGQASTSSQLGADSSSASGQQQ  31.  SGVLSQSGQASTSSQLGTD | 4.0 |
| 8 | OL629248.1 | Brazil | Maranhao | >OL629248.1 Anaplasma marginale isolate Maranhao58 major surface protein 1a (msp1a) gene, partial cds  GGGCTGCCGGTGTGGTAGCGTGCTGGTTGTGTGGTTGTCCTCTTTCCCGATGTTGGGTCGTTCGTTTTAC  GTCTCACAAGTTTGTACGCTGTGCCCCTGGCAGTGTAGGGTTTGTTTGTGTGTGTGTGTGTTATGTCAGA  GTCGTCCCTTCAGCCAACTGATAGCTCGTCAGCGAGTGGTCAGCAGCAAGAGAGTAGTGTGTTATCTCAA  AGTGATCAGGCCAGTACATCGTCTCAATTAGGAGCTGATAGCTCGTCAGCGAGTGGTCAGCAGCAAGAGA  GTAGTGTGTTACCTCAAAGTGGTCAGGCCAGTACATCGTCTCAATTAGGAGCTGATAGCTCGTCAGCGAG  TGGTCAGCAGCAAGAGAGTAGTGTGTTATCTCAAAGTGATCAGGCCAGTACATCGTCTCAATTAGGAGCT  GATAGCTCGTCAGCGAGTGGTCAGCAGCAAGAGAGCAGTGTGTCATCTCAAAGTGATCAGGCCAGTACAT  CATCTCAATTAGGGACAGATTGGCGGCAAGAA | CTGATAGCTCGTCAGCGAGTGGTCAGCAGCAAGAGAGTAGTGTGTTATCTCAAAGTGATCAGGCCAGTACATCGTCTCAATTAGGAG  CTGATAGCTCGTCAGCGAGTGGTCAGCAGCAAGAGAGTAGTGTGTTACCTCAAAGTGGTCAGGCCAGTACATCGTCTCAATTAGGAG  CTGATAGCTCGTCAGCGAGTGGTCAGCAGCAAGAGAGTAGTGTGTTATCTCAAAGTGATCAGGCCAGTACATCGTCTCAATTAGGAG  CTGATAGCTCGTCAGCGAGTGGTCAGCAGCAAGAGAGCAGTGTGTCATCTCAAAGTGATCAGGCCAGTACATCATCTCAATTAGG | 32.  DSSSASGQQQESSVLSQSDQASTSSQLG  33.  DSSSASGQQQESSVLPQSGQASTSSQLG  34.  DSSSASGQQQESSVLSQSDQASTSSQLG  35.  DSSSASGQQQESSVSSQSDQASTSSQL | 4.0 |
| 9 | OL629247.1 | Brazil | Maranhao | >OL629247.1 Anaplasma marginale isolate Maranhao56 major surface protein 1a (msp1a) gene, partial cds  TTTCCCGATGTTGGGTCGTTCGTTTTCCTTCTCACAAGTTTGTACGCTGTGCCCCTGGCAGTGTAGGGTT  TGTTTGTGTGTGTGTGTGTTATGTCAGAGTGTGTGTCCCTTCAGCCAACTGATAGCTCGTCAGCGAGTGG  TCAGCAACAAGAGAGTAGTGTGTTATCTCCAAGTGGTCAGGCCAGTACATCGTCTCAATTAGGAGCTGAT  AACTCGTCAGCGAGTGGTCAGCAACCAGAGAGTAGTGTGTTATCTCCAAGTGGTCAGGCCAGTACATCGT  CTCAATTAGGAGCTGATAGCTCGTCAGCGAGTGGTCAGCAACCAGAGAGTAGTGTGTTATCTCCAAGTGC  TCAGGCCAGTACATCGTCTCAGTTAGGGACTGATAACTCGTCAGCGAGTGGTCAGCAACAAGAGAGCAGT  GTGTCATCTCAAAGTGCTCAGGCCAGTACATCGTCTCAATTAGGAACTGGTTGGCGGCAAGAGATGCGCT  CCAAGGTTGCGAGTGATGAGAGTACATCGTCTCAATTAGGAGCTGATTTGCGGCAAGAGGTGCACTCCAA  GGTTGCGAGTGTTGAG | 1.  CTGATAGCTCGTCAGCGAGTGGTCAGCAACAAGAGAGTAGTGTGTTATCTCCAAGTGGTCAGGCCAGTACATCGTCTCAATTAGGAG  CTGATAACTCGTCAGCGAGTGGTCAGCAACCAGAGAGTAGTGTGTTATCTCCAAGTACATCGTCTCAATTAGGAGAGTGGTCAGGCC  CTGATAGCTCGTCAGCGAGTGGTCAGCAACCAGAGAGTAGTGTGTTATCTCCAAGTGCTCAGGCCAGTACATCGTCTCAGTTAGG-G  ACTGATAACTCGTCAGCGAGTGGTCAGCAACAAGAGAGCAGTGTGTCATCTCAAAGTGCTCAGGCCAGTACATCGTCTCAATTAGGA | 36.  DSSSASGQQQESSVLSPSGQASTSSQLG  37.  DNSSASGQQPESSVLSPSTSSQLGEWSG  38.  DSSSASGQQPESSVLSPSAQASTSSQLG  39.  TDNSSASGQQQESSVSSQSAQASTSSQLG  40.  STSSQLGTGWRQEMRSKVASDE  41.  STSSQLGADLRQEVHSKVASVE | 4.0  2.0 |
| 10 | MF771048.1 | Colombia |  | >MF771048.1 Anaplasma marginale isolate 1325 major surface protein 1a (msp1a) gene, partial cds  CCATATACTGTGCAGTACGGTTGTGCTCCCCAATTGTTAAAATTTAGTATATTAATCTTGCGATTACACG  TTCCGTATGTTACAATCGGGCTGCCGGTGTGGTAGCGTGCTGGTTGTGTGGTTGTCCTCTTTCCCGATGT  TGGGTCGTTCGTTTTACGTCTCACAAGTTTGTACGCTGTGCCCCTGGCAGTGTAGGGTTTGTTTGTGTGT  GTGTGTGTTATGTCAGAGTATGTGTCCTCCTCCCCAGACGATAGCTCGTCAGCGAGTGATCAGCAGCAAG  AGAGTAGTGTGTCATCTCAAAGTGGTCAGGCCAGTACATCGTCTCAATTAGGAGCTGATAGCTCGTCAGC  GAGTGGTCAGCAACAAGAGAGTAGTGTGTCATCTCAAAGTGATGAGGTCAGTACATCGTCTCAATTAGGA  ATCATAACTCGTCAGAGTGGTGGTCAGCAGCAAGAGAGTAGTGTGTCATCTCAAAGTGGTCAGGCCAGTA  CATCGTCTCAATCAGGAGCTAATTGGCGGCAAGAGATGCGCTCCAAGGTTGCGAGTGTTGAGTACATTTT  GGCTGCTCGTGCCCTTATTTCTGTAGGGGTCTATGCTGCTCAGGGAGAGATCGCGAAATCGCAAGGGTGT  GCTCCCCTGCGTGTTGCAGAAGTCGAAGAAATCGTGAGGGATAGCCTTGTACGCAGCCACTTTCATGATA  GTGGCCTTTCACTAGGCTCCATACGACTCGTGCTTATGCAGGTTGGGGATAAGTTGGGGCTACAAGGTTT  GAAGATTGGCGAAGGGTACGCCACCTATCTCGCGCAAGCGTTTGCTGACAACGTGGTGGTTGCGGCTGAT  GTTCAAAGTGGTGGTGCGTGCTCTGCCAGCCTTGACAGCGCGATCGCAAACGTTGAGACGTCGTGGTCCC  TGCACGGCGGCCTGGTAAGCAAAGATTTTGACCGTGATACCAAAGTAGAAAGGGGCGACCTTGAGGCTTT  TGTCGACTTCATGTTTGGCGGTGTGTCGTACAATGATGGGAACGCGTCTGCGGCTAGGAGCGTATTGGAA  ACGCTTGCCGGGCACGTCGATGCACTTGGTATATCGTACAATCAGCTGGATAAGCTTGATGCTGACACTT  TGTATAGTGTCGTATCGTTTAGTGCCGGTTCCGCAATAGACAGAGGTGCGGTTAGCGATGCGGCTGACAA  GTTCCGTGTGATGATGTTTGGTGGTGCTCCTGCGGGGCAAGAGAAAACTGCCGAACCTGAGCATGAGGCT  GCGACCCCGTCAGCTAGTAGCGTTCCGTCAACTGTGCATGGTAAGGTCGTTGATGCAGTTGACCGTGCAA  AAGAAGCGGCTAAGCAGGCCTATGCAGGCGTGCGTAAGTGGTATGTGGCGAAGCCTTCGGACACTACTAC  ACAGCTTGTTGTAGCTATCACGGCGCTGCTTATCACGGCGTTTGCTATCTGTGCGTGTTTGGAACCTAGG  CTTATAGGGGCGTCCGGTCCGCTGATTTGGGGCTGCCTGGCACTAGTAGCACTGCTGCCATTACTTGGTA  TGGCTGTGCATACGGCAGTGAGTGCTTCGAGTCAAAAGAAGGCTGCCGGTGGTGCGCAACGGGTTGCTGC  TCAGGAGAGGTCTAGGGAATTGTCCCGTGCGAGACAGGAAGATCAGCAGAAGTTGCATGTTCCCGCGATA  CTGACCGGGTTGAGCGTGCTTGTGTTTATTGCTGCCGTCGTGGCTTGTATTGCTGTTGACGCGAAGCGCG  GGACGTGGCAGGGCAGCATATGTTTCCTAGCCGCATTTGTGTTGTTTGCGATCAGTGCCGCTGTTGTAAT  GGCAACACGTGACCAATCGTTGGCAGAAGAGTGTGATAGCAAGTGTGCTACAGCTCGTACGGCTCAAGCT  GTACCCGGTGGCCAGCAGCAGCCGCGTGCTACCGAGGGCGTTGTTAGCGGTGGCAGCCAAGAAGGCGGGG  CTGGTGTTCCCGGAACTTCCGTGCCGTCAGCCGGGTCTGGGTCCGTACCTCCTGCTACCATTATGGTCAG  TGTGGATCCACAACTTGTTGCTACTTTGGGAGCAGGTGTGGCGCAGGCGGCGGCG | GATAGCTCGTCAGCGAGTGATCAGCAGCAAGAGAGTAGTGTGTCATCTCAAAGTGGTCAGGCCAGTACATCGTCTCAATTAGGAGCT  GATAGCTCGTCAGCGAGTGGTCAGCAACAAGAGAGTAGTGTGTCATCTCAAAGTGATGAGGTCAGTACATCGTCTCAATTAGGA-AT  CATAACTCGTCAGAGTGGTGGTCAGCAGCAAGAGAGTAGTGTGTCATCTCAAAGTGGTCAGGCCAGTACATCGTCTCAATCAGGAGCT | 42. DSSSASDQQQESSVSSQSGQASTSSQLGA  43. DSSSASGQQQESSVSSQSDEVSTSSQLG  44. ITRQSGGQQQESSVSSQSGQASTSSQSGA | 3 |
| 11 | MF771047.1 | Colombia |  | >MF771047.1 Anaplasma marginale isolate 1278 major surface protein 1a (msp1a) gene, partial cds  CCATATACTGTGCAGTACGGTTGTGCTCCCCAATTGTTAAAATTTAGTATATTAATCTTGCGATTACACG  TTCCGTATGTTACAATCGGGCTGCCGGTGTGGTAGCGTGCTGGTTGTGTGGTTGTCCTCTTTCCCGATGT  TGGGTCGTTCGTTTTACGTCTCACAAGTTTGTACGCTGTGCCCCTGGCAGTGTAGGGTTTGTTTGTGTGT  GTGTGTGTTATGTCAGTCATTTCATCCCCCCAGTCACTCGATTCAGCGAGTGGTCAGCAGCAAGAGAGTA  GTGTGTCATCTCAAAGTGATCAGGCCAGTAGTACATCGTCTCAATTAGGAGCTGATAGCTCGTCAGCGAG  TGGTCAGCAGCAAGAGAGTAGTGTGTTATCTCAAAGTGATCAGGCCAGTACATCGTCTCACTTAGGATCT  GATAGCTCGTCAGAGAGTGGTCAGCAGCAAGAGAGTAGTGTGTCATCTCAAAGTGATCAGGCCAGTACAT  CGTCTCAATCAGGAGCTGATTGGCGGCAAGAGATGCGCTCCAAGGTTGCGAGTGTTGAGTACATGTTGGC  TGCTCGTGCCCTTATTTCTGTAGGGGTCTATGCTGCTCAGGGAGAGATCGCGAGATCGCAAGGGTGTGCT  CCCCTGCGTGTTGCAGAAGTCGAAGAAATCGTGAGGGATAGCCTTGTACGCAGCCACTTTCATGATAGTG  GCCTTTCACTAGGCTCCATACGACTCGTGCTTATGCAGGTTGGGGATAAGTTGGGGCTACAAGGTTTGAA  GATTGGCGAAGGGTACGCCACCTATCTCGCGCAAGCGTTTGCTGACAACGTGGTGGTTGCGGCTGATGTT  CAAAGTGGTGGTGCGTGCTCTGCCAGCCTTGACAGCGCGATCGCAAACGTTGAGACGTCGTGGTCCCTGC  ACGGCGGCCTGGTAAGCAAAGATTTTGACCGTGGTACCAAAGTAGGAAGGGACGACCTTGAGGCTTTTAT  CGACTTCATGTTTGGCTATGAGTACTACAATGATGTGAACGCGGCTGTGGTTAGGAGAGTATTGGAAACG  CTTGCCGGGCACGTTGATGCACTTGGTATATCGTACAATCAGCTGGATAAGCTTGATGCTGACACTTTGT  ATAGTGTCGTATCGTTTAGTGGCGCTTCCGCAATAGACAGAGGTGCGGTTAGCGATGCGGCTGACAAGTT  CCGTGTGATGATGTTTGGTGGTGCTCATGAGAGGCAAGAGAAAACTGCCGAACCTGAGCATGAGGCTGCG  ACCCCGTCAGCTAGTAGCGTTCCGTCAACTGTACATGGTAAGGCAGTTGATCGTGCAAAAGAAGCGGCTA  AGCAGGCCTATGCAGGCGTGCGTAAGTGGCATGTGGCGAAGCATTCGGACACTACTACACAGCTTGTTGT  AGCTATCACGGCGCTGCTTATCACGGCGTTTGCTATCTGTGCGTGTTTGGAACCTAGGCTTATAGGGGCG  TCCGGTCCGCTGATTTGGGGCTGCCTGGCACTAGTAGCACTGCTGCCATTACTTGGTATGGCTGTGCATA  CGGCAGTGAGTGCTTCGAGTCAAAAGAAGGCTGCCGGTGGTGCGCAACGGGTTGCTGCTCAGGAGAGGTC  TAGGGAATTGTCCCGTGCGAGACAGGAAGATCAGCAGAAGTTGCATGTTCCCGCGATACTGACCGGGTTG  AGCGTGCTTGTGTTTATTGCTGCCGTCGTGGCTTGTATTGCTGTTGACGCGAAGCGCGGGACGTGGCAGG  GCAGCATATGTTTCCTAGCCGCATTTGTGTTGTTTGCGATCAGTGCCGCTGTTGTAATGGCAACACGTGA  CCAATCGTTGGCAGAAGAGTGTGATAGCAAGTGTGCTACAGCTCGTACGGCTCAAGCTGTACCCGGTGGC  CAGCAGCAGCCGCGTGCTACCGAGGGCGTTGTTAGCGGTGGCAGCCAAGAAGGCGGGGCTGGTGTTCCCG  GAACTTCCGTGCCGTCAGCCGGGTCTGGGTCCGTACCTCCTGCTACCATTATGGTCAGTGTGGATCCACA  ACTTGTTGCTACTTTGGGAGCAGGTGTGGCGCAGGCGGCGGCG | TCAGCGAGTGGTCAGCAGCAAGAGAGTAGTGTGTCATCTCAAAGTGATCAGGCCAGTAGTACATCGTCTCAATTAGGAGCTGATAGCTCG    TCAGCGAGTGGTCAGCAGCAAGAGAGTAGTGTGTTATCTCAAAGTGATCAGGCCAGTACATCGTCTCACTTAGGATCTGATAGCTCG    TCAGAGAGTGGTCAGCAGCAAGAGAGTAGTGTGTCATCTCAAAGTGATCAGGCCAGTACATCGTCTCAATCAGGAGCTGAT | 45.  SASGQQQESSVSSQSDQASSTSSQLGADSS  46.  SASGQQQESSVLSQSDQASTSSHLGSDSS  47.  SESGQQQESSVSSQSDQASTSSQSGAD | 3 |
| 12 | MF771046.1 | Colombia |  | >MF771046.1 Anaplasma marginale isolate 1277 major surface protein 1a (msp1a) gene, partial cds  CCATATACTGTGCAGTACGGTTGTGCTCCCCAATTGTTAAAATTTAGTATATTAATCTTGCGATTACACG  TTCCGTATGTTACAATCGGGCTGCCGGTGTGGTAGCGTGCTGGTTGTGTGGTTGTCCTCTTTCCCGATGT  TGGGTCGTTCGTTTTACGTCTCACAAGTTTGTACGCTGTGCCCCTGGCAGTGTAGGGTTTGTTTGTGTGT  GTGTGTGTTATGTCAGAGTATGTGTCCCTTCAGCCAACTGACAGAAGCTCGTCAGCGAGTGGTCAGCAGC  AAAGTGAGAGTAGTGTGTTATCTCAAAGTGGTCAGGCCAGTACATCGTCTCAATTAGGAGCTGATAGCTC  GTCAGCGAGTGGTCAGCAGCAAGAGAGTAGTGTGTTATCTCAAAGTGATCAGGCCAGTACATCGTCTCAA  TTAGGAGCTGATAGCTCGTCAGCGAGTGGTCAGCAGCAAGAGAGTGGTGTGTTATCTCAAAGTGATCAGG  CCAGTACATCGTCTCAATTAGGAGCTGATAGCTCGTCAGCGAGTGGTCAGCAGCAAGAGAGTAGTGTGTT  ATCTCAAAGTGATCAGGCCAGTACATCGTCTCAATTAGGGACTGATTGGCGGCGAGAGATGCGCTCCAAG  GTTGCGAGTGTTGAGTACATTTCGGCTGCTCGTGCCCTTATCTCTGTAGGGGTCTATGCTGCTCAGGAAG  AGATCGCGAGATCGCTAGGGCATACTCCCCTGCGTGTTGCAGAAGTCGAAGCAATCGTGAGGGATAGCCT  TGTACGCAGCCACTTTCATGATAGTGGCCTTTCACTAGGCTCCATACGACTCGTGCTTATGCAGGTTGGG  GATAAGTTGGGGCTACAAGGTTTGAAGATTGGCGAAGGGTACGCCACCTATCTCGCGCAAGCGTTTGCTG  ACAGCGTGGTGGTTGCGGCTGATGTTCAGAGTGGTGGTGCGCGCTCTGCTACCAGCCTTGACAAGGCGAT  CGCAGACGTTGAGACGTCGTGGTCCCTGCGCGGCGACCTGATAAGCAAAGATTTTGACCGTGATACCAAA  GTAGGAAGGGACGACCTTGAGGCTTTTGTCGACTTCATGTTTGGCGGTGTGTCGTACAATGATGCGAACG  CGGCTGTGGCTAGGAGCGTATTGGAAACGCTTGCCGGGCACGTCGATGCACTTGGTATATCGTACAATCA  GCTGGATAAGCTTGATGCTGACACTTTGTATAGCGTCGTATCGTTTAGTGGCGCTTCCGCAATAGACAGA  GGTGCGGTTAGCGATGCGGCTGACAAGTTCCGTGTGATGATGTTTGGTGGTGCTCCTGCGGGGCAAGAGA  AAACTCCCGAACCTGAGCATGAGGCTGCGACCCCGTCAGCTAGTAGCGTTCCGTCAACTGTGCATGGTAA  GGTCCTTGATCATGCAGTTGACCGTGCAAAAGAAGCGGCTAAGCAGGCCTATGCAGGCGTGCGTAAGTGG  TATGTGGCGAAGCCTTCGGACACTACTACACAGCTTGTTGTAGCTATCACGGCGCTGCTTATCACGGCGT  TTGCTATCTGTGCGTGTTTGGAACCTAGGCTTATAGGGGCGTCCGGTCCGCTGATTTGGGGCTGCCTGGC  ACTAGTAGCACTGCTGCCATTACTTGGTATGGCTGTGCATACGGCAGTGAGTGCTTCGAGTCAAAAGAAG  GCTGCCGGTGGTGCGCAACGGGTTGCTGCTCAGGAGAGGTCTAGGGAATTGTCCCGTGCGAGACAGGAAG  ATCAGCAGAAGTTGCATGTTCCCGCGATACTGACCGGGTTGAGCGTGCTTGTGTTTATTGCTGCCGTCGT  GGCTTGTATTGCTGTTGACGCGAAGCGCGGGACGTGGCAGGGCAGCATATGTTTCCTAGCCGCATTTGTG  TTGTTTGCGATCAGTGCCGCTGTTGTAATGGCAACACGTGACCAATCGTTGGCAGAAGAGTGTGATAGCA  AGTGTGCTACAGCTCGTACGGCTCAAGCTGTACCCGGTGGCCAGCAGCAGCCGCGTGCTACCGAGGGCGT  TGTTAGCGGTGGCAGCCAAGAAGGCGGGGCTGGTGTTCCCGGAACTTCCGTGCCGTCAGCCGGGTCTGGG  TCCGTACCTCCTGCTACCATTATGGTCAGTGTGGATCCACAACTTGTTGCTACTTTGGGAGCAGGTGTGG  CGCAGGCGGCGGCG | AGCTCGTCAGCGAGTGGTCAGCAGCAAAGTGAGAGTAGTGTGTTATCTCAAAGTGGTCAGGCCAGTACATCGTCTCAATTAGGAGCTGAT  AGCTCGTCAGCGAGTGGTCAGCAGCAAGAGAGTAGTGTGTTATCTCAAAGTGATCAGGCCAGTACATCGTCTCAATTAGGAGCTGAT  AGCTCGTCAGCGAGTGGTCAGCAGCAAGAGAGTGGTGTGTTATCTCAAAGTGATCAGGCCAGTACATCGTCTCAATTAGGAGCTGAT  AGCTCGTCAGCGAGTGGTCAGCAGCAAGAGAGTAGTGTGTTATCTCAAAGTGATCAGGCCAGTACATCGTCTCAATTAGGGACTGATTGG | 48.  SSSASGQQQSESSVLSQSGQASTSSQLGAD  49.  SSSASGQQQESSVLSQSDQASTSSQLGAD  50.  SSSASGQQQESGVLSQSDQASTSSQLGAD  51.  SSSASGQQQESSVLSQSDQASTSSQLGTDW | 4 |
| 13 | MF771045.1 | Colombia |  | >MF771045.1 Anaplasma marginale isolate 1230 major surface protein 1a (msp1a) gene, partial cds  CCATATACTGTGCAGTACGGTTGTGCTCCCCAATTGTTAAAATTTACTATATTAATCTTGCGATTACACG  TTCCGTATGTTACAATCGGGCTGCCGGTGTGGTAGCGTGCTGGTTGTGTGGTTGTCCTCTTTCCCGATGT  TGGGTCGTTCGTTTTACGTCTCACAAGTTTGTACGCTGTGCCCCTGGCAGTGTAGGGTTTGTTTGTGTGT  GTGTGTGTTATGTCAGAGTATGTGTCCCCCCAGCTAGCTGATATCTCGTCAGCGAGTGGTCAGCAGCAAG  AGAGTAGTGTGTCATCTCAAAGTGATCAGGCCAGTACATCGTCTCAATTAGGAGCTGATAGCTCGTCAGC  GGGTGGTCAGCAGCAAGAGAGTAGTGTGTCATCTCAAAGTGGTCAGGCCAGTACATCGTCTCAATTAGGG  ACTGATAGCTCGTCAGCGAGTGGTCAGCAGCAAGAGAGTAGTGTGTCATCTCAAAGTGGTCAGGCCAGTA  CATCGTCTCAATCAGGAGCTAATTGGCGGCAAGAGATGCGCTCCAAGGTTGCGAGTGTTGAGTACATTTT  GGCTGCTCGTGCCCTTATTTCTGTAGGGGTCTATGCTGCTCAGGGAGAGATCGCGAAATCGCAAGGGTGT  GCTCCCCTGCGTGTTGCAGAAGTCGAAGAAATCGTGAGGGATGGCCTTGTACGCAGCCACTTTCATGATA  GTGGCCTTTCACTAGGCTCCATACGACTCGTGCTTATGCAGGTTGGGGATAAGTTGGGGCTACAAGGTTT  GAAGATTGGCGAAGGGTACGCCACCTATCTCGCGCAAGCGTTTGCTGACAACGTGGTGGTTGCGGCTGAT  GTTCAAAGTGGTGGTGCGTGCTCTGCCAGCCTTGACAGCGCGATCGCAAACGTTGAGACGTCGTGGTCCC  TGCACGGCGGCCTGGTAAGCAAAGATTTTGACCGTGATACCAAAGTAGAAAGGGGCGACCTTGAGGCTTT  TGTCGACTTCATGTTTGGCGGTGTGTCGTACAATGATGGGAACGCGTCTGCGGCTAGGAGAGTATTGGAA  ATGCTTGCCGGGCACGTCGATGCACTTGGTATATCGTACAATCAGCAGGATAAGCTTGATGATGACACTT  TGTATAGTGTAGTATCGTTTAGTGCCGGTTCCACAATAGACAGAGGTGCGGTTAGCGATGCGGCTGACAA  GTTCCGTGTGATGATGTTTGGTGGTGCTCCTGCGGGGCAAGAGAAAACTGCCGAACCTGAGCATGAGGCT  GCGACCCCGTCAGCTAGTAGCGTTCCGTCAACTGTGCATGGTAAGGTCGTTGATGCAGTTGACCGTGCAA  AAGAAGCGGCTAAGCAGGCCTATGCAGGCGTGCGTAAGTGGTATGTGGCGAAGCCTTCGGACACTACTAC  ACAGCTTGTTGTAGCTATCACGGCGCTGCTTATCACGGCGTTTGCTATCTGTGCGTGTTTGGAACCTAGG  CTTATAGGGGCGTCCGGTCCGCTGATTTGGGGCTGCCTGGCACTAGTAGCACTGCTGCCATTACTTGGTA  TGGCTGTGCATACGGCAGTGAGTGCTTCGAGTCAAAAGAAGGCTGCCGGTGGTGCGCAACGGGTTGCTGC  TCAGGAGAGGTCTAGGGAATTGTCCCGTGCGAGACAGGAAGATCAGCAGAAGTTGCATGTTCCCGCGATA  CTGACCGGGTTGAGCGTGCTTGTGTTTATTGCTGCCGTCGTGGCTTGTATTGCTGTTGACGCGAAGCGCG  GGACGTGGCAGGGCAGCATATGTTTCCTAGCCGCATTTGTGTTGTTTGCGATCAGTGCCGCTGTTGTAAT  GGCAACACGTGACCAATCGTTGGCAGAAGAGTGTGATAGCAAGTGTGCTACAGCTCGTACGGCTCAAGCT  GTACCCGGTGGCCAGCAGCAGCCGCGTGCTACCGAGGGCGTTGTTAGCGGTGGCAGCCAAGAAGGCGGGG  CTGGTGTTCCCGGAACTTCCGTGCCGTCAGCCGGGTCTGGGTCCGTACCTCCTGCTACCATTATGGTCAG  TGTGGATCCACAACTTGTTGCTACTTTGGGAGCAGGTGTGGCGCAGGCGGCGGCG | AGCTGATATCTCGTCAGCGAGTGGTCAGCAGCAAGAGAGTAGTGTGTCATCTCAAAGTGATCAGGCCAGTACATCGTCTCAATTAGG  AGCTGATAGCTCGTCAGCGGGTGGTCAGCAGCAAGAGAGTAGTGTGTCATCTCAAAGTGGTCAGGCCAGTACATCGTCTCAATTAGG    -GACTGATAGCTCGTCAGCGAGTGGTCAGCAGCAAGAGAGTAGTGTGTCATCTCAAAGTGGTCAGGCCAGTACATCGTCTCAATCAGG | 52.  ADISSASGQQQESSVSSQSDQASTSSQL  53.  ADSSSAGGQQQESSVSSQSGQASTSSQL  54.  TDSSSASGQQQESSVSSQSGQASTSSQS | 3 |
| 14 | MF771044.1 | Colombia |  | >MF771044.1 Anaplasma marginale isolate 1201 major surface protein 1a (msp1a) gene, partial cds  CCATATACTGTGCAGTATGGTTGTGCTCCCCAATTGTTAAAATTTAGTATATTAATCTTGCGATTACACG  TTCCGTATGTTACAATCAGGCCGCCGGTGTGGTAGCGTGCTGGTTGTGTGGTTGTCCTCTTTCCCGATGT  TGGGTCGTTCGTTTTACGTCTCACAAGTTTGTACGCTGTGCCCCTGGCAGTGTAGGGTTTGTTTGTGTGT  GTGTTATGTCAGAGTATGTGTCCAGCCAGTCAGCTGATAGCTCGTCAGCGAGTGGTCAGCAGCAAGAGAG  TAGTGTGTCATCTCAAAGTGGTCAGGCCAGTACATCGTCTCAATTAGGAGCTGATAGCTCGTCAGCGAGT  GGTCAGCAGCAAGAGAGTAGTGTGTCATCTCAAAGTGATCAGGCCAGTACATCGTCTCAATTAGGGACTG  ATACCTCGTCAGCTAGTGGTCAACAGCAAGAGAGTAGTGTGTCATCTCAAAGTGGTCAGGCCAGTACATC  GTCTCAATCAGGAGCTAATTGGCGGCAAGAGATGCGCTCCAAGGTTGCGAGTGTTGAGTACATTTTGGCT  GCTCGTGCCCTTATTTCTGTAGGGGTCTATGCTGCTCAGGGAGAGATCGCGAGATCGCGAGGGTGTGCTC  CCCTGCGTGTTGCAGAAGTCGAAGAAATCGTGAGGGATGGCCTTGTACGCAGCCACTTTCATGATAGTGG  CCTTTCACTAGGCTCCATACGACTCGTGCTTATGCAGGTTGGGGATAAGTTGGGGCTACAAGGTTTGAAG  ATTGGCGAAGGGTACGCCACCTATCTCGCGCAAGCGTTTGCTGACAGCGTGGTGGTTGCGGCTGATGTTC  AAAGTGGTGGTGCGTGCTCTGCTGCCAGCCTTGACAGCGCGATCGCAAACGTTGAGACGTCGTGGTCCCT  GCACGGCGGCCTGGTAAGCAAAGATTTTGACCGTGATACCAAAGTAGAAAGGGGCGACCTTGAGGCTTTT  GTCGACTTCATGTTTGGCGGTGTGTCGTACAATGATGGGAACGCGTCTGCGGCTAGGAGCGTATTGGAAA  CGCTTGCCGGGCACGTCGATGCACTTGGTATATCGTACAATCAGCTGGATAAGCTTGATGCTGACACTTT  GTATAGTGTCGTATCGTTTAGTGCCGGTTCCGCAATAGACAGAGGTGCGGTTAGCGATGCGGCTGACAAG  TTCCGTGTGATGATGTTTGGTGGTGCTCCTGCGGGGCAAGAGAAAACTGCCGAACCTGAGCATGAGGCTG  CGACCCCGTCAGCTAGTAGCGTTCCGTCAACTGTGCATGGTAAGGTCGTTGATGCAGTTGACCGTGCAAA  AGAAGCGGCTAAGCAGGCCTATGCAGGCGTGCGTAAGCGGTATGTGGCGAAGCCTTCGGACACTACTACA  CAGCTTGTTGTAGCTATCACGGCGCTGCTTATCACGGCGTTTGCTATCTGTGCGTGTTTGGAACCTAGGC  TTATAGGGGCGTCCGGTCCGCTGATTTGGGGCTGCCTGGCACTAGTAGCACTGCTGCCATTACTTGGTAT  GGCTGTGCATACGGCAGTGAGTGCTTCGAGTCAAAAGAAGGCTGCCGGTGGTGCGCAACGGGTTGCTGCT  CAGGAGAGGTCTAGGGAATTGTCCCGTGCGAGACAGGAAGATCAGCAGAAGTTGCATGTTCCCGCGATAC  TGACCGGGTTGAGCGTGCTTGTGTTTATTGCTGCCGTCGTGGCTTGTATTGCTGTTGACGCGAAGCGCGG  GACGTGGCAGGGCAGCATATGTTTCCTAGCCGCATTTGTGTTGTTTGCGATCAGTGCCGCTGTTGTAATG  GCAACACGTGACCAATCGTTGGCAGAAGAGTGTGATAGCAAGTGTGCTACAGCTCGTACGGCTCAAGCTG  TACCCGGTGGCCAGCAGCAGCCGCGTGCTACCGAGGGCGTTGTTAGCGGTGGCAGCCAAGAAGGCGGGGC  TGGTGTTCCCGGAACTTCCGTGCCGTCAGCCGGGTCTGGGTCCGTACCTCCTGCTACCATTATGGTCAGT  GTGGATCCACAACTTGTTGCTACTTTGGGAGCAGGTGTGGCGCAGGCGGCGGCG | AGCTGATAGCTCGTCAGCGAGTGGTCAGCAGCAAGAGAGTAGTGTGTCATCTCAAAGTGGTCAGGCCAGTACATCGTCTCAATTAGG  AGCTGATAGCTCGTCAGCGAGTGGTCAGCAGCAAGAGAGTAGTGTGTCATCTCAAAGTGATCAGGCCAGTACATCGTCTCAATTAGG  -GACTGATACCTCGTCAGCTAGTGGTCAACAGCAAGAGAGTAGTGTGTCATCTCAAAGTGGTCAGGCCAGTACATCGTCTCAATCAGG | 55.  ADSSSASGQQQESSVSSQSGQASTSSQL  56.  ADSSSASGQQQESSVSSQSDQASTSSQL  57.  TDTSSASGQQQESSVSSQSGQASTSSQS | 3 |
| 15 | MF771043.1 | Colombia |  | >MF771043.1 Anaplasma marginale isolate 1166 major surface protein 1a (msp1a) gene, partial cds  CCATATACTGTGCAGTACGGTTGTGCTCCCCAATTGTTAAAATTTAGTATATTAATCTTGCGATTACACG  TTCCGTATGTTACAATCGGGCTGCCGGTGTGGTAGCGTGCTGGTTGTGTGGTTGTCCTCTTTCCCGATGT  TGGGTCGTTCGTTTTACGTCTCACAAGTTTGTACGCTGTGCCCCTGGCAGTGTAGGGTTTGTTTGTGTGT  GTGTTATGTCTGAGTATGTGTCCTTTCAGCCAACTGATAGCTCGTCATCAGCGAGTGGTCAGCAGCAAGA  GAGTAGTGTGTTATCTCAAAGTGATCAGGCCAGTAGTACATCGTCTCAATTAGGAGCTGATAGCTCGTCA  GCGAGTGGTCAGCAGCAAGAGAGTAGTGTGTTATCTCAAAGTGATCAGGCCAGTACATCGTCTCAATTAG  GAACTGATTGGCGGTCACAAGAGATGCGCTCCAAGGTTGCGAGTGTTGAGTACATGTTGGCTGCTCGTGC  CCTTATTTCTGTAGGGGTCTATGCTGCTCAGGGAGAGATCGCGAGATCGCAAGGGTGTGCTCCCCTGTGT  GTTGCAGAAGTCGAAGAAATCGTGAGGGATAGCCTTGTACGCAGCCACTTTCATGATAGTGGCCTTTCAC  TAGGCTCCATACGACTCGTGCTTATGCAGGTTGGGGATAAGTTGGGGCTACAAGATTTGAAGATTGGCGA  AGGGTACGCCACCTATCTCGCGCAAGCGTTTGCTGACAGCGTGGTGGTTGCGGCTGATGCTCAGAGTGGT  GGTGCGAGCTCTGCCAGCCTTGACAGGGCGATCGCAGACGTTGAGAAGTCGTGGTCCCTGCACGGCGACC  TGATAAGCAAAGATTTTGACCGTGGTACCAAAGTAGGAAGGGGCGACCTTGAGGCTTTTGTCGACTTCAT  GTTTGGCGGTGTGTCGTACAATGATGTGAACGCGGCTGTGGCTAGGAGCGTATTGGAAACGCTTGCCGGG  CACGTCGATGCACTTGGTATATCGTACAATCAGCTGGATAAGCTTGATGCTGACACTTTGTATAGTGTCG  TATCGTTTAGTGGCGCTTCCGCAATAGACAGAGGTGCGGTTAGCGATGCGGCTGACAAGTTCCGTGTGAT  GATGTTTGGTGGTGCTCCTGCGGGGCAAGAGAAAACTGCCGAACCTGAGCATGAGGCTGCGACCCCGTCA  GCTAGTAGCGTTCCGTCAACTGTACATGGTAAGGTCGTTGATCGTGCAAAAGAAGCGGCTAAGCAGGCCT  ATGCAGGCGTGCGTAAGTGGCATGTGGCGAAGCATTCGGACACTACTACACAGCTTGTTGTAGCTATCAC  GGCGCTGCTTATCACGGCGTTTGCTATCTGTGCGTGTTTGGAACCTAGGCTTATAGGGGCGTCCGGTCCG  CTGATTTGGGGCTGCCTGGCACTAGTAGCACTGCTGCCATTACTTGGTATGGCTGTGCATACGGCAGTGA  GTGCTTCGAGTCAAAAGAAGGCTGCCGGTGGTGCGCAACGGGTTGCTGCTCAGGAGAGGTCTAGGGAATT  GTCCCGTGCGAGACAGGAAGATCAGCAGAAGTTGCATGTTCCCGCGATACTGACCGGGTTGAGCGTGCTT  GTGTTTATTGCTGCCGTCGTGGCTTGTATTGCTGTTGACGCGAAGCGCGGGACGTGGCAGGGCAGCATAT  GTTTCCTAGCCGCATTTGTGTTGTTTGCGATCAGTGCCGCTGTTGTAATGGCAACACGTGACCAATCGTT  GGCAGAAGAGTGTGATAGCAAGTGTGCTACAGCTCGTACGGCTCAAGCTGTACCCGGTGGCCAGCAGCAG  CCGCGTGCTACCGAGGGCGTTGTTAGCGGTGGCAGCCAAGAAGGCGGGGCTGGTGTTCCCGGAACTTCCG  TGCCGTCAGCCGGGTCTGGGTCCGTACCTCCTGCTACCATTATGGTCAGTGTGGATCCACAACTTGTTGC  TACTTTGGGAGCAGGTGTGGCGCAGGCGGCGGCG | AACTGATAGCTCGTCATCAGCGAGTGGTCAGCAGCAAGAGAGTAGTGTGTTATCTCAAAGTGATCAGGCCAGTAGTACATCGTCTCAATTAGG    AGCTGATAGCTCG-TCAGCGAGTGGTCAGCAGCAAGAGAGTAGTGTGTTATCTCAAAGTGATCAGGCCAGTACATCGTCTCAATTAGG | 58.  TDSSSSASGQQQESSVLSQSDQASSTSSQL  59.  ADSSSASGQQQESSVLSQSDQASTSSQL | 2 |
| 16 | MF771042.1 | Colombia |  | >MF771042.1 Anaplasma marginale isolate 1133 major surface protein 1a (msp1a) gene, partial cds  CCATATACTGTGCAGTATGGTTGTGCTCCCCAATTGTTAAAATTTACTATATTAATCTTGCGATTACACG  TTCCGTATGTTACAATCAGGCCGCCGGTGTGGTACCGTGCTGGTTGTGTGGTTGTCCTCTTTCCCGATGT  TGGGTCGTTCGTTTTACGTCCCACAAGTTTGTACGCTGTGCCCCTGGCAGTGTAGGGTTTATTTGTGTGT  GTGTGTGTTATGTCAGAAGATTATGTGTCCCCCCGGCCAGCTGATAGCTCGTCAGCGGGTGGTCAGCAGC  AAGAGAGTAGTGTGTCATCTCAAAGTGATCAGGCCAGTACATCGTCTCAATTAGGAGCTGATAGCTCGTC  AGCGGGTGGTCAGCATCAAGAGAGTAGTGTGTCATCTCAAAGTGATCAGGCCAGTACATCGTCTCAATTA  GGACCTGATACCTCGTCAGCGGGTGGTCATCACCATAAGAGTAGTGTGTCATCTCAAAGTGGTCAGGCCA  GTACATCGTCTCAATTAGGGAGTGATAGGCGGCAAGAGATGCGCTCCAAGGTTGCGAGTGTTGAGTACAT  TTTGGCTGCTCGTGCCCTTATTTCTGTAGGGGTCTATGCTGCTCAGGGAGAGATCGCGAGATCGCGAGGG  TGTGCTTCCCTGCGTGTTGCAGAAGTCGAAGAAATCGTGAGGGATGGCCTTGTACGCAGCCACTTTCATG  ATAGTGGCCTTTCACTAGGCTCCATACGACTCGTGCTTATGCAGGTTGGGGATAAGTTGGGGCTACAAGG  TTTGAAGATTGGCGAAGGGTACGCCACCTATCTCGCGCAAGCGTTTGCTGACAGCGTGGTGGTTGCGGCT  GATGCTCAGAGTGGTGGTGCGTGCCCTGCCAGCCTTGACAGCGCGATCGCAAACGTTGAGACGTCGTGGT  CCCTGCACGGCGGCCTGGTAAGCAAAGGTTTTGACCGTGATACCAAAGTAGAAAGGGGCGACCTTGAGGC  TTTTGTCGACTTCATGTTTGGCGGTGTGTCGTACAATGATGGGAACGCGTCTGCGGCTAGGAGCGTATTG  GAAACGCTTGCCGGGCACGTCGATGCACTTGGTATATCGTACAATCAGCTGGATAAGCTTGATGCTGACA  CTTTGTATAGTGTCGTATCGTTTAGTGCCGGTTCCGCAATAGACAGAGGTGCGGTTAGCGATGCGGCTGA  CAAGTTCCGTGTGATGATGTTTGGTGGTGCTCCTGCGGGGCAAGAGAAAACTGCCGAACCTGAGCATGAG  GCTGCGACCCCGTCAGCTAGTAGCGTTCCGTCAACTGTGCATGGGAAGGTCGTTGATGCCGTTGATCGTG  CAAAAGAAGCGGCTAAGCAGGCCTATGCAGGCGTGCGTAAGTGGCGTGTGGCGAAGCATTCGGACACTAC  TACACAGCTTGTTGTAGCTATCACGGCGCTGCTTATCACGGCGTTTGCTATCTGTGCGTGTTTGGAACCT  AGGCTTATAGGGGCGTCCGGTCCGCTGATTTGGGGCTGCCTGGCACTAGTAGCACTGCTGCCATTACTTG  GTATGGCTGTGCATACGGCAGTGAGTGCTTCGAGTCAAAAGAAGGCTGCCGGTGGTGCGCAACGGGTTGC  TGCTCAGGAGAGGTCTAGGGAATTGTCCCGTGCGAGACAGGAAGATCAGCAGAAGTTGCATGTTCCCGCG  ATACTGACCGGGTTGAGCGTGCTTGTGTTTATTGCTGCCGTCGTGGCTTGTATTGCTGTTGACGCGAAGC  GCGGGACGTGGCAGGGCAGCATATGTTTCCTAGCCGCATTTGTGTTGTTTGCGATCAGTGCCGCTGTTGT  AATGGCAACACGTGACCAATCGTTGGCAGAAGAGTGTGATAGCAAGTGTGCTACAGCTCGTACGGCTCAA  GCTGTACCCGGTGGCCAGCAGCAGCCGCGTGCTACCGAGGGCGTTGTTAGCGGTGGCAGCCAAGAAGGCG  GGGCTGGTGTTCCCGGAACTTCCGTGCCGTCAGCCGGGTCTGGGTCCGTACCTCCTGCTACCATTATGGT  CAGTGTGGATCCACAACTTGTTGCTACTTTGGGAGCAGGTGTGGCGCAGGCGGCGGCG | AGCTGATAGCTCGTCAGCGGGTGGTCAGCAGCAAGAGAGTAGTGTGTCATCTCAAAGTGATCAGGCCAGTACATCGTCTCAATTAGG    AGCTGATAGCTCGTCAGCGGGTGGTCAGCATCAAGAGAGTAGTGTGTCATCTCAAAGTGATCAGGCCAGTACATCGTCTCAATTAGG  ACCTGATACCTCGTCAGCGGGTGGTCATCACCATA-AGAGTAGTGTGTCATCTCAAAGTGGTCAGGCCAGTACATCGTCTCAATTAGGG | 60.  ADSSSAGGQQQESSVSSQSDQASTSSQL  61.  ADSSSAGGQHQESSVSSQSDQASTSSQL  62.  PDTSSAGGHHHKSSVSSQSGQASTSSQLG | 3 |
| 17 | MF771041.1 | Colombia |  | >MF771041.1 Anaplasma marginale isolate 1111 major surface protein 1a (msp1a) gene, partial cds  CCATATACTGTGCAGTACGGTTGTGCTCCCCAATTGTTAAAATTTAGTATATTAATCTTGCGATTACACG  TTCCGTATGTTACAATCGGGCTGCCGGTGTGGTAGCGTGCTGGTTGTGTGGTTGTCCTCTTTCCCGATGT  TGGGTCGTTCGTTTGACGTCTCACAAGTTTGTACGCTGTGCCCCTGGCAGTGTAGGGTTTGTTTGTGTGT  GTGTGTGTGTTATGTCAGCACAGTATGTGTCCACCCCCCCATGTGATAGCTCGTCAGCGGGTGGTCAGCA  GCAAGAGAGTAGTGTGTCATCTCAAAGTGATCAGGCCAGTACATCGTCTCAATTAGGAGCTGATAGCTCG  TCAGCGGGTGATCAGCAGCAAGAGAGTAGTGTGTCATCTCAAAGTGATGCCAGTACATCGTCTCAATTAG  GGACTGATAGCTCGTCAGCGGGTGATCAGCAGCAAGAGAGTAGTGTGTCATCTCAAAGTGGTCATGCCAG  TACATCGTCTCAATTAGGGACTGATTGGCGGCAAGAGATGCGCTCCAATGTTGCGAGTGTTGAGTACATG  TTGGCTGCTCGTGCCCTTATTTCTGTAGGGGTCTATGCTGCTCAGGGAGAGATCGCGAGATCGCGAGGGT  GTGCTCCCCTGCGTGTTGCAGAAGTCGAAGAAATCGTGAGGGATGGCCTTGTACGCAGCCACTTTCATGA  TAGTGGCCTTTCACTAGGCTCCATACGACTCGTGCTTATGCAGGTTGGGGATAAGTTGGGGCTACAAGGT  TTGAAGATTGGCGAAGGGTACGCCACCTATCTCGCGCAAGCGTTTGCTGACAGCGTGGTGGTTGCGGCTG  ATGCTCAGAGTGGTGGTGCGTGCCCTGCCAGCCTTGACAGCGCGATCGCAAACGTTGAGACGTCGTGGTC  CCTGCACGGCGGCCTGGTAAGCAAAGGTTTTGACCGTGATACCAAAGTAGAAAGGGGCGACCTTGAGGCT  TTTGTCGACTTCATGTTTGGCGGTGTGTCGTACAATGATGGGAACGCGTCTGCGGCTAGGAGCGTATTGG  AAACGCTTGCCGGGCACGTCGATGCACTTGGTATATCGTACAATCAGCTGGATAAGCTTGATGCTGACAC  TTTGTATAGTGTCGTATCGTTTAGTGCCGGTTCCGCAATAGACAGAGGTGCGGTTAGCGATGCGGCTGAC  AAGTTCCGTGTGATGATGTTTGGTGGTGATCATGCGGGGCAAGAGAAAACTCCCGAACCTGAGCATGAGG  CTGCGACCCCGTCAGCTAGTAGCGTTCCGTCAACTGTACATGGTAAGGTCGTTGATCGTGCAAAAGAAGC  GGCTAAGCAGGCCTATGCAGGCGTGCGTAAGTGGCGTGTGGCGAAGCATTCGGACACTACTACACAGCTT  GTTGTAGCTATCACGGCGCTGCTTATCACGGCGTTTGCTATCTGTGCGTGTTTGGAACCTAGGCTTATAG  GGGCGTCCGGTCCGCTGATTTGGGGCTGCCTGGCACTAGTAGCACTGCTGCCATTACTTGGTATGGCTGT  GCATACGGCAGTGAGTGCTTCGAGTCAAAAGAAGGCTGCCGGTGGTGCGCAACGGGTTGCTGCTCAGGAG  AGGTCTAGGGAATTGTCCCGTGCGAGACAGGAAGATCAGCAGAAGTTGCATGTTCCCGCGATACTGACCG  GGTTGAGCGTGCTTGTGTTTATTGCTGCCGTCGTGGCTTGTATTGCTGTTGACGCGAAGCGCGGGACGTG  GCAGGGCAGCATATGTTTCCTAGCCGCATTTGTGTTGTTTGCGATCAGTGCCGCTGTTGTAATGGCAACA  CGTGACCAATCGTTGGCAGAAGAGTGTGATAGCAAGTGTGCTACAGCTCGTACGGCTCAAGCTGTACCCG  GTGGCCAGCAGCAGCCGCGTGCTACCGAGGGCGTTGTTAGCGGTGGCAGCCAAGAAGGCGGGGCTGGTGT  TCCCGGAACTTCCGTGCCGTCAGCCGGGTCTGGGTCCGTACCTCCTGCTACCATTATGGTCAGTGTGGAT  CCACAACTTGTTGCTACTTTGGGAGCAGGTGTGGCGCAGGCGGCGGCG | TGATAGCTCGTCAGCGGGTGGTCAGCAGCAAGAGAGTAGTGTGTCATCTCAAAGTGATCAGGCCAGTACATCGTCTCAATTA-GGAGC  TGATAGCTCGTCAGCGGGTGATCAGCAGCAAGAGAGTAGTGTGTCATCTCAAAGTGAT--GCCAGTACATCGTCTCAATTAGGGAC  TGATAGCTCGTCAGCGGGTGATCAGCAGCAAGAGAGTAGTGTGTCATCTCAAAGTGGTCATGCCAGTACATCGTCTCAATTAGGGAC | 63.  DSSSAGGQQQESSVSSQSDQASTSSQLG  64.  DSSSAGDQQQESSVSSQSDASTSSQLG  65.  DSSSAGDQQQESSVSSQSGHASTSSQLG | 3 |
| 18 | MF771040.1 | Colombia |  | >MF771040.1 Anaplasma marginale isolate 1610 major surface protein 1a (msp1a) gene, partial cds  CCATATACTGTGCAGTACGGTTGTGCTCCCCAATTGTTAAAATTTAGTATATTAATCTTGCGATTACACG  TTCCGTATGTTACAATCGGGCTGCCGGTGTGGTAGCGTGCTGGTTGTGTGGTTGTCCTCTTTCCCGATGT  TGGGTCGTTCGTTTTACGTCTCACAAGTTTGTACGCTGTGCCCCTGGCAGTGTAGGGTTTGTTTGTGTGT  GTGTGTGTTATGTCAGAGTTGGTGTCCCTTCACCCAACTGATAGCTCGTCAGCGAGTGGTCAGCAGCAAG  AGAGTAGTGTGTTATCTCAAAGTAGTCAGGCCAGTACATCGTCTCAATTAGGAGCTGATAGCTCGTCAGC  GAGTGGTCAGCAGCAAGAGAGTAGTGTGTTATCTCAAAGTAGTCAGGCCAGTACATCGTCTCAATTAGGA  GCTGATAGCTCGTCAGCGAGTGGTCAGCAGCAAGAGAGTAGTGTGTTATCTCAAAGTGATCAGGCCAGTA  CATCGTCTCAATTAGGGACTAATTGGCGGCAAGAGATGCGCTCCAAGGTTGCGAGTGTTGAGTACATTTT  GGCTGCTCGTGCCCTTATCTCTGTAGGGGTCTATGCTGCTCAGGAAGAGATCGCGAGATCGCTAGGGCAT  GCTCCCCTGCGTGTTGCAGAAGTCGAAGAAATCGTGAGGGATAGCCTTGTACGCAGCCACTTTCATGATA  GTGGCCTTTCACTAGGCTCCATACGACTCGTGCTTATGCAGGTTGGGGATAAGTTGGGGCTACAAGGTTT  GAAGATTGGCGAAGGGTACGCCACCTATCTCGCGCAAGCGTTTGCTGACAGCGTGGTGGTTGCGGCTGAT  GCTCAGAGTGGTGGTGCGTGCCCTGCCAGCCTTGACAGCGCGATCGCAAACGTTGAGACGTCGTGGTCCC  TGCACGGCGTCCTGATAAGCAAAGGTTTTGACCGTGATACCAAAGTAAAAAGGGACGACCTTGAGGCTTT  TGTCGACTTCATGTTTGGCGGTGTGTCGTACAATGATGGGAACGCGTCTGCGGCTAGGAGAGTATTGGAA  ATGCTTGCCGGGCACGTTGATGCACTTGGTATATCGTACAATCAGCAGGATAAGCTTGATGATGACACTT  TGTATAGTGTAGTATCGTTTAGTGCCGCTTCCACAATAGACAGAGGTGCGGTTAGCGATGCGGCTGACAA  GTTCCGTGTGATGATGTTTGGTGGTGCTCCTGCGGGGCAAGAGAAAACTGCCGAACAGGAGCATGAGGCT  GCGACCCCGTCAGCTAGTAGCGTTCCGTCAACTGTGCATGGTAAGGTCGTTGATGCAGTTGACCGTGCAA  AAGAAGCGGCTAAGCAGGCCTATGCAGGCGTGCGTAAGCGGTATGTGGCGAAGCCTTCGGACACTACTAC  ACAGCTTGTTGTAGCTATCACGGCGCTGCTTATCACGGCGTTTGCTATCTGTGCGTGTTTGGAACCTAGG  CTTATAGGGGCGTCCGGTCCGCTGATTTGGGGCTGCCTGGCACTAGTAGCACTGCTGCCATTACTTGGTA  TGGCTGTGCATACGGCAGTGAGTGCTTCGAGTCAAAAGAAGGCTGCCGGTGGTGCGCAACGGGTTGCTGC  TCAGGAGAGGTCTAGGGAATTGTCCCGTGCGAGACAGGAAGATCAGCAGAAGTTGCATGTTCCCGCGATA  CTGACCGGGTTGAGCGTGCTTGTGTTTATTGCTGCCGTCGTGGCTTGTATTGCTGTTGACGCGAGGCGCG  GGACGTGGCAGGGCAGCATATGTTTCCTAGCCGCATTTGTGTTGTTTGCGATCAGTGCCGCTGTTGTAAT  GGCAACACGTGACCAATCGTTGGCAGAAGAGTGTGATAGCAAGTGTGCTACAGCTCGTACGGCTCAAGCT  GTACCCGGTGGCCAGCAGCAGCCGCGTGCTACCGAGGGCGTTGTTAGCGGTGGCAGCCAAGAAGGCGGGG  CTGGTGTTCCCGGAACTTCCGTGCCGTCAGCCGGGTCTGGGTCCGTACCTCCTGCTACCATTATGGTCAG  TGTGGATCCACAACTTGTTGCTACTTTGGGAGCAGGTGTGGCGCAGGCGGCGGCG | CTGATAGCTCGTCAGCGAGTGGTCAGCAGCAAGAGAGTAGTGTGTTATCTCAAAGTAGTCAGGCCAGTACATCGTCTCAATTAGGAG  CTGATAGCTCGTCAGCGAGTGGTCAGCAGCAAGAGAGTAGTGTGTTATCTCAAAGTAGTCAGGCCGTACATCGTCTCAATTAGGAG  CTGATAGCTCGTCAGCGAGTGGTCAGCAGCAAGAGAGTAGTGTGTTATCTCAAAGT-GATCAGGCCAGTACATCGTCTCAATTAGG | 66.  DSSSASGQQQESSVLSQSSQASTSSQLG  67.  DSSSASGQQQESSVLSQSSQASTSSQLG  68.  DSSSASGQQQESSVLSQSDQASTSSQL | 3 |
| 19 | MF771039.1 | Colombia |  | >MF771039.1 Anaplasma marginale isolate 1587 major surface protein 1a (msp1a) gene, partial cds  CCATATACTGTGCAGTACGGTTGTGCTCCCCAATTGTTAAAATTTAGTATATTAATCTTGCGATTACACG  TTCCGTATGTTACAATCGGGCTGCCGGTGTGGTAGCGTGCTGGTTGTGTGGTTGTCCTCTTTCCCGATGT  TGGGTCGTTCGTTTTACGTCTCACAAGTTTGTACGCTGTGCCCCTGGCAGTGTAGGGTTTGTTTGTGTGT  GTGTGTGTTATGTCAGAGTTGGTGTCCCTTCACCCAACTGATAACTCGTCAGCGAGGGGTCATCAGCAAG  AAAATAGTGTGGTATCTCCAAGTGGTCAGGCCAGTACATCGTCTCAATTAGGAGCTGATAGCTCGTCAGC  GAGTGATCAGCAGCAAGAGAGTAGTGTGTTATCTCAAAGTAATCAGGCCAGTACATCGTCTCAATTAGGA  GATGATAGCTCGTCAGCGAGTGATCAGCAACAAGAGAGTAGTGTGTTATCTCAAAGTGATCAGGCCAGTA  CATCGTCTCAATTAGGCACTTATTGCTCGTCAGCGGGTGGTCAGCAGCAAGAGAGTAGTGTGTCATCTCA  AAGTGGTCAGGCCAGTACATCGTCTCAATTAGGGACTGATTGGCGGCAAGAGATGCGCTCCAAGGTTGCG  AGTGTTGAGTACATGTTGGCTGCTCGTGCCCTTATCTCTGTAGGGGTCTATGCTGCTCAGGAAGAGATCG  CGAGATCGCTAGGGCATACTCCCCTGCGTGTTGCAGAAGTCGAAGCAATCGTGAGGGATAGCCTTGTACG  CAGCCACTTTCATGATAGTGGCCTTTCACTAGGCTCCATACGACTCGTGCTTATGCAGGTCGGGGATAAG  TTGGGGCTACAAGGTTTGAAGATTGGCGAAGGGTACGCCACCTATCTCGCGCAAGCGTTTGCTGACAGCG  TGGTGGTTGCGGCTGATGTTCAGAGTAGTGGTGCGTGCCCTGCTGCCAGCCTTGACAGCGCGATCGCAAA  CGTTGAGAAGTCGTGGTCCCTGCACGGCGGCCTGGTAAGCAAAGATTTTGACCGTGATACCGAAGTAAAA  AGGGGCGACCTTGAGGCTTTTGTCGACTTCATGTTTGGCGGTGTGTCGTACGATGATGGGAACGCGTCTG  CGGCTAGGAGAGTATTGAAAACGCTTGCCGGGCACGTCGATGCACTTGGTATATCGTACAATCAGCTGGA  TAAGCTTGATGCTGACACTTTGTATAGTGTCGTATCGTTTAGTGCCGGTTCCGCAATAGACAGAGGTGCG  GTTAGCGATGCGGCTGACAAGTTCCGTGTGATGATGTTTGGTGGTGCTCCTGCGGGGCAAGAGAAAACTG  CCGAACCTGAGCATGAGGCTGCGACCCCGTCAGCTAGTAGCGTTCCGTCAACTGTGCATGGTAAGGTCGT  TGATCATGCAGTTGACCGTGCAAAAGAAGCGGCTAAGCAGGCCTATGCAGGCGTGCGTAAGCGGTATGTG  GCGAAGCCTTCGGACACTACTACACAGCTTGTTGTAGCTATCACGGCGCTGCTTATCACGGCGTTTGCTA  TCTGTGCGTGTTTGGAACCTAGGCTTATAGGGGCGTCCGGTCCGCTGATTTGGGGCTGCCTGGCACTAGT  AGCACTGCTGCCATTACTTGGTATGGCTGTGCATACGGCAGTGAGTGCTTCGAGTCAAAAGAAGGCTGCC  GGTGGTGCGCAACGGGTTGCTGCTCAGGAGAGGTCTAGGGAATTGTCCCGTGCGAGACAGGAAGATCAGC  AGAAGTTGCATGTTCCCGCGATACTGACCGGGTTGAGCGTGCTTGTGTTTATTGCTGCCGTCGTGGCTTG  TATTGCTGTTGACGCGAGGCGCGGGACGTGGCAGGGCAGCATATGTTTCCTAGCCGCATTTGTGTTGTTT  GCGATCAGTGCCGCTGTTGTAATGGCAACACGTGACCAATCGTTGGCAGAAGAGTGTGATAGCAAGTGTG  CTACAGCTCGTACGGCTCAAGCTGTACCCGGTGGCCAGCAGCAGCCGCGTGCTACCGAGGGCGTTGTTAG  CGGTGGCAGCCAAGAAGGCGGGGCTGGTGTTCCCGGAACTTCCGTGCCGTCAGCCGGGTCTGGGTCCGTA  CCTCCTGCTACCATTATGGTCAGTGTGGATCCACAACTTGTTGCTACTTTGGGAGCAGGTGTGGCGCAGG  CGGCGGCG | CTGATAACTCGTCAGCGAGGGGTCATCAGCAAGAAAATAGTGTGGTATCTCCAAGTGGTCAGGCCAGTACATCGTCTCAATTAGGAG  CTGATAGCTCGTCAGCGAGTGATCAGCAGCAAGAGAGTAGTGTGTTATCTCAAAGTAATCAGGCCAGTACATCGTCTCAATTAGGAG  ATGATAGCTCGTCAGCGAGTGATCAGCAACAAGAGAGTAGTGTGTTATCTCAAAGTGATCAGGCCAGTACATCGTCTCAATTAGGCA-  CTTATTGCTCGTCAGCGGGTGGTCAGCAGCAAGAGAGTAGTGTGTCATCTCAAAGTGGTCAGGCCAGTACATCGTCTCAATTAGG | 69.  DNSSARGHQQENSVVSPSGQASTSSQLG  70.  DSSSASDQQQESSVLSQSNQASTSSQLG  71.  DSSSASDQQQESSVLSQSDQASTSSQLG  72.  YCSSAGGQQQESSVSSQSGQASTSSQL | 4 |
| 20 | MF771038.1 | Colombia |  | >MF771038.1 Anaplasma marginale isolate 1528 major surface protein 1a (msp1a) gene, partial cds  CCATATACTGTGCAGTATGGTTGTGCTCCCCAATTGTTAAAATTTAGTATATTAATCTTGCGATTACACG  TTCCGTATGTTACAATCAGGCCGCCGGTGTGGTAGCGTGCTGGTTGTGTGGTTGTCCTCTTTCCCGATGT  TGGGTCGTTCGTTTTACGTCGCACAAGTTTGTACGCTGTGCCCCTGGCAGTGTAGGGTTTATTTGTGTGT  GTGTGTGTTATGTCAGAGTTGGTGTCCCTTCACCCAACTGATAGCTCGTCAGCGGGTGATCAGCAGCAAG  AGAGTAGTGTGTTATCTCAAAGTGGTCAGGCCAGTACATCGTCTCAATTAGGAGCTGATAGCTCGTCAGC  GAGTGATCAGCAGCAAGAGAGTAGTGTGTTATCTCAAAGTAATCAGGCTAGTACATCGTCTCTATTAGGA  GATAATAGCTCGTCAGCTAGTGATCAGCAACAAGATAGTATTGTGTTATCTCAAAGTGATCAGGCCAGTA  CATCGTCTCAATTAGGGACTGATTGGCGGCAAGAGATGCGCTCCAAGGTTGCGAGTGTTGAGTACATTTT  GGCTGCTCGTGCCCTTATCTCTGTAGGGGTCTATGCTGCTCAGGAAGAGATCGCGAGATCGCTAGGGCAT  GCTCCCCTGCGTGTTGCAGAAGTCGAAGAAATCGTGAGGGATAGCCTTGTACGCAGCCACTTTCATGATA  GTGGCCTTTCACTAGGCTCCATACGACTCGTGCTTATGCAGGTTGGGGATAAGTTGGGGCTACAAGGTTT  GAAGATTGGCGAAGGGTACGCCACCTATCTCGCGCAAGCGTTTGCTGACAGCGTGGTGGTTGCGGCTGAT  GCTCAGAGTGGTGGTGCGTGCCCTGCCAGCCTTGACAGCGCGATCGCAAACGTTGAGACGTCGTGGTCCC  TGCACGGCGGCCTGGTAAGCAAAGGTTTTGACCGTGATACCAAAGTAGAAAGGGGCGACCTTGAGGCTTT  TGTCGACTTCATGTTTGGCGGTGTGTCGTACAATGATGGGAACGCGTCTGCGGCTAGGAGCGTATTGGAA  ACGCTTGCCGGGCACGTCGATGCACTTGGTATATCGTACAATCAGCTGGATAAGCTTGATGCTGACACTT  TGTATAGTGTCGTATCGTTTAGTGCCGGTTCCGCAATAGACAGAGGTGCGGTTAGCGATGCGGCTGACAA  GTTCCGTGTGATGATGTTTGGTGGTGCTCCTGCGGGGCAAGAGAAAACTGCCGAACCTGAGCATGAGGCT  GCGACCCCGTCAGCTAGTAGCGTTCCGTCAACTGTGCATGGTAAGGTCGTTGATGCAGTTGACCGTGCAA  AAGAAGCGGCTAAGCAGGCCTATGCAGGCGTGCGTAAGTGGTATGTGGCGAAGCCTTCGGACACTACTAC  ACAGCTTGTTGTAGCTATCACGGCGCTGCTTATCACGGCGTTTGCTATCTGTGCGTGTTTGGAACCTAGG  CTTATAGGGGCGTCCGGTCCGCTGATTTGGGGCTGCCTGGCACTAGTAGCACTGCTGCCATTACTTGGTA  TGGCTGTGCATACGGCAGTGAGTGCTTCGAGTCAAAAGAAGGCTGCCGGTGGTGCGCAACGGGTTGCTGC  TCAGGAGAGGTCTAGGGAATTGTCCCGTGCGAGACAGGAAGATCAGCAGAAGTTGCATGTTCCCGCGATA  CTGACCGGGTTGAGCGTGCTTGTGTTTATTGCTGCCGTCGTGGCTTGTATTGCTGTTGACGCGAAGCGCG  GGACGTGGCAGGGCAGCATATGTTTCCTAGCCGCATTTGTGTTGTTTGCGATCAGTGCCGCTGTTGTAAT  GGCAACACGTGACCAATCGTTGGCAGAAGAGTGTGATAGCAAGTGTGCTACAGCTCGTACGGCTCAAGCT  GTACCCGGTGGCCAGCAGCAGCCGCGTGCTACCGAGGGCGTTGTTAGCGGTGGCAGCCAAGAAGGCGGGG  CTGGTGTTCCCGGAACTTCCGTGCCGTCAGCCGGGTCTGGGTCCGTACCTCCTGCTACCATTATGGTCAG  TGTGGATCCACAACTTGTTGCTACTTTGGGAGCAGGTGTGGCGCAGGCGGCGGCG | CTGATAGCTCGTCAGCGGGTGATCAGCAGCAAGAGAGTAGTGTGTTATCTCAAAGTGGTCAGGCCAGTACATCGTCTCAATTAGGAG  CTGATAGCTCGTCAGCGAGTGATCAGCAGCAAGAGAGTAGTGTGTTATCTCAAAGTAATCAGGCTAGTACATCGTCTCTATTAGGAG  ATAATAGCTCGTCAGCTAGTGATCAGCAACAAGATAGTATTGTGTTATCTCAAAGTGATCAGGCCAGTACATCGTCTCAATTAGG | 73.  DSSSAGDQQQESSVLSQSGQASTSSQLG  74.  DSSSASDQQQESSVLSQSNQASTSSLLG  75.  NSSSASDQQQDSIVLSQSDQASTSSQL | 3 |
| 21 | MF771037.1 | Colombia |  | >MF771037.1 Anaplasma marginale isolate 1513 major surface protein 1a (msp1a) gene, partial cds  CCATATACTGTGCAGTACGGTTGTGCTCCCCAATTGTTAAAATTTAGTATATTAATCTTGCGATTACACG  TTCCGTATGTTACAATCGGGCTGCCGGTGTGGTAGCGTGCTGGTTGTGTGGTTGTCCTCTTTCCCGATGT  TGGGTCGTTCGTTTTACGTCTCACAAGTTTGTACGCTGTGCCCCTGGCAGTGTAGGGTTTGTTTGTGTGT  GTGTTATGTCAGTCATTGTGTCATCCCACTCAACTGCTCGTCAGCTCGGTGGTCAGCAGCAAGAGAGTAG  TGTGTTATCTCAAAGTGATCAGGCCAGTAGTACATCGTCTCAATTAGGAGCTGATAGCTCGTCAGCGAGT  GGTCAGCAGCAAGAGAGTAGTGTGTTATCTCAAAGTGATCAGGCCAGTACATCGTCTCAATTAGGAACTG  ATAGCTCGTCAGCGAGTGGTCAGCAAGAGAGTAGTGTGTTATCTCAAAGTGATCAGGCCAGTACATCGTC  TCAATCAGGAACTGATTGGCGGCAAGAGATGCGCTCCAAGGTTGCGAGTGTTGAGTACATGTTGGCTGCT  CGTGCCCTTATTTCTGTAGGGGTCTATGCTGCTCAGGGAGAGATCGCGAGATCGCGAGGGTGTGCTCCCC  TGCGTGTTGCAGAAGTCGAAGAAATCGTGAGGGATAGCCTTGTACGCAGCCACTTTCATGATAGTGGCCT  TTCACTAGGCTCCATACGACTCGTGCTTATGCAGGTTGGGGATAAGTTGGGGCTACAAGGTTTGAAGATT  GGCGAAGGGTACGCCACCTATCTCGCGCAAGCGTTTGCTGACAGCGTGGTGGTTGCGGCTGATGCTCAGA  GTGGTGGTGCGTGCCCTGCCAGCCTTGACAGCGCGATCGCAAACGTTGAGACGTCGTGGTCCCTGCACGG  CGGCCTGGTAAGCAAAGATTTTGACCGTGATGCCAAAGTAGGAAGGGACGACCTTGAGGCTTTTATCGAC  TTCATGTTTGGCTATGAGTACTACAATGATGTGAACGCGGCTGTGGTTAGGAGAGTATTGGAAACGCTTG  CCGGGCACGTTGATGCACTTGGTATATCGTACAATCAGCTGGATAAGCTTGATGCTGACACTTTGTATAG  TGTCGTATCGTTTAGTGGCGCTTCCGCAATAGACAGAGGTGCGGTTAGCGATGCGGCTGACAAGTTCCGT  GTGATGATGTTTGGTGGTGCTCATGAGGGGCAAGAGAAAACTGCCGAACAGGAGCATGAGGCTGCGACCC  CGTCAGCTAGTAGCGTTCCGTCAACTGTACATGGTAAGGTCGTTGATGCAGTTGACCGTGCAAAAGAAGC  GGCTAAGCAGGCCTATGCAGGCGTGCGTAAGTGGTATGTGGCGAAGCCTTCGGACACTACTACACAGCTT  GTTGTAGCTATCACGGCGCTGCTTATCACGGCGTTTGCTATCTGTGCGTGTTTGGAACCTAGGCTTATAG  GGGCGTCCGGTCCGCTGATTTGGGGCTGCCTGGCACTAGTAGCACTGCTGCCATTACTTGGTATGGCTGT  GCATACGGCAGTGAGTGCTTCGAGTCAAAAGAAGGCTGCCGGTGGTGCGCAACGGGTTGCTGCTCAGGAG  AGGTCTAGGGAATTGTCCCGTGCGAGACAGGAAGATCAGCAGAAGTTGCATGTTCCCGCGATACTGACCG  GGTTGAGCGTGCTTGTGTTTATTGCTGCCGTCGTGGCTTGTATTGCTGTTGACGCGAAGCGCGGGACGTG  GCAGGGCAGCATATGTTTCCTAGCCGCATTTGTGTTGTTTGCGATCAGTGCCGCTGTTGTAATGGCAACA  CGTGACCAATCGTTGGCAGAAGAGTGTGATAGCAAGTGTGCTACAGCTCGTACGGCTCAAGCTGTACCCG  GTGGCCAGCAGCAGCCGCGTGCTACCGAGGGCGTTGTTAGCGGTGGCAGCCAAGAAGGCGGGGCTGGTGT  TCCCGGAACTTCCGTGCCGTCAGCCGGGTCTGGGTCCGTACCTCCTGCTACCATTATGGTCAGTGTGGAT  CCACAACTTGTTGCTACTTTGGGAGCAGGTGTGGCGCAGGCGGCGGCG | GCTCGTCAGCTCG-GTGGTCAGCAGCAAGAGAGTAGTGTGTTATCTCAAAGTGATCAGGCCAGTAGTACATCGTCTCAATTAGGAGCTGATA  GCTCGTCAGCGAGTGGTCAGCAGCAAGAGAGTAGTGTGTTATCTCAAAGTGATCAGGCCAGTACATCGTCTCAATTAGGAACTGATA  GCTCGTCAGCGAGTGGT---CAGCAAGAGAGTAGTGTGTTATCTCAAAGTGATCAGGCCAGTACATCGTCTCAATCAGGAACTGAT | 76.  ARQLGGQQQESSVLSQSDQASSTSSQLGAD  77.  SSASGQQQESSVLSQSDQASTSSQLGTD  78.  SSASGQQESSVLSQSDQASTSSQSGTD | 3 |
| 22 | MF771036.1 | Colombia |  | >MF771036.1 Anaplasma marginale isolate 1484 major surface protein 1a (msp1a) gene, partial cds  CCATATACTGTGCAGTACGGTTGTGCTCCCCAATTGTTAAAATTTAGTATATTAATCTTGCGATTACACG  TTCCGTATGTTACAATCGGGCTGCCGGTGTGGTAGCGTGCTGGTTGTGTGGTTGTCCTCTTTCCCGATGT  TGGGTCGTTCGTTTTACGTCTCACAAGTTTGTACGCTGTGCCCCTGGCAGTGTAGGGTTTGTTTGTGTGT  GTGTTATGTCAGTCCTTGTGTCAGCCAACTCAACTGATCTCTCAGCGAGTGGTCAGCAGCAAGAGAGTAG  TGTGTTATCTCAAAGTGATCAGGCCAGTAGTACATCGTCTCAATTAGGAGCTGATAGCTCGTCAGCGAGT  GGTCAGCAGCAAGAGAGTAGTGTGTTATCTCAAAGTGATCAGGCCAGTACATCGTCTCAATCAGGAACTG  ATAGCTCGTCAGCGAGTGGTCAGCAGCAAGAGAGTAGTGTGTTATCTCAAAGTGATCAGGCCAGTACATC  GTCTCAATTAGGAGCTAATTGGCGGCAAGAGATGCGCTCCAAGGTTGCGAGTGTTGAGTACATTTTGGCT  GCTCGTGCCCTTATTTCTGTAGGGGTCTATGCTGCTCAGGGAGAGATCGCGAAATCGCAAGGGTGTGCTC  CCCTGCGTGTTGCAGAAGTCGAAGAAATCGTGAGGGATGGCCTTGTACGCAGCCACTTTCATGATAGTGG  CCTTTCACTAGGCTCCATACGACTCGTGCTTATGCAGGTTGGGGATAAGTTGGGGCTACAAGGTTTGAAG  ATTGGCGAAGGGTACGCCACCTATCTCGCGCAAGCGTTTGCTGACAACGTGGTGGTTGCGGCTGATGTTC  AAAGTGGTGGTGCGTGCTCTGCCAGCCTTGACAGCGCGATCGCAAACGTTGAGACGTCGTGGTCCCTGCA  CGGCGGCCTGATAAGCAAAGATTTTGACCGTGGTACCAAAGTAGGAAGGGGCGACCTTGAGGCTTTTGTC  GACTTCATGTTTGGCGGTGTGTCGTACAATGATGTGAACGCGGCTGTGGCTAGGAGCGTATTGGAAACGC  TTGCCGGGCACGTCGATGCACTTGGTATATCGTACAATCAGCTGGATAAGCTTGATGCTGACACTTTGTA  TAGTGTCGTATCGTTTAGTGGCGCTTCCGCAATAGACAGAGGTGCGGTTAGCGATGCGGCTGACAAGTTC  CGTGTGATGATGTTTGGTGGTGCTCCTGCGGGGCAAGAGAAAACTGCCGAACCTGAGCATGAGGCTGCGA  CCCCGTCAGCTAGTAGCGTTCCGTCAACTGTACATGGTAAGGTCGTTGATGCAGTTGACCGTGCAAAAGA  AGCGGCTAAGCAGGCCTATGCAGGCGTGCGTAAGTGGTATGTGGCGAAGCCTTCGGACACTACTACACAG  CTTGTTGTAGCTATCACGGCGCTGCTTATCACGGCGTTTGCTATCTGTGCGTGTTTGGAACCTAGGCTTA  TAGGGGCGTCCGGTCCGCTGATTTGGGGCTGCCTGGCACTAGTAGCACTGCTGCCATTACTTGGTATGGC  TGTGCATACGGCAGTGAGTGCTTCGAGTCAAAAGAAGGCTGCCGGTGGTGCGCAACGGGTTGCTGCTCAG  GAGAGGTCTAGGGAATTGTCCCGTGCGAGACAGGAAGATCAGCAGAAGTTGCATGTTCCCGCGATACTGA  CCGGGTTGAGCGTGCTTGTGTTTATTGCTGCCGTCGTGGCTTGTATTGCTGTTGACGCGAAGCGCGGGAC  GTGGCAGGGCAGCATATGTTTCCTAGCCGCATTTGTGTTGTTTGCGATCAGTGCCGCTGTTGTAATGGCA  ACACGTGACCAATCGTTGGCAGAAGAGTGTGATAGCAAGTGTGCTACAGCTCGTACGGCTCAAGCTGTAC  CCGGTGGCCAGCAGCAGCCGCGTGCTACCGAGGGCGTTGTTAGCGGTGGCAGCCAAGAAGGCGGGGCTGG  TGTTCCCGGAACTTCCGTGCCGTCAGCCGGGTCTGGGTCCGTACCTCCTGCTACCATTATGGTCAGTGTG  GATCCACAACTTGTTGCTACTTTGGGAGCAGGTGTGGCGCAGGCGGCGGCG | TCAGCGAGTGGTCAGCAGCAAGAGAGTAGTGTGTTATCTCAAAGTGATCAGGCCAGTAGTACATCGTCTCAATTAGGAGCTGATAGCTCG  TCAGCGAGTGGTCAGCAGCAAGAGAGTAGTGTGTTATCTCAAAGTGATCAGGCCAGTACATCGTCTCAATCAGGAACTGATAGCTCG  TCAGCGAGTGGTCAGCAGCAAGAGAGTAGTGTGTTATCTCAAAGTGATCAGGCCAGTACATCGTCTCAATTAGGAGCT | 79.  SASGQQQESSVLSQSDQASSTSSQLGADSS  80.  SASGQQQESSVLSQSDQASTSSQSGTDSS  81.  SASGQQQESSVLSQSDQASTSSQLGA | 3 |
| 23 | MF771035.1 | Colombia |  | >MF771035.1 Anaplasma marginale isolate 1419 major surface protein 1a (msp1a) gene, partial cds  CCATATACTGTGCAGTACGGTTGTGCTCCCCAATTGTTAAAATTTAGTATATTAATCTTGCGATTACACG  TTCCGTATGTTACAATCGGGCTGCCGGTGTGGTAGCGTGCTGGTTGTGTGGTTGTCCTCTTTCCCGATGT  TGGGTCGTTCGTTTTACGTCTCACAAGTTTGTACGCTGTGCCCCTGGCAGTGTAGGGTTTGTTTGTGTGT  GTGTGTGTTATGTCAGAGTATGTGTCCCCACCCACCACTGATAGCTCGTCAGCGGGTGATCAGCAGCAAG  AGAGTAGTGTGTTATCTCAAAGTGATCAGGCCAGTACATCGTCTCAATTAGGAGCTGATAGCTCGTCAGC  GAGTGGTCAGCAGCAAGAGAGTAGTGTGTTATCTCAAAGTGCTCAGGCCAGTACATCGTCTCAATTAGGA  ACTGATAACTCGTCAGCGAGTGATCAGCAGCAAGAGAGTAGTGTGTCTTCTCAAAGTGGTCGGGCCAGTA  CATCGTCTCAATTAGGGACTGATTGGCGGCAAGAGATGCGCTCCAAGGTTGCGAGTGTTGAGTACATTTT  GGCTGCTCGTGCCCTTATCTCTGTAGGGGTCTATGCTGCTCAGGAAGAGATCGCGAAATCGCTAGGGTAT  GCTCCCCTGCGTGTTGCAGAAGTCGAAGAAATCGTGAGGGATAGCCTTGTACGCAGCCACTTTCATGATA  GTGGCCTTTCACTAGGCTCCATACGACTCGTGCTTATGCAGGTTGGGGATAAGTTGGGGCTACAAGGTTT  GAAGATTGGCGAAGGGTACGCCACCTATCTCGCGCAAGCGTTTGCTGACAACGTGGTGGTTGCGGCTGAT  GTTCAAAGTGGTGGTGCGTGCTCTGCCAGCCTTGACAGCGCGATCGCAAACGTTGAGACGTCGTGGTCCC  TGCACGGCGGCCTGGTAAGCAAAGATTTTGACCGTGATACCAAAGTAGAAAGGGGCGACCTTGAGGCTTT  TGTCGACTTCATGTTTGGCGGTGTGTCGTACAATGATGGGAACGCGTCTGCGGCTAGGAGCGTATTGGAA  ACGCTTGCCGGGCACGTCGATGCACTTGGTATATCGTACAATCAGCTGGATAAGCTTGATGCTGACACTT  TGTATAGTGTCGTATCGTTTAGTGCCGGTTCCGCAATAGACAGAGGTGCGGTTAGCGATGCGGCTGACAA  GTTCCGTGTGATGATGTTTGGTGGTGCTCCTGCGGGGCAAGAGAAAACTGCCGAACCTGAGCATGAGGCT  GCGACCCCGTCAGCTAGTAGCGTTCCGTCAACTGTGCATGGTAAGGTCGTTGATGCAGTTGACCGTGCAA  AAGAAGCGGCTAAGCAGGCCTATGCAGGCGTGCGTAAGTGGTATGTGGCGAAGCCTTCGGACACTACTAC  ACAGCTTGTTGTAGCTATCACGGCGCTGCTTATCACGGCGTTTGCTATCTGTGCGTGTTTGGAACCTAGG  CTTATAGGGGCGTCCGGTCCGCTGATTTGGGGCTGCCTGGCACTAGTAGCACTGCTGCCATTACTTGGTA  TGGCTGTGCATACGGCAGTGAGTGCTTCGAGTCAAAAGAAGGCTGCCGGTGGTGCGCAACGGGTTGCTGC  TCAGGAGAGGTCTAGGGAATTGTCCCGTGCGAGACAGGAAGATCAGCAGAAGTTGCATGTTCCCGCGATA  CTGACCGGGTTGAGCGTGCTTGTGTTTATTGCTGCCGTCGTGGCTTGTATTGCTGTTGACGCGAAGCGCG  GGACGTGGCAGGGCAGCATATGTTTCCTAGCCGCATTTGTGTTGTTTGCGATCAGTGCCGCTGTTGTAAT  GGCAACACGTGACCAATCGTTGGCAGAAGAGTGTGATAGCAAGTGTGCTACAGCTCGTACGGCTCAAGCT  GTACCCGGTGGCCAGCAGCAGCCGCGTGCTACCGAGGGCGTTGTTAGCGGTGGCAGCCAAGAAGGCGGGG  CTGGTGTTCCCGGAACTTCCGTGCCGTCAGCCGGGTCTGGGTCCGTACCTCCTGCTACCATTATGGTCAG  TGTGGATCCACAACTTGTTGCTACTTTGGGAGCAGGTGTGGCGCAGGCGGCGGCG | ACTGATAGCTCGTCAGCGGGTGATCAGCAGCAAGAGAGTAGTGTGTTATCTCAAAGTGATCAGGCCAGTACATCGTCTCAATTAGGA  GCTGATAGCTCGTCAGCGAGTGGTCAGCAGCAAGAGAGTAGTGTGTTATCTCAAAGTGCTCAGGCCAGTACATCGTCTCAATTAGGA  ACTGATAACTCGTCAGCGAGTGATCAGCAGCAAGAGAGTAGTGTGTCT-TCTCAAAGTGGTCGGGCCAGTACATCGTCTCAATTAGGG | 82.  TDSSSAGDQQQESSVLSQSDQASTSSQLG  83.  ADSSSASGQQQESSVLSQSAQASTSSQLG  84.  DNSSASDQQQESSVSSQSGRASTSSQLG | 3 |
| 24 | MF771034.1 | Colombia |  | >MF771034.1 Anaplasma marginale isolate 1376 major surface protein 1a (msp1a) gene, partial cds  CCATATACTGTGCAGTACGGTTGTGCTCCCCAATTGTTAAAATTTAGTATATTAATCTTGCGATTACACG  TTCCGTATGTTACAATCGGGCTGCCGGTGTGGTAGCGTGCTGGTTGTGTGGTTGTCCTCTTTCCCGATGT  TGGGTCGTTCGTTTTACGTCTCACAAGTTTGTACGCTGTGCCCCTGGCAGTGTAGGGTTTGTTTGTGTGT  GTGTGTGTTATGTCAGAGTATGTGTCCCCCCAGCCAGCAGAAACTGATAGCTCGTCAGCGGGTGGTCAGC  AGCAAGAGAGTAGTGTGTCATCTCAAAGTGATCAGGCCAGTACATCGTCTCAATTAGGAGCTGATAGCTC  GTCAGCGAGTGGTCAGCAGCAAGAGAGTAGTGTGTCATCTCAAAGTGGTCAGGCCAGTACATCGTCTCAA  TTAGGAACTGATAGCTCGTCAGCGAGTGGTCAGCAGCAAGAGAGTAGTGTGTTATCTCAAAGTGGTCAGG  CCAGTACATCGTCTCAATCAGGAGCTAATTGGCGGCAAGAGATGCGCTCCAAGGTTGCGAGTGTTGAGTA  CATTTTGGCTGCTCGTGCCCTTATTTCTGTAGGGGTCTATGCTGCTCAGGGAGAGATCGCGAAATCGCAA  GGGTGTGCTCCCCTGCGTGTTGCAGAAGTCGAAGAAATCGTGAGGGATGGCCTTGTACGCAGCCACTTTC  ATGATAGTGGCCTTTCACTAGGCTCCATACGACTCGTGCTTATGCAGGTTGGGGATAAGTTGGGGCTACA  AGGTTTGAAGATTGGCGAAGGGTACGCCACCTATCTCGCGCAAGCGTTTGCTGACAACGTGGTGGTTGCG  GCTGATGTTCAAAGTGGTGGTGCGTGCTCTGCCAGCCTTGACAGCGCGATCGCAAACGTTGAGACGTCGT  GGTCCCTGCACGGCGGCCTGGTAAGCAAAGATTTTGACCGTGATACCAAAGTAGAAAGGGGCGACCTTGA  GGCTTTTGTCGACTTCATGTTTGGCGGTGTGTCGTACAATGATGGGAACGCGTCTGCGGCTAGGAGCGTA  TTGGAAACGCTTGCCGGGCACGTCGATGCACTTGGTATATCGTACAATCAGCTGGATAAGCTTGATGCTG  ACACTTTGTATAGTGTCGTATCGTTTAGTGCCGGTTCCGCAATAGACAGAGGTGCGGTTAGCGATGCGGC  TGACAAGTTCCGTGTGATGATGTTTGGTGGTGCTCCTGCGGGGCAAGAGAAAACTGCCGAACCTGAGCAT  GAGGCTGCGACCCCGTCAGCTAGTAGCGTTCCGTCAACTGTGCATGGTAAGGTCGTTGATGCAGTTGACC  GTGCAAAAGAAGCGGCTAAGCAGGCCTATGCAGGCGTGCGTAAGTGGTATGTGGCGAAGCCTTCGGACAC  TACTACACAGCTTGTTGTAGCTATCACGGCGCTGCTTATCACGGCGTTTGCTATCTGTGCGTGTTTGGAA  CCTAGGCTTATAGGGGCGTCCGGTCCGCTGATTTGGGGCTGCCTGGCACTAGTAGCACTGCTGCCATTAC  TTGGTATGGCTGTGCATACGGCAGTGAGTGCTTCGAGTCAAAAGAAGGCTGCCGGTGGTGCGCAACGGGT  TGCTGCTCAGGAGAGGTCTAGGGAATTGTCCCGTGCGAGACAGGAAGATCAGCAGAAGTTGCATGTTCCC  GCGATACTGACCGGGTTGAGCGTGCTTGTGTTTATTGCTGCCGTCGTGGCTTGTATTGCTGTTGACGCGA  AGCGCGGGACGTGGCAGGGCAGCATATGTTTCCTAGCCGCATTTGTGTTGTTTGCGATCAGTGCCGCTGT  TGTAATGGCAACACGTGACCAATCGTTGGCAGAAGAGTGTGATAGCAAGTGTGCTACAGCTCGTACGGCT  CAAGCTGTACCCGGTGGCCAGCAGCAGCCGCGTGCTACCGAGGGCGTTGTTAGCGGTGGCAGCCAAGAAG  GCGGGGCTGGTGTTCCCGGAACTTCCGTGCCGTCAGCCGGGTCTGGGTCCGTACCTCCTGCTACCATTAT  GGTCAGTGTGGATCCACAACTTGTTGCTACTTTGGGAGCAGGTGTGGCGCAGGCGGCGGCG | CTGATAGCTCGTCAGCGGGTGGTCAGCAGCAAGAGAGTAGTGTGTCATCTCAAAGTGATCAGGCCAGTACATCGTCTCAATTAGGAG  CTGATAGCTCGTCAGCGAGTGGTCAGCAGCAAGAGAGTAGTGTGTCATCTCAAAGTGGTCAGGCCAGTACATCGTCTCAATTAGGAA  CTGATAGCTCGTCAGCGAGTGGTCAGCAGCAAGAGAGTAGTGTGTTATCTCAAAGTGGTCAGGCCAGTACATCGTCTCAATCAGGAG | 85.  DSSSAGGQQQESSVSSQSDQASTSSQLG  86.  DSSSASGQQQESSVSSQSGQASTSSQLG  87.  DSSSASGQQQESSVLSQSGQASTSSQSG | 3 |
| 25 | MF771033.1 | Colombia |  | >MF771033.1 Anaplasma marginale isolate 1104 major surface protein 1a (msp1a) gene, partial cds  CCATATACTGTGCAGTACGGTTGTGCTCCCCAATTGTTAAAATTTAGTATATTAATCTTGCGATTACACG  TTCCGTATGTTACAATCGGGCTGCCGGTGTGGTAGCGTGCTGGTTGTGTGGTTGTCCTCTTTCCCGATGT  TGGGTCGTTCGTTTTACGTCTCACAAGTTTGTACGCTGTGCCCCTGGCAGTGTAGGGTTTGTTTGTGTGT  GTGTGTGTTATGTCAGAGTTGGTGTCCCTTCAGCCAACTGATAGCTCGTCAGCGAGTGGTCAGCAGCAAG  AGAGTAGTGTGTTATCTCAAAGTGGTCAGGCCAGTACATCGTCTCAATTAGGAGCTGATAGCTCGTCAGC  GAGTGGTCAGCAGCAAGAGAGTAGTGTGTTATCTCAAAGTAGTCAGGCCAGTACATCGTCTCAATTAGGA  GCTGATAGCTCGTCAGCGAGTGGTCAGCAGCAAGAGAGTAGTGTGTTATCTCAAAGTGATCAGGCCAGTA  CATCGTCTCAATTAGGAGCTGATAGCTCGTCAGCGAGTGGTCAGCAGCAAGAGAGTAGTGTGTTATCTCA  AAGTGATCAGGCCAGTACATCGTCTCAATTAGGGACTGATTGGCGGCAAGAGATGCGCTCCAAGGTTGCG  AGTGTTGAGTACATTTTGGCTGCTCGTGCCCTTATCTCTGTAGGGGTCTATGCTGCTCAGGAAGAGATCG  CGAGATCGCTAGGGCATACTCCCCTGCGTGTTGCAGAAGTCGAAGAAATCGTGAGGGATAGCCTTGTACG  CAGCCACTTTCATGATAGTGGCCTTTCACTAGGCTCCATACGACTCGTGCTTATGCAGGTTGGGGATAAG  TTGGGGCTACAAGGTTCGAAGATTAGCGAAGGGTACGCCACCTATCTCGCGCAAGCGTTTGCTGACAGCG  TGGTGGTTGCGGCTGATGTTCAGAGTGGTGGTGCGCGCTCTGCTACCAGCCTTGACAAGGCGATCGCAGA  CGTTGAGACGTCGTGGTCCCTGCGCGGCGACCTGATAAGCAAAGATTTTGACCGTGATACCAAAGTAGGA  AGGGACGACCTTGAGGCTTTTGTCGACTTCATGTTTGGCGGTGTGTCGTACAATGATGCGAACGCGGCTG  TGGCTAGGAGCGTATTGGAAACGCTTGCCGGGCACGTCGATGCACTTGGTATATCGTACAATCAGCTGGA  TAAGCTTGATGCTGACACTTTGTATAGTGTCGTATCGTTTAGTGGCGCTTCCGCAATAGACAGAGGTGCG  GTTAGCGATGCGGCTGACAAGTTCCGTGTGATGATGTTTGGTGGTGCTCCTGCGGGGCAAGAGAAAACTG  CCGAACCTGAGCATGAGGCTGCGACCCCGTCAGCTAGTAGCGTTCCGTCAACTGTGCATGGTAAGGTCGT  TGATCATGCAGTTGACCGTGCAAAAGAAGCGGCTAAGCAGGCCTATGCAGGCGTGCGTAAGTGGTATGTG  GCGAAGCCTTCGGACACTACTACACAGCTTGTTGTAGCTATCACGGCGCTGCTTATCACGGCGTTTGCTA  TCTGTGCGTGTTTGGAACCTAGGCTTATAGGGGCGTCCGGTCCGCTGATTTGGGGCTGCCTGGCACTAGT  AGCACTGCTGCCATTACTTGGTATGGCTGTGCATACGGCAGTGAGTGCTTCGAGTCAAAAGAAGGCTGCC  GGTGGTGCGCAACGGGTTGCTGCTCAGGAGAGGTCTAGGGAATTGTCCCGTGCGAGACAGGAAGATCAGC  AGAAGTTGCATGTTCCCGCGATACTGACCGGGTTGAGCGTGCTTGTGTTTATTGCTGCCGTCGTGGCTTG  TATTGCTGTTGACGCGAGGCGCGGGACGTGGCAGGGCAGCATATGTTTCCTAGCCGCATTTGTGTTGTTT  GCGATCAGTGCCGCTGTTGTAATGGCAACACGTGACCAATCGTTGGCAGAAGAGTGTGATAGCAAGTGTG  CTACAGCTCGTACGGCTCAAGCTGTACCCGGTGGCCAGCAGCAGCCGCGTGCTACCGAGGGCGTTGTTAG  CGGTGGCAGCCAAGAAGGCGGGGCTGGTGTTCCCGGAACTTCCGTGCCGTCAGCCGGGTCTGGGTCCGTA  CCTCCTGCTACCATTATGGTCAGTGTGGATCCACAACTTGTTGCTACTTTGGGAGCAGGTGTGGCGCAGG  CGGCGGCG | CTGATAGCTCGTCAGCGAGTGGTCAGCAGCAAGAGAGTAGTGTGTTATCTCAAAGTGGTCAGGCCAGTACATCGTCTCAATTAGGAG  CTGATAGCTCGTCAGCGAGTGGTCAGCAGCAAGAGAGTAGTGTGTTATCTCAAAGT-AGTCAGGCCAGTACATCGTCTCAATTAGGAG  CTGATAGCTCGTCAGCGAGTGGTCAGCAGCAAGAGAGTAGTGTGTTATCTCAAAGTGATCAGGCCAGTACATCGTCTCAATTAGGAG  CTGATAGCTCGTCAGCGAGTGGTCAGCAGCAAGAGAGTAGTGTGTTATCTCAAAGTGATCAGGCCAGTACATCGTCTCAATTAGG | 88.  DSSSASGQQQESSVLSQSGQASTSSQLG  89.  DSSSASGQQQESSVLSQSSQASTSSQLG  90.  DSSSASGQQQESSVLSQSDQASTSSQLG  91.  DSSSASGQQQESSVLSQSDQASTSSQL | 4f |
| 26 | MF771032.1 | Colombia |  | >MF771032.1 Anaplasma marginale isolate 1103 major surface protein 1a (msp1a) gene, partial cds  CCATATACTGTGCAGTACGGTTGTGCTCCCCAATTGTTAAAATTTAGTATATTAATCTTGCGATTACCCG  TTCCGTATGTTACAATCGGGCTGCCGGTGTGGTAGCGTGCTGGTTGTGTGGTTGTCCTCTTTCCCGATGT  TGGGTCGTTCGTTTTACGTCTCACAAGTTTGTACGCTGTGCCCCTGGCAGTGTAGGGTTTGTTTGTGTGT  GTGTGTGTTATGTCAGAGTATGTGTCCCCCCAGCCAGGAATGAGCTCGTCAGCGAGTGGTCAGCAGCAAG  GGAGTGGTGTGTCATATCAAAGTGGTCAGGCCAGTACATCGATCAATTTGGAAACTGATAGCTCGTCAGC  GGGTGGTCAGCAGCAAGAGAGTAGTGTGTTATCTCAAAGTGGTCAGGCCAGTACATCGTCTCAATTAGGA  GCTGATAGCTCGTCAGCGGGTGATCAGCAGCAAGAGAGTAGTGTGTCATCTCAAAGTGGTCAGGCCAGTA  CATCGTCTCAATTAGGAACTGATAGCTCGTCAGCGAGTGGTCAGCAGCAAGAGAGTAGTGTGTTATCTCA  AAGTGGTCAGGCCAGTACATCGTCTCAATTAGGGAATGATTGGCGGCAAGAGATGCGCTCCAAGGTTGCG  AGTGTTGAGTACATTTTGGCTGCTCGTGCCCTTATCTCTGTAGGGGTCTATGCTGCTCAGGAAGAGATCG  CGAAATCGCTAGGGTATGCTCCCCTGCGTGTTGCAGAAGTCGAAGAAATCGTGAGGGATAGCCTTGTACG  CAGCCACTTTCATGATAGTGGCCTTTCACTAGGCTCCATACGACTCGTGCTTATGCAGGTTGGGGATAAG  TTGGGGCTACAAGGTTCGAAGATTAGCGAAGGGTACTCCACCTATCTCGCGCAAGCGTTTGCTGACAGCG  TGGTGGTTGCGGCTGATGTTCAGAGTGGTGGTGCGCGCTCTGCTACCAGCCTTGACAAGGCGATCGCAGA  CGTTGAGACGTCGTGGTCCCTGCGCGGCGACCTGATAAGCAAAGATTTTGACCGTGATACCAAAGTAGGA  AGGGACGACCTTGAGGCTTTTGTCGACTTCATGTTTGGCGGTGTGTCGTACAATGATGCGAACGCGGCTG  TGGCTAGGAGCGTATTGGAAACGCTTGCCGGGCACGTCGATGCACTTGGTATATCGTACAATCAGCTGGA  TAAGCTTGATGCTGACACTTTGTATAGTGTCGTATCGTTTAGTGGCGCTTCCGCAATAGACAGAGGTGCG  GTTAGCGATGCGGCTGACAAGTTCCGTGTGATGATGTTTGGTGGTGCTCCTGCGGGGCAAGAGAAAACTG  CCGAACCTGAGCATGAGGCTGCGACCCCGTCAGCTAGTAGCGTTCCGTCAACTGTGCATGGTAAGGTCGT  TGATCATGCAGTTGACCGTGCAAAAGAAGCGGCTAAGCAGGCCTATGCAGGCGTGCGTAAGTGGTATGTG  GCGAAGCCTTCGGACACTACTACACAGCTTGTTGTAGCTATCACGGCGCTGCTTATCACGGCGTTTGCTA  TCTGTGCGTGTTTGGAACCTAGGCTTATAGGGGCGTCCGGTCCGCTGATTTGGGGCTGCCTGGCACTAGT  AGCACTGCTGCCATTACTTGGTATGGCTGTGCATACGGCAGTGAGTGCTTCGAGTCAAAAGAAGGCTGCC  GGTGGTGCGCAACGGGTTGCTGCTCAGGAGAGGTCTAGGGAATTGTCCCGTGCGAGACAGGAAGATCAGC  AGAAGTTGCATGTTCCCGCGATACTGACCGGGTTGAGCGTGCTTGTGTTTATTGCTGCCGTCGTGGCTTG  TATTGCTGTTGACGCGAGGCGCGGGACGTGGCAGGGCAGCATATGTTTCCTAGCCGCATTTGTGTTGTTT  GCGATCAGTGCCGCTGTTGTAATGGCAACACGTGACCAATCGTTGGCAGAAGAGTGTGATAGCAAGTGTG  CTACAGCTCGTACGGCTCAAGCTGTACCCGGTGGCCAGCAGCAGCCGCGTGCTACCGAGGGCGTTGTTAG  CGGTGGCAGCCAAGAAGGCGGGGCTGGTGTTCCCGGAACTTCCGTGCCGTCAGCCGGGTCTGGGTCCGTA  CCTCCTGCTACCATTATGGTCAGTGTGGATCCACAACTTGTTGCTACTTTGGGAGCAGGTGTGGCGCAGG  CGGCGGCG | AGCTCGTCAGCGAGTGGTCAGCAGCAAGGGAGTGGTGTGTCATATCAAAGTGGTCAGGCCAGTACATCG-ATCAATTTGGAAACTGAT  AGCTCGTCAGCGGGTGGTCAGCAGCAAGAGAGTAGTGTGTTATCTCAAAGTGGTCAGGCCAGTACATCGTCTCAATTAGGAGCTGAT  AGCTCGTCAGCGGGTGATCAGCAGCAAGAGAGTAGTGTGTCATCTCAAAGTGGTCAGGCCAGTACATCGTCTCAATTAGGAACTGAT  AGCTCGTCAGCGAGTGGTCAGCAGCAAGAGAGTAGTGTGTTATCTCAAAGTGGTCAGGCCAGTACATCGTCTCAATTAGG | 92.  SSSASGQQQGSGVSYQSGQASTSINLETD  93.  SSSAGGQQQESSVLSQSGQASTSSQLGAD  94.  SSSAGDQQQESSVSSQSGQASTSSQLGTD  95.  SSSASGQQQESSVLSQSGQASTSSQL | 4 |
| 27 | MF771031.1 | Colombia |  | >MF771031.1 Anaplasma marginale isolate 1100 major surface protein 1a (msp1a) gene, partial cds  CCATATACTGTGCAGTACGGTTGTGCTCCCCAATTGTTAAAATTTAGTATATTAATCTTGCGATTACACG  TTCCGTATGTTACAATCGGGCTGCCGGTGTGGTAGCGTGCTGGTTGTGTGGTTGTCCTCTTTCCCGATGT  TGGGTCGTTCGTTTTACGTCTCACAAGTTTGTACGCTGTGCCCCTGGCAGTGTAGGGTTTGTTTGTGTGT  GTGTGTGTTATGTCAGAGTTGGTGTCCAGCCAGCCAGCTGATAGCTCGTCAGCGGGTGATCAGCAGCAAG  AGAGTAGTGTGTTATCTCAAAGTGGTCAGGCCAGTAGTACATCGTCTCAATTAGGAGCTGATAGCTCGTC  AGCGAGTGATCAGCAGCAAGAGAGAGGTGTGTTATCTCAAAGTGGTCAGGCCAGTACATCGTCTCAATTA  GGAACTGATAGCTCGTCAGCGAGTGGTCAGCAGCAAGAGAGTAGTGTGTCATCTCAAAGTGGTCAGGCCA  GTACATCGTCTCAATTAGGAACTGATTGGCGGCAAGAGATGCACTCCAAGGTTGCGAGTGTTGAGTACAT  TTTGGCTGCTCGTGCCCTTATCTCTGTAGGGGTCTATGCTGCTCAGAGAATGATCGCGCAATCGCGAGGG  TGTGCTCCCCTGAGTGTTGCAGAAGTCGAAGACATCGTGAGGGATAGCCTTGTACGCAGCCACTTTCATG  ATAGTGGCCTTTCACTAGGCTCCATACGACTCGTGCTTATGCAGGTTGGGGATAAGTTGGGGCTACAAGA  TTTGAAGATTGGCGAAGGGTACGCCACCTATCTCGCGCAAGCGTTTGCTGACAGCGTGGTGGTTGCGGCT  GATGTTCAGAGTGGTAGTGGGCGCTATGCTGCCGGCCTTGACAAGGCGATCGCAGACGTTGAGACGTCGT  GGTCCCTGCACGGCGGCCTGATAAGCAAAGATTTTGACCGTGGTACCAAAGTAGGAAGGGGCGACCTTGA  GGCTTTTGTCGACTTCATGTTTGGCGGTGTGTCGTACAATGATGTGAACGCGGCTGTGGCTAGGAGCGTA  TTGGAAACGCTTGCCGGGCATGTCGATGCACTTGGTATATCGTACAATCAGCTGGATAAGCTTGATGCTG  ACACTTTGTATAGTGTCGTATCGTTTAGTGGCGCTTCCGCAATAGACAGAGGTGCGGTTAGCGATGCGGC  TGACAAGTTCCGTGTGATGATGTTTGGTGGTGCTCCTGCGGGGCAAGAGAAAACTGCCGAACCTGAGCAT  GAGGCTGCGACCCCGTCAGCTAGTAGCGTTCCGTCAACTGTGCATGGTAAGGTCGTTGATCATGCAGTTG  ACCGTGCAAAAGAAGCGGCTAAGCAGGCCTATGCAGGCGTGCGTAAGTGGTATGTGGCGAAGCCTTCGGA  CACTACTACACAGCTTGTTGTAGCTATCACGGCGCTGCTTATCACGGCGTTTGCTATCTGTGCGTGTTTG  GAACCTAGGCTTATAGGGGCGTCCGGTCCGCTGATTTGGGGCTGCCTGGCACTAGTAGCACTGCTGCCAT  TACTTGGTATGGCTGTGCATACGGCAGTGAGTGCTTCGAGTCAAAAGAAGGCTGCCGGTGGTGCGCAACG  GGTTGCTGCTCAGGAGAGGTCTAGGGAATTGTCCCGTGCGAGACAGGAAGATCAGCAGAAGTTGCATGTT  CCCGCGATACTGACCGGGTTGAGCGTGCTTGTGTTTATTGCTGCCGTCGTGGCTTGTATTGCTGTTGACG  CGAAGCGCGGGACGTGGCAGGGCAGCATATGTTTCCTAGCCGCATTTGTGTTGTTTGCGATCAGTGCCGC  TGTTGTAATGGCAACACGTGACCAATCGTTGGCAGAAGAGTGTGATAGCAAGTGTGCTACAGCTCGTACG  GCTCAAGCTGTACCCGGTGGCCAGCAGCAGCCGCGTGCTACCGAGGGCGTTGTTAGCGGTGGCAGCCAAG  AAGGCGGGGCTGGTGTTCCCGGAACTTCCGTGCCGTCAGCCGGGTCTGGGTCCGTACCTCCTGCTACCAT  TATGGTCAGTGTGGATCCACAACTTGTTGCTACTTTGGGAGCAGGTGTGGCGCAGGCGGCGGCG | CTGATAGCTCGTCAGCGGGTGATCAGCAGCAAGAGAGTAGTGTGTTATCTCAAAGTGGTCAGGCCAGTAGTACATCGTCTCAATTAGGAG    CTGATAGCTCGTCAGCGAGTGATCAGCAGCAAGAGAG-AGGTGTGTTATCTCAAAGTGGTCAGGCCAGTACATCGTCTCAATTAGGAA  CTGATAGCTCGTCAGCGAGTGGTCAGCAGCAAGAGAGTAGTGTGTCATCTCAAAGTGGTCAGGCCAGTACATCGTCTCAATTAGGAA | 96.  DSSSAGDQQQESSVLSQSGQASSTSSQLG  97.  DSSSASDQQQERGVLSQSGQASTSSQLG  98.  DSSSASGQQQESSVSSQSGQASTSSQLG | 3 |
| 28 | MK809370.1 | Cuba | Western Region | >MK809370.1 Anaplasma marginale strain Oriente CuBov140 major surface protein 1a (msp1a) gene, partial cds  TGTGCTTATGGCAGACATTTCCATATACTGTGCAGTACGGTTGTGCTCCCCAATTGTTAAAATTTAGTAT  ATTAATCTTGCGATTACACGTTCCGTATGTTACAATCAGGCCGTCGGTGTGGTAGCGTGCTGGTTGTGTG  GTTGTCCTCTTTCCCGATGTTGGGTCGTTCGTTTTACGTCTCACAAGTTTGTACGCTGTGCCCCTGGCAG  TGTAGGGTTTGTTTGTGTGTGTGTTATGTCAGAGTATGTGTCCAGCCAGTCAGCTGATAGCTCGTCAGCG  AGTGGTCAGCAGCAAGAGAGTAGTGTGTCATCTCAAAGTGGTCAGGCCAGTACATCGTCTCAATTAGGGA  CTGATAGCTCGTCAGCGAGTGGTCAGCAGCAAGAGAGTAGTGTGTCATCTCAAAGTGGTCAGGCCAGTAC  ATCGTCTCAATTAGGGACTGATAGCTCGTCAGCGAGTGGTCAGCAGCAAGAGAGTAGTGTGTCATCTCAA  AGTGATCAGGCCAGTACATCGTCTCAATTAGGGACTGATTGGCGGCAAGAGATGCGCTCCAAGGTTGCGA  GTGTTGAGTACATGTTGGCTGCTCGTGCCCTTATCTCTGTAGGGGTCTATGCTGCTCAGAGAATGATCGC  GCAATCGCAAGGGTGTGCTTCCCTGAGTGTTGCAGAAGTCGAAGGCATCGTGAAGGATAGCCTTGTACGC  AGCCACTTTCATGATAGTGGCCTTTCACTAGGCTCCATACGACTCGTGCTTATGCAGGTCGGGGATAAGT  TGGGGCTACAAGGTTT | CTGATAGCTCGTCAGCGAGTGGTCAGCAGCAAGAGAGTAGTGTGTCATCTCAAAGTGGTCAGGCCAGTACATCGTCTCAATTAGGGA  CTGATAGCTCGTCAGCGAGTGGTCAGCAGCAAGAGAGTAGTGTGTCATCTCAAAGTGGTCAGGCCAGTACATCGTCTCAATTAGGGA  CTGATAGCTCGTCAGCGAGTGGTCAGCAGCAAGAGAGTAGTGTGTCATCTCAAAGTGATCAGGCCAGTACATCGTCTCAATTAGGGA  CTGAT | 99.  DSSSASGQQQESSVSSQSGQASTSSQLG  100.  DSSSASGQQQESSVSSQSGQASTSSQLG  101.  DSSSASGQQQESSVSSQSDQASTSSQLG  102.  D | 3 |
| 29 | MK809371.1 | Cuba | Western Region | >MK809371.1 Anaplasma marginale strain CuBov09 major surface protein 1a (msp1a) gene, partial cds  TGTGCTTATGGCAGACATTTCCATATACTGTGCAGTACGGTTGTGCTCCCCAATTGTTAAAATTTAGTAT  ATTAATCTTGCGATTACACGTTCCGTATGTTACAATCAGGCCGCCGGTGTGGTAGCGTGCTGGTTGTGTG  GTTGTCCTCTTTCCCGATGTTGGGTCGTTCGTTTTACGTCGCACAAGTTTGTACGCTGTGCCCCTGGCAG  TGTAGGGTTTATTTGTTTGTGTGTGTGTTATGTCAGCAGAGTATGTGTCCTCCCAGCCAGCTGGTAGCTC  GTCAGCGGGTGGTCAGCAGCAAGAGAGTAGTGTGTCATCTCAAAGTGATCAGGCCAGTACATCGTCTCAA  TTAGGAGCTGGTAGCTCGTCAGCGGGTGGTCAGCAGCAAGAGAGTAGTGTGTCATCTCAAAGTGATCAGG  CCAGTACATCGTCTCAATTAGGAGCTGGTAGCTCGTCAGCGGGTGGTCAGCAGCAAGAGAGTAGTGTGTC  ATCTCAAAGTGATCAGGCCAGTACATCGTCTCAATTAGGAGCTGGTAGCTCGTCAGCGGGTGGTCAGCAG  CAAGAGAGTAGTGTGTCATCTCAAAGTGATCAGGCCAGTACATCGTCTCAATTAGGGACTGATTGGCGGC  AAGAGATGCGCTCCAAGGTTGCGAGTGTTGAGTACATGTTGGCTGCTCGTGCCCTTATTTCTGTAGGGGT  CTATGCTGCTCAGGGAGAGATCGCGAGATCGCGAGGGTGTGCTCCCCTGCGTGTTGCAGAAGTCGAAGAA  ATCGTGAGGGATGGCCTTGTACGCAGCCACTTTCATGATAGTGGCCTTTCACTAGGCTCCATACGACTCG  TGCTTATGCAGGTTGGGGATAAGTTGGGGCTACAAGGTTTGAAGATTGGCGAAAGGTACGCCACCTATCT  CGCGCAAGCGTTTGCTGACAGCGTGGTGGTTGCGGCTGATGTTCAGAGTAGTGGTGCGTGCTCTGCCGGC  CTTGACAGCGCGATCGCAAACGTTGAGAAGTCGTGGTCCCTGCACGGCGGCCTGGTAAGCAAAGGTTTTG  ACCGTGA | AGCTGGTAGCTCGTCAGCGGGTGGTCAGCAGCAAGAGAGTAGTGTGTCATCTCAAAGTGATCAGGCCAGTACATCGTCTCAATTAGG  AGCTGGTAGCTCGTCAGCGGGTGGTCAGCAGCAAGAGAGTAGTGTGTCATCTCAAAGTGATCAGGCCAGTACATCGTCTCAATTAGG  AGCTGGTAGCTCGTCAGCGGGTGGTCAGCAGCAAGAGAGTAGTGTGTCATCTCAAAGTGATCAGGCCAGTACATCGTCTCAATTAGG  AGCTGGTAGCTCGTCAGCGGGTGGTCAGCAGCAAGAGAGTAGTGTGTCATCTCAAAGTGATCAGGCCAGTACATCGTCTCAATTAGG | 103.  AGSSSAGGQQQESSVSSQSDQASTSSQL  104.  AGSSSAGGQQQESSVSSQSDQASTSSQL  105.  AGSSSAGGQQQESSVSSQSDQASTSSQL  106.  AGSSSAGGQQQESSVSSQSDQASTSSQL | 4 |
| 30 | MK809372.1 | Cuba | Western Region | >MK809372.1 Anaplasma marginale strain CuBov56 major surface protein 1a (msp1a) gene, partial cds  TGTGCTTATGGCAGACATTTCCATATACTGTGCAGTATGGTTGTGCTCCCCAATTGTTAAAATTTAGTAT  ATTAATCTTGCGATTACACGTTCCGTATGTTACAATCAGGCCGCCGGTGTGGTAGCGTGCTGGTTGTGTG  GTTGTCCTTTTTCCCGATGTTGGGTCGTTCGTTTTACGTCGCACAAGTTTGTACGCTGTGCCCCTGGCAG  TGTAGGGTTTATTTGTTTGTGTGTGTGTTATGTCAGCAGAGTATGTGTCCTCCCAGCCAGCTGGTAGCTC  GTCAGCGGGTGGTCAGCAGCAAGAGAGTAGTGTGTCATCTCAAAGTGATCAGGCCAGTACATCGTCTCAA  TTAGGAGCTGATAGCTCGTCAGCGGGTGGTCAGCAGCAAGAGAGCAGTGTGTCATCTCAAAGTGATCAGG  CCAGTACATCGTCTCAATTAGGAGCTGATAGCTCGTCAGCGGGTGGTCAGCAGCAAGAGAGTAGTGTGTC  ATCTCAAAGTGATCAGGCCAGTACATCGTCTCAATTAGGAGCTGATAGCTCGTCAGCGGGTGGTCAGCAG  CAAGAGAGTAGTGTGTCATCTCAAAGTGATCAGGCCAGTACATCGTCTCAATTAGGGACTGATTGGCGGC  AAGAGATGCGCTCCAAGGTTGCGAGTGTTGAGTACATGTTGGCTGCTCGTGCCCTTATTTCTGTAGGGGT  CTATGCTGCTCAGGGAGAGATCGCGAGATCGCGAGGGTGTGCTCCCCTGCGTGTTGCAGAAGTCGAAGAA  ATCGTGAGGGATGGCCTTGTACGCAGCCACTTTCATGATAGTGGCCTTTCACTAGGCTCCATACGACTCG  TGCTTATGCAGGTTGGGGATAAGTTGGGGCTACAAGGTTTGAAGATTGGCGAAAGGTACGCCACCTATCT  CGCGCAAGCGTTTGCTGACAGCGTGGTGGTTGCGGCTGATGTTCAGAGTAGTGGTGCGTGCTCTGCCGGC  CTTGGCAGCGCGATCGCAAACGTTGAGAAGTCGTGGTCCCTGCACGGCGGCCTGGTAAGCAAAGGTTTTG  ACCGTGA | AGCTGGTAGCTCGTCAGCGGGTGGTCAGCAGCAAGAGAGTAGTGTGTCATCTCAAAGTGATCAGGCCAGTACATCGTCTCAATTAGG  AGCTGATAGCTCGTCAGCGGGTGGTCAGCAGCAAGAGAGCAGTGTGTCATCTCAAAGTGATCAGGCCAGTACATCGTCTCAATTAGG  AGCTGATAGCTCGTCAGCGGGTGGTCAGCAGCAAGAGAGTAGTGTGTCATCTCAAAGTGATCAGGCCAGTACATCGTCTCAATTAGG  AGCTGATAGCTCGTCAGCGGGTGGTCAGCAGCAAGAGAGTAGTGTGTCATCTCAAAGTGATCAGGCCAGTACATCGTCTCAATTAGG | 107.  AGSSSAGGQQQESSVSSQSDQASTSSQL  108.  ADSSSAGGQQQESSVSSQSDQASTSSQL  109.  ADSSSAGGQQQESSVSSQSDQASTSSQL  110.  ADSSSAGGQQQESSVSSQSDQASTSSQL | 4 |
| 31 | MK809373.1 | Cuba | Western Region | >MK809373.1 Anaplasma marginale strain CuBov66 major surface protein 1a (msp1a) gene, partial cds  TGTGCTTATGGCAGACATTTCCATATACTGTGCAGTATGGTTGTGCTCCCCAATTGTTAAAATTTAGTAT  ATTAATCTTGCGATTACACGTTCCGTATGTTACAATCAGGCTGCCGGTGTGGTAGCGTGCTGGTTGTGTG  GTTGTCCTCTTTCCCGATGTTGGGTCGTTCGTTTTACGTCGCACAAGTTTGTACGCTGTGCCCCTGGCAG  TGTAGGGTTTATTTGTTTGTGTGTGTGTTATGTCAGCAGAGTATGTGTCCTCCCAGCCAGCTGATAGCTC  GTCAGCGGGTGGTCAGCAGCAAGAGAGTAGTGTGTCATCTCAAAGTGATCAGGCCAGTACATCGTCTCAA  TTAGGAGCTGATAGCTCGTCAGCGGGTGGTCAGCAGCAAGAGAGTAGTGTGTCATCTCAAAGTGATCAGG  CCAGTACATCGTCTCAATTAGGAGCTGATAGCTCGTCAGCGGGTGGTCAGCAGCAAGAGAGTAGTGTGTC  ATCTCAAAGTGATCAGGCCAGTACATCGTCTCAATTAGGAGCTGATAGCTCGTCAGCGAGTGGTCAGCAG  CAAGAGAGTAGTGTGTTATCTCCAAGTGGTCAGGCCAGTACATCGTCTCAATTAGGAGTAGGAGCTGATA  GCTCGTCAGCGAGTGGTCAGCAGCAAGAGAGTAGTGTGTCATCTCAAAGTGATGCCAGTACATCGTCTCA  ATCAGGGACTGATTGGCGGCAAGAGATGCGCTCCAAGGTTGCGAGTGTTGAGTACATGTTGGCTGCTCGT  GCCCTTATTTCTGTAGGGGTCTATGCTGCTCAGGGAGAGATCGCGAGATCGCGAGGGTGTGCTCCCCTGC  GTGTTGCAGAAGTCGAAGGAATCGTGAGGGATAGCCTTGTACGCAGCCACTTTCATGATAGTGGCCTTTC  ACTAGGCTCCATACGACTCGTGCTTATGCAGGTTGGGGATAAGTTGGGGCTACAAGATTCGAAGATTGGC  GAAGGGTACGCCACCTATCTCGCGCAAGCGTTTGCTGACAGCGTGGTGGTTGCGGCTGATGTTCAGAGTA  GTGATGCGTGCCCTGCCAGCCTTGACAGCGCGATCGCAAACGTTGAGAAGTCGTGGTCCCTGCACGGCGG  CCTGGTAAGCAAAGGTTTTGACCGTGA | AGCTGATAGCTCGTCAGCGGGTGGTCAGCAGCAAGAGAGTAGTGTGTCATCTCAAAGTGATCAGGCCAGTACATCGTCTCAATTAGG  AGCTGATAGCTCGTCAGCGGGTGGTCAGCAGCAAGAGAGTAGTGTGTCATCTCAAAGTGATCAGGCCAGTACATCGTCTCAATTAGG  AGCTGATAGCTCGTCAGCGGGTGGTCAGCAGCAAGAGAGTAGTGTGTCATCTCAAAGTGATCAGGCCAGTACATCGTCTCAATTAGG  AGCTGATAGCTCGTCAGCGAGTGGTCAGCAGCAAGAGAGTAGTGTGTTATCTCCAAGTGGTCAGGCCAGTACATCGTCTCAATTAGGAGTAGG  AGCTGATAGCTCGTCAGCGAGTGGTCAGCAGCAAGAGAGTAGTGTGTCATCTCAAAGTGAT---GCCAGTACATCGTCTCAAT | 111.  ADSSSAGGQQQESSVSSQSDQASTSSQL  112.  ADSSSAGGQQQESSVSSQSDQASTSSQL  113.  ADSSSAGGQQQESSVSSQSDQASTSSQL  114.  ADSSSASGQQQESSVLSPSGQASTSSQLGV  115.  ADSSSASGQQQESSVSSQSDASTSSQ | 5 |
| 32 | MK809374.1 | Cuba | Western Region | >MK809374.1 Anaplasma marginale strain CuBov73 major surface protein 1a (msp1a) gene, partial cds  TGTGCTTATGGCAGACATTTCCATATACTGTGCAGTATGGTTGTGCTCCCCAATTGTTAAAATTTAGTAT  ATTAATCTTGCGATTACACGTTCCGTATGTTACAATCAGGCCGCCGGTGTGGTAGCGTGCTGGTTGTGTG  GTTGTCCTCTTTCCCGATGTTGGGTCGTTCGTTTTACGTCGCACAAGTTTGTACGCTGTGCCCCTGGCAG  TGTAGGGTTTATTTGTTTGTGTGTGTGTTATGTCAGCAGAGTATGTGTCCTCCCAGCCAGCTGGTAGCTC  GTCAGCGGGTGGTCAGCAGCAAGAGAGTAGTGTGTCATCTCAAAGTGATCAGGCCAGTACATCGTCTCAA  TTAGGAGCTGATAGCTCGTCAGCGGGTGGTCAGCAGCAAGAGAGTAGTGTGTCATCTCAAAGTGATCAGG  CCAGTACATCGTCTCAATTAGGAGCTGATAGCTCGTCAGCGGGTGGTCAGCAGCAAGAGAGTAGTGTGTC  ATCTCAAAGTGGTCAGGCCAGTACATCGTCTCAATTAGGGACTGATTGGCGGCAAGAGATGCGCTCCAAG  GTTGCGAGTGTTGAGTACATGTTGGCTGCTCGTGCCCTTATTTCTGTAGGGGTCTATGCTGCTCAGGGAG  AGATCGCGAGATCGCGAGGGTGTGCTCCCCTGCGTGTTGCAGAAGTCGAAGAAATCGTGAGGGATGGCCT  TGTACGCAGCCACTTTCATGATAGTGGCCTTTCACTAGGCTCCATACGACTCGTGCTTATGCAGGTTGGG  GATAAGTTGGGGCTACAAGGTTTGAAGATTGGCGAAGGGTACGCCACCTATCTCGCGCAAGCGTTTGCTG  ACAGCGTGGTGGTTGCGGCTGATGTTCAGAGTAGTGGTGCGTGCCCTGCCAGCCTTGACAGCGCGATCGC  AAACGTTGAGACGTCGTGGTCCCTGCACGGCGGCCTGGTAAGCAAAGGTTTTGACCGTGA | AGCTGGTAGCTCGTCAGCGGGTGGTCAGCAGCAAGAGAGTAGTGTGTCATCTCAAAGTGATCAGGCCAGTACATCGTCTCAATTAGG  AGCTGATAGCTCGTCAGCGGGTGGTCAGCAGCAAGAGAGTAGTGTGTCATCTCAAAGTGATCAGGCCAGTACATCGTCTCAATTAGG  AGCTGATAGCTCGTCAGCGGGTGGTCAGCAGCAAGAGAGTAGTGTGTCATCTCAAAGTGGTCAGGCCAGTACATCGTCTCAATTAGG | 116.  AGSSSAGGQQQESSVSSQSDQASTSSQL  117.  ADSSSAGGQQQESSVSSQSDQASTSSQL  118.  ADSSSAGGQQQESSVSSQSGQASTSSQL | 3 |
| 33 | MK809375.1 | Cuba | Western Region | >MK809375.1 Anaplasma marginale strain CuBov96 major surface protein 1a (msp1a) gene, partial cds  TGTGCTTATGGCAGACATTTCCATATACTGTGCAGTATGGTTGTGCTCCCCAATTGTTAAAATTTAGTAT  ATTAATCTTGCGATTACACGTTCCGTATGTTACAATCAGGCCGCCGGTGTGGTAGCGTGCTGGTTGTGTG  GTTGTCCTCTTTCCCGATGTTGGGTCGTTCGTTTTACGTCGCACAAGTTTGTACGCTGTGCCCCTGGCAG  TGTAGGGTTTATTTGTTTGTGTGTGTGTTATGTCAGCAGAGTATGTGTCCCCCCAGCCAGCTGGTAGCTC  GTCAGCGGGTGGTCAGCAGCAAGAGAGTAGTGTGTCATCTCAAAGTGATCAGGCCAGTACATCGTCTCAA  TTAGGAGCTGATAGCTCGTCAGCGGGTGGTCAGCAGCAAGAGAGTAGTGTGTCATCTCAAAGTGATCAGG  CCAGTACATCGTCTCAATTAGGAGCTGATAGCTCGTCAGCGGGTGGTCAGCAGCAAGAGAGTAGTGTGTC  ATCTCAAAGTGATCAGGCCAGTACATCGTCTCAATCAGGAGCTAATTGGCGGCAAGAGATGCGCTCCAAG  GTTGCGAGTGTTGAGTACATTTTGGCTGCTCGTGCCCTTATTTCTGTAGGGGTCTATGCTGCTCAGGGAG  AGATCGCGAAATCGCAAGGGTGTGCTCCCCTGCGTGTTGCAGAAGTCGAAGAAATCGTGAGGGATGGCCT  TGTACGCAGCCACTTTCATGATAGTGGCCTTTCACTAGGCTCCATACGACTCGTGCTTATGCAGGTTGGG  GATAAGTTGGGGCTACAAGGTTTGAAGATTGGCGAAGGGTACGCCACCTATCTCGCGCAAGCGTTTGCTG  ACAGCGTGGTGGTTGCGGCTGATGTTCAGAGTAGTGGTGCGTGCCCTGCCAGCCTTGACAGCGCGATCGC  AAACGTTGAGACGTCGTGGTCCCTGCACGGCGGCCTGGTAAGCAAAGGTTTTGACCGTGA | AGCTGGTAGCTCGTCAGCGGGTGGTCAGCAGCAAGAGAGTAGTGTGTCATCTCAAAGTGATCAGGCCAGTACATCGTCTCAATTAGG  AGCTGATAGCTCGTCAGCGGGTGGTCAGCAGCAAGAGAGTAGTGTGTCATCTCAAAGTGATCAGGCCAGTACATCGTCTCAATTAGG  AGCTGATAGCTCGTCAGCGGGTGGTCAGCAGCAAGAGAGTAGTGTGTCATCTCAAAGTGATCAGGCCAGTACATCGTCTCAATCAGG | 119.  AGSSSAGGQQQESSVSSQSDQASTSSQL  120.  ADSSSAGGQQQESSVSSQSDQASTSSQL  121.  ADSSSAGGQQQESSVSSQSDQASTSSQS | 3 |
| 34 | MK809376.1 | Cuba | Western Region | >MK809376.1 Anaplasma marginale strain CuBov100 major surface protein 1a (msp1a) gene, partial cds  TGTGCTTATGGCAGACATTTCCATATACTGTGCAGTATGGTTGTGCTCCCCAATTGTTAAAATTTAGTAT  ATTAATCTTGCGATTACACGTTCCGTATGTTACAATCAGGCCGCCGGTGTGGTAGCGTGCTGGTTGTGTG  GCTGTCCTCTTTCCCGATGTTGGGTCGTTCGTTTTACGTCGCACAAGTTTGTACGCTGTGCCCCTGGCAG  TGTAGGGTTTATTTGTTTGTGTGTGTGTTATGTCAGCAGAGTATGTGTCCTCCCAGCCAGCTGGTAGCTC  GTCAGCGGGTGGTCAGCAGCAAGAGAGTAGTGTGTCATCTCAAAGTGATCAGGCCAGTACATCGTCTCAA  TTAGGAGCTGATAGCTCGTCAGCGGGTGGTCAGCAGCAAGAGAGTAGTGTGTCATCTCAAAGTGATCAGG  CCAGTACATCGTCTCAATTAGGAGCTGATAGCTCGTCAGCGGGTGGTCAGCAGCAAGAGAGTAGTGTGTC  ATCTCAAAGTGATCAGGCCAGTACATCGTCTCAATTAGGAGCTGATAGCTCGTCAGCGGGTGGTCAGCAG  CAAGAGAGTAGTGTGTCATCTCAAAGTGATCAGGCCAGTACATCGTCTCAATTAGGAGCTGATAGCTCGT  CAGCGAGTGGTCAGCAGCAAGAGAGTAGTGTGTCATCTCAAAGTGGTCAGGCCAGTACATCGTCTCAATT  AGGGACTGATTGGCGGCAAGAGATGCGCTCCAAGGTTGCGAGTGTTGAGTACATGTTGGCTGCTCGTGCC  CTTATTTCTGTAGGGGTCTATGCTGCTCAGGGAGAGATCGCGAGATCGCGAGGGTGTGCTCCCCTGCGTG  TTGCAGAAGTCGAAGAAATCGTGAGGGATGGCCTTGTACGCAGCCACTTTCATGATAGTGGCCTTTCACT  AGGCTCCATACGACTCGTGCTTATGCAGGTTGGGGATAAGTTGGGGCTACAAGGTTTGAAGATTGGCGAA  AGGTACGCCACCTATCTCGCGCAAGCGTTTGCTGACAGCGTGGTGGTTGCGGCTGATGTTCAGAGTAGTG  GTGCGTGCCCTGCCAGCCTTGACAGCGCGATCGCAAACGTTGAGACGTCGTGGTCCCTGCACGGCGGCCT  GGTAAGCAAAGGTTTTGACCGTGA | AGCTGGTAGCTCGTCAGCGGGTGGTCAGCAGCAAGAGAGTAGTGTGTCATCTCAAAGTGATCAGGCCAGTACATCGTCTCAATTAGG  AGCTGATAGCTCGTCAGCGGGTGGTCAGCAGCAAGAGAGTAGTGTGTCATCTCAAAGTGATCAGGCCAGTACATCGTCTCAATTAGG  AGCTGATAGCTCGTCAGCGGGTGGTCAGCAGCAAGAGAGTAGTGTGTCATCTCAAAGTGATCAGGCCAGTACATCGTCTCAATTAGG  AGCTGATAGCTCGTCAGCGGGTGGTCAGCAGCAAGAGAGTAGTGTGTCATCTCAAAGTGATCAGGCCAGTACATCGTCTCAATTAGG  AGCTGATAGCTCGTCAGCGAGTGGTCAGCAGCAAGAGAGTAGTGTGTCATCTCAAAGTGGTCAGGCCAGTACATCGTCTCAATTAGG | 122.  AGSSSAGGQQQESSVSSQSDQASTSSQL  123.  ADSSSAGGQQQESSVSSQSDQASTSSQL  124.  ADSSSAGGQQQESSVSSQSDQASTSSQL  125.  ADSSSAGGQQQESSVSSQSDQASTSSQL  126.  ADSSSASGQQQESSVSSQSGQASTSSQL | 5 |
| 35 | MK809377.1 | Cuba | Western Region | >MK809377.1 Anaplasma marginale strain CuBov132 major surface protein 1a (msp1a) gene, partial cds  TGTGCTTATGGCAGACATTTCCATATACTGTGCAGTACGGTTGTGCTCCCCAATTGTTAAAATTTAGTAT  ATTAATCTTGCGATTACACGTTCCGTATGTTACAATCGGGCTGCCGGTGTGGTAGCGTGCTGGTTGTGTG  GTTGTCCTCTTTCCCGATGTTGGGTCGTTCGTTTTACGTCTCACAAGTTTGTACGCTGTGCCCCTGGCAG  TGTAGGGTTTGTTTGTGTGTGTGTGTGTTATGTCAGAGTATGTGTCCAGCCAGCCAGCTGATAGCTCGTC  AGCGAGTGGTGTGTTATCTCAAAGTGGTCAGGCCAGTACATCGTCTCAATTAGGAACTGATAGCTCGTCA  GCGGGTGATCAGCAGCAAGGGAGTGGTGTGTCATCTCAAAGTGGTCAGGCCAGTACATCGTCTCAATTAG  GAACTGATAGCTCGTCAGCGGGTGATCAGCAGCAAGGGAGTGGTGTGTCATCTCAAAGTGGTCAGGCCAG  TACATCGTCTCAATTAGGGACTGATAGCTCGTCAGCGAGTGGTCAGCAGCAAGAGAGTAGTGTGTCATCT  CAAAGTGATGCCAGTACATCGTCTCAATTAGGAGCTGATTGGCGGCAAGAGGTGCACTCCAAGGTTGCGA  GTGTTGAGTACATTTTGGCTGCTCGTGCCCTTATCTCTGTAGGGGTCTATGCTGCTCAGAGAATGATCGC  GCAATCGCGAGGGTGTGCTTCCCTGAGTGTTGCAGAAGTCGAAGAAATCGTGAGGGATAGCCTTGTACGC  AGCCACTTTCATGGTAGTGGCCTTTCACTAGGCTCCATACGACTCGTGCTTATGCGGGTTGGGGATAAGT  TGGGGCTACAAGATTTGAAGATTGGCGAAGGGTACGCCACCTATCTCGCGCAAGCGTTTGCTGACAGCGT  GGTGGTTGCGGCTGATGTTCAGAGTGGTGGTGGGCGCTCTGCTGCCGGCCTTGACAAGGCGATCGCAGAC  GTTGAGACGTCGTGGTCCCTGCACGGCGGCCTGGTAAGCAAAGGTTTTGACCGTGA | GAGTGGTGTGTTATCTCAAAGTGGTCAGGCCAGTACATCGTCTCAATTAGGAACTGATAGCTCGTCAGCGGGTGATCAGCAGCAAGG  GAGTGGTGTGTCATCTCAAAGTGGTCAGGCCAGTACATCGTCTCAATTAGGAACTGATAGCTCGTCAGCGGGTGATCAGCAGCAAGG  GAGTGGTGTGTCATCTCAAAGTGGTCAGGCCAGTACATCGTCTCAATTAGGGACTGATAGCTCGTCAGCGAGTGGTCAGCAGCAAGA  GAGTAGTGTGTCATCTCAAAGT-G--ATGCCAGTACATCGTCTCAATTAGGAGCTGAT | 127.  SGVLSQSGQASTSSQLGTDSSSAGDQQQ  128.  SGVSSQSGQASTSSQLGTDSSSAGDQQQ  129.  SGVSSQSGQASTSSQLGTDSSSASGQQQ  130.  SSVSSQSDASTSSQLGAD | 4 |
| 36 | MK809378.1 | Cuba | Western Region | >MK809378.1 Anaplasma marginale strain CuBov135 major surface protein 1a (msp1a) gene, partial cds  TGTGCTTATGGCAGACATTTCCATATACTGCGCAGTATGGTTGTGCTCCCCAATTGTTAAAATTTAGTAT  ATTAATCTTGCGATCACACGTTCCGTATGTTACAATCAGGCCGCCGGTGTGGTAGCGTGCTGGTTGTGTG  GTTGTCCTCTTTCCCGATGTTGGGTCGTTCGTTTTACGTCGCACAAGTTTGTACGCTGTGCCCCTGGCAG  TGTAGGGTTTATTTGTTTGTGTGTGTGTTATGTCAGCAGAGTATGTGTCCTCCCAGCCAGCTGGTAGCTC  GTCAGCGGGTGGTCAGCAGCAAGAGAGTAGTGTGTCATCTCAAAGTGATCAGGCCAGTACATCGTCTCAA  TTAGGAGCTGATAGCTCGTCAGCGGGTGGTCAGCAGCAAGAGAGTAGTGTGTCATCTCAAAGTGATCAGG  CCAGTACATCGTCTCAATTAGGAGCTGATAGCTCGTCAGCGGGTGGTCAGCAGCAAGAGAGTAGTGTGTC  ATCTCAAAGTGGTCAGGCCAGTACATCGTCTCAATTAGGGACTGATAGCTCGTCAGCGAGTGGTCAGCAG  CAAGAGAGTAGTGTGTCATCTCAAAGTGGTCAGGCCAGTACATCGTCTCAATTAGGGACTGATTGGCGGC  AAGAGATGCGCTCCAAGGTTGCGAGTGTTGAGTACATGTTGGCTGCTCGTGCCCTTATTTCTGTAGGGGT  CTATGCTGCTCAGGGAGAGATCGCGAGATCGCGAGGGTGTGCTCCCCTGCGTGTTGCAGAAGTCGAAGAA  ATCGTGAGGGATGGCCTTGTACGCAGCCACTTTCATGATAGTGGCCTTTCACTAGGCTCCATACGACTCG  TGCTTATGCAGGTTGGGGATAAGTTGGGGCTACAAGGTTTGAAGATTGGCGAAGGGTACGCCACCTATCT  CGCGCAAGCGTTTGCTGACAGCGTGGTGGTTGCGGCTGATGTTCAGAGTAGTGATGCGTGCCCTGCCAGC  CTTGACAGCGCGATCGCAAACGTTGAGACGTCGTGGTCCCTGCACGGCGGCCTGGTAAGCAAAGGTTTTG  ACCGTGA | AGCTGGTAGCTCGTCAGCGGGTGGTCAGCAGCAAGAGAGTAGTGTGTCATCTCAAAGTGATCAGGCCAGTACATCGTCTCAATTAGG  AGCTGATAGCTCGTCAGCGGGTGGTCAGCAGCAAGAGAGTAGTGTGTCATCTCAAAGTGATCAGGCCAGTACATCGTCTCAATTAGG  AGCTGATAGCTCGTCAGCGGGTGGTCAGCAGCAAGAGAGTAGTGTGTCATCTCAAAGTGGTCAGGCCAGTACATCGTCTCAATTAGG  -GACTGATAGCTCGTCAGCGAGTGGTCAGCAGCAAGAGAGTAGTGTGTCATCTCAAAGTGGTCAGGCCAGTACATCGTCTCAATTAGG | 131.  AGSSSAGGQQQESSVSSQSDQASTSSQL  132.  ADSSSAGGQQQESSVSSQSDQASTSSQL  133.  ADSSSAGGQQQESSVSSQSGQASTSSQL  134.  TDSSSASGQQQESSVSSQSGQASTSSQL | 4 |
| 37 | MF288044.1 | Ecuador | Zamora-Chinchipe | >MF288044.1 Anaplasma marginale isolate Z25-Oeste major surface protein 1 alpha (msp1a) gene, partial cds AGGGTTTGTTTATTTGTGTGTGTGTTATGTCAGAGTATGGGTCCTCTCAGCCAGCTGATAGCTCGTCAGC  GAGTGGTCAGCAGCAAGAGAGTAGTGTGTTATCTCAAAGTGATCAGGCCAGTACATCGTCTCAATTAGGA  ACTGATAGCTCGTCAGCGAGTGGTCAGCAGCAAGAGAGTAGTGTGTTATCTCAAAGTGATCAGGCCAGTA  CATCGTCTCAATTAGGAACTGATAGCTCGTCAGCGAGTGGTCAGCAGCAAGAGAGTAGTGTGTTATCTCA  AAGTGATCAGGCCAGTACATCGTCTCAATCAGGAACTGATTGGCGGCAAGAGATGCGCTCCAAGGTTGCG  AGTGTTGAGTACATTTTGGCTGCTCGTGCCCTTATCTCTGTAGGGGTCTATGCTGCTCAGGAAGAGA | GAGTGGTCAGCAGCAAGAGAGTAGTGTGTTATCTCAAAGTGATCAGGCCAGTACATCGTCTCAATTAGGAACTGATAGCTCGTCAGC  GAGTGGTCAGCAGCAAGAGAGTAGTGTGTTATCTCAAAGTGATCAGGCCAGTACATCGTCTCAATTAGGAACTGATAGCTCGTCAGC  GAGTGGTCAGCAGCAAGAGAGTAGTGTGTTATCTCAAAGTGATCAGGCCAGTACATCGTCTCAATCAGGAACTGAT | 135.  SGQQQESSVLSQSDQASTSSQLGTDSSS  136.  SGQQQESSVLSQSDQASTSSQLGTDSSS  137.  SGQQQESSVLSQSDQASTSSQSGTD | 3 |
| 38 | MF288043.1 | Ecuador | Zamora-Chinchipe | >MF288043.1 Anaplasma marginale isolate Z19-Oeste major surface protein 1 alpha (msp1a) gene, partial cds GTGCCCCTGGCAGTGTAGGGTTTGTTTATTTGTGTGTGTGTTATGTCAGAGTATGGGTCCTCTCAGCCAG  CTGATAGCTCGTCAGCGAGTGGTCAGCAGCAAGAGAGTAGTGTGTTATCTCAAAGTGATCAGGCCAGTAC  ATCGTCTCAATTAGGAACTGATAGCTCGTCAGCGAGTGGTCAGCAGCAAGAGAGTAGTGTGTTATCTCAA  AGTGATCAGGCCAGTACATCGTCTCAATTAGGAACTGATAGCTCGTCAGCGAGTGGTCAGCAGCAAGAGA  GTAGTGTGTTATCTCAAAGTGATCAGGCCAGTACATCGTCTCAATCAGGAACTGATTGGCGGCAAGAGAT  GCGCTCCAAGGTTGCGAGTGTTGAGTACATTTTAGCTGCTCGTGCCCTTATCTCTGTAGGGGTCTATGCT  GCTCAGGAAGAGATCGCGAGATCGCTAGGG | CTGATAGCTCGTCAGCGAGTGGTCAGCAGCAAGAGAGTAGTGTGTTATCTCAAAGTGATCAGGCCAGTACATCGTCTCAATTAGGAA  CTGATAGCTCGTCAGCGAGTGGTCAGCAGCAAGAGAGTAGTGTGTTATCTCAAAGTGATCAGGCCAGTACATCGTCTCAATTAGGAA  CTGATAGCTCGTCAGCGAGTGGTCAGCAGCAAGAGAGTAGTGTGTTATCTCAAAGTGATCAGGCCAGTACATCGTCTCAATCAGGAA  CTGAT | 138.  DSSSASGQQQESSVLSQSDQASTSSQLG  139.  DSSSASGQQQESSVLSQSDQASTSSQLG  140.  DSSSASGQQQESSVLSQSDQASTSSQSG  141.  D | 3 |
| 39 | MF288042.1 | Ecuador | Zamora-Chinchipe | >MF288042.1 Anaplasma marginale isolate Z11-Oeste major surface protein 1 alpha (msp1a) gene, partial cds GTGCCCCTGGCAGTGTAGGGTTTGTTTATTTGTGTGTGTGTTATGTCAGAGTATGGGTCCTCTCAGCCAG  CTGATAGCTCGTCAGCGAGTGGTCAGCAGCAAGAGAGTAGTGTGTTATCTCAAAGTGATCAGGCCAGTAC  ATCGTCTCAATTAGGAACTGATAGCTCGTCAGCGAGTGGTCAGCAGCAAGAGAGTAGTGTGTTATCTCAA  AGTGATCAGGCCAGTACATCGTCTCAATTAGGAACTGATAGCTCGTCAGCGAGTGGTCAGCAGCAAGAGA  GTAGTGTGTTATCTCAAAGTGATCAGGCCAGTACATCGTCTCAATCAGGAACTGATTGGCGGCAAGAGAT  GCGCTCCAAGGTTGCGAGTGTTGAGTACATTTTGGCTGCTCGTGCCCTTATCTCTGTAGGGGTCTATGCT  GCTCAGGAAGAGATCGCGAGATCGCTAGGG | CTGATAGCTCGTCAGCGAGTGGTCAGCAGCAAGAGAGTAGTGTGTTATCTCAAAGTGATCAGGCCAGTACATCGTCTCAATTAGGAA  CTGATAGCTCGTCAGCGAGTGGTCAGCAGCAAGAGAGTAGTGTGTTATCTCAAAGTGATCAGGCCAGTACATCGTCTCAATTAGGAA  CTGATAGCTCGTCAGCGAGTGGTCAGCAGCAAGAGAGTAGTGTGTTATCTCAAAGTGATCAGGCCAGTACATCGTCTCAATCAGGAA  CTGAT | 142.  DSSSASGQQQESSVLSQSDQASTSSQLG  143.  DSSSASGQQQESSVLSQSDQASTSSQLG  144.  DSSSASGQQQESSVLSQSDQASTSSQSG  145.  D | 3 |
| 40 | MF288041.1 | Ecuador | Zamora-Chinchipe | >MF288041.1 Anaplasma marginale isolate Z1-Oeste major surface protein 1 alpha (msp1a) gene, partial cds GTGCCCCTGGCAGTGTAGGGTTTGTTTGTGTGTGTGTGTGTTATGTCAGAGTATGTGTCCTCCCACCCAG  CTGATAGCTCGTCAGCGAGTGGTGTGTTATCACAAAGTGGTCAGGCCAGTACATCGTCTCAATTAGGGAC  TGATAGCTCGTCAGCGAGTGGTCAGCAGCAAGAGAGTGGTGTGTCATCTCAAAGTGGTCAGGCCAGTACA  TCGTCTCAATTAGGGACTGATAGCTCGTCAGCGAGTGGTCAGCAGCAAGAGAGTGGTGTGTCATCTCAAA  GTGGTCAGGCCAGTACATCGTCTCAATTAGGGACTGATTGGCGGCAAGAGATGCGCTCCAAGGTTGCGAG  TGTTGAGTACATTTTGGCTGCTCGTGCCCTTATCTCTGTAGGGGTCTGTGCTGCTCAGGAAGAGATCGCG  AGATCGCTAGGGTATGCTCCCCTGCGTGTTG | GAGTGGTGTGTTATCACAAAGTGGTCAGGCCAGTACATCGTCTCAATTAGGGACTGATAGCTCGTCAGCGAGTGGTCAGCAGCAAGA  GAGTGGTGTGTCATCTCAAAGTGGTCAGGCCAGTACATCGTCTCAATTAGGGACTGATAGCTCGTCAGCGAGTGGTCAGCAGCAAGA  GAGTGGTGTGTCATCTCAAAGTGGTCAGGCCAGTACATCGTCTCAATTAGGGACTGAT | 146.  SGVLSQSGQASTSSQLGTDSSSASGQQQ  147.  SGVSSQSGQASTSSQLGTDSSSASGQQQ  148.  SGVSSQSGQASTSSQLGTD | 3 |
| 41 | MF288040.1 | Ecuador | Zamora-Chinchipe | >MF288040.1 Anaplasma marginale isolate N31-Este major surface protein 1 alpha (msp1a) gene, partial cds GTGCCCCTGGCAGTGTAGGGTTTGTTTATTTGTGTGTGTGTTATGTCAGAGTATGGGTCCTCTCAGCCAG  CTGATAGCTCGTCAGCGAGTGGTCAGCAGCAAGAGAGTAGTGTGTTATCTCAAAGTGATCAGGCCAGTAC  ATCGTCTCAATTAGGAACTGATAGCTCGTCAGCGAGTGGTCAGCAGCAAGAGAGTAGTGTGTTATCTCAA  AGTGATCAGGCCAGTACATCGTCTCAATTAGGAACTGATAGCTCGTCAGCGAGTGGTCAGCAGCAAGAGA  GTAGTGTGTTATCTCAAAGTGATCAGGCCAGTACATCGTCTCAATTAGGAACTGATAGCTCGTCAGCGAG  TGGTCAGCAGCAAGAGAGTAGTGTGTTATCTCAAAGTGATCAGGCCAGTACATCGTCTCAATCAGGAACT  GATTGGCGGCAAGAGATGCGCTCCAAGGTTGCGAGTGTTGAGTACATTTTGGCTGCTCGTGCCCTTATCT  CTGTAGGGGTCTATGCTGCTCAGGAAGAGATCGCGAGATCGCTAGGGTATGCTCCCCTGCGTGTTGCAGA  AGTCGAAGAAATCGTGAGGGATAGCCTTGTACGCAGCCAC | CTGATAGCTCGTCAGCGAGTGGTCAGCAGCAAGAGAGTAGTGTGTTATCTCAAAGTGATCAGGCCAGTACATCGTCTCAATTAGGAA  CTGATAGCTCGTCAGCGAGTGGTCAGCAGCAAGAGAGTAGTGTGTTATCTCAAAGTGATCAGGCCAGTACATCGTCTCAATTAGGAA  CTGATAGCTCGTCAGCGAGTGGTCAGCAGCAAGAGAGTAGTGTGTTATCTCAAAGTGATCAGGCCAGTACATCGTCTCAATTAGGAA  CTGATAGCTCGTCAGCGAGTGGTCAGCAGCAAGAGAGTAGTGTGTTATCTCAAAGTGATCAGGCCAGTACATCGTCTCAATCAGGAA  CTGAT | 149.  DSSSASGQQQESSVLSQSDQASTSSQLG  150.  DSSSASGQQQESSVLSQSDQASTSSQLG  151.  DSSSASGQQQESSVLSQSDQASTSSQLG  152.  DSSSASGQQQESSVLSQSDQASTSSQSG  153.  D | 4 |
| 42 | MF288039.1 | Ecuador | Zamora-Chinchipe | >MF288039.1 Anaplasma marginale isolate N20-Este major surface protein 1 alpha (msp1a) gene, partial cds GTGCCCCTGGCAGTGTAGGGTTTGTTTATTTGTGTGTGTGTTATGTCAGAGTATGGGTCCTCTCAGCCAG  CTGATAGCTCGTCAGCGAGTGGTCAGCAGCAAGAGAGTAGTGTGTTATCTCAAAGTGATCAGGCCAGTAC  ATCGTCTCAATTAGGAACTGATAGCTCGTCAGCGAGTGGTCAGCAGCAAGAGAGTAGTGTGTTATCTCAA  AGTGATCAGGCCAGTACATCGTCTCAATTAGGAACTGATAGCTCGTCAGCGAGTGGTCAGCAGCAAGAGA  GTAGTGTGTTATCTCAAAGTGATCAGGCCAGTACATCGTCTCAATCAGGAACTGATTGGCGGCAAGAGAT  GCGCTCCAAGGTTGCGAGTGTTGAGTACATTTTGGCTGCTCGTGCCCTTATCTCTGTAGGGGTCTATGCT  GCTCAGGAAGAGATCGCGAGATCGCTAGGGTATGCTCCCCTGCGTGTTGCAGAAGTCGAAGAAATCGTGA  GGGATAGCCT | CTGATAGCTCGTCAGCGAGTGGTCAGCAGCAAGAGAGTAGTGTGTTATCTCAAAGTGATCAGGCCAGTACATCGTCTCAATTAGGAA  CTGATAGCTCGTCAGCGAGTGGTCAGCAGCAAGAGAGTAGTGTGTTATCTCAAAGTGATCAGGCCAGTACATCGTCTCAATTAGGAA  CTGATAGCTCGTCAGCGAGTGGTCAGCAGCAAGAGAGTAGTGTGTTATCTCAAAGTGATCAGGCCAGTACATCGTCTCAATCAGGAA  CTGAT | 154.  DSSSASGQQQESSVLSQSDQASTSSQLG  155.  DSSSASGQQQESSVLSQSDQASTSSQLG  156.  DSSSASGQQQESSVLSQSDQASTSSQSG  157.  D | 3 |
| 43 | MF288038.1 | Ecuador | Zamora-Chinchipe | >MF288038.1 Anaplasma marginale isolate N15-Este major surface protein 1 alpha (msp1a) gene, partial cds GTGCCCCTGGCAGTGTAGGGTTTGTTTGTTTGTGTGTGTGTTATGTCAGAGTATGTGTCCTCCCAGCAAG  CTGATAGCTCGTCAGCGAGTGGTCAGCAGCAAGAGAGTAGTGTGTTATCTCAAAGTGATCAGGCCAGTAC  ATCGTCTCAATTAGGGACTGATAGCTCGTCAGCGAGTGGTCAGCAGCAAGAGAGTAGTGTGTTATCTCAA  AGTGATCAGGCCAGTACATCGTCTCAATTAGGGACTGATAGCTCGTCAGCGAGTGGTCAGCAGCAAGAGA  GTAGTGTGTCATCTCAAAGTGAGGCCAGTACATCGTCTCAATTAGGGACTGATAGCTCGTCAGCGAGTGG  TCAGCAGCAAGAGAGTAGTGTGTCATCTCAAAGTGAGGCCAGTACATCGTCTCAATTAGGGACTGATTGG  CGGCAAGAGATGCGCTCCAAGGTTGCGAGTGTTGAGTACATGTTGGCTGCTCGTGCCCTTATTTCTGTAG  GGGTCTATGCTGCTCAGGGAGAGATCGCGAGATCGCGAGGGTGTGCTCCCCTGCGTGTTGCA | First INDICE:  CTGATAGCTCGTCAGCGAGTGGTCAGCAGCAAGAGAGTAGTGTGTTATCTCAAAGTGATCAGGCCAGTACATCGTCTCAATTAGGGA  CTGATAGCTCGTCAGCGAGTGGTCAGCAGCAAGAGAGTAGTGTGTTATCTCAAAGTGATCAGGCCAGTACATCGTCTCAATTAGGGA  CTGATAGCTCGTCAGCGAGTGGTCAGCAGCAAGAGAGTAGTGTGTCATCTCAAAGTG-AGGCCAGTACATCGTCTCAATTAGGGA  CTGATAGCTCGTCAGCGAGTGGTCAGCAGCAAGAGAGTAGTGTGTCATCTCAAAGTG-AGGCCAGTACATCGTCTCAATTAGGGA  CTGAT  Second INDICE:  CTGATAGCTCGTCAGCGAGTGGTCAGCAGCAAGAGAGTAGTGTGTTATCTCAAAGTGATCAGGCCAGTACATCGTCTCAATTAGGGACTGATAGCTCGTCAGCGAGTGGTCAGCAGCAAGAGAGTAGTGTGTTATCTCAAAGTGATCAGGCCAGTACATCGTCTCAATTAGGGA  CTGATAGCTCGTCAGCGAGTGGTCAGCAGCAAGAGAGTAGTGTGTCATCTCAAAGTG-AGGCCAGTACATCGTCTCAATTAGGGACTGATAGCTCGTCAGCGAGTGGTCAGCAGCAAGAGAGTAGTGTGTCATCTCAAAGTGAGGCCAGTACATCGTCTCAATTAGGGA  CTGAT | First INDICE:  158.  DSSSASGQQQESSVLSQSDQASTSSQLG  159.  DSSSASGQQQESSVLSQSDQASTSSQLG  160.  DSSSASGQQQESSVSSQSEASTSSQLG  161.  DSSSASGQQQESSVSSQSEASTSSQLG  162.  D  Second INDICE:  163.  DSSSASGQQQESSVLSQSDQASTSSQLGTDSSSASGQQQESSVLSQSDQASTSSQLG  164.  DSSSASGQQQESSVSSQSEASTSSQLGTDSSSASGQQQESSVSSQSEASTSSQLG  165.  D | indices: first indice: 4, second indice: 2 |
| 44 | MF288037.1 | Ecuador | Loja | >MF288037.1 Anaplasma marginale isolate N9-Este major surface protein 1 alpha (msp1a) gene, partial cds GTGCCCCTGGCAGTGTAGGGTTTGTTTGTTTGTGTTGTGTTATGTCAGAGTATGTGTCCTCCCAGCAAGC  TGATAGCTCGTCAGCGAGTGGTCAGCAGCAAGAGAGTAGTGTGTTATCTCAAAGTGATCAGGCCAGTACA  TCGTCTCAATTAGGGACTGATAGCTCGTCAGCGAGTGGTCAGCAGCAAGAGAGTAGTGTGTTATCTCAAA  GTGATCAGGCCAGTACATCGTCTCAATTAGGGACTGATAGCTCGTCAGCGAGTGGTCAGCAGCAAGAGAG  TAGTGTGTTATCTCAAAGTGATCAAGCCAGTACATCGTCTCAATTAGGGACTGATTGGCGGCATGAGATG  CGCTCCAAGGTTGCGAGTGTTGAGTACATGTTGGCTGCTCGTGCCCTTATTTCTGTAGGGGTCTATGCTG  CTCAGGGAGAGATCGCGAG | TGATAGCTCGTCAGCGAGTGGTCAGCAGCAAGAGAGTAGTGTGTTATCTCAAAGTGATCAGGCCAGTACATCGTCTCAATTAGGGAC  TGATAGCTCGTCAGCGAGTGGTCAGCAGCAAGAGAGTAGTGTGTTATCTCAAAGTGATCAGGCCAGTACATCGTCTCAATTAGGGAC  TGATAGCTCGTCAGCGAGTGGTCAGCAGCAAGAGAGTAGTGTGTTATCTCAAAGTGATCAAGCCAGTACATCGTCTCAATTAGGGAC  TGAT | 166.  DSSSASGQQQESSVLSQSDQASTSSQLG  167.  DSSSASGQQQESSVLSQSDQASTSSQLG  168.  DSSSASGQQQESSVLSQSDQASTSSQLG  169.  D | 3 |
| 45 | MF288036.1 | Ecuador | Loja | >MF288036.1 Anaplasma marginale isolate Ch34-Sur major surface protein 1 alpha (msp1a) gene, partial cds GTGCCCCTGGCAGTGTAGGGTTTGTTTGTGTGTGTGTTATGTCAGAGTATGTGTCCAGCCAGTCAGCTGA  TAGCTCGTCAGCGAGTGGTCAGCAGCAAGAGAGTAGTGTGTCATCTCAAAGTGAGGCCAGTACATCGTCT  CAATTAGGGACTGATAGCTCGTCAGCGAGTGGTCAGCAGCAAGAGAGTAGTGTGTCATCTCAAAGTGAGG  CCAGTACATCGTCTCAATTAGGGACTGATTGGCGGCAAGAGATGCGCTCCAAGGTTGCGAGTGTTGAGTA  CATGTTGGCTGCTCGTGCCCTTATTTCTGTAGGGGTCTATGCTGCTCAGGGAGAGATCGCGAGATCGCGA | TAGCTCGTCAGCGAGTGGTCAGCAGCAAGAGAGTAGTGTGTCATCTCAAAGTGAGGCCAGTACATCGTCTCAATTAGGGACTGA  TAGCTCGTCAGCGAGTGGTCAGCAGCAAGAGAGTAGTGTGTCATCTCAAAGTGAGGCCAGTACATCGTCTCAATTAGGGACTGA  T | 170.  SSSASGQQQESSVSSQSEASTSSQLGT  171.  SSSASGQQQESSVSSQSEASTSSQLGT | 2 |
| 46 | MF288035.1 | Ecuador | Loja | >MF288035.1 Anaplasma marginale isolate Ch28-Sur major surface protein 1 alpha (msp1a) gene, partial cds GTGCCCCTGGCAGTGTAGGGTTTATTTGTTTGTGTGTGTGTTATGTCAGCAGAGTATGTGTCCTCCCAGC  CAGCTGGTAGCTCGTCAGCGGGTGGTCAGCAGCAAGAGAGTAGTGTGTCATCTCAAAGTGAGGCCAGTAC  ATCGTCTCAATTAGGAGGAGCTGATAGCTCGTCAGCGAGTGGTCAGCAGCAAGAGAGTAGTGTGTTATCT  CAAAGTGAGGCCAGTACATCGTCTCAATTAGGAGGAGCTGATAGCTCGTCAGCGAGTGGTCAGCAGCAAG  AGAGTAGTGTGTCATCTCAAAGTGAGGCCAGTACATCGTCTCAATTAGGAGGAGCTGATAGCTCGTCAGC  GAGTGGTCAGCAGCAAGAGAGTAGTGTGTCATCTCAAAGTGAGGCCAGTACATCGTCTCAATTAGGGACT  GATTGGCGGCAAGAGATGCGCTCCAAGGTTGCGAGTGTTGAGTACATTTTGGCTGCTCGTGCCCTTATCT  CTGTAGGGGTCTATGCTGCTC | AGCTGGTAGCTCGTCAGCGGGTGGTCAGCAGCAAGAGAGTAGTGTGTCATCTCAAAGTGAGGCCAGTACATCGTCTCAATTAGGAGG  AGCTGATAGCTCGTCAGCGAGTGGTCAGCAGCAAGAGAGTAGTGTGTTATCTCAAAGTGAGGCCAGTACATCGTCTCAATTAGGAGG  AGCTGATAGCTCGTCAGCGAGTGGTCAGCAGCAAGAGAGTAGTGTGTCATCTCAAAGTGAGGCCAGTACATCGTCTCAATTAGGAGG  AGCTGATAGCTCGTCAGCGAGTGGTCAGCAGCAAGAGAGTAGTGTGTCATCTCAAAGTGAGGCCAGTACATCGTCTCAATTAGG | 172.  AGSSSAGGQQQESSVSSQSEASTSSQLG  173.  ADSSSASGQQQESSVLSQSEASTSSQLG  174.  ADSSSASGQQQESSVSSQSEASTSSQLG  175.  ADSSSASGQQQESSVSSQSEASTSSQL | 4 |
| 47 | MF288034.1 | Ecuador | Loja | >MF288034.1 Anaplasma marginale isolate Y69-Norte major surface protein 1 alpha (msp1a) gene, partial cds GTGCCCCTGGCAGTGTAGGGTTTGTTTGTGTGTGTGTTATGTCAGAGTATGTGTCCAGCCAGTCAGCTGA  TAGCTCGTCAGCGAGTGGTCAGCAGCAAGAGAGTAGTGTGTCATCTCAAAGTGAGGCCAGTACATCGTCT  CAATTAGGAGCTGATAGCTCGTCAGCGAGTGGTCAGCAGCAAGAGAGTAGTGTGTCATCTCAAAGTGATC  AGGCCAGTACATCGTCTCAATTAGGGACTGATAGCTCGTCAGCGAGTGGTCAGCAGCAAGAGAGTAGTGT  GTCATCTCAAAGTGAGGCCAGTACATCGTCTCAATTAGGGACTGATAGCTCGTCAGCGAGTGGTCAGCAG  CAAGAGAGTAGTGTGTCATCTCAAAGTGAGGCCAGTACATCGTCTCAATTAGGGACTGATTGGCGGCAAG  AGATGCGCTCCAAGGTTGCGAGTGTTGAGTACATTTTGGCTGCTCGTGCCCTTATTTCTGTAGGGGTCTA  TGCTGCTCAGGGAGAGATCGCGAGATCGCGAGGGTGTGCTCCCCTGCGTGTTGCAGAAGTCGAAGAAATC  GTGAGGGATGGCCTTGTACGCAGCCACTTTCATGATAGTGGCCTTTCACTAGGCTCCATACGACTCGTGC  T | First INDICE:  TAGCTCGTCAGCGAGTGGTCAGCAGCAAGAGAGTAGTGTGTCATCTCAAAGTGAGGCCAGTACATCGTCTCAATTA-GGAGCTGA  TAGCTCGTCAGCGAGTGGTCAGCAGCAAGAGAGTAGTGTGTCATCTCAAAGTGATCAGGCCAGTACATCGTCTCAATTAGGGACTGA  TAGCTCGTCAGCGAGTGGTCAGCAGCAAGAGAGTAGTGTGTCATCTCAAAGTGAGGCCAGTACATCGTCTCAATTAGGGACTGA  TAGCTCGTCAGCGAGTGGTCAGCAGCAAGAGAGTAGTGTGTCATCTCAAAGTGAGGCCAGTACATCGTCTCAATTAGGGACTGA  T  Second INDICE:  TAGCTCGTCAGCGAGTGGTCAGCAGCAAGAGAGTAGTGTGTCATCTCAAAGTGAGGCCAGTACATCGTCTCAATTAGGAGCTGATAGCTCGTCAGCGAGTGGTCAGCAGCAAGAGAGTAGTGTGTCATCTCAAAGTGATCAGGCCAGTACATCGTCTCAATTAGGGACTGA  TAGCTCGTCAGCGAGTGGTCAGCAGCAAGAGAGTAGTGTGTCATCTCAAAGTGAGGCCAGTACATCGTCTCAATTAGG-GACTGATAGCTCGTCAGCGAGTGGTCAGCAGCAAGAGAGTAGTGTGTCATCTCAAAGTGAGGCCAGTACATCGTCTCAATTAGGGACTGA  T | First INDICE:  176.  SSSASGQQQESSVSSQSEASTSSQLGA  177.  SSSASGQQQESSVSSQSDQASTSSQLGT  178.  SSSASGQQQESSVSSQSEASTSSQLGT  179.  SSSASGQQQESSVSSQSEASTSSQLGT  Second INDICE:  180. SSSASGQQQESSVSSQSEASTSSQLGADSSSASGQQQESSVSSQSDQASTSSQLGT  181.  SSSASGQQQESSVSSQSEASTSSQLGTDSSSASGQQQESSVSSQSEASTSSQLGT | Total 2 indices: First Indice: 4, Second Indice: 2 |
| 48 | MF288033.1 | Ecuador | Loja | >MF288033.1 Anaplasma marginale isolate Y49-Norte major surface protein 1 alpha (msp1a) gene, partial cds GTGCCCCTGGCAGTGTAGGGTTTGTTTGTGTGTGTGTTATGTCAGAGTATGTGTCCAGCCAGTCAGCTGA  TAGCTCGTCAGCGAGTGGTCAGCAGCAAGAGAGTAGTGTGTCATCTCAAAGTGAGGCCAGTACATCGTCT  CAATTAGGAGGAGCTGATAGCTCGTCAGCGAGTGGTCAGCAGCAAGAGAGTAGTGTGTCATCTCAAAGTG  AGGCCAGTACATCGTCTCAATTAGGAGGAGCTGATAGCTCGTCAGCGAGTGGTCAGCAGCAAGAGAGTAG  TGTGTCATCTCAAAGTGAGGCCAGTACATCGTCTCAATTAGGAGGAGCTGATAGCTCGTCAGCGAGTGGT  CAGCAGCAAGAGAGTAGTGTGTCATCTCAAAGTGAGGCCAGTACATCGTCTCAATTAGGAGGAGCTGATA  GCTCGTCAGCGAGTGGTCAGCAGCAAGAGAGTAGTGTGTCATCTCAAAGTGAGGCCAGTACATCGTCTCA  ATTAGGAGGAGCTGATAGCTCGTCAGCGAGTGGTCAGCAGCAAGAGAGTAGTGTGTCATCTCAAAGTGAG  GCCAGTACATCGTCTCAATTAGGAGGAGCTGATAGCTCGTCAGCGAGTGGTCAGCAGCAAGAGAGTAGTG  TGTCATCTCAAAGTGAGGCCAGTACATCGTCTCAATTAGGGACTGATTGGCGGCAAGAGATGCGCTCCAA  GGTTGCGAGTGTTGAGTACATTTTGGCTGCTCGTGCCCTTATCTCTGTAGGGGTCTATGCTGCTCAGAGA  ATGATCGCGCAATCGCAAGGGTGTGCTTCCCTGAGTGTTGCAGAAGTCGAAGGCATCGTGAAGGATAGCC  TTGTACGCAG | TAGCTCGTCAGCGAGTGGTCAGCAGCAAGAGAGTAGTGTGTCATCTCAAAGTGAGGCCAGTACATCGTCTCAATTAGGAGGAGCTGA  TAGCTCGTCAGCGAGTGGTCAGCAGCAAGAGAGTAGTGTGTCATCTCAAAGTGAGGCCAGTACATCGTCTCAATTAGGAGGAGCTGA  TAGCTCGTCAGCGAGTGGTCAGCAGCAAGAGAGTAGTGTGTCATCTCAAAGTGAGGCCAGTACATCGTCTCAATTAGGAGGAGCTGA  TAGCTCGTCAGCGAGTGGTCAGCAGCAAGAGAGTAGTGTGTCATCTCAAAGTGAGGCCAGTACATCGTCTCAATTAGGAGGAGCTGA  TAGCTCGTCAGCGAGTGGTCAGCAGCAAGAGAGTAGTGTGTCATCTCAAAGTGAGGCCAGTACATCGTCTCAATTAGGAGGAGCTGA  TAGCTCGTCAGCGAGTGGTCAGCAGCAAGAGAGTAGTGTGTCATCTCAAAGTGAGGCCAGTACATCGTCTCAATTAGGAGGAGCTGA  TAGCTCGTCAGCGAGTGGTCAGCAGCAAGAGAGTAGTGTGTCATCTCAAAGTGAGGCCAGTACAT CGTCTCAATTAGG | 182.  SSSASGQQQESSVSSQSEASTSSQLGGA  183.  SSSASGQQQESSVSSQSEASTSSQLGGA  184.  SSSASGQQQESSVSSQSEASTSSQLGGA  185.  SSSASGQQQESSVSSQSEASTSSQLGGA  186.  SSSASGQQQESSVSSQSEASTSSQLGGA  187.  SSSASGQQQESSVSSQSEASTSSQLGGA  188.  SSSASGQQQESSVSSQSEASTSSQL | 7 |
| 49 | MF288032.1 | Ecuador | Loja | >MF288032.1 Anaplasma marginale isolate Y27-Norte major surface protein 1 alpha (msp1a) gene, partial cds GTGCCCCTGGCAGTGTAGGGTTTATTTGTTTGTGTGTGTGTTATGTCAGCAGAGTATGTGTCCTCCCAGC  CAGCTGGTAGCTCGTCAGCGGGTGGTCAGCAGCAAGAGAGTAGTGTGTCATCTCAAAGTGAGGCCAGTAC  ATCGTCTCAATTAGGAGAGCTGATAGCTCGTCAGCGAGTGGTCAGCAGCAAGAGAGTAGTGTGTTATCT  CAAAGTGAGGCCAGTACATCGTCTCAATTAGGAGGAGCTGATAGCTCGTCAGCGAGTGGTCAGCAGCAAG  AGAGTAGTGTGTCATCTCAAAGTGAGGCCAGTACATCGTCTCAATTAGGAGGAGCTGATAGCTCGTCAGC  GAGTGGTCAGCAGCAAGAGAGTAGTGTGTTATCTCAAAGTGAGGCCAGTACATCGTCTCAATTAGGAGGA  GCTGATAGCTCGTCAGCGAGTGGTCAGCAGCAAGAGAGTAGTGTGTCATCTCAAAGTGAGGCCAGTACAT  CGTCTCAATTAGGGACTGATTGGCGGCAAGAGATGCGCTCCAAGGTTGCGAGTGTTGAGTACATTTTGGC  TGCTCGTGCCCTTATCTCTGTAGGGGTCTATGCTGCTCAGAGAATGATCGCGCAATCGCAAGGGTGTGCT  TCCCTGAGTGTTGCAGAAGTCGAAGGCATCGTGAAGGATAGCCTTGTACGCAGCCACTTTCATGATAGTG  GCCTTTCACTAGGCTCCATACG | AGCTGGTAGCTCGTCAGCGGGTGGTCAGCAGCAAGAGAGTAGTGTGTCATCTCAAAGTGAGGCCAGTACATCGTCTCAATTAGGAGG  AGCTGATAGCTCGTCAGCGAGTGGTCAGCAGCAAGAGAGTAGTGTGTTATCTCAAAGTGAGGCCAGTACATCGTCTCAATTAGGAGG  AGCTGATAGCTCGTCAGCGAGTGGTCAGCAGCAAGAGAGTAGTGTGTCATCTCAAAGTGAGGCCAGTACATCGTCTCAATTAGGAGG  AGCTGATAGCTCGTCAGCGAGTGGTCAGCAGCAAGAGAGTAGTGTGTTATCTCAAAGTGAGGCCAGTACATCGTCTCAATTAGGAGG  AGCTGATAGCTCGTCAGCGAGTGGTCAGCAGCAAGAGAGTAGTGTGTCATCTCAAAGTGAGGCCAGTACATCGTCTCAATTAGG | 189.  AGSSSAGGQQQESSVSSQSEASTSSQLG  190.  ADSSSASGQQQESSVLSQSEASTSSQLG  191.  ADSSSASGQQQESSVSSQSEASTSSQLG  192.  ADSSSASGQQQESSVLSQSEASTSSQLG  193.  ADSSSASGQQQESSVSSQSEASTSSQL | 5 |
| 50 | Egypt | El-Wady El-Geded | MN370077 | >MN370077.1 Anaplasma marginale isolate 2cattle major surface protein 1a (msp1a) gene, partial cds  TTGCGATTACACGTTCCGTATGTTACAATCGGGCTGCCGGTGTGGTAGCGTGCTGGTTGTGTGGTTGTCC  TCTTTCCCGATGTTGGGTCGTTCGTTTTACGTCTCACAAGTTTGTACGCTGTGCCCCTGGCAGTGTAGGG  TTTGTTTGTGTGTGTGTGTGTTATGTCAGAGTTGTCCCTTCAGCCAACTGATAGCTCGTCAGCGAGTGGT  CAGCAGCAAGAGAGTAGTGTGTTATCTCCAAGTGGTCAGGCCAGTACATCGTCTCAATTAGGAGCTGATA  GCTCGTCAGCGAGTGGTCAGCAGCAAGAGAGTAGTGTGTTATCTCCAAGTGGTCAGGCCAGTACATCGTC  TCAATCAGGAACTGATAGCTCGTCAGCGAGTGGTCAGCAGCAAGAGAGTAGTGTGTTATCTCAAAGTGAT  CAGGCCAGTACATCGTCTCAATTAGGAACTGATAGCTCGTCAACGAGTGGTCAGCAGCAAGAGAGTAGTG  TGTTATCTCAAAGTGATCAGGCCAGTACATCGTCTCAATCAGGAACTGATTGGCGGCAAGAGATGCGCTC  CAAGGTTGCGAGTGTTGAGTACATGTTGGCTGCTCGTGCCCTTATTTCTGTAGGGGTCTATGCTGCTCAG  GAAGGGATCGCGAGATCGCTAGGGCATACTCCCCTGCGTGTTGCAGAAGTCGAAGCAATCGTGAGGGATA  GCCT | 1. AACTGATAGCTCGTCAGCGAGTGGTCAGCAGCAAGAGAGTAGTGTGTTATCTCCAAGTGGTCAGGCCAGTACATCGTCTCAATTAGG  2. AGCTGATAGCTCGTCAGCGAGTGGTCAGCAGCAAGAGAGTAGTGTGTTATCTCCAAGTGGTCAGGCCAGTACATCGTCTCAATCAGG  3. AACTGATAGCTCGTCAGCGAGTGGTCAGCAGCAAGAGAGTAGTGTGTTATCTCAAAGTGATCAGGCCAGTACATCGTCTCAATTAGG  4. AACTGATAGCTCGTCAACGAGTGGTCAGCAGCAAGAGAGTAGTGTGTTATCTCAAAGTGATCAGGCCAGTACATCGTCTCAATCAGG | 194.  TDSSSASGQQQESSVLSPSGQASTSSQL  195.  ADSSSASGQQQESSVLSPSGQASTSSQS  196.  TDSSSASGQQQESSVLSQSDQASTSSQL  197.  TDSSSTSGQQQESSVLSQSDQASTSSQS | 4 |
| 51 | Egypt | El-Wady El-Geded | MN370076 | >MN370076.1 Anaplasma marginale isolate 370cattle major surface protein 1a (msp1a) gene, partial cds  TATATTAATCTTGCGATTACACGTTCCGTATGTTACAATCAGGCCGCCGGTGTGGTAGCGTGCTGGTTGT  GTGGTTGTCCTCTTTCCCGATGTTGGGTCGTTCGTTTTACGTCGCACAAGTTTGTACGCTGTGCCCCTGG  CAGTGTAGGGTTTATTTGTTTGTGTGTGTGTTATGTCAGCAGAGTATGTGTCCCCCCAGCCAGCTGATAG  CTCGTCAGCGGGTGGTCAGCAGCAAGAGAGTAGTGTGTCATCTCAAAGTGATCAGGCCAGTACATCGTCT  CAATTAGGAGCTGATAGCTCGTCAGCGGGTGGTCAGCAGCAAGAGAGTAGTGTGTCATCTCAAAGTGATC  AGGCCAGTACATCGTCTCAATTAGGAGCTGATAGCTCGTCAGCGGGTGGTCAGCAGCAAGAGAGTAGTGT  GTCATCTCAAAGTGATCAGGCCAGTACATCGTCTCAATTAGGAGCTGATAGCTCGTCAGCGGGTGGTCAG  CAGCAAGAGAGTAGTGTGTCATCTCAAAGTGGTCAGGCCAGTACATCGTCTCAATTAGGGACTGATTGGC  GGCAAGAGATGCGCTCCAAGGTTGCGAGTGTTGAGTACATGTTGGCTGCTCGTGCCCTTATCTCTGTAGG  GGTCTATGCTGCTCAGGGAGAGATCGCGAGATCGCGAGGGTGTGCTCCCCTGCGTGTTGCAGAAGTCGAA  GAAATCGTGAGGGATGGCCTTGTACGCAGCCACTTTCATGATAGTGGCCTTTCACTAGGCTCCATACGAC  TCGTGCTTATGCAGGTTGGGGATAAGTTGGGGCTACAAGGTTTGAAGATTGGCGAAGGGTACGCCACCTA  TCTCGCGCAAGCGTTTGCTGACAGCGTGGTGGTTGCGGCT | AGCTGATAGCTCGTCAGCGGGTGGTCAGCAGCAAGAGAGTAGTGTGTCATCTCAAAGTGATCAGGCCAGTACATCGTCTCAATTAGG  AGCTGATAGCTCGTCAGCGGGTGGTCAGCAGCAAGAGAGTAGTGTGTCATCTCAAAGTGATCAGGCCAGTACATCGTCTCAATTAGG  AGCTGATAGCTCGTCAGCGGGTGGTCAGCAGCAAGAGAGTAGTGTGTCATCTCAAAGTGATCAGGCCAGTACATCGTCTCAATTAGG  AGCTGATAGCTCGTCAGCGGGTGGTCAGCAGCAAGAGAGTAGTGTGTCATCTCAAAGTGGTCAGGCCAGTACATCGTCTCAATTAGG | 198.  ADSSSAGGQQQESSVSSQSDQASTSSQL  199.  ADSSSAGGQQQESSVSSQSDQASTSSQL  200.  ADSSSAGGQQQESSVSSQSDQASTSSQL  201.  ADSSSAGGQQQESSVSSQSGQASTSSQL | 4 |
| 52 | Egypt | El-Wady El-Geded | MN370075 | >MN370075.1 Anaplasma marginale isolate 311cattle major surface protein 1a (msp1a) gene, partial cds  GGTGTGGTAGCGTGCTGGTTGTGTGGCTGTCCTCTTTCCCGATGTTGGGTCGTTCGTTTTACGTCGCACA  AGTTTGTACGCTGTGCCCCTGGCAGTGTAGGGTTTATTTGTTTGTGTGTGTGTTATGTCAGCAGAGTATG  TGTCCTCCCAGCCAGCTGGTAGCTCGTCAGCGGGTGGTCAGCAGCAAGAGAGTAGTGTGTCATCTCAAAG  TGATCAGGCCAGTACATCGTCTCAATTAGGAGCTGATAGCTCGTCAGCGGGTGGTCAGCAGCAAGAGAGT  AGTGTGTCATCTCAAAGTGATCAGGCCAGTACATCGTCTCAATTAGGAGCTGATAGCTCGTCAGCGGGTG  GTCAGCAGCAAGAGAGTAGTGTGTCATCTCAAAGTGATCAGGCCAGTACATCGTCTCAATTAGGAGCTGA  TAGCTCGTCAGCGGGTGGTCAGCAGCAAGAGAGTAGTGTGTCATCTCAAAGTGATCAGGCCAGTACATCG  TCTCAATTAGGAGCTGATAGCTCGTCAGCGAGTGGTCAGCAGCAAGAGAGTAGTGTGTCATCTCAAAGTG  GTCAGGCCAGTACATCGTCTCAATTAGGGACTGATTGGCGGCAAGAGATGCGCTCCAAGGTTGCGAGTGT  TGAGTACATGTTGGCTGCTCGTGCCCTTATTTCTGTAGGGGTCTATGCTGCTCAGGGAGAGATCGCGAGA  TCGCGAGGGTGTGCTCCCCTGCGTGTTGCAGAAGTCGAAGAAATCGTGAGGGATGGCCTTGTACGCAGCC  ACTTTCATGATAGTGGCCTTTCACTAGGCTCCATACGACTCGTGCTTATGCAGGTTGGGGATAAGTTGGG  GCTACAAGGTTTGAAGATTGGCGAAAGGTACGCCACCTATCTCGCGCAAGCGT | AGCTGGTAGCTCGTCAGCGGGTGGTCAGCAGCAAGAGAGTAGTGTGTCATCTCAAAGTGATCAGGCCAGTACATCGTCTCAATTAGG  AGCTGATAGCTCGTCAGCGGGTGGTCAGCAGCAAGAGAGTAGTGTGTCATCTCAAAGTGATCAGGCCAGTACATCGTCTCAATTAGG  AGCTGATAGCTCGTCAGCGGGTGGTCAGCAGCAAGAGAGTAGTGTGTCATCTCAAAGTGATCAGGCCAGTACATCGTCTCAATTAGG  AGCTGATAGCTCGTCAGCGGGTGGTCAGCAGCAAGAGAGTAGTGTGTCATCTCAAAGTGATCAGGCCAGTACATCGTCTCAATTAGG  AGCTGATAGCTCGTCAGCGAGTGGTCAGCAGCAAGAGAGTAGTGTGTCATCTCAAAGTGGTCAGGCCAGTACATCGTCTCAATTAGG | 202.  AGSSSAGGQQQESSVSSQSDQASTSSQL  203.  ADSSSAGGQQQESSVSSQSDQASTSSQL  204.  ADSSSAGGQQQESSVSSQSDQASTSSQL  205.  ADSSSAGGQQQESSVSSQSDQASTSSQL  206.  ADSSSASGQQQESSVSSQSGQASTSSQL | 5 |
| 53 | Egypt | El-Wady El-Geded | MN370074 | >MN370074.1 Anaplasma marginale isolate 136cattle major surface protein 1a (msp1a) gene, partial cds  TATATTAATCTTGCGATTACACGTTCCGTATGTTACAATCAGGCCGCCGGTGTGGTAGCGTGCTGGTTGT  GTGGCTGTCCTCTTTCCCGATGTTGGGTCGTTCGTTTTACGTCGCACAAGTTTGTACGCTGTGCCCCTGG  CAGTGTAGGGTTTATTTGTTTGTGTGTGTGTTATGTCAGCAGAGTATGTGTCCTCCCAGCCAGCTGGTAG  CTCGTCAGCGGGTGGTCAGCAGCAAGAGAGTAGTGTGTCATCTCAAAGTGATCAGGCCAGTACATCGTCT  CAATTAGGAGCTGATAGCTCGTCAGCGGGTGGTCAGCAGCAAGAGAGTAGTGTGTCATCTCAAAGTGATC  AGGCCAGTACATCGTCTCAATTAGGAGCTGATAGCTCGTCAGCGGGTGGTCAGCAGCAAGAGAGTAGTGT  GTCATCTCAAAGTGATCAGGCCAGTACATCGTCTCAATTAGGAGCTGATAGCTCGTCAGCGGGTGGTCAG  CAGCAAGAGAGTAGTGTGTCATCTCAAAGTGATCAGGCCAGTACATCGTCTCAATTAGGAGCTGATAGCT  CGTCAGCGAGTGGTCAGCAGCAAGAGAGTAGTGTGTCATCTCAAAGTGGTCAGGCCAGTACATCGTCTCA  ATTAGGGACTGATTGGCGGCAAGAGATGCGCTCCAAGGTTGCGAGTGTTGAGTACATGTTGGCTGCTCGT  GCCCTTATTTCTGTAGGGGTCTATGCTGCTCAGGGAGAGATCGCGAGATCGCGAGGGTGTGCTCCCCTGC  GTGTTGCAGAAGTCGAAGAAATCGTGAGGGATGGCCTTGTACGCAGCCACTTTCATGATAGTGGCCTTTC  ACTAGGCTCCATACGACTCGTGCTTATGCAGGTTGGGGATAAGTTGGGGCTACAAGGTTTGAAGATTGGC  GAAAGGTACGCCACCTATCTCGCGCAAGCGTTTGCTGACAGCGTGGTGGTTGCGGCTGATGTTCAGAGTA  GTGGTGCGTGCCCTGCCAGCCTTGACAGCGCGATCGC | AGCTGGTAGCTCGTCAGCGGGTGGTCAGCAGCAAGAGAGTAGTGTGTCATCTCAAAGTGATCAGGCCAGTACATCGTCTCAATTAGG  AGCTGATAGCTCGTCAGCGGGTGGTCAGCAGCAAGAGAGTAGTGTGTCATCTCAAAGTGATCAGGCCAGTACATCGTCTCAATTAGG  AGCTGATAGCTCGTCAGCGGGTGGTCAGCAGCAAGAGAGTAGTGTGTCATCTCAAAGTGATCAGGCCAGTACATCGTCTCAATTAGG  AGCTGATAGCTCGTCAGCGGGTGGTCAGCAGCAAGAGAGTAGTGTGTCATCTCAAAGTGATCAGGCCAGTACATCGTCTCAATTAGG  AGCTGATAGCTCGTCAGCGAGTGGTCAGCAGCAAGAGAGTAGTGTGTCATCTCAAAGTGGTCAGGCCAGTACATCGTCTCAATTAGG | 207.  AGSSSAGGQQQESSVSSQSDQASTSSQL  208.  ADSSSAGGQQQESSVSSQSDQASTSSQL  209.  ADSSSAGGQQQESSVSSQSDQASTSSQL  210.  ADSSSAGGQQQESSVSSQSDQASTSSQL  211.  ADSSSASGQQQESSVSSQSGQASTSSQL | 5 |
| 54 | Egypt | El-Wady El-Geded | MN370073 | >MN370073.1 Anaplasma marginale isolate 134cattle major surface protein 1a (msp1a) gene, partial cds  ATATTAATCTTGCGATTACACGTTCCGTATGTTACAATCAGGCCGCCGGTGTGGTAGCGTGCTGGTTGTG  TGGCTGTCCTCTTTCCCGATGTTGGGTCGTTCGTTTTACGTCGCACAAGTTTGTACGCTGTGCCCCTGGC  AGTGTAGGGTTTATTTGTTTGTGTGTGTGTTATGTCAGCAGAGTATGTGTCCTCCCAGCCAGCTGGTAGC  TCGTCAGCGGGTGGTCAGCAGCAAGAGAGTAGTGTGTCATCTCAAAGTGATCAGGCCAGTACATCGTCTC  AATTAGGAGCTGATAGCTCGTCAGCGGGTGGTCAGCAGCAAGAGAGTAGTGTGTCATCTCAAAGTGATCA  GGCCAGTACATCGTCTCAATTAGGAGCTGATAGCTCGTCAGCGGGTGGTCAGCAGCAAGAGAGTAGTGTG  TCATCTCAAAGTGATCAGGCCAGTACATCGTCTCAATTAGGAGCTGATAGCTCGTCAGCGGGTGGTCAGC  AGCAAGAGAGTAGTGTGTCATCTCAAAGTGATCAGGCCAGTACATCGTCTCAATTAGGAGCTGATAGCTC  GTCAGCGAGTGGTCAGCAGCAAGAGAGTAGTGTGTCATCTCAAAGTGGTCAGGCCAGTACATCGTCTCAA  TTAGGGACTGATTGGCGGCAAGAGATGCGCTCCAAGGTTGCGAGTGTTGAGTACATGTTGGCTGCTCGTG  CCCTTATTTCTGTAGGGGTCTATGCTGCTCAGGGAGAGATCGCGAGATCGCGAGGGTGTGCTCCCCTGCG  TGTTGCAGAAGTCGAAGAAATCGTGAGGGATGGCCTTGTACGCAGCCACTTTCATGATAGTGGCCTTTCA  CTAGGCTCCATACGACTCGTGCTTATGCAGGTTGGGGATAAGTTGGGGCTACAAGGTTTGAAGATTGGCG  AAAGGTACGCCACCTATCTCGCGCAAGCGTTTGCTGACAGCGTGGTGGTTGCGGCTGATGTTCAGAGTAG  TGGTGCGTGCCCTGCCAGCCTTGACA | AGCTGGTAGCTCGTCAGCGGGTGGTCAGCAGCAAGAGAGTAGTGTGTCATCTCAAAGTGATCAGGCCAGTACATCGTCTCAATTAGG  AGCTGATAGCTCGTCAGCGGGTGGTCAGCAGCAAGAGAGTAGTGTGTCATCTCAAAGTGATCAGGCCAGTACATCGTCTCAATTAGG  AGCTGATAGCTCGTCAGCGGGTGGTCAGCAGCAAGAGAGTAGTGTGTCATCTCAAAGTGATCAGGCCAGTACATCGTCTCAATTAGG  AGCTGATAGCTCGTCAGCGGGTGGTCAGCAGCAAGAGAGTAGTGTGTCATCTCAAAGTGATCAGGCCAGTACATCGTCTCAATTAGG  AGCTGATAGCTCGTCAGCGAGTGGTCAGCAGCAAGAGAGTAGTGTGTCATCTCAAAGTGGTCAGGCCAGTACATCGTCTCAATTAGG | 212.  AGSSSAGGQQQESSVSSQSDQASTSSQL  213.  ADSSSAGGQQQESSVSSQSDQASTSSQL  214.  ADSSSAGGQQQESSVSSQSDQASTSSQL  215.  ADSSSAGGQQQESSVSSQSDQASTSSQL  216.  ADSSSASGQQQESSVSSQSGQASTSSQL | 5 |
| 55 | Egypt | El-Wady El-Geded | MN370072 | >MN370072.1 Anaplasma marginale isolate 133cattle major surface protein 1a (msp1a) gene, partial cds  TGTTAAAATTTAGTATATTAATCTTGCGATTACACGTTCCGTATGTTACAATCAGGCCGCCGGTGTGGTA  GCGTGCTGGTTGTGTGGCTGTCCTCTTTCCCGATGTTGGGTCGTTCGTTTTACGTCGCACAAGTTTGTAC  GCTGTGCCCCTGGCAGTGTAGGGTTTATTTGTTTGTGTGTGTGTTATGTCAGCAGAGTATGTGTCCTCCC  AGCCAGCTGGTAGCTCGTCAGCGGGTGGTCAGCAGCAAGAGAGTAGTGTGTCATCTCAAAGTGATCAGGC  CAGTACATCGTCTCAATTAGGAGCTGATAGCTCGTCAGCGGGTGGTCAGCAGCAAGAGAGTAGTGTGTCA  TCTCAAAGTGATCAGGCCAGTACATCGTCTCAATTAGGAGCTGATAGCTCGTCAGCGGGTGGTCAGCAGC  AAGAGAGTAGTGTGTCATCTCAAAGTGATCAGGCCAGTACATCGTCTCAATTAGGAGCTGATAGCTCGTC  AGCGGGTGGTCAGCAGCAAGAGAGTAGTGTGTCATCTCAAAGTGATCAGGCCAGTACATCGTCTCAATTA  GGAGCTGATAGCTCGTCAGCGAGTGGTCAGCAGCAAGAGAGTAGTGTGTCATCTCAAAGTGGTCAGGCCA  GTACATCGTCTCAATTAGGGACTGATTGGCGGCAAGAGATGCGCTCCAAGGTTGCGAGTGTTGAGTACAT  GTTGGCTGCTCGTGCCCTTATTTCTGTAGGGGTCTATGCTGCTCAGGGAGAGATCGCGAGATCGCGAGGG  TGTGCTCCCCTGCGTGTTGCAGAAGTCGAAGAAATCGTGAGGGATGGCCTTGTACGCAGCCACTTTCATG  ATAGTGGCCTTTCACTAGGCTCCATACGACTCGTGCTTATGCAGGTTGGGGATAAGTTGGGGCTACAAGG  TTTGAAGATTGGCGAAAGGTACGCCACCTATCTCGCGCAAGCGTTTGCTGACAGCGTGGTGGTTGCGGCT  GATGTTCAGAGTAGTGGTGCGTGCCCTGCCAGCC | AGCTGGTAGCTCGTCAGCGGGTGGTCAGCAGCAAGAGAGTAGTGTGTCATCTCAAAGTGATCAGGCCAGTACATCGTCTCAATTAGG  AGCTGATAGCTCGTCAGCGGGTGGTCAGCAGCAAGAGAGTAGTGTGTCATCTCAAAGTGATCAGGCCAGTACATCGTCTCAATTAGG  AGCTGATAGCTCGTCAGCGGGTGGTCAGCAGCAAGAGAGTAGTGTGTCATCTCAAAGTGATCAGGCCAGTACATCGTCTCAATTAGG  AGCTGATAGCTCGTCAGCGGGTGGTCAGCAGCAAGAGAGTAGTGTGTCATCTCAAAGTGATCAGGCCAGTACATCGTCTCAATTAGG  AGCTGATAGCTCGTCAGCGAGTGGTCAGCAGCAAGAGAGTAGTGTGTCATCTCAAAGTGGTCAGGCCAGTACATCGTCTCAATTAGG | 217.  AGSSSAGGQQQESSVSSQSDQASTSSQL  218.  ADSSSAGGQQQESSVSSQSDQASTSSQL  219.  ADSSSAGGQQQESSVSSQSDQASTSSQL  220.  ADSSSAGGQQQESSVSSQSDQASTSSQL  221.  ADSSSASGQQQESSVSSQSGQASTSSQL | 5 |
| 56 | Egypt | El-Wady El-Geded | MN370071 | >MN370071.1 Anaplasma marginale isolate 337cattle major surface protein 1a (msp1a) gene, partial cds  TATATTAATCTTGCGATTACACGTTCCGTATGTTACAATCAGGCCGCCGGTGTGGTAGCGTGCTGGTTGT  GTGGTTGTCCTCTTTCCCGATGTTGGGTCGTTCGTTTTACGTCGCACAAGTTTGTACGCTGTGCCCCTGG  CAGTGTAGGGTTTATTTGTTTGTGTGTGTGTTATGTCAGCAGAGTATGTGTCCTCCCAGCCAGCTGGTAG  CTCGTCAGCGGGTGGTCAGCAGCAAGAGAGTAGTGTGTCATCTCAAAGTGATCAGGCCAGTACATCGTCT  CAATTAGGAGCTGATAGCTCGTCAGCGGGTGGTCAGCAGCAAGAGAGTAGTGTGTCATCTCAAAGTGATC  AGGCCAGTACATCGTCTCAATTAGGAGCTGATAGCTCGTCAGCGGGTGGTCAGCAGCAAGAGAGTAGTGT  GTCATCTCAAAGTGGTCAGGCCAGTACATCGTCTCAATTAGGGACTGATTGGCGGCAAGAGATGCGCTCC  AAGGTTGCGAGTGTTGAGTACATGTTGGCTGCTCGTGCCCTTATTTCTGTAGGGGTCTATGCTGCTCAGG  GAGAGATCGCGAGATCGCGAGGGTGTGCTCCCCTGCGTGTTGCAGAAGTCGAAGAAATCGTGAGGGATGG  CCTTGTACGCAGCCACTTTCATGATAGTGGCCTTTCACTAGGCTCCATACGACTCGTGCTTATGCAGGTT  GGGGATAAGTTGGGGCTACAAGGTTTGAAGATTGGCGAAGGGTACGCCACCTATCTCGCGCAAGCGTTTG  CTGACAGCGTGGTGGTTGCGGCTGATGTTCAGAGTAGTGGTGCGTC | AGCTGGTAGCTCGTCAGCGGGTGGTCAGCAGCAAGAGAGTAGTGTGTCATCTCAAAGTGATCAGGCCAGTACATCGTCTCAATTAGG  AGCTGATAGCTCGTCAGCGGGTGGTCAGCAGCAAGAGAGTAGTGTGTCATCTCAAAGTGATCAGGCCAGTACATCGTCTCAATTAGG  AGCTGATAGCTCGTCAGCGGGTGGTCAGCAGCAAGAGAGTAGTGTGTCATCTCAAAGTGGTCAGGCCAGTACATCGTCTCAATTAGG | 222.  ADSSSASGQQQESSVSSQSGQASTSSQL  223.  ADSSSAGGQQQESSVSSQSDQASTSSQL  224.  ADSSSAGGQQQESSVSSQSGQASTSSQL | 3 |
| 57 | Egypt |  | MN311246 | >MN311246.1 Anaplasma marginale isolate 288 major surface protein 1a (msp1a) gene, partial cds  TGCGATTACACGTTTCCGTATGTTATAATCAGGCCGCCGGTGTGGTAGCGTGCTGGTTGTGTGGTTGTCC  TCTTTCCCGATGTTAGGTCGTTCGTTTTACGCCTCACAAGTTTGTACGCTGTGCCCCTGGCAGTGTAGGG  TTTGTTTATTTGTGTGTGTGTTATGTCAGAGTATGGGTCCTCTCAGCCAGCTGATAGCTCGTCAGCGAGT  GGTCAGCAGCAAGAGAGTAGTGTGTTATCTCAAAGTGGTCAGGCCAGTACATCGTCTCAATTAGGAACTG  ATAGCTCGTCAGCGAGTGGTCAGCAGCAAGAGAGTGGTGTGTCATCTCAAAGTGATCAGGCCAGTACATC  GTCTCAATTAGGAGCTGATAGCTCGTCAGCGAGTGGTCAGCAGCAAGAGAGTAGTGTGTTATCTCAAAGT  GGTCAGGCCAGTACATCGTCTCAATTAGGAGCTGATAGCTCGTCAGCGAGTGGTCAGCAGCAAGAGAGTG  GTGTGTTATCTCAAAGTGGTCAGGCCAGTACATCGTCTCAATCAGGAACTGATTGGCGGCAAGAGATGCG  CTCCAAGGTTGCGAGTGTTGAGTACATGTTGGCTGCTCGTGCCCTTATCTCTGTAGGGGTCTATGCTGCT  CAGGGAGAGATCGCGAGATCGCAAGGGTGTGCTCCCCTGTGTGTTGCAGAAGTCGAAGAAATCGTGAGGA  ATAGCCTTGTACGCAGCTACTTTCATGATAGTGGCCTTTCACTAGGCTCCATACGACTCGTGCTTATGC | CTGATAGCTCGTCAGCGAGTGGTCAGCAGCAAGAGAGTAGTGTGTTATCTCAAAGTGGTCAGGCCAGTACATCGTCTCAATTAGGAA  CTGATAGCTCGTCAGCGAGTGGTCAGCAGCAAGAGAGTGGTGTGTCATCTCAAAGTGATCAGGCCAGTACATCGTCTCAATTAGGAG  CTGATAGCTCGTCAGCGAGTGGTCAGCAGCAAGAGAGTAGTGTGTTATCTCAAAGTGGTCAGGCCAGTACATCGTCTCAATTAGGAG  CTGATAGCTCGTCAGCGAGTGGTCAGCAGCAAGAGAGTGGTGTGTTATCTCAAAGTGGTCAGGCCAGTACATCGTCTCAATCAGGAA | 225.  DSSSASGQQQESSVLSQSGQASTSSQLG  226.  DSSSASGQQQESGVSSQSDQASTSSQLG  227.  DSSSASGQQQESSVLSQSGQASTSSQLG  228.  DSSSASGQQQESGVLSQSGQASTSSQSG | 4 |
| 58 | Egypt |  | MN311245 | >MN311245.1 Anaplasma marginale isolate 291 major surface protein 1a (msp1a) gene, partial cds  TGCGATTACACGTTTCCGTATGTTATAATCAGGCCGCCGGTGTGGTAGCGTGCTGGTTGTGTGGTTGTCC  TCTTTCCCGATGTTAGGTCGTTCGTTTTACGCCTCACAAGTTTGTACGCTGTGCCCCTGGCAGTGTAGGG  TTTGTTTATTTGTGTGTGTGTTATGTCAGAGTATGGGTCCTCTCAGCCAGCTGATAGCTCGTCAGCGAGT  GGTCAGCAGCAAGAGAGTAGTGTGTTATCTCAAAGTGGTCAGGCCAGTACATCGTCTCAATTAGGAACTG  ATAGCTCGTCAGCGAGTGGTCAGCAGCAAGAGAGTGGTGTGTCATCTCAAAGTGATCAGGCCAGTACATC  GTCTCAATTAGGAGCTGATAGCTCGTCAGCGAGTGGTCAGCAGCAAGAGAGTAGTGTGTTATCTCAAAGT  GGTCAGGCCAGTACATCGTCTCAATTAGGAGCTGATAGCTCGTCAGCGAGTGGTCAGCAGCAAGAGAGTG  GTGTGTTATCTCAAAGTGGTCAGGCCAGTACATCGTCTCAATTAGGAGCTGATAGGCGTCAAGCGATGCG  CTCCAAGGTTGCGAGTGTTGAGTACATGTTGGCTGCTCGTGCCCTTATCTCTGTAGGAGTCTATGCTGGT  CAGGGAGAGATCGCGAGATCGCAAGGGTGTGCTCCCCTGTGTGTTGCAGAAGTCGAAGAAATCGTGAGGA  ATAGCCTTGTACGCAGCTACTTTCATGATAGTGGCCTTTCACTAGGCTCCATACGACTCGTGCTTATGC | AGCTGATAGCTCGTCAGCGAGTGGTCAGCAGCAAGAGAGTAGTGTGTTATCTCAAAGTGGTCAGGCCAGTACATCGTCTCAATTAGG  AACTGATAGCTCGTCAGCGAGTGGTCAGCAGCAAGAGAGTGGTGTGTCATCTCAAAGTGATCAGGCCAGTACATCGTCTCAATTAGG  AGCTGATAGCTCGTCAGCGAGTGGTCAGCAGCAAGAGAGTAGTGTGTTATCTCAAAGTGGTCAGGCCAGTACATCGTCTCAATTAGG  AGCTGATAGCTCGTCAGCGAGTGGTCAGCAGCAAGAGAGTGGTGTGTTATCTCAAAGTGGTCAGGCCAGTACATCGTCTCAATTAGG | 229.ADSSSASGQQQESSVLSQSGQASTSSQL  230.  TDSSSASGQQQESGVSSQSDQASTSSQL  231.  ADSSSASGQQQESSVLSQSGQASTSSQL  232.  ADSSSASGQQQESGVLSQSGQASTSSQL | 4 |
| 59 | Egypt |  | MN311244 | >MN311244.1 Anaplasma marginale isolate 269 major surface protein 1a (msp1a) gene, partial cds  TGCGATTACACGTTTCCGTATGTTATAATCAGGCCGCCGGTGTGGTAGCGTGCTGGTTGTGTGGTTGTCC  TCTTTCCCGATGTTAGGTCGTTCGTTTTACGCCTCACAAGTTTGTACGCTGTGCCCCTGGCAGTGTAGGG  TTTGTTTATTTGTGTGTGTGTTATGTCAGAGTATGGGTCCTCTCAGCCAGCTGATAGCTCGTCAGCGAGT  GGTCAGCAGCAAGAGAGTAGTGTGTTATCTCAAAGTGGTCAGGCCAGTACATCGTCTCAATTAGGAACTG  ATAGCTCGTCAGCGAGTGGTCAGCAGCAAGAGAGTAGTGTGTTATCTCAAAGTGATCAGGCCAGTACATC  GTCTCAATTAGGAGCTGATAGCTCGTCAGCGAGTGGTCAGCAGCAAGAGAGTAGTGTGTTATCTCAAAGT  GGTCAGGCCAGTACATCGTCTCAATTAGGGACTGATAGCTCGTCAGCGAGTGGTCAGCAGCAAGAGAGTG  GTGTGTTATCTCAAAGTGGTCAGGCCAGTACATCGTCTCAATCAGGAACTGATTGGCGGCAAGAGATGCG  CTCCAAGGTTGCGAGTGTTGAGTACATGTTGGCTGCTCGTGCCCTTATTTCTGTAGGGGTCTATGCTGCT  CAGGGAGAGATCGCGAGATCGCAAGGGTGTGCTCCCCTGTGTGTTGCAGAAGTCGAAGAAATCGTGAGGA  ATAGCCTTGTACGCAGCTACTTTCATGATAGTGGCCTTTCACTAGGCTCCATACGACTCGTGCTTATGC | CTGATAGCTCGTCAGCGAGTGGTCAGCAGCAAGAGAGTAGTGTGTTATCTCAAAGTGGTCAGGCCAGTACATCGTCTCAATTAGGAA  CTGATAGCTCGTCAGCGAGTGGTCAGCAGCAAGAGAGTAGTGTGTTATCTCAAAGTGATCAGGCCAGTACATCGTCTCAATTAGGAG  CTGATAGCTCGTCAGCGAGTGGTCAGCAGCAAGAGAGTAGTGTGTTATCTCAAAGTGGTCAGGCCAGTACATCGTCTCAATTAGGGA  CTGATAGCTCGTCAGCGAGTGGTCAGCAGCAAGAGAGTGGTGTGTTATCTCAAAGTGGTCAGGCCAGTACATCGTCTCAATCAGGAA | 233.  DSSSASGQQQESSVLSQSGQASTSSQLG  234.  DSSSASGQQQESSVLSQSDQASTSSQLG  235.  DSSSASGQQQESSVLSQSGQASTSSQLG  236.  DSSSASGQQQESGVLSQSGQASTSSQSG | 4 |
| 60 | Egypt |  | MN311243 | >MN311243.1 Anaplasma marginale isolate 268 major surface protein 1a (msp1a) gene, partial cds  TGCGATTACACGTTTCCGTATGTTATAATCAGGCCGCCGGTGTGGTAGCGTGCTGGTTGTGTGGTTGTCC  TCTTTCCCGATGTTAGGTCGTTCGTTTTACGCCTCACAAGTTTGTACGCTGTGCCCCTGGCAGTGTAGGG  TTTGTTTATTTGTGTGTGTGTTATGTCAGAGTATGGGTCCTCTCAGCCAGCTGATAGCTCGTCAGCGAGT  GGTCAGCAGCAAGAGAGTAGTGTGTTATCTCAAAGTGGTCAGGCCAGTACATCGTCTCAATTAGGAACTG  ATAGCTCGTCAGCGAGTGGTCAGCAGCAAGAGAGTAGTGTGTTATCTCAAAGTGGTCAGGCCAGTACATC  GTCTCAATTAGGAACTGATAGCTCGTCAGCGAGTGGTCAGCAGCAAGAGAGTAGTGTGTTATCTCAAAGT  GGTCAGGCCAGTACATCGTCTCAATTAGGAACTGATAGCTCGTCAGCGAGTGGTCAGCAGCAAGAGAGTA  GTGTGTTATCTCAAAGTGGTCAGGCCAGTACATCGTCTCAATCAGGAACTGATTGGCGGCAAGAGATGCG  CTCCAAGGTTGCGAGTGTTGAGTACATGTTGGCTGCTCGTGCCCTTATCTCTGTAGGGGTCTATGCTGCT  CAGGGAGAGATCGCGAGATCGCAAGGGTGTGCTCCCCTGTGTGTTGCAGAAGTCGAAGAAATCGTGAGGA  ATAGCCTTGTACGCAGCTACTTTCATGATAGTGGCCTTTCACTAGGCTCCATACGACTCGTGCTTATGC | CTGATAGCTCGTCAGCGAGTGGTCAGCAGCAAGAGAGTAGTGTGTTATCTCAAAGTGGTCAGGCCAGTACATCGTCTCAATTAGGAA  CTGATAGCTCGTCAGCGAGTGGTCAGCAGCAAGAGAGTAGTGTGTTATCTCAAAGTGGTCAGGCCAGTACATCGTCTCAATTAGGAA  CTGATAGCTCGTCAGCGAGTGGTCAGCAGCAAGAGAGTAGTGTGTTATCTCAAAGTGGTCAGGCCAGTACATCGTCTCAATTAGGAA  CTGATAGCTCGTCAGCGAGTGGTCAGCAGCAAGAGAGTAGTGTGTTATCTCAAAGTGGTCAGGCCAGTACATCGTCTCAATCAGGAA | 237.  DSSSASGQQQESSVLSQSGQASTSSQLG  238.  DSSSASGQQQESSVLSQSGQASTSSQLG  239.  DSSSASGQQQESSVLSQSGQASTSSQLG  240.  DSSSASGQQQESSVLSQSGQASTSSQSG | 4 |
| 61 | Egypt |  | MN273314 | >MN273314.1 Anaplasma marginale isolate 33 major surface protein 1a (msp1a) gene, partial cds  GCGATTACACGTTCCGTATGTTACAATCAGGCCGCCGGTGTGGTAGCGTGCTGGTTGTGTGGTTGTCCTC  TTTCCCGATGTTGGGTCGTTCGTTTTACGCCTCACAAGTTTGTACGCTGTGCCCCTGGCAGTGTAGGGTT  TGTTTGTTTGTGTGTGTGTTATGTCAGAGTTGGAGTCCTCTCAGCTATCTGGTAGCTCGTCAGCGAGTGG  TCAGCAGCAAGAGAGTAGTGTGTTATCTCAAGGTGGTCAGGCCAGTACATCGTCTCAATTAGGAACTGAT  AGCTCGTCAGCGAGTGGTCAGCAGCAAGAGAGTAGTGTGTCATCTCAAAGTGGTCAGGCCAGTACATCGT  CTCAATTAGGAACTGATAGCTCGTCAGCGAGTGGTCAGCAGCAAGAGAGTAGTGTGTCATCTCAAAGTGG  TCAGGCCAGTACATCGTCTCAATTAGGAACTGATAGCTCGTCAGCGAGTGGTCAGCAGCAAGAGAGTAGT  GTGTCATCTCAAAGTGGTCAGGCCAGTACATCGTCTCAATTAGGAGCTGATAGTCGTCAGCGATGTCGCA  GCAAGATTGCGAGTGTTGAGTACATTTTGGCTGCTCGTGCCCTTATCTCTGTAGGGGTCTATGCTGCTCA  GGAAGAGATCGCGAAATCGCTAGGGTATACTCCCCTGCGTGTTGCAGAAGTCGAAGCAATCGTGAGGGAT  AGCCTTGTACGCAGCCACTTTCATGATAGTGGCCTTTCACTAGGCTCCATACGACTCGTGCTTATGCGGG  TTGGGGATAAGTTGGGGCTACAAGATTCGAAGATTGGCGAAGGGTACGCCACCTATCTCGCGCAAGCGTT  TGCTGACAGCGTGGTGGTTGCGGCTGATGTTCAGAGTGGTGGTGCGCGCTCTGCT | TAGCTCGTCAGCGAGTGGTCAGCAGCAAGAGAGTAGTGTGTTATCTCAAGGTGGTCAGGCCAGTACATCGTCTCAATTAGGAACTGA  TAGCTCGTCAGCGAGTGGTCAGCAGCAAGAGAGTAGTGTGTCATCTCAAAGTGGTCAGGCCAGTACATCGTCTCAATTAGGAACTGA  TAGCTCGTCAGCGAGTGGTCAGCAGCAAGAGAGTAGTGTGTCATCTCAAAGTGGTCAGGCCAGTACATCGTCTCAATTAGGAACTGA  TAGCTCGTCAGCGAGTGGTCAGCAGCAAGAGAGTAGTGTGTCATCTCAAAGTGGTCAGGCCAGTACATCGTCTCAATTAGGAGCTGA | 241.  SSSASGQQQESSVLSQGGQASTSSQLGT  242.  SSSASGQQQESSVSSQSGQASTSSQLGT  243.  SSSASGQQQESSVSSQSGQASTSSQLGT  244.  SSSASGQQQESSVSSQSGQASTSSQLGA | 4 |
| 62 | Egypt |  | MN273313 | >MN273313.1 Anaplasma marginale isolate 214 major surface protein 1a (msp1a) gene, partial cds  ACACGTTCCGTATGTTACAATCAGGCCGCCGGTGTGGTAGCGTGCTGGTTGTGTGGTTGTCCTCTTTCCC  GATGTTGGGTCGTTCGTTTTACGTCTCACAAGTTTGTACGCTGTGCCCCTGGCAGTGTAGGGTTTGTTTA  TTTGTGTGTGTGTTATGTCAGCAGAGTGTGAGTCTTCCCAGCCAGCTGATAGCTCGTCAGCGAGTGGTCA  GCAGCAAGAGAGTGGTGTGCCATCTCAAAGTGGTCAGGCCAGTACATCGTCTCAATTAGGAGCTGATAGC  TCGTCAGCGAGTGGTCAGCAGCAAGAGAGTGGTGTGCCATCTCAAAGTGGTCAGGCCAGTACATCGTCTC  AATTAGGGACTGATAGCTCGTCAGCGAGTGGTCAGCAGCAAGAGAGTGGTGTGCCATCTCAAAGTGGTCA  GGCCAGTACATCGTCTCAATTAGGGACTGATAGCTCGTCAGCGAGTGGTCAGCAGCAAGAGAGTGGTGTG  CCATCTCAAAGTGGTCAGGCCAGTACATCGTCTCAATTAGGGAGTAGAGCTGATAGCTCGTCAGCGAGTG  GTCAGCAGCAAGAGAGTGGTGTGTCATCTCAAAGTGATGCCAGTACATCGTCTCAATTAGGAGCTGATTG  GCGGCAAGAGATGCGCTCCAAGGTTGCGAGTGTTGAGTACATGTTGGCTGCTCGTGCCCTTATCTCTGTA  GGGGTCTATGCTGCTCAGGGAGAGATCGCGAGATCGCTAGGGTATGCTCCCCTGCGTGTTGCAGAAGTCG  AAGAAATCGTGAGGGATAGCCTTGTACGCAGCTACTTTCATGATAGTGGCCTTTCACTAGGCTCCATACG  ACTCGTGCTTATGCAGGTTGGGGATAAGTTGGGGCTACAAGATTCGAAGATTAGCGAAGGGTACGCCACC  TATCTCGCGCAAGCGTTTGCTGACAGCGTGGTGGTTGCGGCTGATGT | AGCTGATAGCTCGTCAGCGAGTGGTCAGCAGCAAGAGAGTGGTGTGCCATCTCAAAGTGGTCAGGCCAGTACATCGTCTCAATTAGG  AGCTGATAGCTCGTCAGCGAGTGGTCAGCAGCAAGAGAGTGGTGTGCCATCTCAAAGTGGTCAGGCCAGTACATCGTCTCAATTAGG  GACTGATAGCTCGTCAGCGAGTGGTCAGCAGCAAGAGAGTGGTGTGCCATCTCAAAGTGGTCAGGCCAGTACATCGTCTCAATTAGG  GACTGATAGCTCGTCAGCGAGTGGTCAGCAGCAAGAGAGTGGTGTGCCATCTCAAAGTGGTCAGGCCAGTACATCGTCTCAATTAGGGAGTAG  AGCTGATAGCTCGTCAGCGAGTGGTCAGCAGCAAGAGAGTGGTGTGTCATCTCAAAGT-G--ATGCCAGTACATCGTCTCAATTAGG | 245.  ADSSSASGQQQESGVPSQSGQASTSSQL  246.  ADSSSASGQQQESGVPSQSGQASTSSQL  247.  TDSSSASGQQQESGVPSQSGQASTSSQL  248.  TDSSSASGQQQESGVPSQSGQASTSSQLGS  249.  ADSSSASGQQQESGVSSQSDASTSSQL | 5 |
| 63 | Egypt |  | MN273312 | >MN273312.1 Anaplasma marginale isolate 59 major surface protein 1a (msp1a) gene, partial cds  TGCAATTACACGTTCCGTATGTTACAATCAGGCCGCCGGTGTGGTAGCGTGCTGGTTGTGTGGTTGTCCT  CTTTCCCGATGTTGGGTCGTTCGTTTTACGTCTCACAAGTTTGTACGCTGTGCCCCTGGCAGTGTAGGGT  TTGTTTATTTGTGTGTGTGTTATGTCAGAGTGTGAGTCTTCCCAGCCAGCTGATAGCTCGTCAGCGAGTG  GTCAGCAGCAAGAGAGTGGTGTGTCATCTCAAAGTGATCAGGCCAGTACATCGTCTCAATTAGGAGCTGA  TAGCTCGTCAGCGAGTGGTCAGCAGCAAGAGAGTGGTGTGCCATCTCAAAGTGGTCAGGCCAGTACATCG  TCTCAATTAGGGACTGATAGCTCGTCAGCGAGTGGTCAGCAGCAAGAGAGTGGTGTGTCATCTCAAAGTG  ATCAGGCCAGTACATCGTCTCAATTAGGAGCTAATTGGCGGCAAGAGATGCGCTCCAAGGTTGCGAGTGT  TGAGTACATGTTGGCTGCTCGTGCCCTTATCTCTGTAGGGGTCTATGCTGCTCAGGGAGAGATCGCGAGA  TCGCTAGGGTATGCTCCCCTGCGTGTTGCAGAAGTCGAAGAAATCGTGAGGGATAGCCTTGTACGCAGCT  ACTTTCATGATAGTGGCCTTTCACTAGGCTCCATACGACTCGTGCTTATGCAGGTTGGGGATAAGTTGGG  GCTACAAGATTCGAAGATTAGCGAAGGGTACGCCACCTATCTCGCGCAAGCGTTTGCTGACAGCGTGGTG  GTTGCGGCTGATGTTCAGAGTGGTGGTGCGCGCCCTGCT | AGCTGATAGCTCGTCAGCGAGTGGTCAGCAGCAAGAGAGTGGTGTGTCATCTCAAAGTGATCAGGCCAGTACATCGTCTCAATTAGG  AGCTGATAGCTCGTCAGCGAGTGGTCAGCAGCAAGAGAGTGGTGTGCCATCTCAAAGTGGTCAGGCCAGTACATCGTCTCAATTAGG  GACTGATAGCTCGTCAGCGAGTGGTCAGCAGCAAGAGAGTGGTGTGTCATCTCAAAGTGATCAGGCCAGTACATCGTCTCAATTAGG | 250.  ADSSSASGQQQESGVSSQSDQASTSSQL  251.  ADSSSASGQQQESGVPSQSGQASTSSQL  252.  TDSSSASGQQQESGVSSQSDQASTSSQL | 3 |
| 64 | Egypt | ElMinia | MN257057 | >MN257057.1 Anaplasma marginale isolate 45 major surface protein 1a (msp1a) gene, partial cds  ATTACACGTTTCCGTATGTTACAATCGGGCTGCCGGTGTGTAGCGTGCTGGTTGTGTGGTTGTCCTCTTT  CCCGATGTTGGGTCGTTCGTTTTACGTCTCACAAGTTTGTACGCTGTGCCCCTGGCAGTGTAGGGTTTGT  TTGTGTGTGTGTGTGTTATGTCAGAGTGTGAGTCTTCCCGGCCAGCTGATAGCTCGTCAGCGAGTGGTCA  GCAGCAAGAGAGTGGTGTGTCATCTCAAAGTGATGCCAGTACATCGTCTCAATTAGGGGCTGATAGCTCG  TCAGCGAGTGGTCAGCAGCAAGAGAGTAGTGTGCCATCTCAAAGTGGTGCCAGTACATCGTCTCAATTAG  GGGCTGATAGCTCGTCAGCGAGTGGTCAGCAGCAAGAGAGTGGTGTGCCATCTCAAAGTGGTGCCAGTAC  ATCGTCTCAATTAGGAGCTGATAGCTCGTCAGCGAGTGGTCAGCAGCAAGAGAGTGGTGTGCCATCTCAA  AGTGGTGCCAGTACATCGTCTCAATTAGGAGCTGATAGCTCGTCAGCGAGTGGTCAGCAGCAAGAGAGTG  GTGTGCCATCTCAAAGTGGTGCCAGTACATCGTCTCAATTAGGGACTGATAGCTCGTCAGCGAGTGGTCA  GCAGCAAGAGAGTGGTGTGTCATCTCAAAGTGATGCCAGTACATCGTCTCAATTAGGAGCTGATTGGCGG  CAAGAGATGCGCTCCAAGGTTGCGAGTGTTGAGTACATGTTGGCTGCTCGTGCCCTTATCTCTGTAGGGG  TCTATGCTGCTCAGGGAGAGATCGCGAGATCGCTAGGGTATGCTCCCCTGCGTGTTGCAGAAGTCGAAGA  AATCGTGAGGGATAGCCTTGTACGCAGCTACTTTCATGATAGTGGCCTTTCACTAGGCTCCATA | AGCTGATAGCTCGTCAGCGAGTGGTCAGCAGCAAGAGAGTGGTGTGTCATCTCAAAGTGATGCCAGTACATCGTCTCAATTAGG  GGCTGATAGCTCGTCAGCGAGTGGTCAGCAGCAAGAGAGTAGTGTGCCATCTCAAAGTGGTGCCAGTACATCGTCTCAATTAGG  GGCTGATAGCTCGTCAGCGAGTGGTCAGCAGCAAGAGAGTGGTGTGCCATCTCAAAGTGGTGCCAGTACATCGTCTCAATTAGG  AGCTGATAGCTCGTCAGCGAGTGGTCAGCAGCAAGAGAGTGGTGTGCCATCTCAAAGTGGTGCCAGTACATCGTCTCAATTAGG  AGCTGATAGCTCGTCAGCGAGTGGTCAGCAGCAAGAGAGTGGTGTGCCATCTCAAAGTGGTGCCAGTACATCGTCTCAATTAGG  -GACTGATAGCTCGTCAGCGAGTGGTCAGCAGCAAGAGAGTGGTGTGTCATCTCAAAGTGATGCCAGTACATCGTCTCAATTAGG | 253.  ADSSSASGQQQESGVSSQSDASTSSQL  254.  ADSSSASGQQQESSVPSQSGASTSSQL  255.  ADSSSASGQQQESGVPSQSGASTSSQL  256.  ADSSSASGQQQESGVPSQSGASTSSQL  257.  ADSSSASGQQQESGVPSQSGASTSSQL  258.  TDSSSASGQQQESGVSSQSDASTSSQL | 6 |
| 65 | Egypt | ElMinia | MN257056 | >MN257056.1 Anaplasma marginale isolate 44 major surface protein 1a (msp1a) gene, partial cds  AAATTTAGTATATTAATCTTGCGATTACACGTTCCGTATGTTACAATCGGGCTGCCGGTGTGGTAGCGTG  CTGGTTGTGTGGTTGTCCTCTTTCCCGATGTTGGGTCGTTCGTTTTACGTCTCACAAGTTTGTACGCTGT  GCCCCTGGCAGTGTAGGGTTTGTTTGTGTGTGTGTGTGTTATGTCAGAGTTGGTCTTCCTTCAGCCAGCT  GATAGCTCGTCAGCGAGTGGTCAGCAGCAAGAGAGTGGTGTGTCATCTCAAAGTGATCAGGCCAGTACAT  CGTCTCAATTAGGAGCTGATAGCTCGTCAGCGAGTGGTCAGCAGCAAGAGAGTGGTGTGTCATCTCAAAG  TAGTCATGCCAGTACATCGTCTCAATTAGGAGCTGATAGCTCGTCAGCGAGTGGTCAGCAGCAAGAGAGT  GGTGTGCCATCTCAAAGTGATCAGGCCAGTACATCGTCTCAATTAGGAGCTGATAGCTCGTCAGCGAGTG  GTCAGCAGCAAGAGAGTGGTGTGTCATCTCAAAGTGATCAGGCCAGTACATCGTCTCAATTAGGAGCTGA  TTGGCGGCAAGAGATGCGCTCCAAGGTTGCGAGTGTTGAGTACATGTTGGCTGCTCGTGCCCTTATCTCT  GTAGGGGTCTATGCTGCTCAGGGAGAGATCGCGAGATCGCTAGGGTATGCTCCCCTGCGTGTTGCAGAAG  TCGAAGAAATCGTGAGGGATAGCCTTGTACGCAGCTACTTTCATGATAGTGGCCTTTCACTAGGCTCCAT  ACGACTCGTGCTTATGCAGGTTGGGGATAAGTTGGGGCTACAAGATTCGAAGATTAGCGAAGGGTACGCC  ACCTATCTCGCGCAAGCGTTTGCTGACAGCGTGGTGGTTGCGGCTGATGTTCAGAGTGGTGGTGCGCGCC  CTGCT | AGCTGATAGCTCGTCAGCGAGTGGTCAGCAGCAAGAGAGTGGTGTGTCATCTCAAAGTGATCAGGCCAGTACATCGTCTCAATTAGG  AGCTGATAGCTCGTCAGCGAGTGGTCAGCAGCAAGAGAGTGGTGTGTCATCTCAAAGT-AGTCATGCCAGTACATCGTCTCAATTAGG  AGCTGATAGCTCGTCAGCGAGTGGTCAGCAGCAAGAGAGTGGTGTGCCATCTCAAAGTGATCAGGCCAGTACATCGTCTCAATTAGG  AGCTGATAGCTCGTCAGCGAGTGGTCAGCAGCAAGAGAGTGGTGTGTCATCTCAAAGTGATCAGGCCAGTACATCGTCTCAATTAGG | 259.  ADSSSASGQQQESGVSSQSDQASTSSQL  260.  ADSSSASGQQQESGVSSQSSHASTSSQL  261.  ADSSSASGQQQESGVPSQSDQASTSSQL  262.  ADSSSASGQQQESGVSSQSDQASTSSQL | 4 |
| 66 | Egypt | ElMinia | MN257055 | >MN257055.1 Anaplasma marginale isolate 43 major surface protein 1a (msp1a) gene, partial cds  CTTGCGATCACACGTTCCGTATGTTACAATCGGGCTGCCGGTGTGGTAGCGTGCTGGTTGTGTGGTTGTC  CTCTTTCCCGATGTTGGGTCGTTCGTTTTACGTCGCACAAGTTTGTACGCTGTGCCCCTGGCAGTGTAGG  GTTTATTTGTTTGTGTGTGTGTTATGTCAGCAGAGTGTGAGTCCTCCCAGCCAGCTGGTAGCTCGTCAGC  GAGTGGTCAGCAGCAAGAGAGTGGTGTGTCATCTCAAAGTGAGCAGGCCAGTACATCGTCTCAATTAGGA  GCTGATAGCTCGTCAGCGAGTGGTCAGCAGCAAGAGAGTGGTGTGTCATCTCAAAGTGATCAGGCCAGTA  CATCGTCTCAATTAGGAGCTGATAGCTCGTCAGCGAGTGGTCAGCAGCAAGAGAGTGGTGTGCCATCTCA  AAGTGGTCAGGCCAGTACATCGTCTCAATTAGGGACTGATAGCTCGTCAGCGAGTGGTCAGCAGCAAGAG  AGTGGTGTGTCATCTCAAAGTGATCAGGCCAGTACATCGTCTCAATTAGGGACTGATTGGCGGCAAGAGA  TGCGCTCCAAGGTTGCGAGTGTTGAGTACATGTTGGCTGCTCGTGCCCTTATTTCTGTAGGGGTCTATGC  TGCTCAGCGAGTGGTCGCGCGATCGCGAGGGTGTGCTCCCCTGCGTGTTGCAGAAGTCGAAGAAATCGTG  AGGGATGGCCTTGTACGCAGCCACTTTCATGATAGTGGCCTTTCACTAGGCTCCATACGACTCGTGCTTA  TGCAGGTTGGGGATAAGTTGGGGCTACAAGGTTTGAAGATTGGCGAAGGGTACGCCACCTATCTCGCGCA  AGCGTTTGCTGACAGCGTGGTGGTTGCGGCTGATGTTCAGAGTAGTAGTGCGTGCCCTGC | AGCTGGTAGCTCGTCAGCGAGTGGTCAGCAGCAAGAGAGTGGTGTGTCATCTCAAAGTGAGCAGGCCAGTACATCGTCTCAATTAGG  AGCTGATAGCTCGTCAGCGAGTGGTCAGCAGCAAGAGAGTGGTGTGTCATCTCAAAGTGATCAGGCCAGTACATCGTCTCAATTAGG  AGCTGATAGCTCGTCAGCGAGTGGTCAGCAGCAAGAGAGTGGTGTGCCATCTCAAAGTGGTCAGGCCAGTACATCGTCTCAATTAGG  -GACTGATAGCTCGTCAGCGAGTGGTCAGCAGCAAGAGAGTGGTGTGTCATCTCAAAGTGATCAGGCCAGTACATCGTCTCAATTAGG | 263.  AGSSSASGQQQESGVSSQSEQASTSSQL  264.  ADSSSASGQQQESGVSSQSDQASTSSQL  265.  ADSSSASGQQQESGVPSQSGQASTSSQL  266.  TDSSSASGQQQESGVSSQSDQASTSSQL | 4 |
| 67 | MW486117.1 | Grenada | St. Andrew Parish | >MW486117.1 Anaplasma marginale isolate GNDAPT4 major surface protein 1a (msp1a) gene, partial cds  TCAGAGAATGATCGCGCAATCGCAAGGGTGTGCTTCCCTGAGTGTTACAGAAGTCGAAGAAATCGTGAGG  GATGGCCTTGTACGCAGCCACTTTCATGATAGTGGCCTTTCACTAGGCTCCATACGACTCGTGCTTATGC  AGGTTGGGGATAAGTTGGGGCTACAAGATTTGAAGATTGGCGAAGGGTACGCCACCTATCTCGCGCAAGC  GTTTGCTGACAGCGTGGTGGTTTCGGCTGATGTTCAGAGTGGTGGTGGGCGCTCTGCTGCCGGCCTTGAC  AAGGCGATCGCAGACGTTGAGACGTCGTGGTCCCTGCACGGCGGCCTGATAAGCAAAGATTTTGACCGTG  GTACCAAAGTAGGAAGGGGCGACCTTGAGGCTTTTGTCGACTTCATGTTTGGCGGTGTGTCGTACAATGA  TAAGAACGCGGCTGTGGCTAGGAGCGTATTGGAAACGCTTGCCGGGCACGTCGATGCACTTGGTATATCG  TACAATCAGCTGGATAAGCTTGATGCTGACACTTTGTATAGTGTCGTATCGTTTAGTGGCGCTTCCGCAA  TAGACAGA | N/A | N/A | N/A |
| 68 | MW486118.1 | Grenada | St. Andrew Parish | >MW486118.1 Anaplasma marginale isolate GNDTJ7 major surface protein 1a (msp1a) gene, partial cds  GCTCCCCAATTGTTAAAATTTAGTATATTAATTCTTGCGATTACACGTTCCGTATGTTACAATCAGGCCG  TCGGTGTGGTAGCGTGCTGGTTGTGTGGTTGTCCTCTTTCCCGATGTTGGGTCGTTCGTTTTACGTCTCA  CAAGTTTGTACGCTGTGCCCCTGGCAGTGTAGGGTTTGTTTGTGTGTGTGTTATGTCAGAATATGTGTCC  AGCCAGTCAGCTGATAGCTCGTCAGCGAGTGGTCACCATCAAGAGAGTAGTGTGTTATCTCAAAGTGAAG  CCAGTACATCGTCTCAATTAAGGACTGATAGCTCGTCAGCGAGTGA | N/A | N/A | N/A |
| 69 | EF053268.1 | Mexico | Chiapas | >EF053268.1 Anaplasma marginale strain Santa Martha major surface protein 1a gene, partial cds  ATGTCAGAGTATGTGTCCAGCCAGCCAGCTGATAGCTCGTCAGCGAGTGGTGTGTTATCTCAAAGTGGTC  AGGCCAGTACATCGTCTCAATTAGGAACTGATAGCTCGTCAGCGGGTGATCAGCAGCAAGGGAGTGGTGT  GTCATCTCAAAGTGGTCAGGCCAGTACATCGTCTCAATTAGGAACTGATAGCTCGTCAGCGGGTGATCAG  CAGCAAGGGAGTGGTGTGTCATCTCAAAGTGGTCAGGCCAGTACATCGTCTCAATTAGGAACTGATAGCT  CGTCAGCGAGTGGTCAGCAGCAAGAGAGTAGTGTGTCATCTCAAAGTGATGCCAGTACATCGTCTCAATT  AGGAACTGATAGCTCGTCAGCGGGTGATCAGCAGCAAGGGAGTGGTGTGTCATCTCAAAGTGGTCAGGCC  AGTACATCGTCTCAATTAGGGACTGCTGATTGGCGGCAAGAGGTGCACTCCAAGGTTGCGAGTGTTGAG | GAGTGGTGTGTTATCTCAAAGTGGTCAGGCCAGTACATCGTCTCAATTAGGAACTGATAGCTCGTCAGCGGGTGATCAGCAGCAAGG  GAGTGGTGTGTCATCTCAAAGTGGTCAGGCCAGTACATCGTCTCAATTAGGAACTGATAGCTCGTCAGCGGGTGATCAGCAGCAAGG  GAGTGGTGTGTCATCTCAAAGTGGTCAGGCCAGTACATCGTCTCAATTAGGAACTGATAGCTCGTCAGCGAGTGGTCAGCAGCAAGA  GAGTAGTGTGTCATCTCAAAGT-G--ATGCCAGTACATCGTCTCAATTAGGAACTGATAGCTCGTCAGCGGGTGATCAGCAGCAAGG  GAGTGGTGTGTCATCTCAAAGTGGTCAGGCCAGTACATCGTCTCAATTAGGGACTG | 267.  SGVLSQSGQASTSSQLGTDSSSAGDQQQ  268.  SGVLSQSGQASTSSQLGTDSSSAGDQQQ  269.  SGVLSQSGQASTSSQLGTDSSSAGDQQQ  270.  SSVSSQSDASTSSQLGTDSSSAGDQQQ  271.  SGVSSQSGQASTSSQLGT | 5 |
| 71 | JN564635.1 | Mexico | Chiapas | >JN564635.1 Anaplasma marginale isolate Palenque Mex-07-065-01 major surface protein 1a (msp1a) gene, partial cds  ATGTCAGAGTATGTGTCCAGCCCGCCAGCTGATAGCTCGTCAGCGAGTGGTGTGTTATCTCAAAGTGGTC  AGGCCAGTACATCGTCTCAATTAGGAACTGATAGCTCGTCAGCGGGTGATCAGCAGCAAGGGAGTGGTGT  GTCATCTCAAAGTGGTCAGGCCAGTACATCGTCTCAATTAGGAACTGATAGCTCGTCAGCGGGTGATCAG  CAGCAAGGGAGTGGTGTGTCATCTCAAAGTGGTCAGGCCAGTACATCGTCTCAATTAGGAACTGATAGCT  CGTCAGCGGGTGATCAGCAGCAAGGGAGTGGTGTGTCATCTCAAAGTGGTCAGGCCAGTACATCGTCTCA  ATTAGGGACTGATAGCTCGTCAGCGAGTGGTCAGCAGCAAGAGAGTAGTGTGTCATCTCAAAGTGATGCC  AGTACATCGTCTCAATTAGGAGCTGATTGGCGGCAAGAGGTGCACTCCAAGGTTGCGAGTGTTGAG | GAGTGGTGTGTTATCTCAAAGTGGTCAGGCCAGTACATCGTCTCAATTAGGAACTGATAGCTCGTCAGCGGGTGATCAGCAGCAAGG  GAGTGGTGTGTCATCTCAAAGTGGTCAGGCCAGTACATCGTCTCAATTAGGAACTGATAGCTCGTCAGCGGGTGATCAGCAGCAAGG  GAGTGGTGTGTCATCTCAAAGTGGTCAGGCCAGTACATCGTCTCAATTAGGAACTGATAGCTCGTCAGCGGGTGATCAGCAGCAAGG  GAGTGGTGTGTCATCTCAAAGTGGTCAGGCCAGTACATCGTCTCAATTAGGGACTGATAGCTCGTCAGCGAGTGGTCAGCAGCAAGA  GAGTAGTGTGTCATCTCAAAGT-G--ATGCCAGTACATCGTCTCAATTAGGAGCTGAT | 275.  SGVLSQSGQASTSSQLGTDSSSAGDQQQ  276.  SGVSSQSGQASTSSQLGTDSSSAGDQQQ  277.  SGVSSQSGQASTSSQLGTDSSSAGDQQQ  278.  SGVSSQSGQASTSSQLGTDSSSASGQQQ  279.  SSVSSQSDASTSSQLGAD | 5 |
| 72 | AF345870.1 | Mexico | Veracruz | >AF345870.1 Anaplasma marginale major surface protein 1a gene, partial cds  TGATGTTTACGGCTGTAGTATTGTGCTTATGGCAGACATTTCCATATACTGTGCAGTACGGTTGTGCTCC  CCAATTGTTAAAATTTAGTATATTAATCTTGCGATTACACGTTCCGTATGTTACAATCGGGCTGCCGGTG  TGGTAGCGTGCTGGTTGTGTGGTTGTCCTCTTTCCCGATGTTGGGTCGTTCGTTTTACGTCTCACAAGTT  TGTACGCTGTGCCCCTGGCAGTGTAGGGTTTGTTTGTGTGTGTGTGTGTTATGTCAGAGTATGTGTCCAG  CCAGCCAGCTGATAGCTCGTCAGCGAGTGGTGTGTTATCTCAAAGTGGTCAGGCCAGTACATCGTCTCAA  TTAGGAACTGATAGCTCGTCAGCGGGTGATCAGCAGCAAGGGAGTGGTGTGTCATCTCAAAGTGGTCAGG  CCAGTACATCGTCTCAATTAGGAACTGATAGCTCGTCAGCGGGTGATCAGCAGCAAGGGAGTGGTGTGTC  ATCTCAAAGTGGTCAGGCCAGTACATCGTCTCAATTAGGGACTGATAGCTCGTCAGCGAGTGGTCAGCAG  CAAGAGAGTAGTGTGTCATCTCAAAGTGATGCCAGTACATCGTCTCAATTAGGAGCTGATTGGCGGCAAG  AGGTGCACTCCAAGGTTGCGAGTGTTGAGTACATTTTGGCTGCTCGTGCCCTTATCTCTGTAGGGGTCTA  TGCTGCTCAGAGAATGATCGCGCAATCGCGAGGGTGTGCTTCCCTGAGT | GAGTGGTGTGTTATCTCAAAGTGGTCAGGCCAGTACATCGTCTCAATTAGGAACTGATAGCTCGTCAGCGGGTGATCAGCAGCAAGG  GAGTGGTGTGTCATCTCAAAGTGGTCAGGCCAGTACATCGTCTCAATTAGGAACTGATAGCTCGTCAGCGGGTGATCAGCAGCAAGG  GAGTGGTGTGTCATCTCAAAGTGGTCAGGCCAGTACATCGTCTCAATTAGGGACTGATAGCTCGTCAGCGAGTGGTCAGCAGCAAGA  GAGTAGTGTGTCATCTCAAAGT-G--ATGCCAGTACATCGTCTCAATTAGGAGCTGAT | 280.  SGVLSQSGQASTSSQLGTDSSSAGDQQQ  281.  SGVSSQSGQASTSSQLGTDSSSAGDQQQ  282.  SGVSSQSGQASTSSQLGTDSSSASGQQQ  283.  SSVSSQSDASTSSQLGAD | 4 |
| 73 | JN564642.1 | Mexico | Hidalgo | >JN564642.1 Anaplasma marginale isolate Atitalaquia Mex-14-010-01 major surface protein 1a (msp1a) gene, partial cds  GTGCTTATGGCAGACATTTCCATATACTGTGCAGTACGGTTGTGCTCCCCAATTGTTAAAATTTAGTATA  TTAATCTTGCGATTACACGTTCCGTATGTTACAATCGGGCTGCCGGTGTGGTAGCGTGCTGGTTGTGTGG  TTGTCCTCTTTCCCGATGTTGGGTCGTTCGTTTTACGTCTCACAAGTTTGTACGCTGTGCCCCTGGCAGT  GTAGGGTTTGTTTGTGTGTGTGTGTGTTATGTCAGAGTTGTCCCTTCAGCCAACTGATAGCTCGTCAGCG  AGTGGTCAGCAGCAAGAGAGTAGTGTGTTATCTCCAAGTGGTCAGGCCAGTACATCGTCTCAATTAGGAG  CTGATAGCTCGTCAGCGAGTGGTCAGCAGCAAGAGAGTAGTGTGTTATCTCCAAGTGGTCAGGCCAGTAC  ATCGTCTCAATCAGGAACTGATAGCTCGTCAGCGAGTGGTCAGCAGCAAGAGAGTAGTGTGTTATCTCAA  AGTGATCAGGCCAGTACATCGTCTCAATTAGGAACTGATAGCTCGTCAGCGAGTGGTCAGCAGCAAGAGA  GTAGTGTGTTATCTCAAAGTGATCAGGCCAGTACATCGTCTCAATCAGGAACTGATTGGCGGCAAGAGAT  GCGCTCCAAGGTTGCGAGTGTTGAG | AACTGATAGCTCGTCAGCGAGTGGTCAGCAGCAAGAGAGTAGTGTGTTATCTCCAAGTGGTCAGGCCAGTACATCGTCTCAATTAGG  AGCTGATAGCTCGTCAGCGAGTGGTCAGCAGCAAGAGAGTAGTGTGTTATCTCCAAGTGGTCAGGCCAGTACATCGTCTCAATCAGG  AACTGATAGCTCGTCAGCGAGTGGTCAGCAGCAAGAGAGTAGTGTGTTATCTCAAAGTGATCAGGCCAGTACATCGTCTCAATTAGG  AACTGATAGCTCGTCAGCGAGTGGTCAGCAGCAAGAGAGTAGTGTGTTATCTCAAAGTGATCAGGCCAGTACATCGTCTCAATCAGG  AACTGAT | 284.  TDSSSASGQQQESSVLSPSGQASTSSQL  285.  ADSSSASGQQQESSVLSPSGQASTSSQS  286.  TDSSSASGQQQESSVLSQSDQASTSSQL  287.  TDSSSASGQQQESSVLSQSDQASTSSQS  288.  TD | 4 |
| 74 | KC181915.1 | Philippines | Iloilo | >KC181915.1 Anaplasma marginale isolate Iloilo_218_23d Msp1a gene, partial cds  ATGTCAGAGTTGTCCCTTCAGTCAACTGATAGCTCGTCAGCGAGTGGTCAGCAGCAAGAGAGTAGTGTGT  TATCTCAAAGTGATCAGGCCAGTACATCGTCTCAATTAGGAGCTGATAGCTCGTCAGCGAGTGGTCAGCA  GCAAGAGAGTAGTGTGTTATCTCAAAGTGATCAGGCCAGTACATCGTCTCAATTAGGAGCTGATAGCTCG  TCAGCGAGTGGTCAGCAGCAAGAGAGTAGTGTGTTATCTCAAAGTGATCAGGCCAGTACATCGTCTCAAT  TAGGGACTGATTGGCGGCAAGAGATGCGCCCCAAGGTTGCGAGTGTTGAGTACATTTTGGCTGCTCGTGC  CCTTATCTCTGTAGGGGTCTATGCTGCTCAGGAAGAGATCGCGAGGTCGCTAGGGTATGCTCCCCTGCGT  GTTGCAGAAGTCGAAGCAATCGTGAGGGATAGCCTTGTACGCAGCCACTTTCATGATAGTGGCCTTTCAC  TAGGCTCCATACGACTCGTGCTTATGCAGGTTGGGGATAAGTTGGGGCTACAAGGTTT | 1.CTGATAGCTCGTCAGCGAGTGGTCAGCAGCAAGAGAGTAGTGTGTTATCTCAAAGTGATCAGGCCAGTACATCGTCTCAATTAGGAG  2.CTGATAGCTCGTCAGCGAGTGGTCAGCAGCAAGAGAGTAGTGTGTTATCTCAAAGTGATCAGGCCAGTACATCGTCTCAATTAGGAG  3.CTGATAGCTCGTCAGCGAGTGGTCAGCAGCAAGAGAGTAGTGTGTTATCTCAAAGTGATCAGGCCAGTACATCGTCTCAATTAGG | 289.  DSSSASGQQQESSVLSQSDQASTSSQLG  290.  DSSSASGQQQESSVLSQSDQASTSSQLG  291.  DSSSASGQQQESSVLSQSDQASTSSQL | 3 |
| 75 | KC181914.1 | Philippines | Iloilo | >KC181914.1 Anaplasma marginale isolate Iloilo_180_22a Msp1a gene, partial cds  ATGTCAGAGTTGTCCCTTCAGTCAACTGATAGCTCGTCAGCGAGTGGTCAGCAGCAAGAGAGTAGTGTGT  TACCTCAAAGTGGTCAGGCCAGTACATCGTCTCAATTAGGAACTGATAGCTCGTCAGCGAGTGGTCAGCA  GCAAGAGAGTAGTGTGTCATCTCAAAGTGGTCAGGCCAGTACATCGTCTCAATTAGGGACTGATTGGCGG  CAAGAGGTGCGCTCCAAGGTTGCGAGTGTTGAGTACAGTTTGGCTGCTCGTGCCCTTACCTCTGTAGGGG  CCTATGCTGCTCAGGAAGAGATCGCGAAATCGCTAGGGTATACTCCCCTGCGTGTTGCAGAAGTCGAAGA  AATCGTGAGGGATGGCCTTGTACGCAGCCACTTTCATTATAGTGGCCTTTCACTAGGCTCCATACGACTC  GTGCTTATGCAGGTTGGGGATAAGTTGGGGCTACAAGGTTT | 1.AACTGATAGCTCGTCAGCGAGTGGTCAGCAGCAAGAGAGTAGTGTGTTACCTCAAAGTGGTCAGGCCAGTACATCGTCTCAATTAGG  2.AACTGATAGCTCGTCAGCGAGTGGTCAGCAGCAAGAGAGTAGTGTGTCATCTCAAAGTGGTCAGGCCAGTACATCGTCTCAATTAGG  GACTGAT | 292.  TDSSSASGQQQESSVLPQSGQASTSSQL  293.  TDSSSASGQQQESSVSSQSGQASTSSQL  294.  GTD | 2 |
| 76 | KC181913.1 | Philippines | Negros Oriental | >KC181913.1 Anaplasma marginale isolate Negros_Oriental_273_21a Msp1a gene, partial cds  ATGTCAGAGTTGTCCCTTCAGTCAACTGATAGCTCGTCAGCGAGTGGTCAGCAGCAAGAGAGTAGTGTGT  TATCTCAAAGTGATCAGGCCAGTACATTGTCTCAATTAGGAGCTGATAGCTCGTCAGCGAGTGGTCAGCA  GCAAGAGAGTAGTGTGTTATCTCAAAGTGGGGCCAGTACATCGTCTCAATTAGGGACTAATTGGCGGCAA  GAGATGCGCTCCAAGGTTGCGAGTGTTGAGTACATTTTGGCTGCTCGTGCCCTTATCTCTGTAGGGGTCT  ATGCTGCTCAGGAAGCGATCGCGAGATCGCTAGGGTATGCTCCCCTGCGTGTTGCAGAAGTCGAAGAAAT  CGTGAGGGATGGCCTTGTACGCAGCTACTTTCATGATAGTGGCCTTTCACTAGGCTCCATACGACTCGTG  CTTATGCAGGTTGGGGATAAGTTGGGGCTACAAGGTTT | 1.CTGATAGCTCGTCAGCGAGTGGTCAGCAGCAAGAGAGTAGTGTGTTATCTCAAAGTGATCAGGCCAGTACATTGTCTCAATTAGGAG  2.CTGATAGCTCGTCAGCGAGTGGTCAGCAGCAAGAGAGTAGTGTGTTATCTCAAAGTGGGGCCAGTACATCGTCTCAATTAGG | 295.  DSSSASGQQQESSVLSQSDQASTLSQLG  296.  DSSSASGQQQESSVLSQSGASTSSQL | 2 |
| 77 | KC181912.1 | Philippines | Negros Occidental | >KC181912.1 Anaplasma marginale isolate Negros_Occidental_91_19c Msp1a gene, partial cds  ATGTCAGAGTTGTCCCTTCAGTCAACTGATAGCTCGTCAGCGAGTGGTCAGCGGCAAGAGAGTAGTGTGT  TATCTCAAAGTGATCAGGCCAGTACATTGTCTCAATTAGGAGCTGATAGCTCGTCAGCGAGTGGTCAGCA  GCAAGAGAGTAGTGTGTTATCTCAAAGTGATCAGGCCAGTACATTGTCTCAATTAGGAGCTGATAGCTCG  TCAGCGAGTGGTCAGCAGCAAGAGAGTAGTGTGTTATCTCAAAGTGGGGCCAGTACATCGTCTCAATTAG  GGACTAATTGGCGGCAAGAGATGCGCTCCAAGGTTGCGAGTGTTGAGTACATTTTGGCTGCTCGTGCCCT  TATCTCTGTAGGGGTCTATGCTGCTCAGGAAGAGATCGCGAGATCGCTAGGGTATGCTCCCCTGCGTGTT  GCAGAAGTCGAAGAAATCGTGAGGGATGGCCTTGTACGCAGCTACTTTCATGATAGTGGCCTTTCACTAG  GCTCCATACGACTCGTGCTTATGCAGGTTGGGGATAAGTTGGGGCTACAAGGTTT | 1.CTGATAGCTCGTCAGCGAGTGGTCAGCGGCAAGAGAGTAGTGTGTTATCTCAAAGTGATCAGGCCAGTACATTGTCTCAATTAGGAG  2.CTGATAGCTCGTCAGCGAGTGGTCAGCAGCAAGAGAGTAGTGTGTTATCTCAAAGTGATCAGGCCAGTACATTGTCTCAATTAGGAG  3.CTGATAGCTCGTCAGCGAGTGGTCAGCAGCAAGAGAGTAGTGTGTTATCTCAAAGTG---GGGCCAGTACATCGTCTCAATTAGG | 297.  DSSSASGQRQESSVLSQSDQASTLSQLG  298.  DSSSASGQQQESSVLSQSDQASTLSQLG  299.  DSSSASGQQQESSVLSQSGASTSSQL | 3 |
| 78 | KC181911.1 | Philippines | Negros Occidental | >KC181911.1 Anaplasma marginale isolate Negros_Occidental_49_18a Msp1a gene, partial cds  ATGTCAGAGTTGTCCCTTCAGCCAACTGATAGCTCGTCAGCGAGTGGTCAGCAGCAAGAGAGTAGTGTGT  TATCTCAAAGTGATCAGGCCAGTACATCGTCTCAATTAGGGACTGATAGCTCGTCAGCGAGTGGTCAGCA  GCAAGAGAGTAGTGTGTTATCTCAAAGTGGGGCCAGTACATCGTCTCAATTAGGGACTAATTGGCGGCAA  GAGATGCGCTCCAAGGTTGCGAGTGTTGAGTACATTTTGGCTGCTCGTGCCCTTATCTCTGTAGGGGTCT  ATGCTGCTCAGGAAGAGATCGCGAGATCGCTAGGGTATGCTCCCCTGCGTGTTGCAGAAGTCGAAGAAAT  CGTGAGGGATGGCCTTGTACGCAGCCACTTTCATGATAGTGGCCTTTCACTAGGCTCCATACGACTCGTG  CTTATGCAGGTTGGGGATAAGTTGGGGCTACAAGGTTT | 1.ACTGATAGCTCGTCAGCGAGTGGTCAGCAGCAAGAGAGTAGTGTGTTATCTCAAAGTGATCAGGCCAGTACATCGTCTCAATTAGGG  2.ACTGATAGCTCGTCAGCGAGTGGTCAGCAGCAAGAGAGTAGTGTGTTATCTCAAAGTG-GGGCCAGTACATCGTCTCAATTAGGGACT | 300.  TDSSSASGQQQESSVLSQSDQASTSSQLG  301.  TDSSSASGQQQESSVLSQSGASTSSQLGT | 2 |
| 79 | KC181910.1 | Philippines | Negros Occidental | >KC181910.1 Anaplasma marginale isolate Negros_Occidental_44_17a Msp1a gene, partial cds  ATGTCAGAGTTGTCCCTTCAGTCAACTGATAGCTCGTCAGCGAGTGGTCAGCGGCAAGAGAGTAGTGTGT  TATCTCAAAGTGATCAGGCCAGTACATTGTCTCAATTAGGAGCTGATAGCTCGTCAGCGAGTGGTCAGCA  GCAAGAGAGTAGTGTGTTATCTCAAAGTGATCAGGCCAGTACATTGTCTCAATTAGGAGCTGATAGCTCG  TCAGCGAGTGGTCAGCAGCAAGAGAGTAGTGTGTTATCTCAAAGTGGGGCCAGTACATCGTCTCAATTAG  GGACTAATTGGCGGCAAGAGATGCGCTCCAAGGTTGCGAGTGTTGAGTACATTTTGGCTGCTCGTGCCCT  TATCTCTGTAGGGGTCTATGCTGCTCAGGAAGAGATCGCGAGATCGCTAGGGTATGCTCCCCTGCGTGTT  GCAGAAGTCGAAGAAATCGTGAGGGATGGCCTTGTACGCAGCTACTTTCATGATAGTGGCCTTTCACTAG  GCTCCATACGACTCGTGCTTATGCAGGTTGGGGATAAGTTGGGGCTACAAGGTTT | 1.CTGATAGCTCGTCAGCGAGTGGTCAGCGGCAAGAGAGTAGTGTGTTATCTCAAAGTGATCAGGCCAGTACATTGTCTCAATTAGGAG  2.CTGATAGCTCGTCAGCGAGTGGTCAGCAGCAAGAGAGTAGTGTGTTATCTCAAAGTGATCAGGCCAGTACATTGTCTCAATTAGGAG  3.CTGATAGCTCGTCAGCGAGTGGTCAGCAGCAAGAGAGTAGTGTGTTATCTCAAAGTG---GGGCCAGTACATCGTCTCAATTAGG | 302.  DSSSASGQRQESSVLSQSDQASTLSQLG  303.  DSSSASGQQQESSVLSQSDQASTLSQLG  304.  DSSSASGQQQESSVLSQSGASTSSQL | 3 |
| 80 | KC181909.1 | Philippines | Negros Occidental | >KC181909.1 Anaplasma marginale isolate Negros_Occidental_37_16a Msp1a gene, partial cds  ATGTCAGAGTTGTCCTTTCAGTCAACTGATAGCTCGTCAGCGAGTGGTCAGCAGCAAGAGAGTAGTGTGT  TATCTCAAAGTGATCAGGCCAGTACATCGTCTCAATTAGGGACTAATTGGCGGCAAGAGATGCGCTCCAA  GGTTGCGAGTGTTGAGTACATTTTGGCTGCTCGTGCCCTTATCTCTGTAGGGGTCTATGCTGCTCAGGAA  GCGATCGCGAGATCGCTAGGGTATGCTCCCCTGCGTGTTGCAGAAGTCGAAGAAATCGTGAGGGATGGCC  TTGTACGCAGCCACTTTCATGATAGTGGCCTTTCACTAGGCTCCATACGACTCGTGCTTATGCAGGTTGG  GGATAAGTTGGGGCTACAAGGTTT | N/A | N/A | N/A |
| 81 | KC181908.1 | Philippines | Negros Occidental | >KC181908.1 Anaplasma marginale isolate Negros_Occidental_35_15a Msp1a gene, partial cds  ATGTCAGAGTATGTGTCCAGCCAGCCAGCTGATAGCTCGTCAGCGGGTGATCAGCAGCAAGAGAGTAGTG  TGTCATCTCAAAGTGATGCCAGTACATCGTCTCAATTAGGGACTGATAGCTCGTCAGCGGGTGATCAGCA  GCAAGAGAGTAGTGTGTCATCTCAAAGTGATGCCAGTACATCGTCTCAATTAGGGACTGATTGGCGGCAA  GAGGTGCACTCCAAGGTTGCGAGTGTTGAGTACATTTTGGCTGCTCGTGCCCTTATCTCTGTAGGGGTCT  ATGCTGCTCAGAGAGTGATCGCGCAATCGCAAGGGTGTGCTTCCCTGAGTGTTGCAGAAGTCGAAGAAAT  CGTGAGGGATAGCCTTGTACGCAGCCACTTTCATGATAGTGGCCTTTCACTAGGCCCCATACGACTCGTG  CTTATGCAGGTTGGGGATAAGTTGGGGCTACAAGGTTT | 1.CTGATAGCTCGTCAGCGGGTGATCAGCAGCAAGAGAGTAGTGTGTCATCTCAAAGTGATGCCAGTACATCGTCTCAATTAGGGA  2.CTGATAGCTCGTCAGCGGGTGATCAGCAGCAAGAGAGTAGTGTGTCATCTCAAAGTGATGCCAGTACATCGTCTCAATTAGGGA  CTGAT | 305.  DSSSAGDQQQESSVSSQSDASTSSQLG  306.  DSSSAGDQQQESSVSSQSDASTSSQLG  307.  TD | 2 |
| 82 | KC181907.1 | Philippines | Iloilo | >KC181907.1 Anaplasma marginale isolate Iloilo_147_13a Msp1a gene, partial cds  ATGTCAGATTATGTGTCCAGCCAGTCAGCTGATAGCTCGTCAGCGGGTGATCAGCAGCAAGAGAGTAGTG  TGTTATCTCAAAGTGGTCAGGCCAGTACATCGTCTCAATTAGGAGCTGATAGCTCGTCAGCGAGTGGTCA  GCAGCAAGAGAGTAGTGTGTCATCTCAAAGTGGTCAGGCCAGTACATCGTCTCAATTAGGAGCTGATAGC  TCGTCAGCGAGTGGTCAGCAGCAAGAGAGTAGTGTGTCATCTCAAAGTGGTCAGGCCAGTACATCGTCTC  AATTAGGAGCTGATTGGTGGCAAGAGATGCGCTCCAAGGTTGCGAGTGTTGAGTACATTCTGGCTGCTCG  TGCCCTTATTTCTGTAGGGGTCTATGCTGCTCAGGAAGAGATCGCGAGATCGCTAGGGTATGCTCCCCTG  TGTGTTGCAGAAGTCGAAGAAATCGTGAGGGATAGCCTTGTACGCAGCCACTTTCATGATAGTGGCCTTT  CACTAGGCTCCATACGACTCGTGCTTATGCGGGTTGGGGATAAGTTGGGGCTACAAGGTTT | 1.AGCTGATAGCTCGTCAGCGGGTGATCAGCAGCAAGAGAGTAGTGTGTTATCTCAAAGTGGTCAGGCCAGTACATCGTCTCAATTAGG  2.AGCTGATAGCTCGTCAGCGAGTGGTCAGCAGCAAGAGAGTAGTGTGTCATCTCAAAGTGGTCAGGCCAGTACATCGTCTCAATTAGG  3.AGCTGATAGCTCGTCAGCGAGTGGTCAGCAGCAAGAGAGTAGTGTGTCATCTCAAAGTGGTCAGGCCAGTACATCGTCTCAATTAGG  AGCTGAT | 308.  ADSSSAGDQQQESSVLSQSGQASTSSQL  309.  ADSSSASGQQQESSVSSQSGQASTSSQL  310.  ADSSSASGQQQESSVSSQSGQASTSSQL  311.  GAD | 3 |
| 83 | KC181906.1 | Philippines | Cavite | >KC181906.1 Anaplasma marginale isolate Cavite_18_9a Msp1a gene, partial cds  ATGTCAGAGTATGCGTCCAACCAGCCAGCTGATAGCTCGTCAGCGAGTGATCAGCAGCAAGAGAGTGGTG  TGCCATCTCAAAGTGAGGCCAGTACATCGTCTCAATTAGGAGCTGATAGCTCGTCAGCGAGTGGTCAGCA  GCAAGAGAGTGGTGTGCCATCTCAAAGTGAGGCCAGTACATCGTCTCAATTAGGAGCTGATAGCTCGTCA  GCGAGTGGTCAGCAGCAAGAGAGTAGTGTGTTATCTCAAAGTGGTCAGGCCAGTACATCGTCTCAATTAG  GGACTGATTGGCGGCAAGAGATGCGCTCCAAGGTTGCGAGTGTTGAGTACATTTTGGCTGCTCGTGCCCC  TATCTCTGTAGGGGTCTATGCTGCTCAGGAAGAGATCGCGAAATCGCTAGGGTATACTCCCCTGCTTGTT  GCAGAAGTCGAAGCAATCGTGAGGGATAGCCTTGTACGCAGCCACTTTTGTGATAGTGGCCTTTCACTAG  GCTCCATACGACTCGTGCTTATGCAGGTTGGGGATAAGTTGGGGCTACAAGGTTT | 1.AGCTGATAGCTCGTCAGCGAGTGATCAGCAGCAAGAGAGTGGTGTGCCATCTCAAAGTGAGGCCAGTACATCGTCTCAATTAGG  2.AGCTGATAGCTCGTCAGCGAGTGGTCAGCAGCAAGAGAGTGGTGTGCCATCTCAAAGTGAGGCCAGTACATCGTCTCAATTAGG  3.AGCTGATAGCTCGTCAGCGAGTGGTCAGCAGCAAGAGAGTAGTGTGTTATCTCAAAGTGGTCAGGCCAGTACATCGTCTCAATTAGG | 312.  ADSSSASDQQQESGVPSQSEASTSSQL  313.  ADSSSASGQQQESGVPSQSEASTSSQL  314.ADSSSASGQQQESSVLSQSGQASTSSQL | 3 |
| 84 | KC181905.1 | Philippines | Cavite | >KC181905.1 Anaplasma marginale isolate Cavite_13_8a Msp1a gene, partial cds  ATGTCAGAGTTGTCCCTTCAGTCAACTGATAGCTCGTCAGCGAGTGGTCAGCAGCAAGAGAGTAGTGTGT  TATCTCAAAGTGATCAGGCCAGTACATCGTCTCAATTAGGAGCTGATAGCTCGTCAGCGAGTGGTCAGCA  GCAAGAGAGTAGTGTGTTATCTCAAAGTGATCAGGCCAGTACATCGTCTCAATTAGGGACTGATTGGCGG  CAAGAGATGCGCTCCAAGGTTGCGAGTGTTGAGTACATTTTGGCTGCTCGTGCCCTTATCTCTGTAGGGG  TCTATGCTGCTCAGGAAGAGATCGCGAGATCGCTAGGGTATGCTCCCCTGCGTGTTGCAGAAGTCGAAGC  AATCGTGAGGGATAGCCTTGTACGCAGCCACTTTCATGATAGTGGCCTTTCACTAGGCTCCATACGACTC  GTGCTTATGCAGGTTGGGGATAAGTTGGGGCTACAAGGTTT | 1.CTGATAGCTCGTCAGCGAGTGGTCAGCAGCAAGAGAGTAGTGTGTTATCTCAAAGTGATCAGGCCAGTACATCGTCTCAATTAGGAG  2.CTGATAGCTCGTCAGCGAGTGGTCAGCAGCAAGAGAGTAGTGTGTTATCTCAAAGTGATCAGGCCAGTACATCGTCTCAATTAGG | 315.  DSSSASGQQQESSVLSQSDQASTSSQLG  316.  DSSSASGQQQESSVLSQSDQASTSSQL | 2 |
| 85 | KC181904.1 | Philippines | Cavite | >KC181904.1 Anaplasma marginale isolate Cavite_12_7a Msp1a gene, partial cds  ATGTCAGAGTATGCGTCCAACCAGCCAGCTGATAGCTCGTCAGCGAGTGATCAGCAGCAAGAGAGTAGTG  TGTTATCTCAAAGTGGTCAGGCCAGTACATCGTCTCAATTAGGGACTGATAGCTCGTCAGCGAGTGGTCA  GCAGCAAGAGAGTAGTGTGTTATCTCAAAGTGGTCAGGCCAGTACATCGTCTCAATTAGGGACTGATAGC  TCGTCAGCGAGTGGTCAGCAGCAAGAGAGTAGTGTGTTATCTCAAAGTGGTCAGGCCAGTACATCGTCTC  AATTAGGGACTGATTGGCGGCAAGAGATGCGCTCCAAGGTTGCGAGTGTTGAGTACATTTTGGCTGCTCG  TGCCCTTATCTCTGTAGGGGTCTATGCTGCTCAGGAAGAGATCGCGAAATCGCTAGGGTATACTCCCCTG  CGTGTTGCAGAAGTCGAAGTAATCGTGAGGGATAGCCTTGTACGCAGCCACTTTTGTGATAGTGGCCTTT  CACTAGGCTCCATACGACTCGTGCTTATGCAGGTTGGGGATAAGTTGGGGCTACAAGGTTT | 1.CTGATAGCTCGTCAGCGAGTGATCAGCAGCAAGAGAGTAGTGTGTTATCTCAAAGTGGTCAGGCCAGTACATCGTCTCAATTAGGGA  2.CTGATAGCTCGTCAGCGAGTGGTCAGCAGCAAGAGAGTAGTGTGTTATCTCAAAGTGGTCAGGCCAGTACATCGTCTCAATTAGGGA  3.CTGATAGCTCGTCAGCGAGTGGTCAGCAGCAAGAGAGTAGTGTGTTATCTCAAAGTGGTCAGGCCAGTACATCGTCTCAATTAGGGA  CTGAT | 317.  DSSSASDQQQESSVLSQSGQASTSSQLG  318.  DSSSASGQQQESSVLSQSGQASTSSQLG  319.  DSSSASGQQQESSVLSQSGQASTSSQLG  320.  TD | 3 |
| 86 | KC181903.1 | Philippines | Batangas | >KC181903.1 Anaplasma marginale isolate Batangas_293_5a Msp1a gene, partial cds  ATGTCAGAGTATGTGTCCAACCAGCCAGCTGATAGCTCGTCAGCGAGTGATCAGCAGCAAGAGAGTGGTG  TGCCATCTCAAAGTGAGGCCAGTACATCGTCTCAATTAGGAGCTGATAGCTCGTCAGCGAGTGGTCAGCA  GCAAGAGAGTAGTGTGTCATCTCAAAGTGGTCAGGCCAGTACATCGTCTCAATTAGGAGCTGATAGCTCG  TCAGCGAGTGGTCAGCAGCAAGAGAGTAGTGTGTTATCTCAAAGTGGTCAGGCCAGTACATCGTCTCAAT  TAGGAGCTGATAGCTCGTCAGCGAGTGGTCAGCAGCAAGAGAGTAGTGTGTTATCTCAAAGTGGTCAGGC  CAGTACATCGTCTCAATTAGGAGCTGATAGCTCGTCAGCGAGTGGTCAGCAGCAAGAGAGTAGTGTGTCA  TCTCAAAGTGGTCAGGCCAGTACATCGTCTCAATTAGGAGCTGATAGCTCGTCAGCGAGTGGTCAGCAGC  AAGAGAGTAGTGTGTTATCTCAAAGTGGTCAGGCCAGTACATCGTCTCAATTAGGGACTGATAGCTCGTC  AGCGAGTGGTCAGCAGCAAGAGAGTAGTGTGTTATCTCAAAGTGGTCAGGCCAGTACATCGTCTCAATTA  GGAGCTGATAGCTCGTCAGCGAGTGGTCAGCAGCAAGAGAGTAGTGTGTCATCTCAAAGTGGTCAGGCCA  GTACATCGTCTCAATTAGGGACTGATTGGCGGCAAGAGATGCGCTCCAAGGTTGCGAGTGTTGAGTACAT  TTTGGCTGCTCGTGCCCTTATCTCTGTAGGGGTCTATGCTGCTCAGGAAGAGATCGCGAAATCGCTAGGG  TATACTCCCCTGCGTGTTGCAGAAGTCGAAGCAATCGTGAGGGATAGCCTTGTACGCAGCCGCTTTTGTG  ATAGTGGCCTTTCACTAGGCTCCATACGACTCGTGCTTATGCAGGTTGGGGATAAGTTGGGGCTACAAGG  TTT | 1.AGCTGATAGCTCGTCAGCGAGTGATCAGCAGCAAGAGAGTGGTGTGCCATCTCAAAGT-G--AGGCCAGTACATCGTCTCAATTAGG  2.AGCTGATAGCTCGTCAGCGAGTGGTCAGCAGCAAGAGAGTAGTGTGTCATCTCAAAGTGGTCAGGCCAGTACATCGTCTCAATTAGG  3.AGCTGATAGCTCGTCAGCGAGTGGTCAGCAGCAAGAGAGTAGTGTGTTATCTCAAAGTGGTCAGGCCAGTACATCGTCTCAATTAGG  4.AGCTGATAGCTCGTCAGCGAGTGGTCAGCAGCAAGAGAGTAGTGTGTTATCTCAAAGTGGTCAGGCCAGTACATCGTCTCAATTAGG  5.AGCTGATAGCTCGTCAGCGAGTGGTCAGCAGCAAGAGAGTAGTGTGTCATCTCAAAGTGGTCAGGCCAGTACATCGTCTCAATTAGG  6.AGCTGATAGCTCGTCAGCGAGTGGTCAGCAGCAAGAGAGTAGTGTGTTATCTCAAAGTGGTCAGGCCAGTACATCGTCTCAATTAGG  7.-GACTGATAGCTCGTCAGCGAGTGGTCAGCAGCAAGAGAGTAGTGTGTTATCTCAAAGTGGTCAGGCCAGTACATCGTCTCAATTAGG  8.AGCTGATAGCTCGTCAGCGAGTGGTCAGCAGCAAGAGAGTAGTGTGTCATCTCAAAGTGGTCAGGCCAGTACATCGTCTCAATTAGG | 321.  ADSSSASDQQQESGVPSQSEASTSSQL  322.  ADSSSASGQQQESSVSSQSGQASTSSQL  323.  ADSSSASGQQQESSVLSQSGQASTSSQL  324.  ADSSSASGQQQESSVLSQSGQASTSSQL  325.  ADSSSASGQQQESSVSSQSGQASTSSQL  326.  ADSSSASGQQQESSVLSQSGQASTSSQL  327.  TDSSSASGQQQESSVLSQSGQASTSSQL  328.  ADSSSASGQQQESSVSSQSGQASTSSQL | 8 |
| 87 | KC181902.1 | Philippines | Batangas | >KC181902.1 Anaplasma marginale isolate Batangas_235_4a Msp1a gene, partial cds  ATGTCAGATTATGTGTCCAGCCAGTCAGCTGATAGCTCGTCAGCGAGTGGTCAGCAGCAAGAGAGTAGTG  TGTCATCTCAATTAGGAGCTGATAGCTCGTCAGCGAGTGGTCAGCAGCAAGAGAGTAGTGTGTCATCTCA  ATTAGGAGCTGATAGCTCGTCAGCGAGTGGTCAGCAGCAAGAGAGTAGTGTGTCATCTCAATTAGGAGCT  GATAGCTCGTCAGCGAGTGGTCAGCAGCAAGAGAGTAGTGTGTCATCTCAATTAGGAGCTGATAGCTCGT  CAGCGAGTGGTCAGCAGCAAGAGAGTAGTGTGTCATCTCAAAGTGGTCAGGCCAGTACATCGTCTCAATT  AGGGACTGATTGGCGGCAAGAGATGCGCTCCAAGGTTGCGAGTGTTGAGTACATTTTGGCTGCTCGTGCC  CTTATCTCTGTAGGGGTCTATGCTGCTCAGGAAGAGATCGCGAAATCGCTAGGGTATACTCCCCTGCGTG  TTGCAGAAGTCGAAGCAATCGTGAGGGATAGCCTTGTACGCAGCCACTTTCGTGATAGTGGCCTTTCACT  AGGCTCCATACGACTCGTGCTTATGCAGGTTGGGGATAAGTTGGGGCTACAAGGTTT | 1.AGCTGATAGCTCGTCAGCGAGTGGTCAGCAGCAAGAGAGTAGTGTGTCATCTCAATTAGG  2. AGCTGATAGCTCGTCAGCGAGTGGTCAGCAGCAAGAGAGTAGTGTGTCATCTCAATTAGG  3.AGCTGATAGCTCGTCAGCGAGTGGTCAGCAGCAAGAGAGTAGTGTGTCATCTCAATTAGG  4.AGCTGATAGCTCGTCAGCGAGTGGTCAGCAGCAAGAGAGTAGTGTGTCATCTCAATTAGG  5.AGCTGATAGCTCGTCAGCGAGTGGTCAGCAGCAAGAGAGTAGTGTGTCATCTCAA | 329.  ADSSSASGQQQESSVSSQL  330.  ADSSSASGQQQESSVSSQL  331.  ADSSSASGQQQESSVSSQL  332.  ADSSSASGQQQESSVSSQL  333.  ADSSSASGQQQESSVSSQ | 5 |
| 88 | KC181901.1 | Philippines | Batangas | >KC181901.1 Anaplasma marginale isolate Batangas_46_2a Msp1a gene, partial cds  ATGTCAGAGTTGTTCCTTCAGTCAACTGATAGCTCGTCAGCGAGTGGTCAGCAGCAAGAGAGTAGTGTGT  TATCTCAAAGTGATCAGGCCAGTACATCGTCTCAATTAGGGACTAATTGGCGGCAAGAGATGCGCTCCAA  GGTTGCGAGTGTTGAGTACATTTTGGCTGCTCGTGCCCTTATCTCTGTAGGGGTCTATGCTGCTCAGGAA  GCGATCGCGAGATCGCTAGGGTATGCTCCCCTGCGTGTTGCAGAAGTCGAAGAAATCGTGAGGGATGGCC  TTGTACGCAGCCACTTTCATGATAGTGGCCTTTCACTAGGCTCCATACGACTCGTGCTTATGCAGGTTGG  GGATAAGTTGGGGCTACAAGGTTT | N/A | N/A | N/A |
| 89 | KC181900.1 | Philippines | Negros Occidental | >KC181900.1 Anaplasma marginale isolate Negros_Oriental_269 Msp1a gene, partial cds  ATGTCAGAGTGTGTGTCCCTTCAGCCAACTGATAGCTCGTCAGCGAGTGGTCAGAAGCAAGAGAGTAGTG  TGTTATCTCAAAGTGATCAGGCCAGTACATCGTCTCAATTAGGGACTGATTGGCGGCAAGAGATGCGCTC  CAAGGTTGCGAGTGTTGAGTACATTTTGGCTGCTCGTGCCCTTATCTCTGTAGGGGTCTATGCTGCTCAG  GAAGAGATCGCGAGATCGCTAGGGTATGCTCCCCTGCGTGCTGCAGAAGTCGAAGAAATCGTGAGGGATG  GCCTTGTACGCAGCCACTTTCATGATAGTGGCCTTTCACTAGGCTCCATACGACTCGTGCTTATGCAGGT  TGGGGATAAGTTGGGGCTACAAGGTTT | N/A | N/A | N/A |
| 90 | KC181899.1 | Philippines | Negros Occidental | >KC181899.1 Anaplasma marginale isolate Negros_Occidental_30 Msp1a gene, partial cds  ATGTCAGAGTTGTCCCTTCAGTCAACTGATAGCTCGTCAGCGAGTGGTCAGCAGCAAGAGAGTAGTGTGT  TATCTCAAAGTGATCAGGCCAGTACATCGTCTCAATTAGGAGCTGATAGCTCGTCAGCGAGTGGTCAGCA  GCAAGAGAGTAGTGTGTTATCTCAAAGTGATCAGGCCAGTACATCGTCTCAATTAGGGACTAATTGGCGG  CAAGAGATGCGCTCCAAGGTTGCGAGTGTTGAGTACATTTTGGCTGCTCGTGCCCTTATCTCTGTAGGGG  TCTATGCTGCTCAGGAAGCGATCGCGAGATCGCTAGGGTATGCTCCCCTGCGTGTTGCAGAAGTCGAAGA  AATCGTGAGGGATGGCCTTGTACGCAGCCACTTTCATGATAGTGGCCTTTCACTAGGCTCCATACGACTC  GTGCTTATGCAGGTTGGGGATAAGTTGGGGCTACAAGGTTT | 1.CTGATAGCTCGTCAGCGAGTGGTCAGCAGCAAGAGAGTAGTGTGTTATCTCAAAGTGATCAGGCCAGTACATCGTCTCAATTAGGAG  2.CTGATAGCTCGTCAGCGAGTGGTCAGCAGCAAGAGAGTAGTGTGTTATCTCAAAGTGATCAGGCCAGTACATCGTCTCAATTAGG | 334.  DSSSASGQQQESSVLSQSDQASTSSQLG  335.  DSSSASGQQQESSVLSQSDQASTSSQL | 2 |
| 91 | KC181898.1 | Philippines | Iloilo | >KC181898.1 Anaplasma marginale isolate Iloilo_122 Msp1a gene, partial cds  ATGTCAGAGTATGTGTCCAGCCAGCCAGCTGATAGCTCGTCAGCGGGTGATCAGCAGCAAGAGAGTAGTG  TGTCATCTCAAAGTGGTGCCAGTACATCGTCTCAATTAGGGACTGATAGCTCGTCAGCGGGTGATCAGCA  GCAAGAGAGTAGTGTGTCATCTCAAAGTGATGCCAGTACATCGTCTCAATTAGGGACTGATAGCTCGTCA  GCGGGTGATCAGCAGCAAGAGAGTAGTGTGTCATCTCAAAGTGATGCCAGTACATCGTCTCAATTAGGGA  CTGATAGCTCGTCAGCGGGTGATCAGCAGCAAGAGAGTAGTGTGTCATCTCAAAGTGGTGCCAGTACATC  GTCTCAATTAGGGACTGATAGCTCGTCAGCGGGTGATCAGCAGCAAGAGAGTAGTGTGTCATCTCAAAGT  GGTGCCAGTACATCGTCTCAATTAGGGACTGATTGGCGGCAAGAGGTGCACTCCAAGGTTGCGAGTGTTG  AGTACATTTTGGCTGCTCGTGCCCTTATCTCTGTAGGGGTCTATGCTGCTCAGAGAGTGATCGCGCAATC  GCAAGGGTGTGCTTCCCTGAGTGTTGCAGAAGTCGAAGAAATCGTGAGGGATAGCCTTGTACGCAGCCAC  TTTCATGATAGTGGCCTTTCACTAGGCTCCATACGACTCGTGCTTATGCAGGTTGGGGATAAGTTGGGGC  TACAAGGTTT | 1.CTGATAGCTCGTCAGCGGGTGATCAGCAGCAAGAGAGTAGTGTGTCATCTCAAAGTGGTGCCAGTACATCGTCTCAATTAGGGA  2.CTGATAGCTCGTCAGCGGGTGATCAGCAGCAAGAGAGTAGTGTGTCATCTCAAAGTGATGCCAGTACATCGTCTCAATTAGGGA  3.CTGATAGCTCGTCAGCGGGTGATCAGCAGCAAGAGAGTAGTGTGTCATCTCAAAGTGATGCCAGTACATCGTCTCAATTAGGGA  4.CTGATAGCTCGTCAGCGGGTGATCAGCAGCAAGAGAGTAGTGTGTCATCTCAAAGTGGTGCCAGTACATCGTCTCAATTAGGGA  5.CTGATAGCTCGTCAGCGGGTGATCAGCAGCAAGAGAGTAGTGTGTCATCTCAAAGTGGTGCCAGTACATCGTCTCAATTAGGGA  CTGAT | 336.  DSSSAGDQQQESSVSSQSGASTSSQLG  337.  DSSSAGDQQQESSVSSQSDASTSSQLG  338. DSSSAGDQQQESSVSSQSDASTSSQLG  339.  DSSSAGDQQQESSVSSQSGASTSSQLG  340.  DSSSAGDQQQESSVSSQSGASTSSQLG  342.  TD | 5 |
| 92 | KC181897.1 | Philippines | Cavite | >KC181897.1 Anaplasma marginale isolate Cavite_7 Msp1a gene, partial cds  ATGTCAGAGTTGTCCCTTCAGTCAACTGATAGCTCGTCAGCGAGTGGTCAGCAGCAAGAGAGTAGTGTGT  TATCTCAAAGTGATCAGGCCAGTACATCGTCTCAATTAGGAGCTGATAGCTCGTCAGCGAGTGGTCAGCA  GCAAGAGAGTAGTGTGTTATCTCAAAGTGATCAGGCCAGTACATCGTCTCAATTAGGGACTAATTGGCGG  CAAGAGATGCGCTCCAAGGTTGCGAGTGTTGAGTACATTTTGGCTGCTCGTGCCCTTATCTCTGTAGGGG  TCTATGCTGCTCAGGAAGCGATCGCGAGATCGCTAGGGTATGCTCCCCTGCGTGTTGCAGAAGTCGAAGA  AATCGTGAGGGATGGCCTTGTACGCAGCCACTTTCATGATAGTGGCCTTTCACTAGGCTCCATACGACTC  GTGCTTATGCAGGTTGGGGATAAGTTGGGGCTACAAGGTTT | 1.CTGATAGCTCGTCAGCGAGTGGTCAGCAGCAAGAGAGTAGTGTGTTATCTCAAAGTGATCAGGCCAGTACATCGTCTCAATTAGGAG  2.CTGATAGCTCGTCAGCGAGTGGTCAGCAGCAAGAGAGTAGTGTGTTATCTCAAAGTGATCAGGCCAGTACATCGTCTCAATTAGG | 343.  DSSSASGQQQESSVLSQSDQASTSSQLG  344.  DSSSASGQQQESSVLSQSDQASTSSQL | 2 |
| 93 | KC181868.1 | Philippines | Batangas | >KC181868.1 Anaplasma marginale isolate Batangas_1c Msp1a gene, partial cds  ATGTCAGAGTTGTCCCTTCGGTCAACTGATAGCTCGTCAGCGAGTGGTCAGCAGCAAGAGAGTAGTGTGT  TATCTCAAAGTGATCAGGCCAGTACATCGTCTCAATTAGGAGCTGATAGCTCGTCAGCGAGTGGTCAGCA  GCAAGAGAGTAGTGTGTTATCTCAAAGTGATCAGGCCAGTACATCGTCTCAATTAGGAGCTGATAGCTCG  TCAGCGAGTGGTCAGCAGCAAGAGAGTAGTGTGTTATCTCAAAGTGATCAGGCCAGTACATCGTCTCAAT  TAGGGACTGATTGGCGGCAAGAGATGCGCTCCAAGGTTGCGAGTGTTGAGTACATTTTGGCTGCTCGTGC  CCTTATCTCTGTAGGGGTCTATGCTGCTCAGGAAGAGATCGCGAGATCGCTAGGGTATGCTCCCCTGCGT  GTTGCAGAAGTCGAAGCAATCGTGAGGGATAGCCTTGTACGCAGCCACTTTCATGATAGTGGCCTTTCAC  TAGGCTCCATACGACTCGTGCTTATGCAGGTTGGGGATAAGTTGGGGCTACAAGGTTT | 1.CTGATAGCTCGTCAGCGAGTGGTCAGCAGCAAGAGAGTAGTGTGTTATCTCAAAGTGATCAGGCCAGTACATCGTCTCAATTAGGAG  2.CTGATAGCTCGTCAGCGAGTGGTCAGCAGCAAGAGAGTAGTGTGTTATCTCAAAGTGATCAGGCCAGTACATCGTCTCAATTAGGAG  3.CTGATAGCTCGTCAGCGAGTGGTCAGCAGCAAGAGAGTAGTGTGTTATCTCAAAGTGATCAGGCCAGTACATCGTCTCAATTAGG | 345.  DSSSASGQQQESSVLSQSDQASTSSQLG  346.  DSSSASGQQQESSVLSQSDQASTSSQLG  347.  DSSSASGQQQESSVLSQSDQASTSSQL | 3 |
| 94 | KC181867.1 | Philippines | Batangas | >KC181867.1 Anaplasma marginale isolate Batangas_1b Msp1a gene, partial cds  ATGTCAGAGTATGTGTCCAGCCAGCCAGCTGATAGCTCGTCAGCGAGTGGTGTGTTATCTAAAAGTGATC  AGGCCAGTACATCGTCTCAATTAGGAGCTGATAGCTCGTCAGCGAGTGGTCAGCAGCAAGAGAGTAGTGT  GTTATCTCAAAGTGATCAGGCCAGTACATCGTCTCAATTAGGAGCTGATAGCTCGTCAGCGAGTGGTCAG  CAGCAAGAGAGTAGTGTGTTATCTCAAAGTGATCAGGCCAGTACATCGTCTCAATTAGGGACTGATTGGC  GGCAAGAGATGCGCTCCAAGGTTGCGAGTGTTGAGTACATTTTGGCTGCTCGTGCCCTTATCTCTGTAGG  GGTCTATGCTGCTCAGGAAGAGATCGCGAGATCGCTAGGGTATGCTCCCCTGCGTGTTGCAGAAGTCGAA  GCAATCGTGAGGGATAGCCTTGTACGCAGCCACTTTCATGATAGTGGCCTTTCACTAGGCTCCATACGAC  TCGTGCTTATGCAGGTTGGGGATAAGTTGGGGCTACAAGGTTT | 1.GAGTGGTGTGTTATCTAAAAGTGATCAGGCCAGTACATCGTCTCAATTAGGAGCTGATAGCTCGTCAGCGAGTGGTCAGCAGCAAGA  2.GAGTAGTGTGTTATCTCAAAGTGATCAGGCCAGTACATCGTCTCAATTAGGAGCTGATAGCTCGTCAGCGAGTGGTCAGCAGCAAGA  3.GAGTAGTGTGTTATCTCAAAGTGATCAGGCCAGTACATCGTCTCAATTAGG | 348.  SGVLSKSDQASTSSQLGADSSSASGQQQ  349.  SSVLSQSDQASTSSQLGADSSSASGQQQ  350.  SSVLSQSDQASTSSQL | 3 |
| 95 | KC181866.1 | Philippines | Batangas | >KC181866.1 Anaplasma marginale isolate Batangas_1a Msp1a gene, partial cds  ATGTCAGAGTATGTGTCCAGCCAGCCAGCTGATAGCTCGTCAGCGAGTGGTGTGTTATCTAAAAGTGATC  AGGCCAGTACATCGTCTCAATTAGGAACTGATAGCTCGTCAGCGGGTGATCAGCAGCAAGGGAGTGGTGT  GTCATCTCAAAGTGGTCAGGCCAGTACATCGTCTCAATTAGGAACTGATAGCTCGTCAGCGGGTGATCAG  CAGCAAGGGAGTGGTGTGTCATCTCAAAGTGGTCAGGCCAGTACATCGTCTCAATTAGGGACTGATAGCT  CGTCAGCGAGTGGTCAGCAGCAAGAGAGTAGTGTGTCATCTCAAAGTGATGCCAGTACATCGTCTCAATT  AGGAACTGATAGCTCGTCAGCGGGTGATCAGCAGCAAGGGAGTGGTGTGTCATCTCAAAGTGGTCAGGCC  AGTACATCGTCTCAATTAGGAACTGATAGCTCGTCAGCGGGTGATCAGCAGCAAGGGAGTGGTGTGTCAT  CTCAAAGTGGTCAGGCCAGTACATCGTCTCAATTAGGGACTGATAGCTCGTCAGCGAGTGGTCAGCAGCA  AGAGAGTAGTGTGTCATCTCAAAGTGATGCCAGTACATCGTCTCAATTAGGAGCTGATTGGCGGCAAGAG  GTGCACTCCAAGGTTGCGAGTGTTGAGTACATTTTGGCTGCTCGTGCCCTTATCTCTGTAGGGGTCTATG  CTGCTCAGAGAATGATCGCGCAATCGCGAGGGTGTGCTTCCCTGAGTGTTGCAGAAGTCGAAGAAATCGT  GAAGGATAGCCTTGTACGCAGCCACTTTCATGATAGTGGCCTTTCACTAGGCTCCATACGACTCGTGCTT  ATGCAGGTTGGGGATAAGTTGGGGCTACAAGGTTT | *INDICE 1:*- 1:GAGTGGTGTGTTATCTAAAAGTGATCAGGCCAGTACATCGTCTCAATTAGGAACTGATAGCTCGTCAGCGGGTGATCAGCAGCAAGG  2.GAGTGGTGTGTCATCTCAAAGTGGTCAGGCCAGTACATCGTCTCAATTAGGAACTGATAGCTCGTCAGCGGGTGATCAGCAGCAAGG  3.GAGTGGTGTGTCATCTCAAAGTGGTCAGGCCAGTACATCGTCTCAATTAGGGACTGATAGCTCGTCAGCGAGTGGTCAGCAGCAAGA  4.GAGTAGTGTGTCATCTCAAAGT-G--ATGCCAGTACATCGTCTCAATTAGGAACTGATAGCTCGTCAGCGGGTGATCAGCAGCAAGG  5.GAGTGGTGTGTCATCTCAAAGTGGTCAGGCCAGTACATCGTCTCAATTAGGAACTGATAGCTCGTCAGCGGGTGATCAGCAGCAAGG  6.GAGTGGTGTGTCATCTCAAAGTGGTCAGGCCAGTACATCGTCTCAATTAGGGACTGATAGCTCGTCAGCGAGTGGTCAGCAGCAAGA  7.GAGTAGTGTGTCATCTCAAAGT-G--ATGCCAGTACATCGTCTCAATTAGGAGCTGAT  *INDICE 2*:- 1.GAGTGGTGTGTTATCTAAAAGTGATCAGGCCAGTACATCGTCTCAATTAGGAACTGATAGCTCGTCAGCGGGTGATCAGCAGCAAGGGAGTGGTGTGTCATCTCAAAGTGGTCAGGCCAGTACATCGTCTCAATTAGGAACTGATAGCTCGTCAGCGGGTGATCAGCAGCAAGG  2.GAGTGGTGTGTCATCTCAAAGTGGTCAGGCCAGTACATCGTCTCAATTAGGGACTGATAGCTCGTCAGCGAGTGGTCAGCAGCAAGAGAGTAGTGTGTCATCTCAAAGT-G-ATGCCAGTACATCGTCTCAATTAGGAACTGATAGCTCGTCAGCGGGTGATCAGCAGCAAGG  3.GAGTGGTGTGTCATCTCAAAGTGGTCAGGCCAGTACATCGTCTCAATTAGGAACTGATAGCTCGTCAGCGGGTGATCAGCAGCAAGGGAGTGGTGTGTCATCTCAAAGTGGTCAGGCCAGTACATCGTCTCAATTAGGGACTGATAGCTCGTCAGCGAGTGGTCAGCAGCAAGA  GAGTAGTGTGTCATCTCAAAGT-G--ATGCCAGTACATCGTCTCAATTAGGAGCTGAT  *INDICE 3*:- 1.GAGTGGTGTGTTATCTAAAAGTGATCAGGCCAGTACATCGTCTCAATTAGGAACTGATAGCTCGTCAGCGGGTGATCAGCAGCAAGGGAGTGGTGTGTCATCTCAAAGTGGTCAGGCCAGTACATCGTCTCAATTAGGAACTGATAGCTCGTCAGCGGGTGATCAGCAGCAAGGGAGTGGTGTGTCATCTCAAAGTGGTCAGGCCAGTACATCGTCTCAATTAGGGACTGATAGCTCGTCAGCGAGTGGTCAGCAGCAAG  2.GAGTAGTGTGTCATCTCAAAGTGATGCCAGTACATCGTCTCAATTAGGAACTGATAGCTCGTCAGCGGGTGATCAGCAGCAAGGGAGTGGTGTGTCATCTCAAAGTGGTCAGGCCAGTACATCGTCTCAATTAGGAACTGATAGCTCGTCAGCGGGTGATCAGCAGCAAGGGAGTGGTGTGTCATCTCAAAGTGGTCAGGCCAGTACATCGTCTCAATTAGGGACTGATAGCTCGTCAGCGAGTGGTCAGCAGCAAGA  GAGTAGTGTGTCATCTCAAAGTGATGCCAGTACATCGTCTCAATTAGGAGCTGAT | *indice 1*:-  351.  SGVLSKSDQASTSSQLGTDSSSAGDQQQ  352.  SGVSSQSGQASTSSQLGTDSSSAGDQQQ  353.  SGVSSQSGQASTSSQLGTDSSSASGQQQ  354.  SSVSSQSDASTSSQLGTDSSSAGDQQQ  355.  SGVSSQSGQASTSSQLGTDSSSAGDQQQ  356.SGVSSQSGQASTSSQLGTDSSSASGQQQ  357.  SSVSSQSDASTSSQLGAD | first indice: 7, |
| 96 | MT664982.1 | Spain | Southern region | >MT664982.1 Anaplasma marginale isolate LO13 major surface protein (msp1a) gene, partial cds  GCGATTACAAGTTCCCTATGTTACAATCCGGCCGTCGGTGTGGTAGCGTGCTGGTTGTGTGGTTGTCCTC  TTTCCCGATGTTGGGTCGTTCGTTTTACGTCTCACAAGTTTGTACGCTGTGCCCCTGGCAGTGTAGGGTT  TGTTTGTGTGTGTGTTATGTCAGAGTATGTGTCCAGCCAGTCAGCTGATAGCTCGTCAGCGAGTGGTCAG  CAGCAAGAGAGTAGTGTGTCATCTCAAAGTGAGGCCAGTACATCGTCTCAATTAGGGACTGATAGCTCGT  CAGCGAGTGGTCAGCAGCAAGAGAGTAGTGTGTCATCTCAAAGTGAGGCCAGTACATCGTCTCAATTAGG  GACTGATAGCTCGTCAGCGAGTGGTCAGCAGCAAGAGAGTAGTGTGTCATCTCAAAGTGAGGCCAGTACA  TCGTCTCAATTAGGGACTGATAGCTCGTCAGCGAGTGGTCAGCAGCAAGAGAGTAGTGTGTCATCTCAAA  GTGAGGCCAGTACATCGTCTCACTTAGGGACTGATAGCTCGTCAGCGAGTGGTCAGCATCAAGAGAGTAC  TGTGTCATCTCAAAGTGATGCCAGTACATCGGCTCAATTAGACACTGATAG | CTGATAGCTCGTCAGCGAGTGGTCAGCAGCAAGAGAGTAGTGTGTCATCTCAAAGTGAGGCCAGTACATCGTCTCAATTAGGGA  CTGATAGCTCGTCAGCGAGTGGTCAGCAGCAAGAGAGTAGTGTGTCATCTCAAAGTGAGGCCAGTACATCGTCTCAATTAGGGA  CTGATAGCTCGTCAGCGAGTGGTCAGCAGCAAGAGAGTAGTGTGTCATCTCAAAGTGAGGCCAGTACATCGTCTCAATTAGGGA  CTGATAGCTCGTCAGCGAGTGGTCAGCAGCAAGAGAGTAGTGTGTCATCTCAAAGTGAGGCCAGTACATCGTCTCACTTAGGGA  CTGATAGCTCGTCAGCGAGTGGTCAGCATCAAGAGAGTACTGTGTCATCTCAAAGTGATGCCAGTACATCGGCTCAATTAGACA  CTGATAG | 365.  DSSSASGQQQESSVSSQSEASTSSQLG  366.  DSSSASGQQQESSVSSQSEASTSSQLG  367.  DSSSASGQQQESSVSSQSEASTSSQLG  368.  DSSSASGQQQESSVSSQSEASTSSHLG  369.  DSSSASGQHQESTVSSQSDASTSAQLD  370.  D | 5 |
| 97 | MF787747.1 | Turkey | Elazig | >MF787747.1 Anaplasma marginale isolate Cs72 major surface protein 1 alpha (msp1a) gene, partial cds  ATTGTTAAATTTAGTATATTAATCTTGCGATTACACGTTTCCGTATGTTACAATCAGGCCGCCGGTGTGG  TAGCGTGCTGGTTGTGTGGTTGTCCTCTTTCCCGATGTTGGGTCGTTCGTTTTACGCCTCACAAGTTTGT  ACGCTGTGCCCCTGGCAGTGTAGGGTTTGTTTGTTTGTGTGTGTGTTATGTCAAAGTATGGGTCCTCTCA  GCTATCTGGTAGCTCGTCAGCGAGTGGTCAGCAGCAAGAGAGTAGTGTGTTATCTCAAGGTGGTCAGGCC  AGTACATCGTCTCAATTAGGAACTGATAGCTCGTCAGCGAGTGGTCAGCAGCAAGGGGGTGGTGTGTCAC  CTCAAAGTGAGGCCAGTACATCGTCTCAATTAGGGACTGATAGCTCGTCAGCGAGTGGTCAGCAGCAAGA  GAGTGGTGTGTCACCTCAAAGTGAGGCCAGTACATCGTCTCAATTAGGGACTGATAGCTCGTCAGCGAGT  GGTCAGCAGCAAGAAAGTGGTGTGTCACCTCAAAGTGAGGCCAGTACATCGTCTCAATTAGGAGCTGATT  GGCGGCAAGAGATGC | 1.TAGCTCGTCAGCGAGTGGTCAGCAGCAAGAGAGTAGTGTGTTATCTCAAGGTGGTCAGGCCAGTACATCGTCTCAATTAGGAACTGA  2.TAGCTCGTCAGCGAGTGGTCAGCAGCAAGGGGGTGGTGTGTCACCTCAAAGTGAGGCCAGTACATCGTCTCAATTAGGGACTGA  3.TAGCTCGTCAGCGAGTGGTCAGCAGCAAGAGAGTGGTGTGTCACCTCAAAGTGAGGCCAGTACATCGTCTCAATTAGGGACTGA  4.TAGCTCGTCAGCGAGTGGTCAGCAGCAAGAAAGTGGTGTGTCACCTCAAAGTGAGGCCAGTACATCGTCTCAATTAGG | 371.  **S**SSASGQQQESSVLSQGGQASTSSQLGT  372.  **S**SSASGQQQGGGVSPQSEASTSSQLGT  373.  **S**SSASGQQQESGVSPQSEASTSSQLGT  374.**S**SSASGQQQESGVSPQSEASTSSQL | 4 |
| 98 | MF787746.1 | Turkey | Elazig | >MF787746.1 Anaplasma marginale isolate O24 major surface protein 1 alpha (msp1a) gene, partial cds  GGTTGTGCTCCCCAATTGTTAAAATTTAGTATATTAATCTTGCGATTACACGTTCCGTATGTTACAATCA  GGCCGTCGGTGTGGTAGCGTGCTGGTTGTGTGGTTGTCCTCTTTCCCGATGTTGGGTCGTTCGTTTTACG  TCTCACAAGTTTGTACGCTGTGCCCCTGGCAGTGTAGGGTTTGTTTGTGTGTGTGTTATGTCAGAGTATG  TGTCCAGACAGCCAGCTGATAGCTCGTCAGCGGGTGGTCAGCAGCAAGAGAGTAGTGTGTCATCTCAAAG  TGATCAGGCCAGTACATCGTCTCAATTAGGAACTGATAGCTCGTCAGCGAGTGGTCAGCAGCAAGAGAGT  AGTGTGTCATCTCAAAGTGATCAGGCCAGTACATCGTCTCAATTAGGAACTGATAGCTCGTCAGCGAGTG  GTCAGCAGCAAGAGAGTAGTGTGTCATCTCAAAGTGATCAGGCCAGTACATCGTCTCAATTAGGAACTGA  TTGGCGGCAAGAGATGCGCTCCAAGGTTGCGAGTGTTGAGTACATTTTGGCTGCTCGTGCCCTTATTTCT  GTAGGGGTCTATGCTGCTCAGGGAGAGATCGCGAGATCGCAAGGGTGTGCTCCCCTGCGTGTTGCAGAAG  TCGAAGAAATCGTGAGGGATAGCCTTGTACGCAGCCACTTTCATGATAGTGGCCTTTCACTAGGCTCCAT  ACGACTCGTGCTTATGCAGGTTGGGGATAAGTTGGGGCTACAAGGTTT | 1.CTGATAGCTCGTCAGCGGGTGGTCAGCAGCAAGAGAGTAGTGTGTCATCTCAAAGTGATCAGGCCAGTACATCGTCTCAATTAGGAA  2.CTGATAGCTCGTCAGCGAGTGGTCAGCAGCAAGAGAGTAGTGTGTCATCTCAAAGTGATCAGGCCAGTACATCGTCTCAATTAGGAA  3.CTGATAGCTCGTCAGCGAGTGGTCAGCAGCAAGAGAGTAGTGTGTCATCTCAAAGTGATCAGGCCAGTACATCGTCTCAATTAGGAA  4.CTGAT | 375.  **D**SSSAGGQQQESSVSSQSDQASTSSQLG  376.  **D**SSSASGQQQESSVSSQSDQASTSSQLG  377.  **D**SSSASGQQQESSVSSQSDQASTSSQLG  378.**D** | 3 |
| 99 | MF787745.1 | Turkey | Elazig | >MF787745.1 Anaplasma marginale isolate Bkz21 major surface protein 1 alpha (msp1a) gene, partial cds  AAATTTAGTATATTAATCTTGCGATTACACGTTTCCGTATGTTACAATCAGGCCGCCGGTGTGTAGCGTG  CTGGTTGTGTGGTTGTCCTCTTTCCCGATGTTGGGTCGTTCGTTTTACGTCTCACAAGTTTGTACGCTGT  GCCCCTGGCAGTGTAGGGTTTGTTTATTTGTGTGTGTGTTATGTCAGAGTGTGAGTCTTCCCAGCCAGCT  GATAGCTCGTCAGCGAGTGGTCAGCAGCAAGAGAGTAGTGTGTCATCTCAAAGTGATGCCAGTACATCGT  CTCAATTAGGGACTGATAGCTCGTCAGCGAGTGGTCAGCAGCAAGAGAGTAGTGTGTCATCTCAAAGTGA  TGCCAGTACATCGTCTCAATTAGGGACTGATAGCTCGTCAGCGAGTGGTCAGCAGCAAGAGAGTAGTGTG  TCATCTCAAAGTGATGCCAGTACATCGTCTCAATTAGGGACTGATAGCTCGTCAGCGAGTGGTCAGCAGC  AAGAGAGTAGTGTGTCATCTCAAAGTGATGCCAGTACATCGTCTCAATTAGGGACTGATAGCTCGTCAGC  GAGTGGTCAGCAGCAAGAGAGTGGTGTGTCATCTCAAAGTGATGCCAGTACATCGTCTCAATTAGGAGCT  GATAGCTCGTCAGCGAGTGGTCAGCAGCAAGAGAGTGGTGTGTCATCTCAAAGTGATGCCAGTACATCGT  CTCAATTAGGAGCTGATTGGCGGCAAGAGATGCGCTCCAAGGTTGCGAGTGTTGAGTACATGTTGGCTGC  TCGTGCCCTTATCTCTGTAGGGGTCTATGCTGCTCAGGGAGAGATCGCGAGATCGCTAGGGTATGCTCCC  CTGCGTGTTGCAGAAGTCGAAGAAATCGTGAGGGATAGCCTTGTACGCAGCTACTTTCATGATAGTGGCC  TTTCACTAGGCTCCATA | 1.CTGATAGCTCGTCAGCGAGTGGTCAGCAGCAAGAGAGTAGTGTGTCATCTCAAAGTGATGCCAGTACATCGTCTCAATTAGGGA  2.CTGATAGCTCGTCAGCGAGTGGTCAGCAGCAAGAGAGTAGTGTGTCATCTCAAAGTGATGCCAGTACATCGTCTCAATTAGGGA  3.CTGATAGCTCGTCAGCGAGTGGTCAGCAGCAAGAGAGTAGTGTGTCATCTCAAAGTGATGCCAGTACATCGTCTCAATTAGGGA  4.CTGATAGCTCGTCAGCGAGTGGTCAGCAGCAAGAGAGTAGTGTGTCATCTCAAAGTGATGCCAGTACATCGTCTCAATTAGGGA  5.CTGATAGCTCGTCAGCGAGTGGTCAGCAGCAAGAGAGTGGTGTGTCATCTCAAAGTGATGCCAGTACATCGTCTCAATTA-GGA  6.GCTGATAGCTCGTCAGCGAGTGGTCAGCAGCAAGAGAGTGGTGTGTCATCTCAAAGTGATGCCAGTACATCGTCTCAATTAGG | 379.  **D**SSSASGQQQESSVSSQSDASTSSQLG  380.**D**SSSASGQQQESSVSSQSDASTSSQLG  381.**D**SSSASGQQQESSVSSQSDASTSSQLG  382.**D**SSSASGQQQESSVSSQSDASTSSQLG  383.**D**SSSASGQQQESGVSSQSDASTSSQL  384.**A**DSSSASGQQQESGVSSQSDASTSSQL | 6 |
| 100 | MF787744.1 | Turkey | Elazig | >MF787744.1 Anaplasma marginale isolate Brt21 major surface protein 1 alpha (msp1a) gene, partial cds  TTGTGCTTATGGCAGACATTTCCATATACTGTGCAGTATGGTTGTGCTCCCCAATTGTTAAAATTTAGTA  TATTAATCTTGCAATTACACGTTTCCGTATGTTACAATCAGGCCGCCGGTGTGTAGCGTGCTGGTTGTGT  GGTTGTCCTCTTTCCCGATGTTGGGTCGTTCGTTTTACGTCTCACAAGTTTGTACGCTGTGCCCCTGGCA  GTGTAGGGTTTGTTTATTTGTGTGTGTGTTATGTCAGAGTGTGAGTCTTCCCAGCCAGCTGGTAGCTCGT  CAGCGAGTGGTCAGCAGCAAGAGAGTGGTGTGTCATCTCAAAGTGATGCCAGTACATCGTCTCAATTAGG  AGCTGGTAGCTCGTCAGCGAGTGGTCAGCAGCAAGAGAGTGGTGTGTCATCTCAAAGTGATGCCAGTACA  TCGTCTCAATTAGGAGCTGATAGCTCGTCAGCGAGTGGTCAGCAGCAAGAGAGTGGTGTGTCATCTCAAA  GTGATGCCAGTACATCGTCTCAATTAGGAGCTGATAGCTCGTCAGCGAGTGGTCAGCAGCAAGAGAGTGG  TGTGTCATCTCAAAGTGATGCCAGTACATCGTCTCAATTAGGAGCTGATTGGCGGCAAGAGATGCGCTCC  AAGGTTGCGAGTGTTGAGTACATGTTGGCTGCTCGTGCCCTTATCTCTGTAGGGGTCTATGCTGCTCAGG  GAGAGATCGCGAGATCGCTAGGGTATGCTCCCCTGCGTGTTGCAGAAGTCGAAGAAATCGTGAGGGATAG  CCTTGTACGCAGCTACTTTCATGATAGTGGCCTTTCACTAGGCTCCATACGACTCG | 1.AGCTGGTAGCTCGTCAGCGAGTGGTCAGCAGCAAGAGAGTGGTGTGTCATCTCAAAGTGATGCCAGTACATCGTCTCAATTAGG  2.AGCTGGTAGCTCGTCAGCGAGTGGTCAGCAGCAAGAGAGTGGTGTGTCATCTCAAAGTGATGCCAGTACATCGTCTCAATTAGG  3.AGCTGATAGCTCGTCAGCGAGTGGTCAGCAGCAAGAGAGTGGTGTGTCATCTCAAAGTGATGCCAGTACATCGTCTCAATTAGG  4.AGCTGATAGCTCGTCAGCGAGTGGTCAGCAGCAAGAGAGTGGTGTGTCATCTCAAAGTGATGCCAGTACATCGTCTCAATTAGG  5.AGCTGAT | 385.  **A**GSSSASGQQQESGVSSQSDASTSSQL  386.**A**GSSSASGQQQESGVSSQSDASTSSQL  387.**A**DSSSASGQQQESGVSSQSDASTSSQL  388.**A**DSSSASGQQQESGVSSQSDASTSSQL  389.**A**D | 4 |
| 101 | MF787743.1 | Turkey | Elazig | >MF787743.1 Anaplasma marginale isolate Brt39 major surface protein 1 alpha (msp1a) gene, partial cds  CAGTTGTTAAATTTAGTATATTAATCTTGCGATTACACGTTTCCGTATGTTACAATCAGGCCGCCGGTGT  GGTAGCGTGCTGGTTGTGTGGTTGTCCTCTTTCCCGATGTTGGGTCGTTCGTTTTACGCCTCACAAGTTT  GTACGCTGTGCCCCTGGCAGTGTAGGGTTTGTTTGTTTGTGTGTGTGTTATGTCAGAGTATGAGTCCTCT  CAGCTATCTGGTAGCTCGTCAGCGAGTGGTCAGCAGCAAGAGAGTAGTGTGTTATCTCAAAGTGGTCAGG  CCAGTACATCGTCTCAATTAGGAGCTGATAGCTCGTCAGCGAGTGGTCAGCAGCAAGAGAGTGGTGTGCC  ATCTCAAAGTGGTCAGGCCAGTACATCGTCTCAATTAGGAGCTGATAGCTCGTCAGCGAGTGGTCAGCAG  CAAGAGAGTGGTGTGTCATCTCAAAGTGATGCCAGTACATCGTCTCAATTAGGAGCTGGTTGGCGGCAAA  GGATGCGCTCCGAGGTTGCGAGTGTTGAGTACATGTTGGCTGCTCGTGCCCTTATCTCTGTAGGGGTCTA  TGCTGCTCAGGGAGAGATCGCGGGATCGCTAGGGTATGCTCCCCTGCGTGTTGCAGAAGTCGAAGAAATC  GTGAGGGATAGCCTTGTACGCAGCTACTTTCATGATAGTGGCCTT | 1.TAGCTCGTCAGCGAGTGGTCAGCAGCAAGAGAGTAGTGTGTTATCTCAAAGTGGTCAGGCCAGTACATCGTCTCAATTAGGAGCTGA  2.TAGCTCGTCAGCGAGTGGTCAGCAGCAAGAGAGTGGTGTGCCATCTCAAAGTGGTCAGGCCAGTACATCGTCTCAATTAGGAGCTGA  3.TAGCTCGTCAGCGAGTGGTCAGCAGCAAGAGAGTGGTGTGTCATCTCAAAGT-G--ATGCCAGTACATCGTCTCAATTAGGAGCTG | 390.  **S**SSASGQQQESGVPSQSGQASTSSQLGA  391.  **S**SSASGQQQESGVPSQSGQASTSSQLGA  392  **S**SSASGQQQESGVSSQSDASTSSQLGA | 3 |
| 102 | MF787742.1 | Turkey | Elazig | >MF787742.1 Anaplasma marginale isolate Krk49 major surface protein 1 alpha (msp1a) gene, partial cds  TGGTTGTGCTCCCCAATTGTTAAAATTTAGTATATTAATCTTGCGATTACACGTTCCGTATGTTACAATC  GGGCTGCCGGTGTGGTAGCGTGCTGGTTGTGTGGTTGTCCTCTTTCCCGATGTTGGGTCGTTCGTTTTAC  GTCTCACAAGTTTGTACGCTGTGCCCCTGGCAGTGTAGGGTTTGTGTGTGTGTGTGTGTGTTATGTCAGA  GTATGCGTCCAGCCAGCCAGCTGATAGCTCGTCAGCGAGTGGTGTGTTATCTCAAAGTGGTCAGGCCAGT  ACATCGTCTCAATTAGGGACTGATAGCTCGTCAGCGGGTGATCAGCAGCAAGAGAGTGGTGTGTCATCTC  AAAGTGGTCAGGCCAGTACATCGTCTCAATTAGGGACTGATAGCTCGTCAGCGGGTGATCAGCAGCAAGA  GAGTGGTGTGTCATCTCAAAGTGGTCAGGCCAGTACATCGTCTCAATTAGGGACTGATAGCTCGTCAGCG  GGTGATCAGCAGCAAGAGAGTGGTGTGTCATCTCAAAGTGGTCAGGCCAGTACATCGTCTCAATTAGGGA  CTGATAGCTCGTCAGCGGGTGATCAGCAGCAAGAGAGTGGTGTGTCATCTCAAAGTGGTCAGGCCAGTAC  ATCGTCTCAATTAGGGACTGATAGCTCGTCAGCGGGTGATCAGCAGCAAGAGAGTGGTGTGTCATCTCAA  AGTGGTCAGGCCAGTACATCGTCTCAATTAGGGACTGATAGCTCGTCAGCGGGTGATCAGCAGCAAGAGA  GTGGTGTGTCATCTCAAAGTGGTCAGGCCAGTACATCGTCTCAATTAGGGACTGATTGGCGGCAAGAGAT  GCGCTCCAAGGTTGCGAGTGTTGAGTACATTTTGGCTGCTCGTGCCCTTATCTCTGTAGGGGTCTATGCT  GCTCAGGAAGAGATCGCGAAATCGCGAGGGTATGCTCCCCTGCGTGTTGCAGAAGTCGAAGAAATCGTGA  GGGATGGCCTTGTACGCAGCCACTTTCATGATAGTGGCCTTTCACTAGGCTCCATACGACTCTTGCTT | 1.GAGTGGTGTGTTATCTCAAAGTGGTCAGGCCAGTACATCGTCTCAATTAGGGACTGATAGCTCGTCAGCGGGTGATCAGCAGCAAGA  2.GAGTGGTGTGTCATCTCAAAGTGGTCAGGCCAGTACATCGTCTCAATTAGGGACTGATAGCTCGTCAGCGGGTGATCAGCAGCAAGA  3.GAGTGGTGTGTCATCTCAAAGTGGTCAGGCCAGTACATCGTCTCAATTAGGGACTGATAGCTCGTCAGCGGGTGATCAGCAGCAAGA  4.GAGTGGTGTGTCATCTCAAAGTGGTCAGGCCAGTACATCGTCTCAATTAGGGACTGATAGCTCGTCAGCGGGTGATCAGCAGCAAGA  5.GAGTGGTGTGTCATCTCAAAGTGGTCAGGCCAGTACATCGTCTCAATTAGGGACTGATAGCTCGTCAGCGGGTGATCAGCAGCAAGA  6.GAGTGGTGTGTCATCTCAAAGTGGTCAGGCCAGTACATCGTCTCAATTAGGGACTGATAGCTCGTCAGCGGGTGATCAGCAGCAAGA  7.GAGTGGTGTGTCATCTCAAAGTGGTCAGGCCAGTACATCGTCTCAATTAGGGACTGAT | 393.  **S**GVLSQSGQASTSSQLGTDSSSAGDQQQ  394.**S**GVSSQSGQASTSSQLGTDSSSAGDQQQ  395.**S**GVSSQSGQASTSSQLGTDSSSAGDQQQ  396.**S**GVSSQSGQASTSSQLGTDSSSAGDQQQ  397.**S**GVSSQSGQASTSSQLGTDSSSAGDQQQ  398.**S**GVSSQSGQASTSSQLGTDSSSAGDQQQ  399.**S**GVSSQSGQASTSSQLGTD | 7 |
| 103 | MF377395.1 | Turkey | Elazig | >MF377395.1 Anaplasma marginale isolate t43 major surface protein 1 alpha (msp1a) gene, partial cds  TCGGTTGTGCTCCCCAATTGTTAAAATTTAGTATATTAATCTTGCGATTACACGTTCCGTATGTTACAAT  CAGGCCGTCGGTGTGGTAGCGTACTGGTTGTGTGGTTGTCCTCTTTCCCGATGTTGGGTCGTTCGTTTTA  CGTCTCACAAGTTTGTACGCTGTGCCCCTGGCAGTGTAGGGTTTGTTTGTGTGTGTGTGTGTTATGTCAG  AGTATGCGTCCAGCCAGCCAGCTGATAGCTCGTCAGCGGGTGATCAGCAGCAAGAGAGTAGTGTGTTATC  TCAAAGTGATCAGGCCAGTACATCGTCTCAATTAGGAACTGATAGCTCGTCAGCGGGTGATCAGCAGCAA  GAGAGTGGTGTGTCATCTCAAAGTGGTCAGGCCAGTACATCGTCTCAATTAGGGACTGATTGGCGGCAAG  AGATGCGCTCCAAGGTTGCGAGTGTTGAGTACATTTTGGCTGCTCGTGCCCTTATCTCTGTAGGGGTCTA  TGCTGCTCAGGGAGAGATCGCGAGATTGCAAGGGTGTGCTCCCCTGAGTGTTGTAGAAGTCGAAGAAATC  GTGAAGGATGGCCTTGTACGCAGCCACTTTCATGATAGTGGCCTTTCACTAGGCTCCATACGACTCGTGC  TTATGCAGGTTGG | 1.CTGATAGCTCGTCAGCGGGTGATCAGCAGCAAGAGAGTAGTGTGTTATCTCAAAGTGATCAGGCCAGTACATCGTCTCAATTAGGAA  2.CTGATAGCTCGTCAGCGGGTGATCAGCAGCAAGAGAGTGGTGTGTCATCTCAAAGTGGTCAGGCCAGTACATCGTCTCAATTAGGGA  3.CTGAT | 400.**D**SSSAGDQQQESSVLSQSDQASTSSQLG  401.**D**SSSAGDQQQESGVSSQSGQASTSSQLG  402.**D** | 2 |
| 104 | MF377394.1 | turkey | Elazig | >MF377394.1 Anaplasma marginale isolate t41 major surface protein 1 alpha (msp1a) gene, partial cds  TCGGTTGTGCTCCCCAATTGTTAAAATTTAGTATATTAATCTTGCAATTACACGTTTCCGTATGTTACAA  TCAGGCCGCCGGTGTGTAGCGTGCTGGTTGTGTGGTTGTCCTCTTTCCCGATGTTGGGTCGTTCGTTTTA  CGTCTCACAAGTTTGTACGCTGTGCCCCTGGCAGTGTAGGGTTTGTTTATTTGTGTGTGTGTTATGTCAG  AGTGTGAGTCTTCCCAGCCAGCTGATAGCTCGTCAGCGAGTGGTCAGCAGCAAGAGAGTGGTGTGTCATC  TCAAAGTGATGCCAGTACATCGTCTCAATTAGGAGCTGATAGCTCGTCAGCGAGTGGTCAGCAGCAAGAG  AGTGGTGTGTCATCTCAAAGTGATGCCAGTACATCGTCTCAATTAGGAGCTGATAGCTCGTCAGCGAGTG  GTCAGCAGCAAGAGAGTGGTGTGTCATCTCAAAGTGATGCCAGTACATCGTCTCAATTAGGAGCTGATAG  CTCGTCAGCGAGTGGTCAGCAGCAAGAGAGTGGTGTGCCATCTCAAAGTGGTCAGGCCAGTACATCGTCT  CAATTAGGGACTGATAGCTCGTCAGCGAGTGGTCAGCAGCAAGAGAGTGGTGTGTCATCTCAAAGTGATG  CCAGTACATCGTCTCAATTAGGAGCTGGTTGGCGGCAAGAGATGCGCCCCAAGGTTGCGAGTGTTGAGTA  CATGTTGGCTGCTCGTGCCCTTATCTCTGTAGGGGTCTATGCTGCTCAGGGAGAGATCGCGAGATCGCTA  GGGTATGCTCCCCTGCGTGTTGCAGAAGTCGAAGAAATCGTGAGGGATAGCCTTGTACGCAGCTACTTTC  ATGATAGTGGCCTTTCACTAGGCTCCATACGACTCGTGCTTATGCAGGTTGG | 1.AGCTGATAGCTCGTCAGCGAGTGGTCAGCAGCAAGAGAGTGGTGTGTCATCTCAAAGTGATGCCAGTACATCGTCTCAATTAGG  2.AGCTGATAGCTCGTCAGCGAGTGGTCAGCAGCAAGAGAGTGGTGTGTCATCTCAAAGTGATGCCAGTACATCGTCTCAATTAGG  3.AGCTGATAGCTCGTCAGCGAGTGGTCAGCAGCAAGAGAGTGGTGTGTCATCTCAAAGTGATGCCAGTACATCGTCTCAATTAGG  4.AGCTGATAGCTCGTCAGCGAGTGGTCAGCAGCAAGAGAGTGGTGTGCCATCTCAAAGTGGTCAGGCCAGTACATCGTCTCAATTAGG  5.GACTGATAGCTCGTCAGCGAGTGGTCAGCAGCAAGAGAGTGGTGTGTCATCTCAAAGTGATGCCAGTACATCGTCTCAATTAGG  6.AGCTG | 403.  **A**DSSSASGQQQESGVSSQSDASTSSQL  404.  **A**DSSSASGQQQESGVSSQSDASTSSQL  405.  **A**DSSSASGQQQESGVSSQSDASTSSQL  406.**A**DSSSASGQQQESGVPSQSGQASTSSQL  407.**T**DSSSASGQQQESGVSSQSDASTSSQL  408. **A** | 5 |
| 105 | MF377393.1 | Turkey | Elazig | >MF377393.1 Anaplasma marginale isolate s175 major surface protein 1 alpha (msp1a) gene, partial cds  TCGGTTGTGCTCCCCAATTGTTAAAATTTAGTATATTAATCTTGCGATTACACGTTCCGTATGTTACAAT  CAGGCCGTCGGTGTGGTAGCGTACTGGTTGTGTGGTTGTCCTCTTTCCCGATGTTGGGTCGTTCGTTTTA  CGTCTCACAAGTTTGTACGCTGTGCCCCTGGCAGTGTAGGGTTTGTTTGTGTGTGTGTGTGTTATGTCAG  AGTATGCGTCCAGCCAGCCAGCTGATAGCTCGTCAGCGGGTGATCAGCAGCAAGAGAGTAGTGTGTTATC  TCAAAGTGATCAGGCCAGTACATCGTCTCAATTAGGAACTGATAGCTCGTCAGCGGGTGATCAGCAGCAA  GAGAGTGGTGTGTCATCTCAAAGTGGTCAGGCCAGTACATCGTCTCAATTAGGGACTGATTGGCGGCAAG  AGATGCGCTCCAAGGTTGCGAGTGTTGAGTACATTTTGGCTGCTCGTGCCCTTATCTCTGTAGGGGTCTA  TGCTGCTCAGGGAGAGATCGCGAGATTGCAAGGGTGTGCTCCCCTGAGTGTTGTAGAAGTCGAAGAAATC  GTGAAGGATGGCCTTGTACGCAGCCACTTTCATGATAGTGGCCTTTCACTAGGCTCCATACGACTCGTGC  TTATGCAGGTTGG | 1.CTGATAGCTCGTCAGCGGGTGATCAGCAGCAAGAGAGTAGTGTGTTATCTCAAAGTGATCAGGCCAGTACATCGTCTCAATTAGGAA  2.CTGATAGCTCGTCAGCGGGTGATCAGCAGCAAGAGAGTGGTGTGTCATCTCAAAGTGGTCAGGCCAGTACATCGTCTCAATTAGGA  3.CTGAT | 409.  **D**SSSAGDQQQESSVLSQSDQASTSSQLG  410.**D**SSSAGDQQQESGVSSQSGQASTSSQLG  411.D | 2 |
| 106 | MF377392.1 | Turkey | Elazig | >MF377392.1 Anaplasma marginale isolate s67 major surface protein 1 alpha (msp1a) gene, partial cds  CGGTTGTGCTCCCCAATTGTTAAAATTTAGTATATTAATCTTGCGATTACACGTTCCGTATGTTACAATC  GGGCTGCCGGTGTGGTAGCGTGCTGGTTGTGTGGTTGTCCTCTTTCCCGATGTTGGGTCGTTCGTTTTAC  GTCTCACAAGTTTGTACGCTGTGCCCCTGGCAGTGTAGGGTTTGTTTGTGTGTGTGTTATGTCAGAGTAT  GTGTCCAGCCAGCCAGCTGATAGCTCGTCAGCGAGTGGTCAGCAGCAAGAGAGTGGTGTGTCATCTCAAA  GTGATGCCAGTACATCGTCTCGATTAGGAGCTGATTGGCGGCAAGAGATGCGCTCCAAGGTTGCGAGTGT  TGAGTACATGTTGGCTGCTCGTGCCCTTATCTCTGTAGGGGTCTATGCTGCTCAGGGAGAGATCGCGAGA  TCGCTAGGGTATGCTCCCCTGCGTGTTGCAGAAGTCGAAGAAATCGTGAGGGATAGCCTTGTACGCAGCT  ACTTTCATGATAGTGGCCTTTCACTAGGCTCCATACGACTCGTGCT | N/A | N/A | N/A |
| 107 | MF377391.1 | Turkey | Elazig | >MF377391.1 Anaplasma marginale isolate s22 major surface protein 1 alpha (msp1a) gene, partial cds  TCGGTTGTGCTCCCCAATTGTTAAAATTTAGTATATTAATCTTGCGATTACACGTTCCGTATGTTACAAT  CAGGCCGTCGGTGTGGTAGCGTACTGGTTGTGTGGTTGTCCTCTTTCCCGATGTTGGGTCGTTCGTTTTA  CGTCTCACAAGTTTGTACGCTGTGCCCCTGGCAGTGTAGGGTTTGTTTGTGTGTGTGTGTGTTATGTCAG  AGTATGCGTCCAGCCAGCCAGCTGATAGCTCGTCAGCGGGTGATCAGCAGCAAGAGAGTAGTGTGTTATC  TCAAAGTGATCAGGCCAGTACATCGTCTCAATTAGGAACTGATAGCTCGTCAGCGGGTGATCAGCAGCAA  GAGAGTGGTGTGTCATCTCAAAGTGGTCAGGCCAGTACATCGTCTCAATTAGGGACTGATTGGCGGCAAG  AGATGCGCTCCAAGGTTGCGAGTGTTGAGTACATTTTGGCTGCTCGTGCCCTTATCTCTGTAGGGGTCTA  TGCTGCTCAGGGAGAGATCGCGAGATTGCAAGGGTGTGCTCCCCTGAGTGTTGTAGAAGTCGAAGAAATC  GTGAAGGATGGCCTTGTACGCAGCCACTTTCATGATAGTGGCCTTTCACTAGGCTCCATACGACTCGTGC  TTATGCAGGTTGG | 1.CTGATAGCTCGTCAGCGGGTGATCAGCAGCAAGAGAGTAGTGTGTTATCTCAAAGTGATCAGGCCAGTACATCGTCTCAATTAGGAA  2.TGATAGCTCGTCAGCGGGTGATCAGCAGCAAGAGAGTGGTGTGTCATCTCAAAGTGGTCAGGCCAGTACATCGTCTCAATTAGGGA  3.CTGAT | 412.  **D**SSSAGDQQQESSVLSQSDQASTSSQLG  413.  **D**SSSAGDQQQESGVSSQSGQASTSSQLG  414.D | 2 |
| 108 | MF377390.1 | Turkey | Elazig | >MF377390.1 Anaplasma marginale isolate s18 major surface protein 1 alpha (msp1a) gene, partial cds  TCGGTTGTGCTCCCCAATTGTTAAAATTTAGTATATTAATCTTGCGATTACACGTTCCGTATGTTACAAT  CAGGCCGTCGGTGTGGTAGCGTACTGGTTGTGTGGTTGTCCTCTTTCCCGATGTTGGGTCGTTCGTTTTA  CGTCTCACAAGTTTGTACGCTGTGCCCCTGGCAGTGTAGGGTTTGTTTGTGTGTGTGTGTGTTATGTCAG  AGTATGCGTCCAGCCAGCCAGCTGATAGCTCGTCAGCGGGTGATCAGCAGCAAGAGAGTAGTGTGTTATC  TCAAAGTGATCAGGCCAGTACATCGTCTCAATTAGGAACTGATAGCTCGTCAGCGGGTGATCAGCAGCAA  GAGAGTGGTGTGTCATCTCAAAGTGGTCAGGCCAGTACATCGTCTCAATTAGGGACTGATTGGCGGCAAG  AGATGCGCTCCAAGGTTGCGAGTGTTGAGTACATTTTGGCTGCTCGTGCCCTTATCTCTGTAGGGGTCTA  TGCTGCTCAGGGAGAGATCGCGAGATTGCAAGGGTGTGCTCCCCTGAGTGTTGTAGAAGTCGAAGAAATC  GTGAAGGATGGCCTTGTACGCAGCCACTTTCATGATAGTGGCCTTTCACTAGGCTCCATACGACTCGTGC  TTATGCAGGTTGG | 1.CTGATAGCTCGTCAGCGGGTGATCAGCAGCAAGAGAGTAGTGTGTTATCTCAAAGTGATCAGGCCAGTACATCGTCTCAATTAGGAA  2.CTGATAGCTCGTCAGCGGGTGATCAGCAGCAAGAGAGTGGTGTGTCATCTCAAAGTGGTCAGGCCAGTACATCGTCTCAATTAGGGA  3.CTGAT | 415.**D**SSSAGDQQQESSVLSQSDQASTSSQLG  416.**D**SSSAGDQQQESGVSSQSGQASTSSQLG  417.D | 2 |
| 109 | MF377389.1 | Turkey | Elazig | >MF377389.1 Anaplasma marginale isolate s13 major surface protein 1 alpha (msp1a) gene, partial cds  TCGGTTGTGCTCCCCAATTGTTAAAATTTAGTATATTAATCTTGCGATTACACGTTCCGTATGTTACAAT  CAGGCCGTCGGTGTGGTAGCGTACTGGTTGTGTGGTTGTCCTCTTTCCCGATGTTGGGTCGTTCGTTTTA  CGTCTCACAAGTTTGTACGCTGTGCCCCTGGCAGTGTAGGGTTTGTTTGTGTGTGTGTGTGTTATGTCAG  AGTATGCGTCCAGCCAGCCAGCTGATAGCTCGTCAGCGGGTGATCAGCAGCAAGAGAGTAGTGTGTTATC  TCAAAGTGATCAGGCCAGTACATCGTCTCAATTAGGAACTGATAGCTCGTCAGCGGGTGATCAGCAGCAA  GAGAGTGGTGTGTCATCTCAAAGTGGTCAGGCCAGTACATCGTCTCAATTAGGGACTGATTGGCGGCAAG  AGATGCGCTCCAAGGTTGCGAGTGTTGAGTACATTTTGGCTGCTCGTGCCCTTATCTCTGTAGGGGTCTA  TGCTGCTCAGGGAGAGATCGCGAGATTGCAAGGGTGTGCTCCCCTGAGTGTTGTAGAAGTCGAAGAAATC  GTGAAGGATGGCCTTGTACGCAGCCACTTTCATGATAGTGGCCTTTCACTAGGCTCCATACGACTCGTGC  TTATGCAGGTTGG | 1.CTGATAGCTCGTCAGCGGGTGATCAGCAGCAAGAGAGTAGTGTGTTATCTCAAAGTGATCAGGCCAGTACATCGTCTCAATTAGGAA  2.CTGATAGCTCGTCAGCGGGTGATCAGCAGCAAGAGAGTGGTGTGTCATCTCAAAGTGGTCAGGCCAGTACATCGTCTCAATTAGGGA  3.CTGAT | 418.**D**SSSAGDQQQESSVLSQSDQASTSSQLG  419.**D**SSSAGDQQQESGVSSQSGQASTSSQLG  420.D | 2 |
| 110 | MF377388.1 | Turkey | Elazig | >MF377388.1 Anaplasma marginale isolate s7 major surface protein 1 alpha (msp1a) gene, partial cds  CGGTTGTGCTCCCCAATTGTTAAAATTTAGTATATTAATCTTGCGATTACACGTTCCGTATGTTACAATC  AGGCCGTCGGTGTGGTAGCGTACTGGTTGTGTGGTTGTCCTCTTTCCCGATGTTGGGTCGTTCGTTTTAC  GTCTCACAAGTTTGTACGCTGTGCCCCTGGCAGTGTAGGGTTTGTTTGTGTGTGTGTGTGTTATGTCAGA  GTATGCGTCCAGCCAGCCAGCTGATAGCTCGTCAGCGGGTGATCAGCAGCAAGAGAGTAGTGTGTTATCT  CAAAGTGATCAGGCCAGTACATCGTCTCAATTAGGAACTGATAGCTCGTCAGCGGGTGATCAGCAGCAAG  AGAGTGGTGTGTCATCTCAAAGTGGTCAGGCCAGTACATCGTCTCAATTAGGGACTGATTGGCGGCAAGA  GATGCGCTCCAAGGTTGCGAGTGTTGAGTACATTTTGGCTGCTCGTGCCCTTATCTCTGTAGGGGTCTAT  GCTGCTCAGGGAGAGATCGCGAGATTGCAAGGGTGTGCTCCCCTGAGTGTTGTAGAAGTCGAAGAAATCG  TGAAGGATGGCCTTGTACGCAGCCACTTTCATGATAGTGGCCTTTCACTAGGCTCCATACGACTCGTGCT  TATGCAGGTTGG | 1.CTGATAGCTCGTCAGCGGGTGATCAGCAGCAAGAGAGTAGTGTGTTATCTCAAAGTGATCAGGCCAGTACATCGTCTCAATTAGGAA  2.CTGATAGCTCGTCAGCGGGTGATCAGCAGCAAGAGAGTGGTGTGTCATCTCAAAGTGGTCAGGCCAGTACATCGTCTCAATTAGGGA  3.CTGAT | 421.**D**SSSAGDQQQESSVLSQSDQASTSSQLG  422.**D**SSSAGDQQQESGVSSQSGQASTSSQLG  423.D | 2 |
| 111 | MG983517.1 |  |  | >MG983517.1 Anaplasma marginale isolate kr78 major surface protein 1 alpha gene, partial cds  TGTGCAGTACGGTTGTGCTCCCCAATTGTTAAAATTTAGTATATTAATCTTGCGATTACACGTTCCGTAT  GTTACAATCGGGCTGCCGGTGTGGTAGCGTGCTGGTTGTGTGGTTGTCCTCTTTCCCGATGTTGGGTCGT  TCGTTTTACGTCTCACAAGTTTGTACGCTGTGCCCCTGGCAGTGTAGGGTTTGTTTGTGTGTGTGTGTGT  TATGTCAGAGTGTGTGTCCAGCCAGCCAGCTGATAGCTCGTCAGCGGGTGGTGTGTTATCTCAAAGTGGT  CAGGCCAGTACATCGTCTCAATTAGGGACTGATAGCTCGTCAGCGGGTGATCAGCAGCAAGAGAGTGGTG  TGTCATCTCAAAGTGGTCAGGCCAGTACATCGTCTCAATTAGGGACTGATAGCTCGTCAGCGGGTGATCA  GCAGCAAGAGAGTGGTGTGTCATCTCAAAGTGGTCAGGCCAGTACATCGTCTCAATTAGGGACTGATAGC  TCGTCAGCGGGTGATCAGCAGCAAGAGAGTGGTGTGTCATCTCAAAGTGGTCAGGCCAGTACATCGTCTC  AATTAGGAACTGATGGGTGGCAAGAGATGCGCTCCAAGGTTGCGAGTGTTGAGTACATTTTGGCTGCTCG  TGCCCTTATTTCTGTAGGGGTCTATGCTGCTCAGGGAGAGATCGCGAGATCGCAAGGGTGTGCTCCCCTG  CGTGTTGCAAAAGTCGAAGAAATCGTGAGGGATAGCCTTGTACGCAGCCACTTTCATGATAGTGGCCTTT  CACTAGGCTCCATACGACTCGTGCTTATGCAGGTTGGGGATAAGTTGGG | 1.GTGGTGTGTTATCTCAAAGTGGTCAGGCCAGTACATCGTCTCAATTAGGGACTGATAGCTCGTCAGCGGGTGATCAGCAGCAAGAGA  2.GTGGTGTGTCATCTCAAAGTGGTCAGGCCAGTACATCGTCTCAATTAGGGACTGATAGCTCGTCAGCGGGTGATCAGCAGCAAGAGA  3.GTGGTGTGTCATCTCAAAGTGGTCAGGCCAGTACATCGTCTCAATTAGGGACTGATAGCTCGTCAGCGGGTGATCAGCAGCAAGAGA  4.GTGGTGTGTCATCTCAAAGTGGTCAGGCCAGTACATCGTCTCAATTAGGAACTGAT | 424.**G**VLSQSGQASTSSQLGTDSSSAGDQQQE  425.**G**VSSQSGQASTSSQLGTDSSSAGDQQQE  426.**G**VSSQSGQASTSSQLGTDSSSAGDQQQE  427.**G**VSSQSGQASTSSQLGTD | 4 |
| 112 | MG983516.1 | Turkey | Elazig | >MG983516.1 Anaplasma marginale isolate kr58 major surface protein 1 alpha gene, partial cds  CCAATTGTTAAAATTTAGTATATTAATCTTGCGATTACACGTTCCGTATGTTACAATCGGGCTGCCGGTG  TGGTAGCGTGCTGGTTGTGTGGTTGTCCTCTTTCCCGATGTTGGGTCGTTCGTTTTACGTCTCACAAGTT  TGTACGCTGTGCCCCTGGCAGTGTAGGGTTTGTTTGTGTGTGTGTGTGTTATGTCAGAGTGTGTGTCCAG  CCAGCCAGCTGATAGCTCGTCAGCGGGTGGTGTGTTATCTCAAAGTGGTCAGGCCAGTACATCGTCTCAA  TTAGGGACTGATAGCTCGTCAGCGGGTGATCAGCAGCAAGAGAGTGGTGTGTCATCTCAAAGTGGTCAGG  CCAGTACATCGTCTCAATTAGGGACTGATAGCTCGTCAGCGGGTGATCAGCAGCAAGAGAGTGGTGTGTC  ATCTCAAAGTGGTCAGGCCAGTACATCGTCTCAATTAGGGACTGATAGCTCGTCAGCGGGTGATCAGCAG  CAAGAGAGTGGTGTGTCATCTCAAAGTGGTCAGGCCAGTACATCGTCTCAATTAGGAACTGATGGGTGGC  AAGAGATGCGCTCCAAGGTTGCGAGTGTTGAGTACATTTTGGCTGCTCGTGCCCTTATTTCTGTAGGGGT  CTATGCTGCTCAGGGAGAGATCGCGAGATCGCAAGGGTGTGCTCCCCTGCGTGTTGCAAAAGTCGAAGAA  ATCGTGAGGGATAGCCTTGTACGCAGCCACTTTCATGATAGTGGCCTTTCACTAGGCTCCATACGACTCG  TGCTTATGCAGGTTGGGGATAAGTTGGG | 1.GTGGTGTGTTATCTCAAAGTGGTCAGGCCAGTACATCGTCTCAATTAGGGACTGATAGCTCGTCAGCGGGTGATCAGCAGCAAGAGA  2.GTGGTGTGTCATCTCAAAGTGGTCAGGCCAGTACATCGTCTCAATTAGGGACTGATAGCTCGTCAGCGGGTGATCAGCAGCAAGAGA  3.GTGGTGTGTCATCTCAAAGTGGTCAGGCCAGTACATCGTCTCAATTAGGGACTGATAGCTCGTCAGCGGGTGATCAGCAGCAAGAGA  4.GTGGTGTGTCATCTCAAAGTGGTCAGGCCAGTACATCGTCTCAATTAGGAACTGAT | 428.**G**VLSQSGQASTSSQLGTDSSSAGDQQQE  429.**G**VSSQSGQASTSSQLGTDSSSAGDQQQE  430.**G**VSSQSGQASTSSQLGTDSSSAGDQQQE  431.**G**VSSQSGQASTSSQLGTD | 4 |
| 113 | MG983515.1 | Turkey | Elazig | >MG983515.1 Anaplasma marginale isolate kr37 major surface protein 1 alpha gene, partial cds  TACGGTTGTGCTCCCCAATTGTTAAAATTTAGTATATTAATCTTGCGATTACACGTTCCGTATGTTACAA  TCAGGCCGTCGGTGTGGTAGCGTACCTGGTTGTGTGGTTGTCCTCTTTCCCGATGTTGGGTCGTTCGTTT  TACGTCTCACAAGTTTGTACGCTGTGCCCCTGGCAGTGTAGGGTTTGTTTGTGTGTGTGTGTGTTATGTC  AGAGTATGCGTCCAGCCAGCCAGCTGATAGCTCGTCAGCGGGTGATCAGCAGCAAGAGAGTAGTGTGTTA  TCTCAAAGTGATCAGGCCAGTACATCGTCTCAATTAGGAACTGATAGCTCGTCAGCGGGTGATCAGCAGC  AAGAGAGTGGTGTGTCATCTCAAAGTGGTCAGGCCAGTACATCGTCTCAATTAGGGACTGATTGGCGGCA  AGAGATGCGCTCCAAGGTTGCGAGTGTTGAGTACATTTTGGCTGCTCGTGCCCTTATCTCTGTAGGGGTC  TATGCTGCTCAGGGAGAGATCGCGAGATTGCAAGGGTGTGCTCCCCTGAGTGTTGTAGAAGTCGAAGAAA  TCGTGAAGGATGGCCTTGTACGCAGCCACTTTCATGATAGTGGCCTTTCACTAGGCTCCATACGACTCGT  GCTTATGCAGGTTGGGGATAAGTTGGG | 1.CTGATAGCTCGTCAGCGGGTGATCAGCAGCAAGAGAGTAGTGTGTTATCTCAAAGTGATCAGGCCAGTACATCGTCTCAATTAGGAA  2.CTGATAGCTCGTCAGCGGGTGATCAGCAGCAAGAGAGTGGTGTGTCATCTCAAAGTGGTCAGGCCAGTACATCGTCTCAATTAGGGA  3.CTGAT | 432.**D**SSSAGDQQQESSVLSQSDQASTSSQLG  433.**D**SSSAGDQQQESGVSSQSGQASTSSQLG  434.D | 2 |
| 114 | MG983514.1 | Turkey | Elazig | >MG983514.1 Anaplasma marginale isolate kr7 major surface protein 1 alpha gene, partial cds  GTACGGTTGTGCTCCCCAATTGTTAAAATTTAGTATATTAATCTTGCGATTACACGTTCCGTATGTTACA  ATCGGGCTGCCGGTGTGGTAGCGTGCTGGTTGTGTGGTTGTCCTCTTTCCCGATGTTGGGTCGTTCGTTT  TACGTCTCACAAGTTTGTACGCTGTGCCCCTGGCAGTGTAGGGTTTGTTTGTGTGTGTGTGTGTTATGTC  AGAGTGTGTGTCCAGCCAGCCAGCTGATAGCTCGTCAGCGGGTGGTGTGTTATCTCAAAGTGGTCAGGCC  AGTACATCGTCTCAATTAGGGACTGATAGCTCGTCAGCGGGTGATCAGCAGCAAGAGAGTGGTGTGTCAT  CTCAAAGTGGTCAGGCCAGTACATCGTCTCAATTAGGGACTGATAGCTCGTCAGCGGGTGATCAGCAGCA  AGAGAGTGGTGTGTCATCTCAAAGTGGTCAGGCCAGTACATCGTCTCAATTAGGGACTGATAGCTCGTCA  GCGGGTGATCAGCAGCAAGAGAGTGGTGTGTCATCTCAAAGTGGTCAGGCCAGTACATCGTCTCAATTAG  GAACTGATGGGTGGCAAGAGATGCGCTCCAAGGTTGCGAGTGTTGAGTACATTTTGGCTGCTCGTGCCCT  TATTTCTGTAGGGGTCTATGCTGCTCAGGGAGAGATCGCGAGATCGCAAGGGTGTGCTCCCCTGCGTGTT  GCAAAAGTCGAAGAAATCGTGAGGGATAGCCTTGTACGCAGCCACTTTCATGATAGTGGCCTTTCACTAG  GCTCCATACGACTCGTGCTTATGCAGGTTGGGGATAAGTTGGG | 1.GTGGTGTGTTATCTCAAAGTGGTCAGGCCAGTACATCGTCTCAATTAGGGACTGATAGCTCGTCAGCGGGTGATCAGCAGCAAGAGA  2.GTGGTGTGTCATCTCAAAGTGGTCAGGCCAGTACATCGTCTCAATTAGGGACTGATAGCTCGTCAGCGGGTGATCAGCAGCAAGAGA  3.GTGGTGTGTCATCTCAAAGTGGTCAGGCCAGTACATCGTCTCAATTAGGGACTGATAGCTCGTCAGCGGGTGATCAGCAGCAAGAGA  4.GTGGTGTGTCATCTCAAAGTGGTCAGGCCAGTACATCGTCTCAATTAGGAACTGAT | 435.**G**VLSQSGQASTSSQLGTDSSSAGDQQQE  436.**G**VSSQSGQASTSSQLGTDSSSAGDQQQE  437.**G**VSSQSGQASTSSQLGTDSSSAGDQQQE  438.**G**VSSQSGQASTSSQLGTD | 4 |
| 115 | MG983513.1 | Turkey | Elazig | >MG983513.1 Anaplasma marginale isolate kr3 major surface protein 1 alpha gene, partial cds  CTGTGCGTACGGTTGTGCTCCCCAATTGTTAAAATTTAGTATATTAATCTTGCGATTACACGTTCCGTAT  GTTACAATCAGGCCGTCGGTGTGGTAGCGTACCTGGTTGTGTGGTTGTCCTCTTTCCCGATGTTGGGTCG  TTCGTTTTACGTCTCACAAGTTTGTACGCTGTGCCCCTGGCAGTGTAGGGTTTGTTTGTGTGTGTGTGTG  TTATGTCAGAGTATGCGTCCAGCCAGCCAGCTGATAGCTCGTCAGCGGGTGATCAGCAGCAAGAGAGTAG  TGTGTTATCTCAAAGTGATCAGGCCAGTACATCGTCTCAATTAGGAACTGATAGCTCGTCAGCGGGTGAT  CAGCAGCAAGAGAGTGGTGTGTCATCTCAAAGTGGTCAGGCCAGTACATCGTCTCAATTAGGGACTGATT  GGCGGCAAGAGATGCGCTCCAAGGTTGCGAGTGTTGAGTACATTTTGGCTGCTCGTGCCCTTATCTCTGT  AGGGGTCTATGCTGCTCAGGGAGAGATCGCGAGATTGCAAGGGTGTGCTCCCCTGAGTGTTGTAGAAGTC  GAAGAAATCGTGAAGGATGGCCTTGTACGCAGCCACTTTCATGATAGTGGCCTTTCACTAGGCTCCATAC  GACTCGTGCTTATGCAGGTTGGGGATAAGTTGGG | 1.CTGATAGCTCGTCAGCGGGTGATCAGCAGCAAGAGAGTAGTGTGTTATCTCAAAGTGATCAGGCCAGTACATCGTCTCAATTAGGAA  2.CTGATAGCTCGTCAGCGGGTGATCAGCAGCAAGAGAGTGGTGTGTCATCTCAAAGTGGTCAGGCCAGTACATCGTCTCAATTAGGGA  3.CTGAT | 439.**D**SSSAGDQQQESSVLSQSDQASTSSQLG  440.**D**SSSAGDQQQESGVSSQSGQASTSSQLG  441.D | 2 |
| 116 | OQ384912.1 | South Africa: Mpumalanga |  | >OQ384912.1 Anaplasma marginale isolate 42c major surface protein 1A (msp1a) gene, partial cds  ATGGCTGCGCGCCGCAGCTGCTGAAATTTAGCATTCTGATTCTGCGCCTGCATGTGCCGTATGTGACCAT  TGGCCTGCCGGTGTGGCGCGCGGGCTGCGTGGTGGTGCTGTTTCCGGATGTGGGCAGCTTTGTGCTGCGC  CTGACCAGCCTGTATGCGGTGCCGCTGGCGGTGGGCCTGTTTGTGTGCGTGTGCGTGATGAGCGAATATG  TGAGCAGCCAGCCGGCGGATAGCAGCAGCGCGAGCGGCGTGAGCAGCCAGAGCGGCCAGGCGAGCACCAG  CAGCCAGCTGGGCACCGATTGGTGGCAGGAAATGCGCAGCAAAGTGGCGAGCGTGGAATATATTCTGGCG  GCGCGCGCGCTGATTAGCGTGGGCGTGTATGCGGCGCAGGAAGGCATTGCGAAAAGCCTGGGCTATACCC  CGCTGCGCGTGGCGGAAGTGGAAGAAATTGTGCGCGATGGCCTGGTGCGCAGCCATTTTCATGATAGCGG  CCTGAGCCTGGGCAGCATTCGCCTGGTGCTGATGCAGGTGGGCGATAAACTGGGCCTGCAGGGC | NULL | NULL | No Repeats Found |
| 117 | OQ384909.1 | South Africa: Mpumalanga |  | >OQ384909.1 Anaplasma marginale isolate 41c major surface protein 1A (msp1a) gene, partial cds  ATGGCTGCGCGCCGCAGCTGCTGAAATTTAGCATTCTGATTCTGCGCCTGCATGTGCCGTATGTGACCAT  TGGCCTGCCGGTGTGGCGCGCGGGCTGCGTGGTGGTGCTGTTTCCGGATGTGGGCAGCTTTGTGCTGCGC  CTGACCAGCCTGTATGCGGTGCCGCTGGCGGTGGGCCTGTTTGTGTGCGTGTGCGTGATGAGCGAATATG  TGAGCAGCCAGCCGGCGGATAGCAGCAGCGCGAGCGGCGTGAGCAGCCAGAGCGGCCAGGCGAGCACCAG  CAGCCAGCTGGGCACCGATAGCAGCAGCGCGGGCGATCAGCAGCAGGGCAGCGGCGTGAGCAGCCAGAGC  GGCCAGGCGAGCACCAGCAGCCAGCTGGGCACCGATAGCAGCAGCGCGGGCGATCAGCAGCAGGGCAGCG  GCGTGAGCAGCCAGAGCGGCCAGGCGAGCACCAGCAGCCAGCTGGGCACCGATAGCAGCAGCGCGGGCGA  TCAGCAGCAGGGCAGCGGCGTGAGCAGCCAGAGCGGCCAGGCGAGCACCAGCAGCCAGCTGGGCACCGAT  AGCAGCAGCGCGGGCGATCAGCAGCAGGGCAGCGGCGTGAGCAGCCAGAGCGGCCAGGCGAGCACCAGCA  GCCAGCTGGGCACCGATAGCAGCAGCGCGAGCGGCCAGCAGCAGGAAAGCAGCGTGAGCAGCCAGAGCGG  CCAGGCGAGCACCAGCAGCCAGCTGGGCACCGATTGGCGCCAGGAAATGCGCAGCAAAGTGGCGAGCGTG  GAATATATTCTGGCGGCGCGCGCGCTGATTAGCGTGGGCGTGTATGCGGCGCAGGAAGGCATTGCGAAAA  GCCTGGGCTATACCCCGCTGCGCGTGGCGGAAGTGGAAGAAATTGTGCGCGATGGCCTGGTGCGCAGCCA  TTTTCATGATAGCGGCCTGAGCCTGGGCAGCATTCGCCTGGTGCTGATGCAGGTGGGCGATAAACTGGGC  CTGCAGGGC | NULL | NULL | No Repeats Found |
| 118 | OQ384906.1 | South Africa: Mpumalanga |  | >OQ384906.1 Anaplasma marginale isolate 40 major surface protein 1A (msp1a) gene, partial cds  ATGGCTGCGCGCCGCAGCTGCTGAAATTTAGCATTCTGATTCTGCGCCTGCATGTGCCGTATGTGACCAT  TGGCCTGCCGGTGTGGCGCGCGGGCTGCGTGGTGGTGCTGTTTCCGGATGTGGGCAGCTTTGTGCTGCGC  CTGACCAGCCTGTATGCGGTGCCGCTGGCGGTGGGCCTGTTTGTGTGCGTGTGCGTGATGAGCGAATGCG  TGTTTAGCCAGCCGGCGGATAGCAGCAGCGCGGGCAACCAGCAGCAGGAAAGCAGCGTGAGCAGCCAGAG  CGGCGCGAGCACCAGCAGCCAGCTGGGCACCGATAGCAGCAGCGCGGGCGATCAGCAGCAGGAAAGCAGC  GTGCTGAGCCCGAGCGGCCAGGCGAGCACCAGCAGCCAGAGCGGCACCGATAGCAGCAGCGCGAGCGGCC  AGCAGCAGGAAAGCAGCGTGCTGAGCCAGAGCAGCCAGGCGAGCACCAGCAGCCAGCTGGGCGCGGATAG  CAGCAGCGCGGGCAACCAGCAGCAGGAAAGCAGCGTGAGCAGCCAGAGCGATGCGAGCACCAGCAGCCAG  CTGGGCGCGGATAGCAGCAGCGCGGGCAACCAGCAGCAGGAAAGCAGCGTGAGCAGCCAGAGCGATGCGA  GCACCAGCAGCCAGCTGGGCACCGATTGGCGCCAGGATGTGCGCAGCAAAGTGGCGAGCGTGGAATATAT  TCTGGCGGCGCGCGCGCTGATTAGCGTGGGCGTGTATGCGGCGCAGAGCATGATTGCGCAGAGCCAGGGC  TGCGCGAGCCTGAGCGTGGCGGAAGTGGAAGAAATTGTGCGCGATAGCCTGGTGCGCAGCCATTTTCATG  ATAGCGGCCTGAGCCTGGGCAGCATTCGCCTGGTGCTGATGCAGGTGGGCGATAAACTGGGCCTGCAGGG  C | 1.GATAGCAGCAGCGCGGGCAACCAGCAGCAGGAAAGCAGCGTGAGCAGCCAGAGCG—GCGCGAGCACCAGCAGCCAGCTGGGCACC    2.GATAGCAGCAGCGCGGGCGATCAGCAGCAGGAAAGCAGCGTGCTG-AGCCCGAGCGGCCAGGCGAGCACCAGCAGCCAG-AGCGGCACC    3.GATAGCAGCAGCGCGAGCGGCCAGCAGCAGGAAAGCAGCGTGCTG-AGCCAGAGCAGCCAGGCGAGCACCAGCAGCCAGCTGGGCGCG    4.GATAGCAGCAGCGCGGGCAACCAGCAGCAGGAAAGCAGCGTGAGCAGCCAGAGCG-ATGCGAGCACCAGCAGCCAGCTGGGCGCG  5.GATAGCAGCAGCGCGGGCAACCAGCAGCAGGAAAGCAGCGTGAGCAGCCAGAGCG-ATGCGAGCACCAGCAGCCAGCTGGGCACC | 442.DSSSAGNQQQESSVSSQSGASTSSQLGT  443.DSSSAGDQQQESSVLSPSGQASTSSQSGT  444.DSSSASGQQQESSVLSQSSQASTSSQLGA  445.DSSSAGNQQQESSVSSQSDASTSSQLGA  446.DSSSAGNQQQESSVSSQSDASTSSQLGT | 5 |
| 119 | OQ384905.1 | SouthAfrica:Mpumalanga |  | >OQ384905.1Anaplasma marginale isolate 39 major surface protein 1A (msp1a) gene, partial cds  ATGGCTGCGCGCCGCAGCTGCTGAAATTTAGCATTCTGATTCTGCGCCTGCATGTGCCGTATGTGACCAT  TGGCCTGCCGGTGTGGCGCGCGGGCTGCGTGGTGGTGCTGTTTCCGGATGTGGGCAGCTTTGTGCTGCGC  CTGACCAGCCTGTATGCGGTGCCGCTGGCGGTGGGCCTGTTTGTGTGCGTGTGCGTGATGAGCGAATGCG  GCTTTCTGCAGCCGACCGATAGCAGCAGCGCGAGCGGCCAGCAGCAGGAAAGCAGCGTGCTGCCGCAGAG  CGATGCGAGCACCAGCAGCCAGCTGGGCGCGGATAGCAGCAGCGCGGGCAACCAGCAGCAGGAAAGCAGC  GTGAGCAGCCAGAGCGATGCGAGCACCAGCAGCCAGAGCGGCGCGGATAGCAGCAGCGCGAGCGGCCAGC  AGCAGGAAAGCAGCGTGCTGAGCCAGAGCAGCCAGGCGAGCACCAGCAGCCAGCTGGGCGCGGATAGCAG  CAGCGCGGGCAACCAGCAGCAGGAAAGCAGCGTGAGCAGCCAGAGCGATGCGAGCACCAGCAGCCAGCTG  GGCGCGGATAGCAGCAGCGCGGGCAACCAGCAGCAGGAAAGCAGCGTGAGCAGCCAGAGCGATGCGAGCA  CCAGCAGCCAGAGCGGCGCGGATAGCAGCAGCGCGAGCGGCCAGCAGCAGGAAAGCAGCGTGCTGAGCCA  GAGCAGCCAGGCGAGCACCAGCAGCCAGCTGGGCGCGGATAGCAGCAGCGCGGGCAACCAGCAGCAGGAA  AGCAGCGTGAGCAGCCAGAGCGATGCGAGCACCAGCAGCCAGCTGGGCGCGGATAGCAGCAGCGCGGGCA  ACCAGCAGCAGGAAAGCAGCGTGAGCAGCCAGAGCGATGCGAGCACCAGCAGCCAGCTGGGCACCAACTG  GCGCCAGGAAATGCGCAGCAAAGTGGCGAGCGTGGAATATATTCTGGCGGCGCGCGCGCTGATTAGCGTG  GGCGTGTATGCGGCGCAGGAAGGCATTGCGCGCAGCCTGGGCCATACCCCGCTGCGCGTGGCGGAAGTGG  AAGCGATTGTGCGCGATAGCCTGGTGCGCAGCCATTTTCATGATAGCGGCCTGAGCCTGGGCAGCATTCG  CCTGGTGCTGATGCAGGTGGGCGATAAACTGGGCCTGCAGGGC | In.1:  1.GATAGCAGCAGCGCGAGCGGCCAGCAGCAGGAAAGCAGCGTGCTGCCG-CAGAGCGATGCGAGCACCAGCAGCCAGCTGGGCGCG  2.GATAGCAGCAGCGCGGGCAACCAGCAGCAGGAAAGCAGCGTGAGCAGCCAGAGCGATGCGAGCACCAGCAGCCAG-AGCGGCGCG  3.GATAGCAGCAGCGCGAGCGGCCAGCAGCAGGAAAGCAGCGTGCTG-AGCCAGAGCAGCCAGGCGAGCACCAGCAGCCAGCTGGGCGCG  4.GATAGCAGCAGCGCGGGCAACCAGCAGCAGGAAAGCAGCGTGAGCAGCCAGAGCGATGCGAGCACCAGCAGCCAGCTGGGCGCG  5.GATAGCAGCAGCGCGGGCAACCAGCAGCAGGAAAGCAGCGTGAGCAGCCAGAGCGATGCGAGCACCAGCAGCCAG-AGCGGCGCG  6.GATAGCAGCAGCGCGAGCGGCCAGCAGCAGGAAAGCAGCGTGCTG-AGCCAGAGCAGCCAGGCGAGCACCAGCAGCCAGCTGGGCGCG  7.GATAGCAGCAGCGCGGGCAACCAGCAGCAGGAAAGCAGCGTGAGCAGCCAGAGCGATGCGAGCACCAGCAGCCAGCTGGGCGCG  8.GATAGCAGCAGCGCGGGCAACCAGCAGCAGGAAAGCAGCGTGAGCAGCCAGAGCGATGCGAGCACCAGCAGCCAGCTGGGC  In.2:  1.GATAGCAGCAGCGCGAGCGGCCAGCAGCAGGAAAGCAGCGTGCTGCCG-CAGAGCGATGCGAGCACCAGCAGCCAGCTGGGCGCGGATAGCAGCAGCGCGGGCAACCAGCAGCAGGAAAGCAGCGTGAGCAGCCAGAGCGATGCGAGCACCAGCAGCCAG-AGCGGCGCG  2.GATAGCAGCAGCGCGAGCGGCCAGCAGCAGGAAAGCAGCGTGCTG-AGCCAGAGCAGCCAGGCGAGCACCAGCAGCCAGCTGGGCGCGGATAGCAGCAGCGCGGGCAACCAGCAGCAGGAAAGCAGCGTGAGCAGCCAGAGCGATGCGAGCACCAGCAGCCAGCTGGGCGCG  3.GATAGCAGCAGCGCGGGCAACCAGCAGCAGGAAAGCAGCGTGAGCAGCCAGAGCGATGCGAGCAC-AGCGGCGCGGATAGCAGCAGCGCGAGCGGCCAGCAGCAGGAAAGCAGCGTGCTG-AGCCAGAGCAGCCAGGCGAGCACCAGCAGCCAGCTGGGCGCG  4.GATAGCAGCAGCGCGGGCAACCAGCAGCAGGAAAGCAGCGTGAGCAGCCAGAGCGATGCGAGCACCAGCAGCCAGCTGGGCGCGGATAGCAGCAGCGCGGGCAACCAGCAGCAGGAAAGCAGCGTGAGCAGCCAGAGCGATGCGAGCACCAGCAGCCAGCTGGGC  In.3:  1.GATAGCAGCAGCGCGAGCGGCCAGCAGCAGGAAAGCAGCGTGCTGCCG-CAGAGCGATGCGAGCACCAGCAGCCAGCTGGGCGCGGATAGCAGCAGCGCGGGCAACCAGCAGCAGGAAAGCAGCGTGAGCAGCCAGAGCGATGCGAGCACCAGCAGCCAGAGCGGCGCGGATAGCAGCAGCGCGAGCGGCCAGCAGCAGGAAAGCAGCGTGCTGAGCCAGAGCAGCCAGGCGAGCACCAGCAGCCAGCTGGGCGCG  2.GATAGCAGCAGCGCGGGCAACCAGCAGCAGGAAAGCAGCGTGAGCAGCCAGAGCGATGCGAGCACCAGCAGCCAGCTGGGCGCGGATAGCAGCAGCGCGGGCAACCAGCAGCAGGAAAGCAGCGTGAGCAGCCAGAGCGATGCGAGCACCAGCAGCCAGAGCGGCGCGGATAGCAGCAGCGCGAGCGGCCAGCAGCAGGAAAGCAGCGTGCTGAGCCAGAGCAGCCAGGCGAGCACCAGCAGCCAGCTGGGCGCG  3.GATAGCAGCAGCGCGGGCAACCAGCAGCAGGAAAGCAGCGTGAGCAGCCAGAGCGATGCGAGCACCAGCAGCCAGCTGGGCGCGGATAGCAGCAGCGCGGGCAACCAGCAGCAGGAAAGCAGCGTGAGCAGCCAGAGCGATGCGAGCACCAGCAGCCAG | In.1:  447.DSSSASGQQQESSVLPQSDASTSSQLGA  448.DSSSAGNQQQESSVSSQSDASTSSQSGA  449.DSSSASGQQQESSVLSQSSQASTSSQLGA  450.DSSSAGNQQQESSVSSQSDASTSSQLGA  451.DSSSAGNQQQESSVSSQSDASTSSQSGA  452.DSSSASGQQQESSVLSQSSQASTSSQLGA  453.DSSSAGNQQQESSVSSQSDASTSSQLGA  454.DSSSAGNQQQESSVSSQSDASTSSQLG  In.2:  455.DSSSASGQQQESSVLPQSDASTSSQLGADSSSAGNQQQESSVSSQSDASTSSQSGA  456.DSSSASGQQQESSVLSQSSQASTSSQLGADSSSAGNQQQESSVSSQSDASTSSQLGA  457.DSSSAGNQQQESSVSSQSDASTAARIAAARAASSRKAAC  458.DSSSAGNQQQESSVSSQSDASTSSQLGADSSSAGNQQQESSVSSQSDASTSSQLG  In.3:  459.DSSSASGQQQESSVLPQSDASTSSQLGADSSSAGNQQQESSVSSQSDASTSSQSGADSSSASGQQQESSVLSQSSQASTSSQLGA  460.DSSSAGNQQQESSVSSQSDASTSSQLGADSSSAGNQQQESSVSSQSDASTSSQSGADSSSASGQQQESSVLSQSSQASTSSQLGA  461.DSSSAGNQQQESSVSSQSDASTSSQLGADSSSAGNQQQESSVSSQSDASTSSQ | Indices 1: 8  Indices 2: 4  Indices 3: 3 |
| 120 | OQ384904.1 | South Africa: Mpumalanga |  | >OQ384904.1 Anaplasma marginale isolate 38 major surface protein 1A (msp1a) gene, partial cds  ATGGCTGCGCGCCGCAGCTGCTGAAATTTAGCATTCTGATTCTGCGCCTGCATGTGCCGTATGTGACCAT  TGGCCTGCCGGTGTGGCGCGCGGGCTGCGTGGTGGTGCTGTTTCCGGATGTGGGCAGCTTTGTGCTGCGC  CTGACCAGCCTGTATGCGGTGCCGCTGGCGGTGGGCCTGTTTGTGTGCGTGTGCGTGATGAGCGAATGCG  TGTTTCTGCAGCCGACCGATAGCAGCAGCGCGAGCGGCCAGCAGCAGGAAAGCAGCGTGCTGCCGCAGAG  CGATGCGAGCACCAGCAGCCAGCTGGGCGCGGATAGCAGCAGCGCGGGCAACCAGCAGCAGGAAAGCAGC  GTGAGCAGCCAGAGCGATGCGAGCACCAGCAGCCAGAGCGGCGCGGATAGCAGCAGCGCGAGCGGCCAGC  AGCAGGAAAGCAGCGTGCTGAGCCAGAGCAGCCAGGCGAGCACCAGCAGCCAGCTGGGCGCGGATAGCAG  CAGCGCGGGCAACCAGCAGCAGGAAAGCAGCGTGAGCAGCCAGAGCGATGCGAGCACCAGCAGCCAGCTG  GGCACCAACTGGCGCCAGGAAATGCGCAGCAAAGTGGCGAGCGTGGAATATATTCTGGCGGCGCGCGCGC  TGATTAGCGTGGGCGTGTATGCGGCGCAGGAAGGCATTGCGCGCAGCCTGGGCCATACCCCGCTGCGCGT  GGCGGAAGTGGAAGCGATTGTGCGCGATAGCCTGGTGCGCAGCCATTTTCATGATAGCGGCCTGAGCCCG  CGCCTGCATACCACCCGCGCGTATGCGGGCTGGGGC | In.1:  1.GATAGCAGCAGCGCGAGCGGCCAGCAGCAGGAAAGCAGCGTGCTGCCG-CAGAGCGATGCGAGCACCAGCAGCCAGCTGGGCGCG  2.GATAGCAGCAGCGCGGGCAACCAGCAGCAGGAAAGCAGCGTGAGCAGCCAGAGCGATGCGAGCACCAGCAGCCAG-AGCGGCGCG  3.GATAGCAGCAGCGCGAGCGGCCAGCAGCAGGAAAGCAGCGTGCTG-AGCCAGAGCAGCCAGGCGAGCACCAGCAGCCAGCTGGGCGCG  4.GATAGCAGCAGCGCGGGCAACCAGCAGCAGGAAAGCAGCGTGAGCAGCCAGAGCGATGCGAGCACCAGCAGCCAGCTGGGC  In.2:  1.GATAGCAGCAGCGCGAGCGGCCAGCAGCAGGAAAGCAGCGTGCTGCCGCAGAGCGATGCGAGCACCAGCAGCCAGCTGGGCGCGGATAGCAGCAGCGCGGGCAACCAGCAGCAGGAAAGCAGCGTGAGCAGCCAGAGCGATGCGAGCACCAGCAGCCAGAGCGGCGCG  2.GATAGCAGCAGCGCGAGCGGCCAGCAGCAGGAAAGCAGCGTGCTGAGCCAG-AGCAGCCAGGCGAGCACCAGCAGCCAGCTGGGCGCGGATAGCAGCAGCGCGGGCAACCAGCAGCAGGAAAGCAGCGTGAGCAGCCAGAGCGATGCGAGCACCAGCAGCCAG | In.1:  462.DSSSASGQQQESSVLPQSDASTSSQLGA  463.DSSSAGNQQQESSVSSQSDASTSSQSGA  464.DSSSASGQQQESSVLSQSSQASTSSQLGA  465.DSSSAGNQQQESSVSSQSDASTSSQLG  In.2:  466.DSSSASGQQQESSVLPQSDASTSSQLGADSSSAGNQQQESSVSSQSDASTSSQSGA  467.DSSSASGQQQESSVLSQSSQASTSSQLGADSSSAGNQQQESSVSSQSDASTSSQ | Indices 1: 4  Indices 2: 2 |
| 121 | OQ384903.1 | South Africa: Mpumalanga |  | >OQ384903.1 Anaplasma marginale isolate 37b major surface protein 1A (msp1a) gene, partial cds  ATGGCTGCGCGCCGCAGCTGCTGAAATTTAGCATTCTGATTCTGCGCCTGCATGTGCCGTATGTGACCAT  TGGCCTGCCGGTGTGGCGCGCGGGCTGCGTGGTGGTGCTGTTTCCGGATGTGGGCAGCTTTGTGCTGCGC  CTGACCAGCCTGTATGCGGTGCCGCTGGCGGTGGGCCTGTTTGTGTGCGTGTGCGTGATGAGCGAATGCG  TGAGCCTGCAGCCGACCGATAGCAGCAGCGCGAGCGGCCAGCAGCAGGAAAGCAGCGTGCTGCCGCAGAG  CGGCCAGGCGAGCACCAGCAGCCAGAGCGGCGCGGATAGCAGCAGCGCGAGCGGCCAGCAGCAGGAAAGC  AGCGTGCTGAGCCAGAGCAGCCAGGCGAGCACCAGCAGCCAGCTGGGCGCGGATAGCAGCAGCGCGGGCA  ACCAGCAGCAGGAAAGCAGCGTGAGCAGCCAGAGCGATGCGAGCACCAGCAGCCAGCTGGGCACCAACTG  GCGCCAGGAAATGCGCAGCAAAGTGGCGAGCGTGGAATATATTCTGGCGGCGCGCGCGCTGATTAGCGTG  GGCGTGTATGCGGCGCAGGAAGGCATTGCGCGCAGCCTGGGCCATACCCCGCTGCGCGTGGCGGAAGTGG  AAGCGATTGTGCGCGATAGCCTGGTGCGCAGCCATTTTCATGATAGCGGCCTGAGCCTGGGCAGCATTCG  CCTGGTGCTGATGCAGGTGGGCGATAAACTGGGCCTGCAGGGC | 1.GATAGCAGCAGCGCGAGCGGCCAGCAGCAGGAAAGCAGCGTGCTGCCG-CAGAGCGGCCAGGCGAGCACCAGCAGCCAG-AGCGGCGCG  2.GATAGCAGCAGCGCGAGCGGCCAGCAGCAGGAAAGCAGCGTGCTG-AGCCAGAGCAGCCAGGCGAGCACCAGCAGCCAGCTGGGCGCG  3.GATAGCAGCAGCGCGGGCAACCAGCAGCAGGAAAGCAGCGTG-AGCAGCCAGAGC-G-ATGCGAGCACCAGCAGCCAGCTGGGC | 468.DSSSASGQQQESSVLPQSGQASTSSQSGA  469.DSSSASGQQQESSVLSQSSQASTSSQLGA  470.DSSSAGNQQQESSVSSQSDASTSSQLG | 3 |
| 122 | OQ384901.1 | South Africa: Mpumalanga |  | >OQ384901.1 Anaplasma marginale isolate 36c major surface protein 1A (msp1a) gene, partial cds  ATGGCTGCGCGCCGCAGCTGCTGAAATTTAGCATTCTGATTCTGCGCCTGCATGTGCCGTATGTGACCAT  TGGCCTGCCGGTGTGGCGCGCGGGCTGCGTGGTGGTGCTGTTTCCGGATGTGGGCAGCTTTGTGCTGCGC  CTGACCAGCCTGTATGCGGTGCCGCTGGCGGTGGGCCTGTTTGTGTGCGTGTGCGTGATGAGCGAATGCG  GCTTTCTGCAGCCGACCGATAGCAGCAGCGCGAGCGGCCAGCAGCAGGAAAGCAGCGTGCTGCCGCAGAG  CGATGCGAGCACCAGCAGCCAGCTGGGCGCGGATAGCAGCAGCGCGGGCAACCAGCAGCAGGAAAGCAGC  GTGAGCAGCCAGAGCGATGCGAGCACCAGCAGCCAGAGCGGCGCGGATAGCAGCAGCGCGAGCGGCCAGC  AGCAGGAAAGCAGCGTGCTGAGCCAGAGCAGCCAGGCGAGCACCAGCAGCCAGCTGGGCGCGGATAGCAG  CAGCGCGGGCAACCAGCAGCAGGAAAGCAGCGTGAGCAGCCAGAGCGATGCGAGCACCAGCAGCCAGCTG  GGCGCGGATAGCAGCAGCGCGGGCAACCAGCAGCAGGAAAGCAGCGTGAGCAGCCAGAGCGATGCGAGCA  CCAGCAGCCAGAGCGGCGCGGATAGCAGCAGCGCGAGCGGCCAGCAGCAGGAAAGCAGCGTGCTGAGCCA  GAGCAGCCAGGCGAGCACCAGCAGCCAGCTGGGCGCGGATAGCAGCAGCGCGGGCAACCAGCAGCAGGAA  AGCAGCGTGAGCAGCCAGAGCGATGCGAGCACCAGCAGCCAGCTGGGCGCGGATAGCAGCAGCGCGGGCA  ACCAGCAGCAGGAAAGCAGCGTGAGCAGCCAGAGCGATGCGAGCACCAGCAGCCAGCTGGGCACCAACTG  GCGCCAGGAAATGCGCAGCAAAGTGGCGAGCGTGGAATATATTCTGGCGGCGCGCGCGCTGATTAGCGTG  GGCGTGTATGCGGCGCAGGAAGGCATTGCGCGCAGCCTGGGCCATACCCCGCTGCGCGTGGCGGAAGTGG  AAGCGATTGTGCGCGATAGCCTGGTGCGCAGCCATTTTCATGAT | In.1:  1.GATAGCAGCAGCGCGAGCGGCCAGCAGCAGGAAAGCAGCGTGCTGCCG-CAGAGCGATGCGAGCACCAGCAGCCAGCTGGGCGCG  2.GATAGCAGCAGCGCGGGCAACCAGCAGCAGGAAAGCAGCGTGAGCAGCCAGAGCGATGCGAGCACCAGCAGCCAG-AGCGGCGCG  3.GATAGCAGCAGCGCGAGCGGCCAGCAGCAGGAAAGCAGCGTGCTG-AGCCAGAGCAGCCAGGCGAGCACCAGCAGCCAGCTGGGCGCG  4.GATAGCAGCAGCGCGGGCAACCAGCAGCAGGAAAGCAGCGTGAGCAGCCAGAGCGATGCGAGCACCAGCAGCCAGCTGGGCGCG  5.GATAGCAGCAGCGCGGGCAACCAGCAGCAGGAAAGCAGCGTGAGCAGCCAGAGCGATGCGAGCACCAGCAGCCAG-AGCGGCGCG  6.GATAGCAGCAGCGCGAGCGGCCAGCAGCAGGAAAGCAGCGTGCTG-AGCCAGAGCAGCCAGGCGAGCACCAGCAGCCAGCTGGGCGCG  7.GATAGCAGCAGCGCGGGCAACCAGCAGCAGGAAAGCAGCGTGAGCAGCCAGAGCGATGCGAGCACCAGCAGCCAGCTGGGCGCG  8.GATAGCAGCAGCGCGGGCAACCAGCAGCAGGAAAGCAGCGTGAGCAGCCAGAGCGATGCGAGCACCAGCAGCCAGCTGGGC  In.2:  1.GATAGCAGCAGCGCGAGCGGCCAGCAGCAGGAAAGCAGCGTGCTGCCG-CAGAGCGATGCGAGCACCAGCAGCCAGCTGGGCGCGGATAGCAGCAGCGCGGGCAACCAGCAGCAGGAAAGCAGCGTGAGCAGCCAGAGCGATGCGAGCACCAGCAGCCAG-AGCGGCGCG  2.GATAGCAGCAGCGCGAGCGGCCAGCAGCAGGAAAGCAGCGTGCTG-AGCCAGAGCAGCCAGGCGAGCACCAGCAGCCAGCTGGGCGCGGATAGCAGCAGCGCGGGCAACCAGCAGCAGGAAAGCAGCGTGAGCAGCCAGAGCGATGCGAGCACCAGCAGCCAGCTGGGCGCG  3.GATAGCAGCAGCGCGGGCAACCAGCAGCAGGAAAGCAGCGTGAGCAGCCAGAGCGATGCGAGCACCAGCAGCCAG-AGCGGCGCGGATAGCAGCAGCGCGAGCGGCCAGCAGCAGGAAAGCAGCGTGCTG-AGCCAGAGCAGCCAGGCGAGCACCAGCAGCCAGCTGGGCGCG  4.GATAGCAGCAGCGCGGGCAACCAGCAGCAGGAAAGCAGCGTGAGCAGCCAGAGCGATGCGAGCACCAGCAGCCAGCTGGGCGCGGATAGCAGCAGCGCGGGCAACCAGCAGCAGGAAAGCAGCGTGAGCAGCCAGAGCGATGCGAGCACCAGCAGCCAGCTGGGC  In.3:  1.GATAGCAGCAGCGCGAGCGGCCAGCAGCAGGAAAGCAGCGTGCTGCCG-CAGAGCGATGCGAGCACCAGCAGCCAGCTGGGCGCGGATAGCAGCAGCGCGGGCAACCAGCAGCAGGAAAGCAGCGTGAGCAGCCAGAGCGATGCGAGCACCAGCAGCCAGAGCGGCGCGGATAGCAGCAGCGCGAGCGGCCAGCAGCAGGAAAGCAGCGTGCTGAGCCAGAGCAGCCAGGCGAGCACCAGCAGCCAGCTGGGCGCG  2.GATAGCAGCAGCGCGGGCAACCAGCAGCAGGAAAGCAGCGTGAGCAGCCAGAGCGATGCGAGCACCAGCAGCCAGCTGGGCGCGGATAGCAGCAGCGCGGGCAACCAGCAGCAGGAAAGCAGCGTGAGCAGCCAGAGCGATGCGAGCACCAGCAGCCAGAGCGGCGCGGATAGCAGCAGCGCGAGCGGCCAGCAGCAGGAAAGCAGCGTGCTGAGCCAGAGCAGCCAGGCGAGCACCAGCAGCCAGCTGGGCGCG  3.GATAGCAGCAGCGCGGGCAACCAGCAGCAGGAAAGCAGCGTGAGCAGCCAGAGCGATGCGAGCACCAGCAGCCAGCTGGGCGCGGATAGCAGCAGCGCGGGCAACCAGCAGCAGGAAAGCAGCGTGAGCAGCCAGAGCGATGCGAGCACCAGCAGCCAG | In.1:  471.DSSSASGQQQESSVLPQSDASTSSQLGA  472.DSSSAGNQQQESSVSSQSDASTSSQSGA  473.DSSSASGQQQESSVLSQSSQASTSSQLGA  474.DSSSAGNQQQESSVSSQSDASTSSQLGA  475.DSSSAGNQQQESSVSSQSDASTSSQSGA  476.DSSSASGQQQESSVLSQSSQASTSSQLGA  477.DSSSAGNQQQESSVSSQSDASTSSQLGA  478.DSSSAGNQQQESSVSSQSDASTSSQLG  In.2:  479.DSSSASGQQQESSVLPQSDASTSSQLGADSSSAGNQQQESSVSSQSDASTSSQSGA  480.DSSSASGQQQESSVLSQSSQASTSSQLGADSSSAGNQQQESSVSSQSDASTSSQLGA  481.DSSSAGNQQQESSVSSQSDASTSSQSGADSSSASGQQQESSVLSQSSQASTSSQLGA  482.DSSSAGNQQQESSVSSQSDASTSSQLGADSSSAGNQQQESSVSSQSDASTSSQLG  In.3:  483.DSSSASGQQQESSVLPQSDASTSSQLGADSSSAGNQQQESSVSSQSDASTSSQSGADSSSASGQQQESSVLSQSSQASTSSQLGA  484.DSSSAGNQQQESSVSSQSDASTSSQLGADSSSAGNQQQESSVSSQSDASTSSQSGADSSSASGQQQESSVLSQSSQASTSSQLGA  485.DSSSAGNQQQESSVSSQSDASTSSQLGADSSSAGNQQQESSVSSQSDASTSSQ | Indices 1: 8  Indices 2: 4  Indices 3: 3 |
| 123 | OQ384898.1 | South Africa: Mpumalanga |  | >OQ384898.1 Anaplasma marginale isolate 35d major surface protein 1A (msp1a) gene, partial cds  ATGGCTGCGCGCCGCAGCTGCTGAAATTTAGCATTCTGATTCTGCGCCTGCATGTGCCGTATGTGACCAT  TGGCCTGCCGGTGTGGCGCGCGGGCTGCGTGGTGGTGCTGTTTCCGGATGTGGGCAGCTTTGTGCTGCGC  CTGACCAGCCTGTATGCGGTGCCGCTGGCGGTGGGCCTGTTTGTGTGCGTGTGCGTGATGAGCGAATGCG  TGTTTCTGCAGCCGACCGATAGCAGCAGCGCGAGCGGCCAGCAGCAGGAAAGCAGCGTGCTGCCGCAGAG  CGATGCGAGCACCAGCAGCCAGCTGGGCGCGGATAGCAGCAGCGCGGGCAACCAGCAGCAGGAAAGCAGC  GTGAGCAGCCAGAGCGATGCGAGCACCAGCAGCCAGAGCGGCGCGGATAGCAGCAGCGCGAGCGGCCAGC  AGCAGGAAAGCAGCGTGCTGAGCCAGAGCAGCCAGGCGAGCACCAGCAGCCAGCTGGGCGCGGATAGCAG  CAGCGCGGGCAACCAGCAGCAGGAAAGCAGCGTGAGCAGCCAGAGCGATGCGAGCACCAGCAGCCAGCTG  GGCGCGGATAGCAGCAGCGCGGGCAACCAGCAGCAGGAAAGCAGCGTGAGCAGCCAGAGCGATGCGAGCA  CCAGCAGCCAGCTGGGCACCAACTGGCGCCAGGAAATGCGCAGCAAAGTGGCGAGCGTGGAATATATTCT  GGCGGCGCGCGCGCATTATCTGTGCCGCGGCCTGTGCTGCAGCGGCCGCGATCGCGAAATTGCGCGCGCG  TATAGCCCGGCGTGCTGCCGCAGCCGCAGCAACCGCGAAGGC | In.1:  1.GATAGCAGCAGCGCGAGCGGCCAGCAGCAGGAAAGCAGCGTGCTGCCG-CAGAGCGATGCGAGCACCAGCAGCCAGCTGGGCGCG  2.GATAGCAGCAGCGCGGGCAACCAGCAGCAGGAAAGCAGCGTGAGCAGCCAGAGCGATGCGAGCACCAGCAGCCAG-AGCGGCGCG  3.GATAGCAGCAGCGCGAGCGGCCAGCAGCAGGAAAGCAGCGTGCTG-AGCCAGAGCAGCCAGGCGAGCACCAGCAGCCAGCTGGGCGCG  4.GATAGCAGCAGCGCGGGCAACCAGCAGCAGGAAAGCAGCGTGAGCAGCCAGAGCGATGCGAGCACCAGCAGCCAGCTGGGCGCG  5.GATAGCAGCAGCGCGGGCAACCAGCAGCAGGAAAGCAGCGTGAGCAGCCAGAGCGATGCGAGCACCAGCAGCCAGCTGGGC  In.2:  1.GATAGCAGCAGCGCGAGCGGCCAGCAGCAGGAAAGCAGCGTGCTG-CCGCAGAGC-G-ATGCGAGCACCAGCAGCCAGCTGGGCGCGGATAGCAGCAGCGCGGGCAACCAGCAGCAGGAAAGCAGCGTGAGCAGCCAGAGCGATGCGAGCACCAGCAGCCAG-AGCGGCGCG  2.GATAGCAGCAGCGCGAGCGGCCAGCAGCAGGAAAGCAGCGTGCTGAGCCAGAGCAGCCAGGCGAGCACCAGCAGCCAGCTGGGCGCGGATAGCAGCAGCGCGGGCAACCAGCAGCAGGAAAGCAGCGTGAGCAGCCAGAGCGATGCGAGCACCAGCAGCCAGCTGGGCGCG  3.GATAGCAGCAGCGCGGGCAACCAGCAGCAGGAAAGCAGCGTG-AGCAGCCAGAGC-G-ATGCGAGCACCAGCAGCCAGCTGGGC | In.1:  486.DSSSASGQQQESSVLPQSDASTSSQLGA  487.DSSSAGNQQQESSVSSQSDASTSSQSGA  488.DSSSASGQQQESSVLSQSSQASTSSQLGA  489.DSSSAGNQQQESSVSSQSDASTSSQLGA  490.DSSSAGNQQQESSVSSQSDASTSSQLG  In.2:  491.DSSSASGQQQESSVLPQSDASTSSQLGADSSSAGNQQQESSVSSQSDASTSSQSGA  492.DSSSASGQQQESSVLSQSSQASTSSQLGADSSSAGNQQQESSVSSQSDASTSSQLGA  493.DSSSAGNQQQESSVSSQSDASTSSQLG | Indices 1: 5  Indices 2: 3 |
| 124 | OQ384894.1 | South Africa: Mpumalanga |  | >OQ384894.1 Anaplasma marginale isolate 34d major surface protein 1A (msp1a) gene, partial cds  ATGGCTGCGCGCCGCAGCTGCTGAAATTTAGCATTCTGATTCTGCGCCTGCATGTGCCGTATGTGACCAT  TGGCCTGCCGGTGTGGCGCGCGGGCTGCGTGGTGGTGCTGTTTCCGGATGTGGGCAGCTTTGTGCTGCGC  CTGACCAGCCTGTATGCGGTGCCGCTGGCGGTGGGCCTGTTTGTGTGCGTGTGCGTGATGAGCGAACTGA  GCCTGCAGCCGACCGATAGCAGCAGCGCGAGCGGCCAGCAGCAGGAAAGCAGCGTGCTGAGCCCGAGCGG  CCAGGCGAGCACCAGCAGCCAGCTGGGCGCGGATAGCAGCAGCGCGAGCGGCCAGCAGCAGGAAAGCAGC  GTGCTGAGCCCGAGCGGCCAGGCGAGCACCAGCAGCCAGAGCGGCACCGATAGCAGCAGCGCGAGCGGCC  AGCAGCAGGAAAGCAGCGTGCTGAGCCAGAGCGATCAGGCGAGCACCAGCAGCCAGCTGGGCACCGATAG  CAGCAGCGCGAGCGGCCAGCAGCAGGAAAGCAGCGTGCTGAGCCAGAGCGATCAGGCGAGCACCAGCAGC  CAGAGCGGCACCGATTGGCGCCAGGAAATGCGCAGCAAAGTGGCGAGCGTGGAATATATGCTGGCGGCGC  GCGCGCTGA | 1.ACCGATAGCAGCAGCGCGAGCGGCCAGCAGCAGGAAAGCAGCGTGCTGAGCCCGAGCGGCCAGGCGAGCACCAGCAGCCAGCTGGGC  2.GCGGATAGCAGCAGCGCGAGCGGCCAGCAGCAGGAAAGCAGCGTGCTGAGCCCGAGCGGCCAGGCGAGCACCAGCAGCCAG-AGCGGC  3.ACCGATAGCAGCAGCGCGAGCGGCCAGCAGCAGGAAAGCAGCGTGCTGAGCCAGAGCGATCAGGCGAGCACCAGCAGCCAGCTGGGC  4.ACCGATAGCAGCAGCGCGAGCGGCCAGCAGCAGGAAAGCAGCGTGCTGAGCCAGAGCGATCAGGCGAGCACCAGCAGCCAG | 494.TDSSSASGQQQESSVLSPSGQASTSSQLG  495.ADSSSASGQQQESSVLSPSGQASTSSQSG  496.TDSSSASGQQQESSVLSQSDQASTSSQLG  497.TDSSSASGQQQESSVLSQSDQASTSSQ | 4 |
| 125 | OQ384890.1 | South Africa: Mpumalanga |  | >OQ384890.1 Anaplasma marginale isolate 33j major surface protein 1A (msp1a) gene, partial cds  ATGGCTGCGCGCCGCAGCTGCTGAAATTTAGCATTCTGATTCTGCGCCTGCATGTGCCGTATGTGACCAT  TGGCCTGCCGGTGTGGCGCGCGGGCTGCGTGGTGGTGCTGTTTCCGGATGTGGGCAGCTTTGTGCTGCGC  CTGACCAGCCTGTATGCGGTGCCGCTGGCGGTGGGCCTGTTTGTGTGCGTGTGCGTGATGAGCGAATATG  TGAGCCTGCAGCCGACCGATAGCAGCAGCGCGAGCGGCCAGCAGCAGGAAAGCAGCGTGAGCAGCCAGAG  CGGCCAGGCGAGCACCAGCAGCCAGAGCGGCGCGGATAGCAGCAGCGCGAGCGGCCAGCAGCAGGAAAGC  AGCGTGAGCAGCCAGAGCGGCCAGGCGAGCACCAGCAGCCAGCTGGGCGCGGATAGCAGCAGCGCGAGCG  GCCAGCAGCAGGAAAGCAGCGTGCTGAGCCAGAGCGATCAGGCGAGCACCAGCAGCCAGCTGGGCACCGA  TTGGCGCCAGGAAATGCGCAGCAAAGTGGCGAGCGTGGAATATATTCTGGCGGCGCGCGCGCTGATTAGC  GTGGGCGTGTATGCGGCGCAGGAAGGCATTGCGCGCAGCCTGGGCCATACCCCGCTGCGCGTGGCGGAAG  TGGAAGCGATTGTGCGCGATAGCCTGGTGCGCAGCCATTTTCATGATAGCGGCCTGAGCCTGGGCAGCAT  TCGCCTGGTGCTGATGCAGGTGGGCGATAAACTGGGCCTGCAGGGC | In.1:  1.AGCAGCCAGAGCGGCCAGGCGAGCACC  2.AGCAGCCAGAGCGGCGC-GGATAGCAGC  3.AGC-G-C-GAGCGGCCAGCAGCAGGAAAGCAGCGTG    4.AGCAGCCAGAGCGGCCAGGCGAGCACC  5.AGCAGCCAG  In.2:  1.GATAGCAGCAGCGCGAGCGGCCAGCAGCAGGAAAGCAGCGTGAGCAGCCAGAGCGGCCAGGCGAGCACCAGCAGCCAG-AGCGGCGCG  2.GATAGCAGCAGCGCGAGCGGCCAGCAGCAGGAAAGCAGCGTGAGCAGCCAGAGCGGCCAGGCGAGCACCAGCAGCCAGCTGGGCGCG  3.GATAGCAGCAGCGCGAGCGGCCAGCAGCAGGAAAGCAGCGTGCTG-AGCCAGAGCGATCAGGCGAGCACCAGCAGCCAGCTGGGC | In.1:  498.SSQSGQAST  499.SSQSGADSS  500.SASGQQQESSV  501.SSQSGQAST  502.SSQ  In.2:  503.DSSSASGQQQESSVSSQSGQASTSSQSGA  504.DSSSASGQQQESSVSSQSGQASTSSQLGA  505.DSSSASGQQQESSVLSQSDQASTSSQLG | Indices 1: 5  Indices 2: 3 |
| 126 | MG570159 | Russia | Moscow | >MG570159.1 Anaplasma marginale isolate RM11 major surface protein 1 alpha (msp1a) gene, partial cds CGCATTACACGTTCCGTATGTTACAATCAGACACGCCGGTGTGGTAGCGTGCTGGTTGTGTGGTTGTCCT CTTTCCCGATGTTGGGTCGTTGTTTTACGTCTCACAAGTTTGTACGCTGTGCCCCTGGCGGTGTAGGGTT TGTTTGTGTGTGTGTGTGTTATGTCAGGGTATGTGTCCAGCCAGCCAGCTGATAGCTTGTCAGCGGGTAA TCAGCAGCAAGAGAGTGATGTGTCATCTCAAAGTGAGGCCAGTACATCGTCTCAATTAGGAGCTGATAGC TTGTCAGCGGGTAATCAGCAGCAAGAGAGTGATGTGTCATCTCAAAGTGAGGCCAGTACATCGTCTCAAT TAGGAGCTGATAGCTCGTCAGCGAGTGGTCAGCAGCAAGAGAGTGGTGTGTCATCTCAAAGTGAGGCCAG TACATCGTCTCAATTAGGAGCTGATAGCTTGTCAGCGGGTAATCAGCAGCAAGAGAGTGATGTGTCATCT CAAAGTGAGGCCAGTACATCGTCTCAATTAGGGACTGATTGGCGGCAAGAGATGCGCTCCAAGGTTGCGA GTGTTGAG | AGCTGATAGCTTGTCAGCGGGTAATCAGCAGCAAGAGAGTGATGTGTCATCTCAAAGTGAGGCCAGTACATCGTCTCAATTAGG AGCTGATAGCTTGTCAGCGGGTAATCAGCAGCAAGAGAGTGATGTGTCATCTCAAAGTGAGGCCAGTACATCGTCTCAATTAGG AGCTGATAGCTCGTCAGCGAGTGGTCAGCAGCAAGAGAGTGGTGTGTCATCTCAAAGTGAGGCCAGTACATCGTCTCAATTAGG AGCTGATAGCTTGTCAGCGGGTAATCAGCAGCAAGAGAGTGATGTGTCATCTCAAAGTGAGGCCAGTACATCGTCTCAATTAGG | 506. ADSLSAGNQQQESDVSSQSEASTSSQL  507. ADSLSAGNQQQESDVSSQSEASTSSQL  508. ADSSSASGQQQESGVSSQSEASTSSQL  509. ADSLSAGNQQQESDVSSQSEASTSSQL | 4 |
| 127 | MG570158 | Russia | Moscow | >MG570158.1 Anaplasma marginale isolate RM10 major surface protein 1 alpha (msp1a) gene, partial cds CGCATTACACGTTCCGTATGTTACAATCAGACACGCCGGTGTGGTAGCGTGCTGGTTGTGTGGTTGTCCT CTTTCCCGATGTTGGGTCGTTGTTTTACGTCTCACAAGTTTGTACGCTGTGCCCCTGGCGGTGTAGGGTT TGTTTGTGTGTGTGTGTGTTATGTCAGGGTATGTGTCCAGCCAGCCAGCTGATAGCTCGTCAGCGAGTGG TCAGCAGCAAGAGAGTGGTGTGTCATCTCAAAGTGAGGCCAGTACATCGTCTCAATTAGGAGCTGATAGC TTGTCAGCGGGTAATCAGCAGCAAGAGAGTGATGTGTCATCTCAAAGTGAGGCCAGTACATCGTCTCAAT TAGGAGCTGATAGCTCGTCAGCGAGTGGTCAGCAGCAAGAGAGTGGTGTGTCATCTCAAAGTGAGGCCAG TACATCGTCTCAATTAGGAGCTGATAGCTTGTCAGCGGGTAATCAGCAGCAAGAGAGTGATGTGTCATCT CAAAGTGAGGCCAGTACATCGTCTCAATTAGGAGCTGATAGCTCGTCAGCGAGTGGTCAGCAGCAAGAGA GTGGTGTGTCATCTCAAAGTGAGGCCAGTACATCGTCTCAATTAGGAGCTGATAGCTTGTCAGCGGGTAA TCAGCAGCAAGAGAGTGATGTGTCATCTCAAAGTGAGGCCAGTACATCGTCTCAATTAGGGACTGATTGG CGGCAAGAGATGCGCTCCAAGGTTGCGAGTGTTGAG | AGCTGATAGCTCGTCAGCGAGTGGTCAGCAGCAAGAGAGTGGTGTGTCATCTCAAAGTGAGGCCAGTACATCGTCTCAATTAGGAGCTGATAGCTTGTCAGCGGGTAATCAGCAGCAAGAGAGTGATGTGTCATCTCAAAGTGAGGCCAGTACATCGTCTCAATTAGG AGCTGATAGCTCGTCAGCGAGTGGTCAGCAGCAAGAGAGTGGTGTGTCATCTCAAAGTGAGGCCAGTACATCGTCTCAATTAGGAGCTGATAGCTTGTCAGCGGGTAATCAGCAGCAAGAGAGTGATGTGTCATCTCAAAGTGAGGCCAGTACATCGTCTCAATTAGG AGCTGATAGCTCGTCAGCGAGTGGTCAGCAGCAAGAGAGTGGTGTGTCATCTCAAAGTGAGGCCAGTACATCGTCTCAATTAGGAGCTGATAGCTTGTCAGCGGGTAATCAGCAGCAAGAGAGTGATGTGTCATCTCAAAGTGAGGCCAGTACATCGTCTCAATTAGG AGCTGATAGCTCGTCAGCGAGTGGTCAGCAGCAAGAGAGTGGTGTGTCATCTCAAAGTGAGGCCA GTACATCGTCTCAATTAGG AGCTGATAGCTTGTCAGCGGGTAATCAGCAGCAAGAGAGTGATGTGTCATCTCAAAGTGAGGCCAGTACATCGTCTCAATTAGG AGCTGATAGCTCGTCAGCGAGTGGTCAGCAGCAAGAGAGTGGTGTGTCATCTCAAAGTGAGGCCAGTACATCGTCTCAATTAGG AGCTGATAGCTTGTCAGCGGGTAATCAGCAGCAAGAGAGTGATGTGTCATCTCAAAGTGAGGCCAGTACATCGTCTCAATTAGG AGCTGATAGCTCGTCAGCGAGTGGTCAGCAGCAAGAGAGTGGTGTGTCATCTCAAAGTGAGGCCAGTACATCGTCTCAATTAGG AGCTGATAGCTTGTCAGCGGGTAATCAGCAGCAAGAGAGTGATGTGTCATCTCAAAGTGAGGCCAGTACATCGTCTCAATTAGG | 510. ADSSSASGQQQESGVSSQSEASTSSQLGADSLSAGNQQQESDVSSQSEASTSSQL    511. ADSSSASGQQQESGVSSQSEASTSSQLGADSLSAGNQQQESDVSSQSEASTSSQL  512. ADSSSASGQQQESGVSSQSEASTSSQLGADSLSAGNQQQESDVSSQSEASTSSQL | 3 |
| 128 | MG570157 | Russia | Moscow | >MG570157.1 Anaplasma marginale isolate RM9 major surface protein 1 alpha (msp1a) gene, partial cds CGCATTACACGTTCCGTATGTTACAATCAGACACGCCGGTGTGGTAGCGTGCTGGTTGTGTGGTTGTCCT CTTTCCCGATGTTGGGTCGTTGTTTTACGTCTCACAAGTTTGTACGCTGTGCCCCTGGCGGTGTAGGGTT TGTTTGTGTGTGTGTGTGTTATGTCAGGGTATGTGTCCAGCCAGCCAGCTGATAGCTCGTCAGCGAGTGG TCAGCAGCAAGAGAGTGGTGTGTCATCTCAAAGTGAGGCCAGTACATCGTCTCAATTAGGAGCTGATAGC TTGTCAGCGGGTAATCAGCAGCAAGAGAGTGATGTGTCATCTCAAAGTGAGGCCAGTACATCGTCTCAAT TAGGGACTGATTGGCGGCAAGAGATGCGCTCCAAGGTTGCGAGTGTTGAG | AGCTGATAGCTCGTCAGCGAGTGGTCAGCAGCAAGAGAGTGGTGTGTCATCTCAAAGTGAGGCCAGTACATCGTCTCAATTAGG AGCTGATAGCTTGTCAGCGGGTAATCAGCAGCAAGAGAGTGATGTGTCATCTCAAAGTGAGGCCAGTACATCGTCTCAATTAGG | 519. ADSSSASGQQQESGVSSQSEASTSSQL  520. ADSLSAGNQQQESDVSSQSEASTSSQL | 2 |
| 129 | MG570156 | Russia | Moscow | >MG570156.1 Anaplasma marginale isolate RM8 major surface protein 1 alpha (msp1a) gene, partial cds CGCATTACACGTTCCGTATGTTACAATCAGGCCGCCGGTGTGGTAGCGTGCTGGTTGTGTGGTTGTCATC TTTCCCGATGTTGGGTCGTTCGTTTTACGTCTCACAAGTTTGTACGCTGTGCCCCTGGCAGTGTAGGGTT TATTTGTTTGTGTGTGTGTTATGTCAGCAGAGTGTGTGTCCTCCCAACCAGCTAATAGCTCGTCAGCGAG TGATCAGCAGCAAGGGAGTGGTGTGTCATCTCAAAGTGAGGCCAGTACATCGTCTCAATTAGGGACTGAT AGCTCGTCAGCGAGTGGTCAGCAGCAAGAGAGTGGTGTGTCATCTCAAAGTGAGGCCAGTACATCGTCTC AATTAGGGACTGATAGCTCGTCAGCGAGTGGTCAGCAGCAAGAGAGTGGTGTGTCATCTCAAAGTGAGGC CAGTACATCGTCTCAATTAGGGACTGATAGCTCGTCAGCGAGTGGTCAGCAGCAAGAGAGTGGTGTGTCA TCTCAAAGTGAGGCCAGTACATCGTCTCAATTAGGGACTGATTGGCGGCAAGAGATGCGCTCCAAGGTTG CGAGTGTTGAG | ATAGCTCGTCAGCGAGTGATCAGCAGCAAGGGAGTGGTGTGTCATCTCAAAGTGAGGCCAGTACATCGTCTCAATTAGGGACTG ATAGCTCGTCAGCGAGTGGTCAGCAGCAAGAGAGTGGTGTGTCATCTCAAAGTGAGGCCAGTACATCGTCTCAATTAGGGACTG ATAGCTCGTCAGCGAGTGGTCAGCAGCAAGAGAGTGGTGTGTCATCTCAAAGTGAGGCCAGTACATCGTCTCAATTAGGGACTG ATAGCTCGTCAGCGAGTGGTCAGCAGCAAGAGAGTGGTGTGTCATCTCAAAGTGAGGCCAGTACATCGTCTCAATTAGGGACTG | 521. SSSASDQQQGSGVSSQSEASTSSQLGT  522. SSSASGQQQESGVSSQSEASTSSQLGT  523. SSSASGQQQESGVSSQSEASTSSQLGT  524. SSSASGQQQESGVSSQSEASTSSQLGT | 4 |
| 130 | MG570155 | Russia | Moscow | >MG570155.1 Anaplasma marginale isolate RM7 major surface protein 1 alpha (msp1a) gene, partial cds CGCATTACACGTTCCGTATGTTACAATCAGGCCGCCGGTGTGGTAGCGTGCTGGTTGTGTGGTTGTCATC TTTCCCGATGTTGGGTCGTTCGTTTTACGTCTCACAAGTTTGTACGCTGTGCCCCTGGCAGTGTAGGGTT TATTTGTTTGTGTGTGTGTTATGTCAGCAGAGTGTGTGTCCTCCCAACCAGCTAATAGCTCGTCAGCGAG TGATCAGCAGCAAGGGAGTGGTGTGTCATCTCAAAGTGAGGCCAGTACATCGTCTCAATTAGGGACTGAT AGCTCGTCAGCGAGTGGTCAGCAGCAAGAGAGTGGTGTGTCATCTCAAAGTGAGGCCAGTACATCGTCTC AATTAGGGACTGATAGCTCGTCAGCGAGTGGTCAGCAGCAAGAGAGTGGTGTGTCATCTCAAAGTGAGGC CAGTACATCGTCTCAATTAGGGACTGATTGGCGGCAAGAGATGCGCTCCAAGGTTGCGAGTGTTGAG | ATAGCTCGTCAGCGAGTGATCAGCAGCAAGGGAGTGGTGTGTCATCTCAAAGTGAGGCCAGTACATCGTCTCAATTAGGGACTG ATAGCTCGTCAGCGAGTGGTCAGCAGCAAGAGAGTGGTGTGTCATCTCAAAGTGAGGCCAGTACATCGTCTCAATTAGGGACTG ATAGCTCGTCAGCGAGTGGTCAGCAGCAAGAGAGTGGTGTGTCATCTCAAAGTGAGGCCAGTACATCGTCTCAATTAGGGACTG | 525. SSSASDQQQGSGVSSQSEASTSSQLGT  526. SSSASGQQQESGVSSQSEASTSSQLGT  527. SSSASGQQQESGVSSQSEASTSSQLGT | 3 |
| 131 | MG570154 | Russia | Moscow | >MG570154.1 Anaplasma marginale isolate RM6 major surface protein 1 alpha (msp1a) gene, partial cds CGCATTACACGTTCCGTATGTTACAATCAGGCCGCCGGTGTGGTAGCGTGCTGGTTGTGTGGTTGTCATC TTTCCCGATGTTGGGTCGTTCGTTTTACGTCTCACAAGTTTGTACGCTGTGCCCCTGGCAGTGTAGGGTT TATTTGTTTGTGTGTGTGTTATGTCAGCAGAGTGTGTGTCCTCCCAACCAGCTAATAGCTCGTCAGCGAG TGATCAGCAGCAAGGGAGTGGTGTGTCATCTCAAAGTGAGGCCAGTACATCGTCTCAATTAGGGACTGAT AGCTCGTCAGCGAGTGGTCAGCAGCAAGAGAGTGGTGTGTCATCTCAAAGTGAGGCCAGTACATCGTCTC AATTAGGGACTGATTGGCGGCAAGAGATGCGCTCCAAGGTTGCGAGTGTTGAG | ATAGCTCGTCAGCGAGTGATCAGCAGCAAGGGAGTGGTGTGTCATCTCAAAGTGAGGCCAGTACATCGTCTCAATTAGGGACTG ATAGCTCGTCAGCGAGTGGTCAGCAGCAAGAGAGTGGTGTGTCATCTCAAAGTGAGGCCAGTACATCGTCTCAATTAGGGACTG | 528. SSSASDQQQGSGVSSQSEASTSSQLGT  529. SSSASGQQQESGVSSQSEASTSSQLGT | 2 |
| 132 | MG570153 | Russia | Moscow | >MG570153.1 Anaplasma marginale isolate RM5 major surface protein 1 alpha (msp1a) gene, partial cds CGCATTACACGTTCCGTATGTTACAATCAGGCCGCCGGTGTGGTAGCGTGCTGGTTGTGTGGTTGTCATC TTTCCCGATGTTGGGTCGTTCGTTTTACGTCTCACAAGTTTGTACGCTGTGCCCCTGGCAGTGTAGGGTT TATTTGTTTGTGTGTGTGTTATGTCAGCAGAGTGTGTGTCCTCCCAACCAGCTAATAGCTCGTCAGCGAG TGGTCAGCAGCAAGAGAGTGGTGTGTCATCTCAAAGTGAGGCCAGTACATCGTCTCAATTAGGGACTGAT AGCTCGTCAGCGAGTGGTCAGCAGCAAGAGAGTGGTGTGTCATCTCAAAGTGAGGCCAGTACATCGTCTC AATTAGGGACTGATAGCTCGTCAGCGAGTGGTCAGCAGCAAGAGAGTGGTGTGTCATCTCAAAGTGAGGC CAGTACATCGTCTCAATTAGGGACTGATTGGCGGCAAGAGATGCGCTCCAAGGTTGCGAGTGTTGAG | ATAGCTCGTCAGCGAGTGGTCAGCAGCAAGAGAGTGGTGTGTCATCTCAAAGTGAGGCCAGTACATCGTCTCAATTAGGGACTG ATAGCTCGTCAGCGAGTGGTCAGCAGCAAGAGAGTGGTGTGTCATCTCAAAGTGAGGCCAGTACATCGTCTCAATTAGGGACTG ATAGCTCGTCAGCGAGTGGTCAGCAGCAAGAGAGTGGTGTGTCATCTCAAAGTGAGGCCAGTACATCGTCTCAATTAGGGACTG | 530. SSSASGQQQESGVSSQSEASTSSQLGT  531. SSSASGQQQESGVSSQSEASTSSQLGT  532. SSSASGQQQESGVSSQSEASTSSQLGT | 3 |
| 133 | MG570152 | Russia | Moscow | >MG570152.1 Anaplasma marginale isolate RM4 major surface protein 1 alpha (msp1a) gene, partial cds CGCATTACACGTTCCGTATGTTACAATCAGGCACGCCGGTGTGGTAGCGTGCTGGTTGTGTGGTTGTCCT CGTTCCCGATGTTGGGTCGTTGTTTTACGTCTCACAAGTTTGTACGCTGTGCCCCTGGCAGTGTAGGGTT TGTGTGTGTGTGTGTGTTATGTTAGAGTATGTGTCCAACCAGCCAGCTGATAGCTCGTCAGCGAGTGATC AGCAGCAAGAGAGTGGTTTGTCATCTCAAAGTGAGGCCAGTACATCGTCTCAATTAGGGACTGATAGCTC GTCAGCGAGTGGTCAGCAGCAAGAGAGTGGTTTGTCATCTCAAAGTGAGGCCAGTACATCGTCTCAATTA GGGACTGATAGCTCGTCAGCGAGTGGTCAGCAGCAAGAGAGTGGTTTGTCATCTCAAAGTGAGGCCAGTA CATCGTCTCAATTAGGGACTGATAGCTCGTCAGCGAGTGGTCAGCAGCAAGAGAGTGGTTTGTCATCTCA AAGTGAGGCCAGTACATCGTCTCAATTAGGGACTGATAGCTCGTCAGCGAGTGGTCAGCAGCAAGAGAGT GGTTTGTCATCTCAAAGTGAGGCCAGTACATCGTCTCAATTAGGGACTGATAGCTCGTCAGCGAGTGGTC AGCAGCAAGAGAGTGGTTTGTCATCTCAAAGTGAGGCCAGTACATCGTCTCAATTAGGAACTGATTGGCG GCAAGAGATGCGCTCCAAGGTTGCGAGTGTTGAG | CTGATAGCTCGTCAGCGAGTGATCAGCAGCAAGAGAGTGGTTTGTCATCTCAAAGTGAGGCCAGTACATCGTCTCAATTAGGGA CTGATAGCTCGTCAGCGAGTGGTCAGCAGCAAGAGAGTGGTTTGTCATCTCAAAGTGAGGCCAGTACATCGTCTCAATTAGGGA CTGATAGCTCGTCAGCGAGTGGTCAGCAGCAAGAGAGTGGTTTGTCATCTCAAAGTGAGGCCAGTACATCGTCTCAATTAGGGA CTGATAGCTCGTCAGCGAGTGGTCAGCAGCAAGAGAGTGGTTTGTCATCTCAAAGTGAGGCCAGTACATCGTCTCAATTAGGGA CTGATAGCTCGTCAGCGAGTGGTCAGCAGCAAGAGAGTGGTTTGTCATCTCAAAGTGAGGCCAGTACATCGTCTCAATTAGGGA CTGATAGCTCGTCAGCGAGTGGTCAGCAGCAAGAGAGTGGTTTGTCATCTCAAAGTGAGGCCAGTACATCGTCTCAATTAGGAA | 533. DSSSASDQQQESGLSSQSEASTSSQLG    534. DSSSASGQQQESGLSSQSEASTSSQLG  535. DSSSASGQQQESGLSSQSEASTSSQLG  536. DSSSASGQQQESGLSSQSEASTSSQLG  537. DSSSASGQQQESGLSSQSEASTSSQLG  538. DSSSASGQQQESGLSSQSEASTSSQLG | 6 |
| 134 | MG570151 | Russia | Moscow | >MG570151.1 Anaplasma marginale isolate RM3 major surface protein 1 alpha (msp1a) gene, partial cds CGCATTACACGTTCCGTATGTTACAATCAGGCACGCCGGTGTGGTAGCGTGCTGGTTGTGTGGTTGTCCT CGTTCCCGATGTTGGGTCGTTGTTTTACGTCTCACAAGTTTGTACGCTGTGCCCCTGGCAGTGTAGGGTT TGTGTGTGTGTGTGTGTTATGTTAGAGTATGTGTCCAACCAGCCAGCTGATAGCTCGTCAGCGAGTGATC AGCAGCAAGAGAGTGGTTTGTCATCTCAAAGTGAGGCCAGTACATCGTCTCAATTAGGGACTGATAGCTC GTCAGCGAGTGGTCAGCAGCAAGAGAGTGGTTTGTCATCTCAAAGTGAGGCCAGTACATCGTCTCAATTA GGAACTGATTGGCGGCAAGAGATGCGCTCCAAGGTTGCGAGTGTTGAG | CTGATAGCTCGTCAGCGAGTGATCAGCAGCAAGAGAGTGGTTTGTCATCTCAAAGTGAGGCCAGTACATCGTCTCAATTAGGGA CTGATAGCTCGTCAGCGAGTGGTCAGCAGCAAGAGAGTGGTTTGTCATCTCAAAGTGAGGCCAGTACATCGTCTCAATTAGGAA | 539. DSSSASDQQQESGLSSQSEASTSSQLG  540. DSSSASGQQQESGLSSQSEASTSSQLG | 2 |
| 135 | MG570150 | Russia | Moscow | >MG570150.1 Anaplasma marginale isolate RM2 major surface protein 1 alpha (msp1a) gene, partial cds CGCATTACACGTTCCGTATGTTACAATCAGGCACGCCGGTGTGGTAGCGTGCTGGTTGTGTGGTTGTCCT CGTTCCCGATGTTGGGTCGTTGTTTTACGTCTCACAAGTTTGTACGCTGTGCCCCTGGCAGTGTAGGGTT TGTGTGTGTGTGTGTGTTATGTTAGAGTATGTGTCCAACCAGCCAGCTGATAGCTCGTCAGCGAGTGGTC AGCAGCAAGAGAGTGGTTTGTCATCTCAAAGTGAGGCCAGTACATCGTCTCAATTAGGGACTGATAGCTC GTCAGCGAGTGGTCAGCAGCAAGAGAGTGGTTTGTCATCTCAAAGTGAGGCCAGTACATCGTCTCAATTA GGAACTGATTGGCGGCAAGAGATGCGCTCCAAGGTTGCGAGTGTTGAG | CTGATAGCTCGTCAGCGAGTGGTCAGCAGCAAGAGAGTGGTTTGTCATCTCAAAGTGAGGCCAGTACATCGTCTCAATTAGGGA CTGATAGCTCGTCAGCGAGTGGTCAGCAGCAAGAGAGTGGTTTGTCATCTCAAAGTGAGGCCAGTACATCGTCTCAATTAGGAA | 541. DSSSASGQQQESGLSSQSEASTSSQLG  542. DSSSASGQQQESGLSSQSEASTSSQLG | 2 |
| 136 | MG570149 | Russia | Moscow | >MG570149.1 Anaplasma marginale isolate RM1 major surface protein 1 alpha (msp1a) gene, partial cds CGCATTACACGTTCCGTATGTTACAATCAGGCACGCCGGTGTGGTAGCGTGCTGGTTGTGTGGTTGTCCT CGTTCCCGATGTTGGGTCGTTGTTTTACGTCTCACAAGTTTGTACGCTGTGCCCCTGGCAGTGTAGGGTT TGTGTGTGTGTGTGTGTTATGTTAGAGTATGTGTCCAACCAGCCAGCTGATAGCTCGTCAGCGAGTGGTC AGCAGCAAGAGAGTGGTTTGTCATCTCAAAGTGAGGCCAGTACATCGTCTCAATTAGGAACTGATTGGCG GCAAGAGATGCGCTCCAAGGTTGCGAGTGTTGAG | N/A | N/A |  |
| 137 | MG570164.1 | Russia | Saratov region | >MG570164.1 Anaplasma marginale isolate RS5 major surface protein 1 alpha (msp1a) gene, partial cds  CGCATTACACGTTCCGTATGTTACAATCGGGCATGCCGGTGTGGTAGCGTGCTGGTTGTGTGGTTGTCCT  CTTTCCCGATGTGGGTCGTTCGTTTTACGTCTCACAAGTTTGTACGCTGTGCCCCTGGCAGTGTAGGGTT  TGTGTGTGTGTGTGTTATGTCAGAGTATGTGTCCCCCCAGCAAGCTGATAGCTCGTCAGCGAGTGGTCAG  CAGCAAGAGAGTGGTGTGTCATCTCAAAGTGAGGCCAGTACATCGTCTCAATTAGGAACTGATAGCTCGT  CAGCGAGTGGTCAGCAGCAAGAGAGTGGTGTGTCATCTCAAAGTGAGGCCAGTACATCGTCTCAATTAGG  AACTGATAGCTCGTCAGCGAGTGGTCAGCAGCAAGAGAGTGGTGTGTCATCTCAAAGTGAGGCCAGTACA  TCGTCTCAATTAGGAACTGATAGCTCGTCAGCGAGTGGTCAGCAGCAAGAGAGTGGTGTGTCATCTCAAA  GTGAGGCCAGTACATCGTCTCAATTAGGAACTGATAGCTCGTCAGCGAGTGGTCAGCAGCAAGAGAGTGG  TGTGTCATCTCAAAGTGAGGCCAGTACATCGTCTCAATTAGGAACTGATAGCTCGTCAGCGAGTGGTCAG  CAGCAAGAGAGTGGTGTGTCATCTCAAAGTGAGGCCAGTACATCGTCTCAATTAGGAACTGATTGGCGGC  AAGAGATGCGCTCCAAGGTTGCGAGTGTTGAG | CTGATAGCTCGTCAGCGAGTGGTCAGCAGCAAGAGAGTGGTGTGTCATCTCAAAGTGAGGCCAGTACATCGTCTCAATTAGGAA  CTGATAGCTCGTCAGCGAGTGGTCAGCAGCAAGAGAGTGGTGTGTCATCTCAAAGTGAGGCCAGTACATCGTCTCAATTAGGAA  CTGATAGCTCGTCAGCGAGTGGTCAGCAGCAAGAGAGTGGTGTGTCATCTCAAAGTGAGGCCAGTACATCGTCTCAATTAGGAA  CTGATAGCTCGTCAGCGAGTGGTCAGCAGCAAGAGAGTGGTGTGTCATCTCAAAGTGAGGCCAGTACATCGTCTCAATTAGGAA  CTGATAGCTCGTCAGCGAGTGGTCAGCAGCAAGAGAGTGGTGTGTCATCTCAAAGTGAGGCCAGTACATCGTCTCAATTAGGAA  CTGAT | 543  DSSSASGQQQESGVSSQSEASTSSQLG  544.  DSSSASGQQQESGVSSQSEASTSSQLG  545.  DSSSASGQQQESGVSSQSEASTSSQLG  546.  DSSSASGQQQESGVSSQSEASTSSQLG  547.  DSSSASGQQQESGVSSQSEASTSSQLG  548.  D | 5 |
| 138 | MG570163.1 | Russia | Saratov region | >MG570163.1 Anaplasma marginale isolate RS4 major surface protein 1 alpha (msp1a) gene, partial cds  CGCATTACACGTTCCGTATGTTACAATCGGGCATGCCGGTGTGGTAGCGTGCTGGTTGTGTGGTTGTCCT  CTTTCCCGATGTGGGTCGTTCGTTTTACGTCTCACAAGTTTGTACGCTGTGCCCCTGGCAGTGTAGGGTT  TGTGTGTGTGTGTGTTATGTCAGAGTATGTGTCCCCCCAGCAAGCTGATAGCTCGTCAGCGAGTGGTCAG  CAGCAAGAGAGTGGTGTGTCATCTCAAAGTGAGGCCAGTACATCGTCTCAATTAGGAACTGATAGCTCGT  CAGCGAGTGGTCAGCAGCAAGAGAGTGGTGTGTCATCTCAAAGTGAGGCCAGTACATCGTCTCAATTAGG  AACTGATAGCTCGTCAGCGAGTGGTCAGCAGCAAGAGAGTGGTGTGTCATCTCAAAGTGAGGCCAGTACA  TCGTCTCAATTAGGAACTGATAGCTCGTCAGCGAGTGGTCAGCAGCAAGAGAGTGGTGTGTCATCTCAAA  GTGAGGCCAGTACATCGTCTCAATTAGGAACTGATAGCTCGTCAGCGAGTGGTCAGCAGCAAGAGAGTGG  TGTGTCATCTCAAAGTGAGGCCAGTACATCGTCTCAATTAGGAACTGATTGGCGGCAAGAGATGCGCTCC  AAGGTTGCGAGTGTTGAG | CTGATAGCTCGTCAGCGAGTGGTCAGCAGCAAGAGAGTGGTGTGTCATCTCAAAGTGAGGCCAGTACATCGTCTCAATTAGGAA  CTGATAGCTCGTCAGCGAGTGGTCAGCAGCAAGAGAGTGGTGTGTCATCTCAAAGTGAGGCCAGTACATCGTCTCAATTAGGAA  CTGATAGCTCGTCAGCGAGTGGTCAGCAGCAAGAGAGTGGTGTGTCATCTCAAAGTGAGGCCAGTACATCGTCTCAATTAGGAA  CTGATAGCTCGTCAGCGAGTGGTCAGCAGCAAGAGAGTGGTGTGTCATCTCAAAGTGAGGCCAGTACATCGTCTCAATTAGGAA  CTGATAGCTCGTCAGCGAGTGGTCAGCAGCAAGAGAGTGGTGTGTCATCTCAAAGTGAGGCCAGT  ACATCGTCTCAATTAGGAA | 549.  DSSSASGQQQESGVSSQSEASTSSQLG  550.  DSSSASGQQQESGVSSQSEASTSSQLG  551.  DSSSASGQQQESGVSSQSEASTSSQLG  552.  DSSSASGQQQESGVSSQSEASTSSQLG  553.  DSSSASGQQQESGVSSQSEASTSSQLG | 5 |
| 139 | MG570162.1 | Russia | Saratov region | >MG570162.1 Anaplasma marginale isolate RS3 major surface protein 1 alpha (msp1a) gene, partial cds  CGCATTACACGTTCCGTATGTTACAATCGGGCATGCCGGTGTGGTAGCGTGCTGGTTGTGTGGTTGTCCT  CTTTCCCGATGTGGGTCGTTCGTTTTACGTCTCACAAGTTTGTACGCTGTGCCCCTGGCAGTGTAGGGTT  TGTGTGTGTGTGTGTGTGTGTTATGTCAGAGTATGTGTCCCCCCAGCAAGCTGATAGCTCGTCAGCGAGT  GGTCAGCAGCAAGAGAGTGGTGTGTCATCTCAAAGTGAGGCCAGTACATCGTCTCAATTAGGAACTGATA  GCTCGTCAGCGAGTGGTCAGCAGCAAGAGAGTGGTGTGTCATCTCAAAGTGAGGCCAGTACATCGTCTCA  ATTAGGAACTGATAGCTCGTCAGCGAGTGGTCAGCAGCAAGAGAGTGGTGTGTCATCTCAAAGTGAGGCC  AGTACATCGTCTCAATTAGGAACTGATAGCTCGTCAGCGAGTGGTCAGCAGCAAGAGAGTGGTGTGTCAT  CTCAAAGTGAGGCCAGTACATCGTCTCAATTAGGAACTGATAGCTCGTCAGCGAGTGGTCAGCAGCAAGA  GAGTGGTGTGTCATCTCAAAGTGAGGCCAGTACATCGTCTCAATTAGGAACTGATTGGCGGCAAGAGATG  CGCTCCAAGGTTGCGAGTGTTGAG | CTGATAGCTCGTCAGCGAGTGGTCAGCAGCAAGAGAGTGGTGTGTCATCTCAAAGTGAGGCCAGTACATCGTCTCAATTAGGAA  CTGATAGCTCGTCAGCGAGTGGTCAGCAGCAAGAGAGTGGTGTGTCATCTCAAAGTGAGGCCAGTACATCGTCTCAATTAGGAA  CTGATAGCTCGTCAGCGAGTGGTCAGCAGCAAGAGAGTGGTGTGTCATCTCAAAGTGAGGCCAGTACATCGTCTCAATTAGGAA  CTGATAGCTCGTCAGCGAGTGGTCAGCAGCAAGAGAGTGGTGTGTCATCTCAAAGTGAGGCCAGTACATCGTCTCAATTAGGAA  CTGATAGCTCGTCAGCGAGTGGTCAGCAGCAAGAGAGTGGTGTGTCATCTCAAAGTGAGGCCAGTACATCGTCTCAATTAGGAA | 554.  DSSSASGQQQESGVSSQSEASTSSQLG  555.  DSSSASGQQQESGVSSQSEASTSSQLG  556.  DSSSASGQQQESGVSSQSEASTSSQLG  557.  DSSSASGQQQESGVSSQSEASTSSQLG  558.  DSSSASGQQQESGVSSQSEASTSSQLG | 5 |
| 140 | MG570161.1 | Russia | Saratov region |  | CTGATAGCTCGTCAGCGAGTGGTCAGCAGCAAGAGAGTGGTGTGTCATCTCAAAGTGAGGCCAGTACATCGTCTCAATTAGGAA  CTGATAGCTCGTCAGCGAGTGGTCAGCAGCAAGAGAGTGGTGTGTCATCTCAAAGTGAGGCCAGTACATCGTCTCAATTAGGAA  CTGATAGCTCGTCAGCGAGTGGTCAGCAGCAAGAGAGTGGTGTGTCATCTCAAAGTGAGGCCAGTACATCGTCTCAATTAGGAA  CTGATAGCTCGTCAGCGAGTGGTCAGCAGCAAGAGAGTGGTGTGTCATCTCAAAGTGAGGCCAGTACATCGTCTCAATTAGGAA  CTGATAGCTCGTCAGCGAGTGGTCAGCAGCAAGAGAGTGGTGTGTCATCTCAAAGTGAGGCCAGTACATCGTCTCAATTAGGAA | 559.  DSSSASGQQQESGVSSQSEASTSSQLG  560.  DSSSASGQQQESGVSSQSEASTSSQLG  561.  DSSSASGQQQESGVSSQSEASTSSQLG  562.  DSSSASGQQQESGVSSQSEASTSSQLG  563.  DSSSASGQQQESGVSSQSEASTSSQLG | 5 |
| 141 | MG570160.1 | Russia | Saratov region | >MG570160.1 Anaplasma marginale isolate RS1 major surface protein 1 alpha (msp1a) gene, partial cds  CGCATTACACGTTCCGTATGTTACAATCGGGCATGCCGGTGTGGTAGCGTGCTGGTTGTGTGGTTGTCCT  CTTTCCCGATGTGGGTCGTTCGTTTTACGTCTCACAAGTTTGTACGCTGTGCCCCTGGCAGTGTAGGGTT  TGTGTGTGTGTGTGTGTGTTATGTCAGAGTATGTGTCCCCCCAGCAAGCTGATAGCTCGTCAGCGAGTGG  TCAGCAGCAAGAGAGTGGTGTGTCATCTCAAAGTGAGGCCAGTACATCGTCTCAATTAGGAACTGATAGC  TCGTCAGCGAGTGGTCAGCAGCAAGAGAGTGGTGTGTCATCTCAAAGTGAGGCCAGTACATCGTCTCAAT  TAGGAACTGATAGCTCGTCAGCGAGTGGTCAGCAGCAAGAGAGTGGTGTGTCATCTCAAAGTGAGGCCAG  TACATCGTCTCAATTAGGAACTGATAGCTCGTCAGCGAGTGGTCAGCAGCAAGAGAGTGGTGTGTCATCT  CAAAGTGAGGCCAGTACATCGTCTCAATTAGGAACTGATTGGCGGCAAGAGATGCGCTCCAAGGTTGCGA  GTGTTGAG | CTGATAGCTCGTCAGCGAGTGGTCAGCAGCAAGAGAGTGGTGTGTCATCTCAAAGTGAGGCCAGTACATCGTCTCAATTAGGAA  CTGATAGCTCGTCAGCGAGTGGTCAGCAGCAAGAGAGTGGTGTGTCATCTCAAAGTGAGGCCAGTACATCGTCTCAATTAGGAA  CTGATAGCTCGTCAGCGAGTGGTCAGCAGCAAGAGAGTGGTGTGTCATCTCAAAGTGAGGCCAGTACATCGTCTCAATTAGGAA  CTGATAGCTCGTCAGCGAGTGGTCAGCAGCAAGAGAGTGGTGTGTCATCTCAAAGTGAGGCCAGTACATCGTCTCAATTAGGAA | 564.  DSSSASGQQQESGVSSQSEASTSSQLG  565.  DSSSASGQQQESGVSSQSEASTSSQLG  566.  DSSSASGQQQESGVSSQSEASTSSQLG  567.  DSSSASGQQQESGVSSQSEASTSSQLG | 4 |
| 142 | ON489302.1 | **Thailand** | **N/A** | >ON489302.1 Anaplasma marginal e clone 2 major surface protein 1 a(msp1a) gene, partial cds  ATATACTGTGCAGTACGGTTGTGCTCCCCAATTGTTAAAATTTAGTATATTAATCTTGCGATTACACGTT  CCGTATGTTACAATCGGGCTGCCGGTGTGGTAGCGTGCTGGTTGTGTGGTTGTCCTCTTTCCCGATGTTG  GGTCGTTCGTTTTACGTCTCACAAGTTTGTACGCTGTGCCCCTGGCAGTGTAGGGTTTGTTTGTGTGTGT  GTGTGTGTTATGTCAGAGTTGTCCCTTCAGCCAACTGATAGCTCGTCAGCGAGTGGTCAGCAGCAAGAGA  GTAGTGTGTTATCTCCAAGTGGTCAGGCCAGTACATCGTCTCAATTAGGAGCTGATAGCTCGTCAGCGAG  TGGTCAGCAGCAAGAGAGTAGTGTGTTATCTCAAAGTGATCAGGCCAGTACATCGTCTCAATTAGGGACT  GATAGCTCGTCAGCGAGTGGTCAGCAGCAAGAGAGTAGTGTGTTATCTCAAAGTGGGGCCAGTACATCGT  CTCAATTAGGGACTAATTGGCGGCAAGAGATGCGCTCCAAGGTTGCGAGTGTTGAGTACATTTTGGCTGC  TCGTGCCCTTATCTCTGTAGGGGTCTATGCTGCTCAGGAAGAGATCGCGAGATCGCTAGGGTATGCTCCC  CTGCGTGTTGCAGAAGTCGAAGAAATCGTGAGGGATGGCCTTGTACGCAGCCACTTTCATGATAGTGGCC  TTTCACTAGGCTCCATACGACTCGTGCTTATGCAGGTTGG | ACTGATAGCTCGTCAGCGAGTGGTCAGCAGCAAGAGAGTAGTGTGTTATCTCCAAGTGGTCAGGCCAGTACATCGTCTCAATTA-GG  AGCTGATAGCTCGTCAGCGAGTGGTCAGCAGCAAGAGAGTAGTGTGTTATCTCAAAGTGATCAGGCCAGTACATCGTCTCAATTAGGG  ACTGATAGCTCGTCAGCGAGTGGTCAGCAGCAAGAGAGTAGTGTGTTATCTCAAAGTG--GGGCCAGTACATCGTCTCAATTAGGG | 568.  TDSSSASGQQQESSVLSPSGQASTSSQL  569.  ADSSSASGQQQESSVLSQSDQASTSSQLG  570.  TDSSSASGQQQESSVLSQSGASTSSQLG | 3 |
| 143 | ON489301.1 | **Thailand** | **N/A** | >ON489301.1 Anaplasma marginal e clone 1 major surface protein 1a  ATATACTGTGCAGTACGGTTGTGCTCCCCAATTGTTAAAATTTAGTATATTAATCTTGCGATTACACGTT  CCGTATGTTACAATCGGGCTGCCGGTGTGGTAGCGTGCTGGTTGTGTGGTTGTCCTCTTTCCCGATGTTG  GGTCGTTCGTTTTACGTCTCACAAGTTTGTACGCTGTGCCCCTGGCAGTGTAGGGTTTGTTTGTGTGTGT  GTGTGTTATGTCAGAGTTGTCCCTTCAGCCAACTGATAGCTCGTCAGCGAGTGGTCAGCAGCAAGAGAGT  AGTGTGTTATCTCCAAGTGGTCAGGCCAGTACATCGTCTCAATTAGGAGCTGATAGCTCGTCAGCGAGTG  GTCAGCAGCAAGAGAGTAGTGTGTTATCTCAAAGTGGTCAGGCCAGTACATCGTCTCAATTAGGAGCTGA  TAGCTCGTCAGCGAGTGGTCAGCAGCAAGAGAGTAGTGTGTTATCTCAAAGTGATCAGGCCAGTACATCG  TCTCAATTAGGGACTGGTAGCTCGTCAGCGAGTGGTCAGCAGCAAGAGAGTAGTGTGTTATCTCAAAGTG  GGGCCAGTACATCGTCTCAATTAGGGACTAATTGGCGGCAAGAGATGCGCTCCAAGGTTGCGAGTGTTGA  GTACATTTTGGCTGCTCGTGCCCTTATCTCTGTAGGGGTCTATGCTGCTCAGGAAGAGATCGCGAGATCG  CTAGGGTATGCTCCCCTGCGTGTTGCAGAAGTCGAAGAAATCGTGAGGGATGGCCTTGTACGCAGCCACT  TTCATGATAGTGGCCTTTCACTAGGCTCCATACGACTCGTGCTTATGCAGGTTGG | CTGATAGCTCGTCAGCGAGTGGTCAGCAGCAAGAGAGTAGTGTGTTATCTCCAAGTGGTCAGGCCAGTACATCGTCTCAATTAGGAG  CTGATAGCTCGTCAGCGAGTGGTCAGCAGCAAGAGAGTAGTGTGTTATCTCAAAGTGGTCAGGCCAGTACATCGTCTCAATTAGGAG  CTGATAGCTCGTCAGCGAGTGGTCAGCAGCAAGAGAGTAGTGTGTTATCTCAAAGTGATCAGGCCAGTACATCGTCTCAATTAGG-G  ACTGGTAGCTCGTCAGCGAGTGGTCAGCAGCAAGAGAGTAGTGTGTTATCTCAAAGTGG---GGCCAGTACATCGTCTCAATTAGG | 571.  DSSSASGQQQESSVLSPSGQASTSSQLG  572.  DSSSASGQQQESSVLSQSGQASTSSQLG  573.  DSSSASGQQQESSVLSQSDQASTSSQLG  574.  TGSSSASGQQQESSVLSQSGASTSSQL | 4 |
| 144 | ON489300.1 | **Thailand** | **N/A** | >ON489300.1 Anaplasma marginal e clone 1 major surface protein 1a  ATATACTGTGCAGTACGGTTGTGCTCCCCAATTGTTAAAATTTAGTATATTAATCTTGCGATTACACGTT  CCGTATGTTACAATCGGGCTGCCGGTGTGGTAGCGTGCTGGTTGTGTGGTTGTCCTCTTTCCCGATGTTG  GGTCGTTCGTTTTACGTCTCACAAGTTTGTACGCTGTGCCCCTGGCAGTGTAGGGTTTGTTTGTGTGTGT  GTGTGTGTTATGTCAGAGTTGTCCCTTCAGTCAACTGATAGCTCGTCAGCGAGTAGTCAGCAGCAAGAGA  GTAGTGTGTTATCTCAAAGTGATCAGGCCAGTACATCGTCTCAATTAGGAGCTGATAGCTCGTCAGCGAG  TGGTCAGCAGCAAGAGAGTAGTGTGTTATCTCCAAGTGGTCAGGCCAGTACATCGTCTCAATTAGGAGCT  GATAGCTCGTCAGCGAGTGGTCAGCAGCAAGAGAGTAGTGTGTCATCTCAAAGTGGTCAGGCCAGTACAT  CGTCTCAATTAGGAACTGATGGCTCGTCAGCGAGTGGTCAGCAGCAAGAGAGTAGTGTGTCATCTCAAAG  TGGTCAGGCCAGTACATCGTCTCAATTAGGGACTGATTGGCGGCAAGAGGTGCACTCCAAGGTTGCGAGT  GTTGAGTACATTTTGGCTGCTCGTGCCCTTATCTCTGTAGGGGTCTATGCTGCTCAGAGAATGATCGCGC  AATCGCAAGGGTGTGCTTCCCTGAGTGTTGCAGAAGTCGAAGAAATCGTGAGGGATAGCCTTGTACGCAG  CCACTTTCATGATAGTGGCCTTTCACTAGGCTCCATACGACTCGTGTTTATGCAGGTTGG | CTGATAGCTCGTCAGCGAGTAGTCAGCAGCAAGAGAGTAGTGTGTTATCTCAAAGTGATCAGGCCAGTACATCGTCTCAATTAGGAG  CTGATAGCTCGTCAGCGAGTGGTCAGCAGCAAGAGAGTAGTGTGTTATCTCCAAGTGGTCAGGCCAGTACATCGTCTCAATTAGGAG  CTGATAGCTCGTCAGCGAGTGGTCAGCAGCAAGAGAGTAGTGTGTCATCTCAAAGTGGTCAGGCCAGTACATCGTCTCAATTAGGAA  CTGATGGCTCGTCAGCGAGTGGTCAGCAGCAAGAGAGTAGTGTGTCATCTCAAAGTGGTCAGGCCAGTACATCGTCTCAATTAGG | 575.  DSSSASSQQQESSVLSQSDQASTSSQLG  576.  DSSSASGQQQESSVLSPSGQASTSSQLG  577.  DSSSASGQQQESSVSSQSGQASTSSQLG  578.  DGSSASGQQQESSVSSQSGQASTSSQL | 4 |
| 145 | ON489299.1 | **Thailand** | **N/A** | >ON489299.1 Anaplasma marginal e clone 2 major surface protein 1 a(msp1a) gene, partial cds  ATATACTGTGCAGTACGGTTGTGCTCCCCAATTGTTAAAATTTAGTATATTAATCTTGCGATTACACGTT  CCGTATGTTACAATCGGGCTGCCGGTGTGGTAGCGTGCTGGTTGTGTGGTTGTCCTCTTTCCCGATGTTG  GGTCGTTCGCTTTACGTCTCACAAGTTTGTACGCTGTGCCCCTGGCAGTGTAGGGTTTGTTTGTGTGTGT  GTGTGTTATGTCAGAGTTGTCCCTTCAGCCAACTGATAGCTCGTCAGCGAGTGGTCAGCAGCAAGAGAGT  AGTGTGTTATCTCAAAGTGATCAGGCCAGTACATCGTCTCAATTAGGGACTGATAGCTCGTCAGCGAGTG  GTCAGCAGCAAGAGAGTAGTGTGTTATCTCAAAGTGATCAGGCCAGTACATCGTCTCAATTAGGGACTGA  TAGCTCGTCAGCGAGTGGTCAGCAGCAAGAGAGTAGTGTGTTATCTCAAAGTGATCAGGCCAGTACATCG  TCTCAATTAGGAACTGATAGCTCGTCAGCGAGTGGTCAGCAGCAAGAGAGTAGTGTGTTATCTCCAAGTG  GTCAGGCCAGTACATCGTCTCAATCAGGAACTGATAGCTCGTCAGCGAGTGGTCAGCAGCAAGAGAGTAG  TGTGTTATCTCAAAGTGATCAGGCCAGTACATCGTCTCAATCAGGAACTGATTGGCGGCAAGAGATGCGC  TCCAAGGTTGCGAGTGTTGAGTACATGTTGGCTGCTCGTGCCCTTATTTCTGTAGGGGTCTATGCTGCTC  AGGGAGAGATCGCGAGATTGCAAGGGTGTGCTTCCCTGAGTGTTGCAGAAGTCGAAGAAATCGTGAGGGA  TAGCCTTGTACGCAGCCACTTTCATTATAGTGGCCTTTCACTAGGCTCCATACGACTCGTGCTTATGCGG  GTTGG | Index1  AACTGATAGCTCGTCAGCGAGTGGTCAGCAGCAAGAGAGTAGTGTGTTATCTCAAAGTGATCAGGCCAGTACATCGTCTCAATTAGG  GACTGATAGCTCGTCAGCGAGTGGTCAGCAGCAAGAGAGTAGTGTGTTATCTCAAAGTGATCAGGCCAGTACATCGTCTCAATTAGG  GACTGATAGCTCGTCAGCGAGTGGTCAGCAGCAAGAGAGTAGTGTGTTATCTCAAAGTGATCAGGCCAGTACATCGTCTCAATTAGG  AACTGATAGCTCGTCAGCGAGTGGTCAGCAGCAAGAGAGTAGTGTGTTATCTCCAAGTGGTCAGGCCAGTACATCGTCTCAATCAGG  AACTGATAGCTCGTCAGCGAGTGGTCAGCAGCAAGAGAGTAGTGTGTTATCTCAAAGTGATCAGGCCAGTACATCGTCTCAATCAGG  Index2  AACTGATAGCTCGTCAGCGAGTGGTCAGCAGCAAGAGAGTAGTGTGTTATCTCAAAGTGATCAGGCCAGTACATCGTCTCAATTAGG  GACTGATAGCTCGTCAGCGAGTGGTCAGCAGCAAGAGAGTAGTGTGTTATCTCAAAGTGATCAGGCCAGTACATCGTCTCAATTAGG  GACTGATAGCTCGTCAGCGAGTGGTCAGCAGCAAGAGAGTAGTGTGTTATCTCAAAGTGATCAGGCCAGTACATCGTCTCAATTAGG  AACTGATAGCTCGTCAGCGAGTGGTCAGCAGCAAGAGAGTAGTGTGTTATCTCCAAGTGGTCAGGCCAGTACATCGTCTCAATCAGG  AACTGATAGCTCGTCAGCGAGTGGTCAGCAGCAAGAGAGTAGTGTGTTATCTCAAAGTGATCAGGCCAGTACATCGTCTCAATCAGG | Index1:  579.  TDSSSASGQQQESSVLSQSDQASTSSQL  580.  TDSSSASGQQQESSVLSQSDQASTSSQL  581.  TDSSSASGQQQESSVLSQSDQASTSSQL  582.  TDSSSASGQQQESSVLSPSGQASTSSQS  583.  TDSSSASGQQQESSVLSQSDQASTSSQS  Index 2:  584.  TDSSSASGQQQESSVLSQSDQASTSSQL  585.  TDSSSASGQQQESSVLSQSDQASTSSQL  586.  TDSSSASGQQQESSVLSQSDQASTSSQL  587.  TDSSSASGQQQESSVLSPSGQASTSSQS  588.  TDSSSASGQQQESSVLSQSDQASTSSQS | Index1:5  Index2:5 |
| 146 | ON489298.1 | **Thailand** | **N/A** | >ON489298.1 Anaplasma marginal e clone 1 major surface protein 1a  ATATACTGTGCAGTACGGTTGTGCTCCCCAATTGTTAAAATTTAGTATATTAATCTTGCGATTACACGTT  CCGTATGTTACAATCAGGCCGCCGGTGTGGTAGCGTGCTGGTTGTGTGGTTGTCCTCTTTCCCGATGTTG  GGTCGTTCGTTTTACGTCTCACAAGTTTGTACGCTGTGCCCCTGGCAGTGTAGGGTTTGTTTGTTTGTGT  GTGTGTTATGTCAGAGTATGTGTCCTCCCAGCAAGCTGGTAGCTCGTCAGCGAGTGGTCAGCAGCAAGAG  AGTAGTGTGTTATCTCAAAGTGATCAGGCCAGTACATCGTCTCAATTAGGGACTGATAGCTCGTCAGCGA  GTGGTCAGCAGCAAGAGAGTAGTGTGTTATCTCAAAGTGATCAGGCCAGTACATCGTCTCAATTAGGAAC  TGATAGCTCGTCAGCGAGTGGTCAGCAGCAAGAGAGTAGTGTGTTATCTCAAAGTGATCAGGCCAGTACA  TCGTCTCAATTAGGAGCTGATAGCTCGTCAGCGAGTGGTCAGCAGCAAGAGAGTAGTGTGTTATCTCCAA  GTGGTCAGGCCAGTACATCGTCTCAATTAGGAGCTGATAGCTCGTCAGCGAGTGGTCAGCAGCAAGAGAG  TAGTGTGTTATCTCAAAGTGATCAGGCCAGTACATCGTCTCAATTAGGGACTAATTGGCGGCAAGAGATG  CGCTCCAAGGTTGCGAGTGTTGAGTACATTTTGGCTGCTCGTGCCCTTATCTCTGTAGGGGTCCATGCTG  CTCAGGAAGAGATCGCGAGATCGCTAGGGTATGCTCCCCTGCGTGTTGCAGAAGTCGAAGAAATCGTGAG  GGATAGCCTTGTACGCAGCCACTTTCATTATAGTGGCCTTTCACTAGGCTCCATACGACTCGTGCTTATG  CAGGTTGG | TAGCTCGTCAGCGAGTGGTCAGCAGCAAGAGAGTAGTGTGTTATCTCAAAGTGATCAGGCCAGTACATCGTCTCAATTAGGGACTGA  TAGCTCGTCAGCGAGTGGTCAGCAGCAAGAGAGTAGTGTGTTATCTCAAAGTGATCAGGCCAGTACATCGTCTCAATTAGGAACTGA  TAGCTCGTCAGCGAGTGGTCAGCAGCAAGAGAGTAGTGTGTTATCTCAAAGTGATCAGGCCAGTACATCGTCTCAATTA-GGAGCTGA  TAGCTCGTCAGCGAGTGGTCAGCAGCAAGAGAGTAGTGTGTTATCTCCAAGTGGTCAGGCCAGTACATCGTCTCAATTA-GGAGCTGA  TAGCTCGTCAGCGAGTGGTCAGCAGCAAGAGAGTAGTGTGTTATCTCAAAGTGATCAGGCCAGTACATCGTCTCAATTAGGGACT | 589.  SSSASGQQQESSVLSQSDQASTSSQLGT  590.  SSSASGQQQESSVLSQSDQASTSSQLGT  591.  SSSASGQQQESSVLSQSDQASTSSQLGA  592.  SSSASGQQQESSVLSPSGQASTSSQLGA  593.  SSSASGQQQESSVLSQSDQASTSSQLGT | 5 |
| 147 | ON489296.1 | **Thailand** | **N/A** | >ON489296.1 Anaplasma marginal e clone 1 major surface protein 1a  ATATACTGTGCAGTACGGTTGTGCTCCCCAATTGTTAAAATTTAGTATATTAATCTTGCGATTACACGTT  CCGTATGTTACAATCGGGCTGCCGGTGTGGTAGCGTGCTGGTTGTGTGGTTGTCCTCTTTCCCGATGTTG  GGTCGTTCGTTTTACGTCTCACAAGTTTGTACGCTGTGCCCCTGGCAGTGTAGGGTTTGTTTGTGTGTGT  GTGTTATGACAGAGTGTGTGTCCCTTGAGCCAACTGATAGCTCGTCAGCGAGTGGTCAGCAGCAAGAGAG  TAGTGTGTTACCTCAAAGTGGTCAGGCCAGTACATCGTCTCAATTAGGAGCTGATAGCTCGTCAGCGAGT  GGTCAGCAGCAAGAGAGTAGTGTGTTATCTCAAAGTGGTCAGGCCAGTACATCGTCTCAATTAGGAGCTG  ATAGCTCGTCAGCGAGTGGTCAGCAGCAAGAGAGTAGTGTGTTATCTCAAAGTGGTCAGGCCAGTACATC  GTCTCAATTAGGAGCTGATAGCTCGTCAGCGAGTGGTCAGCAGCAAGAGAGTAGTGTGTTATCTCAAAGT  GATCAGGCCAGTACATCGTCTCAATTAGGGACTGATTGGCGGCAAGAGATGCGCTCCAAGGTTGCGAGTG  TTGAGTACATTTTGGCTGCTCGTGCCCTTATCTCTGTAGGGGTCTATGCTGCTCAGGAAGAGATCGCGAG  ATCGCTAGGGTATGCTCCCCTGCGTGTTGCAGAAGTCGAAGAAATCGTGAGGGATGGCCTTGTACGCAGC  CACTCTCATGATAGTGGCCTTTCACTAGGCTCCATACGACTCGTGCTTATGCAGGTTGG | CTGATAGCTCGTCAGCGAGTGGTCAGCAGCAAGAGAGTAGTGTGTTACCTCAAAGTGGTCAGGCCAGTACATCGTCTCAATTAGGAG  CTGATAGCTCGTCAGCGAGTGGTCAGCAGCAAGAGAGTAGTGTGTTATCTCAAAGTGGTCAGGCCAGTACATCGTCTCAATTAGGAG  CTGATAGCTCGTCAGCGAGTGGTCAGCAGCAAGAGAGTAGTGTGTTATCTCAAAGTGGTCAGGCCAGTACATCGTCTCAATTAGGAG  CTGATAGCTCGTCAGCGAGTGGTCAGCAGCAAGAGAGTAGTGTGTTATCTCAAAGTGATCAGGCCAGTACATCGTCTCAATTAGG | 594.  DSSSASGQQQESSVLPQSGQASTSSQLG  595.  DSSSASGQQQESSVLSQSGQASTSSQLG  596.  DSSSASGQQQESSVLSQSGQASTSSQLG  597.  DSSSASGQQQESSVLSQSDQASTSSQL | 4 |
| 148 | ON489295.1 | **Thailand** | **N/A** | >ON489295.1 Anaplasma marginal e clone 2 major surface protein 1 a(msp1a) gene, partial cds  ATATACTGTGCAGTACGGTTGTGCTCCCCAATTGTTAAAATTTAGTATATTAATCTTGCGATTACACGTT  CCGTATGTTACAATCGGGCTGCCGGTGTGGTAGCGTGCTGGTTGTGTGGTTGTCCTCTTTCCCGATGTTG  GGTCGTTCGTTTTACGTCTCACAAGTTTGTACGCTGTGCCCCTGGCAGTGTAGGGTTTGTGTGTGTGTGT  GTGTGTTATGTCAGAGTATGCGTCCAGTACATCGTCTCAATTAGGAACTGATAGCTCGTCAGCGAGTGGT  CAGCAGCAAGAGAGCAGTGTGTCATCTCAAAGTGGTCAGGCCAGTACATCGTCTCAGTTAGGGACTGATA  GCTCGTCAGCGAGTGGTCAGCAGCAAGAGAGCAGTGTGTCATCTCAAAGTGGTCAGGCCAGTACATCGTC  TCAGTTAGGGACTGATAGCTCGTCAGCGAGTGGTCAGCAGCAAGAGAGCAGTGTGTCATCTCAAAGTGGT  CAGGCCAGTACATCGTCTCAGTTAGGGACTGATAGCTCGTCAGCGAGTGGTCAGCAGCAAGAGAGCAGTG  TGTCATCTCAAAGTGGTCAGGCCAGTACATCGTCTCAGTTAGGGACTGATAGCTCGTCAGCGAGTGGTCA  GCAGCAAGAGAGCAGTGTGTCATCTCAAAGTGGTCAGGCCAGTACATCGTCTCAGTTAGGGACTGATTGG  CGGCAAGAGATGCGCTCCAAGGTTGCGAGTGTTGAGTACATTTTGGCTGCTCGTGCCCTTATTTCTGTAG  GGGTCTATGCTGCTCAGGGAGAGATCGCGAGATTGCTAGGGTATACTCCCCTGCGTGTTGCAGAAGTCGA  AGCAATCGTGAGGGATAGCCTTGTACGCAGCCACTTTCATGATAGTGGCCTTTCACTAGGCTCCATACGA  CTCGTGCTTATGCAGGTTGG | CCAGTACATCGTCTCAATTAGGAACTGATAGCTCGTCAGCGAGTGGTCAGCAGCAAGAGAGCAGTGTGTCATCTCAAAGTGGTCAGG  CCAGTACATCGTCTCAGTTAGGGACTGATAGCTCGTCAGCGAGTGGTCAGCAGCAAGAGAGCAGTGTGTCATCTCAAAGTGGTCAGG  CCAGTACATCGTCTCAGTTAGGGACTGATAGCTCGTCAGCGAGTGGTCAGCAGCAAGAGAGCAGTGTGTCATCTCAAAGTGGTCAGG  CCAGTACATCGTCTCAGTTAGGGACTGATAGCTCGTCAGCGAGTGGTCAGCAGCAAGAGAGCAGTGTGTCATCTCAAAGTGGTCAGG  CCAGTACATCGTCTCAGTTAGGGACTGATAGCTCGTCAGCGAGTGGTCAGCAGCAAGAGAGCAGTGTGTCATCTCAAAGTGGTCAGG | 598.  STSSQLGTDSSSASGQQQESSVSSQSGQ  599.  STSSQLGTDSSSASGQQQESSVSSQSGQ  600.  STSSQLGTDSSSASGQQQESSVSSQSGQ  601.  STSSQLGTDSSSASGQQQESSVSSQSGQ  602.  STSSQLGTDSSSASGQQQESSVSSQSGQ | 5 |
| 149 | ON489294.1 | **Thailand** | **N/A** | >ON489294.1 Anaplasma marginal e clone 1 major surface protein 1a  ATATACTGTGCAGTACGGTTGTGCTCCCCAATTGTTAAAATTTAGTATATTAATCTTGCGATTACACGTT  CCGTATGTTACAATCGGGCTGCCGGTGTGGTAGCGTGCTGGTTGTGTGGTTGTCCTCTTTCCCGATGTTG  GGTCGTTCGTTTTACGTCTCACAAGTTTGTACGCTGTGCCCCTGGCAGTGTAGGGTTTGTGTGTGTGTGT  GTGTGTTATGTCAGAGTATGCGTCCAGTACATCGTCTCAATTAGGAACTGATAGCTCGTCAGCGAGTGGT  CAGCAGCAAGAGAGCAGTGTGTCATCTCAAAGTGGTCAGGCCAGTACATCGTCTCAGTTAGGGACTGATA  GCTCGTCAGCGAGTGGTCAGCAGCAAGAGAGCAGTGTGTCATCTCAAAGTGGTCAGGCCAGTACATCGTC  TCAGTTAGGGACTGATAGCTCGTCAGCGAGTGGTCAGCAGCAAGAGAGCAGTGTGTCATCTCAAAGTGGT  CAGGCCAGTACATCGTCTCAGTTAGGGACTGATAGCTCGTCAGCGAGTGGTCAGCAGCAAGAGAGCAGTG  TGTCATCTCAAAGTGGTCAGGCCAGTACATCGTCTCAGTTAGGGACTGATAGCTCGTCAGCGAGTGGTCA  GCAGCAAGAGAGCAGTGTGTCATCTCAAAGTGGTCAGGCCAGTACATCGTCTCAGTTAGGGACTGATTGG  CGGCAAGAGATGCGCTCCAAGGTTGCGAGTGTTGAGTACATTTTGGCTGCTCGTGCCCTTATTTCTGTAG  GGGTCTATGCTGCTCAGGGAGAGATCGCGAGATTGCTAGGGTATACTCCCCTGCGTGTTGCAGAAGTCGA  AGCAATCGTGAGGGATAGCCTTGTACGCAGCCACTTTCATGATAGTGGCCTTTCACTAGGCTCCATACGA  CTCGTGCTTATGCAGGTTGG | CCAGTACATCGTCTCAATTAGGAACTGATAGCTCGTCAGCGAGTGGTCAGCAGCAAGAGAGCAGTGTGTCATCTCAAAGTGGTCAGG  CCAGTACATCGTCTCAGTTAGGGACTGATAGCTCGTCAGCGAGTGGTCAGCAGCAAGAGAGCAGTGTGTCATCTCAAAGTGGTCAGG  CCAGTACATCGTCTCAGTTAGGGACTGATAGCTCGTCAGCGAGTGGTCAGCAGCAAGAGAGCAGTGTGTCATCTCAAAGTGGTCAGG  CCAGTACATCGTCTCAGTTAGGGACTGATAGCTCGTCAGCGAGTGGTCAGCAGCAAGAGAGCAGTGTGTCATCTCAAAGTGGTCAGG  CCAGTACATCGTCTCAGTTAGGGACTGATAGCTCGTCAGCGAGTGGTCAGCAGCAAGAGAGCAGTGTGTCATCTCAAAGTGGTCAGG | 603.  STSSQLGTDSSSASGQQQESSVSSQSGQ  604.  STSSQLGTDSSSASGQQQESSVSSQSGQ  605.  STSSQLGTDSSSASGQQQESSVSSQSGQ  606.  STSSQLGTDSSSASGQQQESSVSSQSGQ  607.  STSSQLGTDSSSASGQQQESSVSSQSGQ | 5 |
| 150 | ON489293.1 | **Thailand** | **N/A** | >ON489293.1 Anaplasma marginal e clone 2 major surface protein 1 a(msp1a) gene, partial cds  ATATACTGTGCAGTACGGTTGTGCTCCCCAATTGTTAAAATTTAGTATATTAATCTTGCGATTACACGTT  CCGTATGTTACAATCGGGCTGCCGGTGTGGTAGCGTGCTGGTTGTGTGGTTGTCCTCTTTCCCGATGTTG  GGTCGTTCGTTTTACGTCTCACAAGTTTGTACGCTGTGCCCCTGGCAGTGTAGGGTTTGTGTGTGTGTGT  GTGTGTTATGTCAGAGTATGCGTCCAGTACATCGTCTCAATTAGGAACTGATAGCTCGTCAGCGAGTGGT  CAGCAGCAAGAGAGCAGTGTGTCATCTCAAAGTGGTCAGGCCAGTACATCGTCTCAGTTAGGGACTGATA  GCTCGTCAGCGAGTGGTCAGCAGCAAGAGAGCAGTGTGTCATCTCAAAGTGGTCAGGCCAGTACATCGTC  TCAGTTAGGGACTGATAGCTCGTCAGCGAGTGGTCAGCAGCAAGAGAGCAGTGTGTCATCTCAAAGTGGT  CAGGCCAGTACATCGTCTCAGTTAGGGACTGATAGCTCGTCAGCGAGTGGTCAGCAGCAAGAGAGCAGTG  TGTCATCTCAAAGTGGTCAGGCCAGTACATCGTCTCAGTTAGGGACTGATAGCTCGTCAGCGAGTGGTCA  GCAGCAAGAGAGCAGTGTGTCATCTCAAAGTGGTCAGGCCAGTACATCGTCTCAGTTAGGGACTGATTGG  CGGCAAGAGATGCGCTCCAAGGTTGCGAGTGTTGAGTACATTTTGGCTGCTCGTGCCCTTATTTCTGTAG  GGGTCTATGCTGCTCAGGGAGAGATCGCGAGATTGCTAGGGTATACTCCCCTGCGTGTTGCAGAAGTCGA  AGCAATCGTGAGGGATAGCCTTGTACGCAGCCACTTTCATGATAGTGGCCTTTCACTAGGCTCCATACGA  CTCGTGCTTATGCAGGTTGG | CCAGTACATCGTCTCAATTAGGAACTGATAGCTCGTCAGCGAGTGGTCAGCAGCAAGAGAGCAGTGTGTCATCTCAAAGTGGTCAGG  CCAGTACATCGTCTCAGTTAGGGACTGATAGCTCGTCAGCGAGTGGTCAGCAGCAAGAGAGCAGTGTGTCATCTCAAAGTGGTCAGG  CCAGTACATCGTCTCAGTTAGGGACTGATAGCTCGTCAGCGAGTGGTCAGCAGCAAGAGAGCAGTGTGTCATCTCAAAGTGGTCAGG  CCAGTACATCGTCTCAGTTAGGGACTGATAGCTCGTCAGCGAGTGGTCAGCAGCAAGAGAGCAGTGTGTCATCTCAAAGTGGTCAGG  CCAGTACATCGTCTCAGTTAGGGACTGATAGCTCGTCAGCGAGTGGTCAGCAGCAAGAGAGCAGTGTGTCATCTCAAAGTGGTCAGG | 608.  STSSQLGTDSSSASGQQQESSVSSQSGQ  609.  STSSQLGTDSSSASGQQQESSVSSQSGQ  610.  STSSQLGTDSSSASGQQQESSVSSQSGQ  611.  STSSQLGTDSSSASGQQQESSVSSQSGQ  612.  STSSQLGTDSSSASGQQQESSVSSQSGQ | 5 |
| 151 | ON489292.1 | **Thailand** | **N/A** | >ON489292.1 Anaplasma marginal e clone 1 major surface protein 1a  ATATACTGTGCAGTACGGTTGTGCTCCCCAATTGTTAAAATTTAGTATATTAATCTTGCGATTACACGTT  CCGTATGTTACAATCGGGCTGCCGGTGTGGTAGCGTGCTGGTTGTGTGGTTGTCCTCTTTCCCGATGTTG  GGTCGTTCGTTTTACGTCTCACAAGTTTGTACGCTGTGCCCCTGGCAGTGTAGGGTTTGTGTGTGTGTGT  GTGTGTTATGTCAGAGTATGCGTCCAGTACATCGTCTCAATTAGGAACTGATAGCTCGTCAGCGAGTGGT  CAGCAGCAAGAGAGCAGTGTGTCATCTCAAAGTGGTCAGGCCAGTACATCGTCTCAGTTAGGGACTGATA  GCTCGTCAGCGAGTGGTCAGCAGCAAGAGAGCAGTGTGTCATCTCAAAGTGGTCAGGCCAGTACATCGTC  TCAGTTAGGGACTGATAGCTCGTCAGCGAGTGGTCAGCAGCAAGAGAGCAGTGTGTCATCTCAAAGTGGT  CAGGCCAGTACATCGTCTCAGTTAGGGACTGATAGCTCGTCAGCGAGTGGTCAGCAGCAAGAGAGCAGTG  TGTCATCTCAAAGTGGTCAGGCCAGTACATCGTCTCAGTTAGGGACTGATAGCTCGTCAGCGAGTGGTCA  GCAGCAAGAGAGCAGTGTGTCATCTCAAAGTGGTCAGGCCAGTACATCGTCTCAGTTAGGGACTGATTGG  CGGCAAGAGATGCGCTCCAAGGTTGCGAGTGTTGAGTACATTTTGGCTGCTCGTGCCCTTATTTCTGTAG  GGGTCTATGCTGCTCAGGGAGAGATCGCGAGATTGCTAGGGTATACTCCCCTGCGTGTTGCAGAAGTCGA  AGCAATCGTGAGGGATAGCCTTGTACGCAGCCACTTTCATGATAGTGGCCTTTCACTAGGCTCCATACGA  CTCGTGCTTATGCAGGTTGG | CCAGTACATCGTCTCAATTAGGAACTGATAGCTCGTCAGCGAGTGGTCAGCAGCAAGAGAGCAGTGTGTCATCTCAAAGTGGTCAGG  CCAGTACATCGTCTCAGTTAGGGACTGATAGCTCGTCAGCGAGTGGTCAGCAGCAAGAGAGCAGTGTGTCATCTCAAAGTGGTCAGG  CCAGTACATCGTCTCAGTTAGGGACTGATAGCTCGTCAGCGAGTGGTCAGCAGCAAGAGAGCAGTGTGTCATCTCAAAGTGGTCAGG  CCAGTACATCGTCTCAGTTAGGGACTGATAGCTCGTCAGCGAGTGGTCAGCAGCAAGAGAGCAGTGTGTCATCTCAAAGTGGTCAGG  CCAGTACATCGTCTCAGTTAGGGACTGATAGCTCGTCAGCGAGTGGTCAGCAGCAAGAGAGCAGTGTGTCATCTCAAAGTGGTCAGG | 613.  STSSQLGTDSSSASGQQQESSVSSQSGQ  614.  STSSQLGTDSSSASGQQQESSVSSQSGQ  615.  STSSQLGTDSSSASGQQQESSVSSQSGQ  616.  STSSQLGTDSSSASGQQQESSVSSQSGQ  617.  STSSQLGTDSSSASGQQQESSVSSQSGQ | 5 |
| 152 | ON489291.1 | **Thailand** | **N/A** | >ON489291.1 Anaplasma marginal e clone 2 major surface protein 1 a(msp1a) gene, partial cds  ATATACTGTGCAGTACGGTTGTGCTCCCCAATTGTTAAAATTTAGTATATTAATCTTGCGATTACACGTT  CCGTATGTTACAATCAGGCCGTCGGTGTGGTAGCGTGCTGGTTGTGTGGTTGTCCTCTTTCCCGATGTTG  GGTCGTTCGTTTTACGTCTCACAAGTTTGTACGCTGTGCCCCTGGCAGTGTAGAGTTTGTTTGTGTGTGT  GTGTGTTATGTCAGAGTGTGTGTCCAGCCAGCCAGCTGATGGCTCGTCAGCGAGTGGTCAGCAGCAAGAG  AGTAGTGTGTCATCTCAAAGTGGTCAGGCCAGTACATCGTCTCAATTAGGAACTGATGGCTCGTCAGCGA  GTGGTCAGCAGCAAGAGAGTAGTGTGTTATCTCAAAGTGGTCAGGCCAGTACATCGTCTCAATTAGGAAC  TGATAGCTCGTCAGCGAGTGGTCAGCAGCAAGAGAGTAGTGTGTCATCTCAAAGTGGTCAGGCCAGTACA  TCGTCTCAATTAGGAACTGATAGCTCGTCAGCGAGTGGTCAGCAGCAAGAGAGTAGTGTGTCATCTCAAA  GTGGTCAGGCCAGTACATCGTCTCAATTAGGAACTGATGGCTCGTCAGCGAGTGGTCAGCAGCAAGAGAG  TAGTGTGTTATCTCAAAGTGGTCAGGCCAGTACATCGTCTCAATTAGGAACTGATAGCTCGTCAGCGAGT  GGTCAGCAGCAAGAGAGTAGTGTGTCATCTCAAAGTGGTCAGGTCAGTACATCGTCTCAATTAGGGACTG  ATTGGCGGCAAGAGGTGCACTCCAAGGTTGCGAGTGTTGAGTACATTTTGGCTGCTCGTGCCCTTATCTC  TGTAGGGGTCTATGCTGCTCAGAGAATGATCGCGCAATCGCAAGGGTGTGCTTCCCTGAGTGTTGCAGAA  GTCGAAGAAATCGTGAGGGATAGCCTTGTACGCAGCCACTTTCATTATAGTGGCCTTTCACTAGGCTCCA  TACGACTCGTGCTTATGCAGGTTGG | CTGATGGCTCGTCAGCGAGTGGTCAGCAGCAAGAGAGTAGTGTGTCATCTCAAAGTGGTCAGGCCAGTACATCGTCTCAATTAGGAA  CTGATGGCTCGTCAGCGAGTGGTCAGCAGCAAGAGAGTAGTGTGTTATCTCAAAGTGGTCAGGCCAGTACATCGTCTCAATTAGGAA  CTGATAGCTCGTCAGCGAGTGGTCAGCAGCAAGAGAGTAGTGTGTCATCTCAAAGTGGTCAGGCCAGTACATCGTCTCAATTAGGAA  CTGATAGCTCGTCAGCGAGTGGTCAGCAGCAAGAGAGTAGTGTGTCATCTCAAAGTGGTCAGGCCAGTACATCGTCTCAATTAGGAA  CTGATGGCTCGTCAGCGAGTGGTCAGCAGCAAGAGAGTAGTGTGTTATCTCAAAGTGGTCAGGCCAGTACATCGTCTCAATTAGGAA  CTGATAGCTCGTCAGCGAGTGGTCAGCAGCAAGAGAGTAGTGTGTCATCTCAAAGTGGTCAGGTCAGTACATCGTCTCAATTAGGGA | 618.  DGSSASGQQQESSVSSQSGQASTSSQLG  619.  DGSSASGQQQESSVLSQSGQASTSSQLG  620.  DSSSASGQQQESSVSSQSGQASTSSQLG  621.  DSSSASGQQQESSVSSQSGQASTSSQLG  622.  DSSSASGQQQESSVSSQSGQASTSSQLG  623.  DSSSASGQQQESSVSSQSGQVSTSSQLG | 6 |
| 153 | ON489290.1 | **Thailand** | **N/A** | >ON489290.1 Anaplasma marginal e clone 1 major surface protein 1a  ATATACTGTGCAGTACGGTTGTGCTCCCCAATTGTTAAAATTTAGTATATTAATCTTGCGATTACACGTT  CCGTATGTTACAATCAGGCCGTCGGTGTGGTAGCGTGCTGGTTGTGTGGTTGTCCTCTTTCCCGATGTTG  GGTCGTTCGTTTTACGTCTCACAAGTTTGTACGCTGTGCCCCTGGCAGTGTAGAGTTTGTTTGTGTGTGT  GTGTGTTATGTCAGAGTGTGTGTCCAGCCAGCCAGCTGATGGCTCGTCAGCGAGTGGTCAGCAGCAAGAG  AGTAGTGTGTCATCTCAAAGTGGTCAGGCCAGTACATCGTCTCAATTAGGAACTGATAGCTCGTCAGCGA  GTGGTCAGCAGCAAGAGAGTAGTGTGTCATCTCAAAGTGGTCAGGCCAGTACATCGTCTCAATTAGGGAC  TGATTGGCGGCAAGAGGTGCACTCCAAGGTTGCGAGTGTTGAGTACATTTTGGCTGCTCGTGCCCTTATC  TCTGTAGGGGTCTATGCTGCTCAGAGAATGATCGCGCAATCGCAAGGGTGTGCTTCCCTGAGTGTTGCAG  AAGTCGAAGAAATCGTGAGGGATAGCCTTGTACGCAGCCACTTTCATTATAGTGGCCTTTCACTAGGCTC  CATACGACTCGTGCTTATGCAGGTTGG | CTGATGGCTCGTCAGCGAGTGGTCAGCAGCAAGAGAGTAGTGTGTCATCTCAAAGTGGTCAGGCCAGTACATCGTCTCAATTAGGAA  CTGATAGCTCGTCAGCGAGTGGTCAGCAGCAAGAGAGTAGTGTGTCATCTCAAAGTGGTCAGGCCAGTACATCGTCTCAATTAGGGA | 624.  DGSSASGQQQESSVSSQSGQASTSSQLG  625.  DSSSASGQQQESSVSSQSGQASTSSQLG | 2 |
| 154 | ON489289.1 | **Thailand** | **N/A** | >ON489289.1 Anaplasma marginal e clone 2 major surface protein 1 a(msp1a) gene, partial cds  ATATACTGTGCAGTACGGTTGTGCTCCCCAATTGTTAAAATTTAGTATATTAATCTTGCGATTACACGTT  CCGTATGTTACAATCAGGCCGTCGGTGTGGTAGCGTGCTGGTTGTGTGGTTGTCCTCTTTCCCGATGTTG  GGTCGTTCGTTTTACGTCTCACAAGTTTGTACGCTGTGCCCCTGGCAGTGTAGAGTTTGTTTGTGTGTGT  GTGTGTTATGTCAGAGTGTGTGTCCAGCCAGCCAGCTGATGGCTCGTCAGCGAGTGGTCAGCAGCAAGAG  AGTAGTGTGTCATCTCAAAGTGGTCAGGCCAGTACATCGTCTCAATTAGGAACTGATGGCTCGTCAGCGA  GTGGTCAGCAGCAAGAGAGTAGTGTGTCATCTCAAAGTGGTCAGGCCAGTACATCGTCTCAATTAGGAAC  TGATAGCTCGTCAGCGAGTGGTCAGCAGCAAGAGAGTAGTGTGTCATCTCAAAGTGGTCAGGCCAGTACA  TCGTCTCAATTAGGGACTGATTGGCGGCAAGAGGTGCACTCCAAGGTTGCGAGTGTTGAGTACATTTTGG  CTGCTCGTGCCCTTATCTCTGTAGGGGTCTATGCTGCTCAGAGAATGATCGCGCAATCGCAAGGGTGTGC  TTCCCTGAGTGTTGCAGAAGTCGAAGAAATCGTGAGGGATAGCCTTGTACGCAGCCACTTTCATTATGGT  GGCCTTTCACTAGGCTCCATACGACTCGTGCTTATGCAGGTTGG | CTGATGGCTCGTCAGCGAGTGGTCAGCAGCAAGAGAGTAGTGTGTCATCTCAAAGTGGTCAGGCCAGTACATCGTCTCAATTAGGAA  CTGATGGCTCGTCAGCGAGTGGTCAGCAGCAAGAGAGTAGTGTGTCATCTCAAAGTGGTCAGGCCAGTACATCGTCTCAATTAGGAA  CTGATAGCTCGTCAGCGAGTGGTCAGCAGCAAGAGAGTAGTGTGTCATCTCAAAGTGGTCAGGCCAGTACATCGTCTCAATTAGGGA | 626.  DGSSASGQQQESSVSSQSGQASTSSQLG  627.  DGSSASGQQQESSVSSQSGQASTSSQLG  628.  DSSSASGQQQESSVSSQSGQASTSSQLG | 3 |
| 155 | ON489288.1 | **Thailand** | **N/A** | >ON489288.1 Anaplasma marginal e clone 1 major surface protein 1a  ATATACTGTGCAGTACGGTTGTGCTCCCCAATTGTTAAAATTTAGTATATTAATCTTGCGATTACACGTT  CCGTATGTTACAATCAGGCCGTCGGTGTGGTAGCGTGCTGGTTGTGTGGTTGTCCTCTTTCCCGATGTTG  GGTCGTTCGTTTTACGTCTCACAAGTTTGTACGCTGTGCCCCTGGCAGTGTAGAGTTTGTTTGTGTGTGT  GTGTGTTATGTCAGAGTGTGTGTCCAGCCAGCCAGCTGATGGCTCGTCAGCGAGTGGTCAGCAGCAAGAG  AGTAGTGTGTCATCTCAAAGTGGTCAGGCCAGTACATCGTCTCAATTAGGAACTGATGGCTCGTCAGCGA  GTGGTCAGCAGCAAGAGAGTAGTGTGTCATCTCAAAGTGGTCAGGCCAGTACATCGTCTCAATTAGGAAC  TGATGGCTCGTCAGCGAGTGGTCAGCAGCAAGAGAGTAGTGTGTCATCTCAAAGTGGTCAGGCCAGTACA  TCGTCTCAATTAGGAACTGATAGCTCGTCAGCGAGTGGTCAGCAGCAAGAGAGTAGTGTGTCATCTCAAA  GTGGTCAGGCCAGTACATCGTCTCAATTAGGGACTGATTGGCGGCAAGAGGTGCACTCCAAGGTTGCGAG  TGTTGAGTACATTTTGGCTGCTCGTGCCCTTATCTCTGTAGGGGTCTATGCTGCTCAGAGAATGATCGCG  CAATCGCAAGGGTGTGCTTCCCTGAGTGTTGCAGAAGTCGAAGAAATCGTGAGGGATAGCCTTGTACGCA  GCCACTTTCATTATGGTGGCCTTTCACTAGGCTCCATACGACTCGTGCTTATGCAGGTTGG  >ON489287.1 Anaplasma marginal e clone 2 major surface protein 1 a(msp1a) gene, partial cds  ATATACTGTGCAGTACGGTTGTGCTCCCCAATTGTTAAAATTTAGTATATTAATCTTGCGATTACACGTT  CCGTATGTTACAATCAGGCCGTCGGTGTGGTAGCGTGCTGGTTGTGTGGTTGTCCTCTTTCCCGATGTTG  GGTCGTTCGTTTTACGTCTCACAAGTTTGTACGCTGTGCCCCTGGCAGTGTAGAGTTTGTTTGTGTGTGT  GTGTGTTATGTCAGAGTGTGTGTCCAGCCAGCCAGCTGATGGCTCGTCAGCGAGTGGTCAGCAGCAAGAG  AGTAGTGTGTCATCTCAAAGTGGTCAGGCCAGTACATCGTCTCAATTAGGAACTGATGGCTCGTCAGCGA  GTGGTCAGCAGCAAGAGAGTAGTGTGTCATCTCAAAGTGGTCAGGCCAGTACATCGTCTCAATTAGGAAC  TGATGGCTCGTCAGCGAGTGGTCAGCAGCAAGAGAGTAGTGTGTTATCTCAAAGTGGTCAGGCCAGTACA  TCGTCTCAATTAGGAACTGATAGCTCGTCAGCGAGTGGTCAGCAGCAAGAGAGTAGTGTGTCATCTCAAA  GTGGTCAGGCCAGTACATCGTCTCAATTAGGAACTGATAGCTCGTCAGCGAGTGGTCAGCAGCAAGAGAG  TAGTGTGTCATCTCAAAGTGGTCAGGCCAGTACATCGTCTCAATTAGGGACTGATTGGCGGCAAGAGGTG  CACTCCAAGGTTGCGAGTGTTGAGTACATTTTGGCTGCTCGTGCCCTTATCTCTGTAGGGGTCTATGCTG  CTCAGAGAATGATCGCGCAATCGCAAGGGTGTGCTTCCCTGAGTGTTGCAGAAGTCGAAGAAATCGTGAG  GGATAGCCTTGTACGCAGCCACTTTCATTATAGTGGCCTTTCACTAGGCTCCATACGACTCGTGCTTATG  CAGGTTGG | INDEX1:  CTGATGGCTCGTCAGCGAGTGGTCAGCAGCAAGAGAGTAGTGTGTCATCTCAAAGTGGTCAGGCCAGTACATCGTCTCAATTAGGAA  CTGATGGCTCGTCAGCGAGTGGTCAGCAGCAAGAGAGTAGTGTGTCATCTCAAAGTGGTCAGGCCAGTACATCGTCTCAATTAGGAA  CTGATGGCTCGTCAGCGAGTGGTCAGCAGCAAGAGAGTAGTGTGTCATCTCAAAGTGGTCAGGCCAGTACATCGTCTCAATTAGGAA  CTGATAGCTCGTCAGCGAGTGGTCAGCAGCAAGAGAGTAGTGTGTCATCTCAAAGTGGTCAGGCCAGTACATCGTCTCAATTAGGGA  INDEX2:  CTGATGGCTCGTCAGCGAGTGGTCAGCAGCAAGAGAGTAGTGTGTCATCTCAAAGTGGTCAGGCCAGTACATCGTCTCAATTAGGAA  CTGATGGCTCGTCAGCGAGTGGTCAGCAGCAAGAGAGTAGTGTGTCATCTCAAAGTGGTCAGGCCAGTACATCGTCTCAATTAGGAA  CTGATGGCTCGTCAGCGAGTGGTCAGCAGCAAGAGAGTAGTGTGTTATCTCAAAGTGGTCAGGCCAGTACATCGTCTCAATTAGGAA  CTGATAGCTCGTCAGCGAGTGGTCAGCAGCAAGAGAGTAGTGTGTCATCTCAAAGTGGTCAGGCCAGTACATCGTCTCAATTAGGAA  CTGATAGCTCGTCAGCGAGTGGTCAGCAGCAAGAGAGTAGTGTGTCATCTCAAAGTGGTCAGGCCAGTACATCGTCTCAATTAGGGA | Index:1  629.  DGSSASGQQQESSVSSQSGQASTSSQLG  630.  DGSSASGQQQESSVSSQSGQASTSSQLG  631.  DGSSASGQQQESSVSSQSGQASTSSQLG  632.  DSSSASGQQQESSVSSQSGQASTSSQLG  INDEX2:  633.  DGSSASGQQQESSVSSQSGQASTSSQLG  634.  DGSSASGQQQESSVSSQSGQASTSSQLG  635.  DGSSASGQQQESSVLSQSGQASTSSQLG  636.  DSSSASGQQQESSVSSQSGQASTSSQLG  637.  DSSSASGQQQESSVSSQSGQASTSSQLG | INDEX1:4  INDEX2:5 |
| 156 | ON489287.1 | **Thailand** | **N/A** | >ON489287.1 Anaplasma marginal e clone 2 major surface protein 1 a(msp1a) gene, partial cds  ATATACTGTGCAGTACGGTTGTGCTCCCCAATTGTTAAAATTTAGTATATTAATCTTGCGATTACACGTT  CCGTATGTTACAATCAGGCCGTCGGTGTGGTAGCGTGCTGGTTGTGTGGTTGTCCTCTTTCCCGATGTTG  GGTCGTTCGTTTTACGTCTCACAAGTTTGTACGCTGTGCCCCTGGCAGTGTAGAGTTTGTTTGTGTGTGT  GTGTGTTATGTCAGAGTGTGTGTCCAGCCAGCCAGCTGATGGCTCGTCAGCGAGTGGTCAGCAGCAAGAG  AGTAGTGTGTCATCTCAAAGTGGTCAGGCCAGTACATCGTCTCAATTAGGAACTGATGGCTCGTCAGCGA  GTGGTCAGCAGCAAGAGAGTAGTGTGTCATCTCAAAGTGGTCAGGCCAGTACATCGTCTCAATTAGGAAC  TGATGGCTCGTCAGCGAGTGGTCAGCAGCAAGAGAGTAGTGTGTTATCTCAAAGTGGTCAGGCCAGTACA  TCGTCTCAATTAGGAACTGATAGCTCGTCAGCGAGTGGTCAGCAGCAAGAGAGTAGTGTGTCATCTCAAA  GTGGTCAGGCCAGTACATCGTCTCAATTAGGAACTGATAGCTCGTCAGCGAGTGGTCAGCAGCAAGAGAG  TAGTGTGTCATCTCAAAGTGGTCAGGCCAGTACATCGTCTCAATTAGGGACTGATTGGCGGCAAGAGGTG  CACTCCAAGGTTGCGAGTGTTGAGTACATTTTGGCTGCTCGTGCCCTTATCTCTGTAGGGGTCTATGCTG  CTCAGAGAATGATCGCGCAATCGCAAGGGTGTGCTTCCCTGAGTGTTGCAGAAGTCGAAGAAATCGTGAG  GGATAGCCTTGTACGCAGCCACTTTCATTATAGTGGCCTTTCACTAGGCTCCATACGACTCGTGCTTATG  CAGGTTGG | CTGATGGCTCGTCAGCGAGTGGTCAGCAGCAAGAGAGTAGTGTGTCATCTCAAAGTGGTCAGGCCAGTACATCGTCTCAATTAGGAA  CTGATGGCTCGTCAGCGAGTGGTCAGCAGCAAGAGAGTAGTGTGTCATCTCAAAGTGGTCAGGCCAGTACATCGTCTCAATTAGGAA  CTGATGGCTCGTCAGCGAGTGGTCAGCAGCAAGAGAGTAGTGTGTTATCTCAAAGTGGTCAGGCCAGTACATCGTCTCAATTAGGAA  CTGATAGCTCGTCAGCGAGTGGTCAGCAGCAAGAGAGTAGTGTGTCATCTCAAAGTGGTCAGGCCAGTACATCGTCTCAATTAGGAA  CTGATAGCTCGTCAGCGAGTGGTCAGCAGCAAGAGAGTAGTGTGTCATCTCAAAGTGGTCAGGCCAGTACATCGTCTCAATTAGGGA | 638.  DGSSASGQQQESSVSSQSGQASTSSQLG  639.  DGSSASGQQQESSVSSQSGQASTSSQLG  640.  DGSSASGQQQESSVLSQSGQASTSSQLG  641.  DSSSASGQQQESSVSSQSGQASTSSQLG  642.  DSSSASGQQQESSVSSQSGQASTSSQLG | 5 |
| 157 | ON489286.1 | **Thailand** | **N/A** | >ON489286.1 Anaplasma marginal e clone 1 major surface protein 1a  ATATACTGTGCAGTACGGTTGTGCTCCCCAATTGTTAAAATTTAGTATATTAATCTTGCGATTACACGTT  CCGTATGTTACAATCAGGCCGTCGGTGTGGTAGCGTGCTGGTTGTGTGGTTGTCCTCTTTCCCGATGTTG  GGTCGTTCGTTTTACGTCTCACAAGTTTGTACGCTGTGCCCCTGGCAGTGTAGAGTTTGTTTGTGTGTGT  GTGTGTTATGTCAGAGTGTGTGTCCAGCCAGCCAGCTGATGGCTCGTCAGCGAGTGGTCAGCAGCAAGAG  AGTAGTGTGTCATCTCAAAGTGGTCAGGCCAGTACATCGTCTCAATTAGGAACTGATAGCTCGTCAGCGA  GTGGTCAGCAGCAAGAGAGTAGTGTGTCATCTCAAAGTGGTCAGGCCAGTACATCGTCTCAATTAGGAAC  TGATAGCTCGTCAGCGAGTGGTCAGCAGCAAGAGAGTAGTGTGTCATCTCAAAGTGGTCAGGCCAGTACA  TCGTCTCAATTAGGGACTGATTGGCGGCAAGAGGTGCACTCCAAGGTTGCGAGTGTTGAGTACATTTTGG  CTGCTCGTGCCCTTATCTCTGTAGGGGTCTATGCTGCTCAGAGAATGATCGCGCAATCGCAAGGGTGTGC  TTCCCTGAGTGTTGCAGAAGTCGAAGAAATCGTGAGGGATAGCCTTGTACGCAGCCACTTTCATTATAGT  GGCCTTTCACTAGGCTCCATACGACTCGTGCTTATGCAGGTTGG | CTGATGGCTCGTCAGCGAGTGGTCAGCAGCAAGAGAGTAGTGTGTCATCTCAAAGTGGTCAGGCCAGTACATCGTCTCAATTAGGAA  CTGATAGCTCGTCAGCGAGTGGTCAGCAGCAAGAGAGTAGTGTGTCATCTCAAAGTGGTCAGGCCAGTACATCGTCTCAATTAGGAA  CTGATAGCTCGTCAGCGAGTGGTCAGCAGCAAGAGAGTAGTGTGTCATCTCAAAGTGGTCAGGCCAGTACATCGTCTCAATTAGGGA | 643.  DGSSASGQQQESSVSSQSGQASTSSQLG  644.  DSSSASGQQQESSVSSQSGQASTSSQLG  645.  DSSSASGQQQESSVSSQSGQASTSSQLG | 3 |
