# Supplementary material for: Nomenclature, classification, and in-silico structural analysis of MSP1a variants of Anaplasma marginale
Source: PLoS One. 2026 Oct 21;21(10):e0360505. doi: 10.1371/journal.pone.0360505 (PMC13686480; doi:10.1371/journal.pone.0360505)
Supplement: Supplementary file 3 [file pone.0360505.s003.docx]

| **Serial No.** | **Strain Name** | **Accession ID** | **Tandem repeat structure** | **No. of Tandems** |
| --- | --- | --- | --- | --- |
| 1. | Brazil/Maranhao/-(A,B,C) | OL629255.1 | A \| B \| C | 3 |
| 2. | Brazil/Maranhao/-(A,S,C,V) | OL629249.1 | A \| S \| C \| V | 4 |
| 3 | Brazil/Maranhao/-(A,S²,T,U) | OL629250.1 | A \| S \| S \| T \| U | 5 |
| 4 | Brazil/Maranhao/-(E,F,G,H) | OL629254.1 | E \| F \| G \| H | 4 |
| 5 | Brazil/Maranhao/-(J,I,J²,K) | OL629253.1 | J \| I \| J \| J \| K | 5 |
| 6 | Brazil/Maranhao/-(J,α,β,Γ,π,Σ) | OL629247.1 | J \| α \| β \| Γ \| π \| Σ | 6 |
| 7 | Brazil/Maranhao/-(L,M,N,O) | OL629252.1 | L \| M \| N \| O | 4 |
| 8 | Brazil/Maranhao/-(Q,P,Q,W) | OL629248.1 | Q \| P \| Q \| W | 4 |
| 9 | Brazil/Maranhao/-(Q,P,Q²,R) | OL629251.1 | Q \| P \| Q \| Q \| R | 5 |
| 10 | Colombia/-(10,11,12) | MF771044.1 | 10 \| 11 \| 12 | 3 |
| 11 | Colombia/-(13,14) | MF771043.1 | 13 \| 14 | 2 |
| 12 | Colombia/-(15,16,17) | MF771042.1 | 15 \| 16 \| 17 | 3 |
| 13 | Colombia/-(18,19,20) | MF771041.1 | 18 \| 19 \| 20 | 3 |
| 14 | Colombia/-(18,38,39) | MF771034.1 | 18 \| 38 \| 39 | 3 |
| 15 | Colombia/-(${21}^{2}$,R) | MF771040.1 | 21 \| 21 \| R | 3 |
| 16 | Colombia/-(22,23,24,25) | MF771039.1 | 22 \| 23 \| 24 \| 25 | 4 |
| 17 | Colombia/-(26,27,28) | MF771038.1 | 26 \| 27 \| 28 | 3 |
| 18 | Colombia/-(29,30,31) | MF771037.1 | 29 \| 30 \| 31 | 3 |
| 19 | Colombia/-(3,4,5,6) | MF771046.1 | 3 \| 4 \| 5 \| 6 | 4 |
| 20 | Colombia/-(32,33,34) | MF771036.1 | 32 \| 33 \| 34 | 3 |
| 21 | Colombia/-(35,36,37) | MF771035.1 | 35 \| 36 \| 37 | 3 |
| 22 | Colombia/-(40,21,Q,R) | MF771033.1 | 40 \| 21 \| Q \| R | 4 |
| 23 | Colombia/-(41,42,43,44) | MF771032.1 | 41 \| 42 \| 43 \| 44 | 4 |
| 24 | Colombia/-(45,46,38) | MF771031.1 | 45 \| 46 \| 38 | 3 |
| 25 | Colombia/-(7,8,9) | MF771045.1 | 7 \| 8 \| 9 | 3 |
| 26 | Colombia/-(σ,μ,τ) | MF771048.1 | σ \| μ \| τ | 3 |
| 27 | Colombia/-(Φ,1,2) | MF771047.1 | Φ \| 1 \| 2 | 3 |
| 28 | Cuba/Western Cuba/-($15^{3}$, 49, 50) | MK809373.1 | 15 \| 15 \| 15 \| 49 \| 50 | 5 |
| 29 | Cuba/Western Cuba/-($38^{2}$, 47) | MK809370.1 | 38 \| 38 \| 47 | 3 |
| 30 | Cuba/Western Cuba/-($48, 15, 51$) | MK809375.1 | 48 \| 15 \| 51 | 3 |
| 31 | Cuba/Western Cuba/-(48, 15, 8, 54) | MK809378.1 | 58 \| 15 \| 8 \| 54 | 4 |
| 32 | Cuba/Western Cuba/-($48, 15, 8$) | MK809374.1 | 48 \| 15 \| 8 | 3 |
| 33 | Cuba/Western Cuba/-(48, $15^{3}$, 10) | MK809376.1 | 48 \| 15 \| 15 \| 15 \| 10 | 5 |
| 34 | Cuba/Western Cuba/-($48, 15^{3}$) | MK809372.1 | 48 \| 15 \| 15 \| 15 | 4 |
| 35 | Cuba/Western Cuba/-($48^{3}$) | MK809371.1 | 48 \| 48 \| 48 | 3 |
| 36 | Cuba/Western Cuba/-(A, S, 52, 53) | MK809377.1 | A \| S \| 52 \| 53 | 4 |
| 37 | Ecuador/Loja/-( ${67}^{6}$, 68) | MF288033.1 | 67 \| 67 \| 67 \| 67 \| 67 \| 67 \| 68 | 7 |
| 38 | Ecuador/Loja/-(${60}^{2}$) | MF288036.1 | 60 \| 60 | 2 |
| 39 | Ecuador/Loja/-(61, 62, 63, 62, 64) | MF288032.1 | 61 \| 62 \| 63 \| 62 \| 64 | 5 |
| 40 | Ecuador/Loja/-(65, 66, ${60}^{2}$) | MF288034.1 | 65 \| 66 \| 60 \| 60 | 4 |
| 41 | Ecuador/Loja/-($Q^{3}$) | MF288037.1 | Q \| Q \| Q | 3 |
| 42 | Ecuador/Zamora-Chinchipe/-(${55}^{2}$,56) | MF288044.1 | 55 \| 55 \| 56 | 3 |
| 43 | Ecuador/Zamora-Chinchipe/-(99,141,57) | MF288043.1 | 99 \| 141 \| 57 | 3 |
| 44 | Ecuador/Zamora-Chinchipe/-(L, 52, 58) | MF288041.1 | L \| 52 \| 58 | 3 |
| 45 | Ecuador/Zamora-Chinchipe/-($Q^{2}$, 57) | MF288039.1 | Q \| Q \| 57 | 3 |
| 46 | Ecuador/Zamora-Chinchipe/-($Q^{2}$, ${59}^{2}$) | MF288038.1 | Q \| Q \| 59 \| 59 | 4 |
| 47 | Ecuador/Zamora-Chinchipe/-($Q^{3}$, 57) | MF288040.1 | Q \| Q \| Q \| 57 | 4 |
| 48 | Egypt/-$\left( 40^{2}, 73, 74 \right)$ | MN311244.1 | 40 \| 40 \| 73 \| 74 | 4 |
| 49 | Egypt/-$\left( {40}^{3}, 74 \right)$ | MN311243.1 | 40 \| 40 \| 40 \| 74 | 4 |
| 50 | Egypt/-$\left( 75^{2},76, 77 \right)$ | MN311245.1 | 75 \| 75 \| 76 \| 77 | 4 |
| 51 | Egypt/-$\left( 78, {79}^{2}, 80 \right)$ | MN273314.1 | 78 \| 79 \| 79 \| 80 | 4 |
| 52 | Egypt/-$\left( {81}^{2}, 82, 83, 84 \right)$ | MN273313.1 | 81 \| 81 \| 82 \| 83 \| 84 | 5 |
| 53 | Egypt/-$\left( 85,81,76 \right)$ | MN273312.1 | 85 \| 81 \| 76 | 3 |
| 54 | Egypt/El-Wady El-Geded/-$\left( 10, 15, 8 \right)$ | MN370071.1 | 10 \| 15 \| 8 | 3 |
| 55 | Egypt/El-Wady El-Geded/-$\left( {15}^{3},8 \right)$ | MN3700761 | 15 \| 15 \| 15 \| 8 | 4 |
| 56 | Egypt/El-Wady El-Geded/-$\left( 48, {15}^{3}, 10 \right)$ | MN370072.1 | 48 \| 15 \| 15 \| 15 \| 10 | 5 |
| 57 | Egypt/El-Wady El-Geded/-$\left( 69, 70, 71,72 \right)$ | MN370077.1 | 69 \| 70 \| 71 \| 72 | 4 |
| 58 | Egypt/ElMinia/-$\left( 84,86, {87}^{3}, 88 \right)$ | MN257057.1 | 84 \| 86 \| 87 \| 87 \| 87 \| 88 | 6 |
| 59 | Egypt/ElMinia/-$\left( 85, 89, 90, 85 \right)$ | MN257056.1 | 85 \| 89 \| 90 \| 85 | 4 |
| 60 | Egypt/ElMinia/-$\left( 91, 85,81,76 \right)$ | MN257055.1 | 91 \| 85 \| 81 \| 76 | 4 |
| 61 | Mexico/Chiapas/-(A, $S^{2}$, 52, 53) | JN564635.1 | A \| S \| S \| 52 \| 53 | 5 |
| 62 | Mexico/Chiapas/-($A^{3}$, 92, 93) | EF053268.1 | A \| A \| A \| 92 \| 93 | 5 |
| 63 | Mexico/Hidalgo/-(69, 70, 71, 94) | JN564642.1 | 69 \| 70 \| 71 \| 94 | 4 |
| 64 | Mexico/Veracruz/-($A^{2}$, 52, 53) | AF345870.1 | A \| A \| 52 \| 53 | 4 |
| 65 | Philippines/Batangas/-(104, 10^3^, 75^3^, 107) | KC181903.1 | 104 \| 10 \| 75 \| 75 \| 10 \| 75 \| 107 \| 10 | 8 |
| 66 | Philippines/Batangas/-(108^4^, 109) | KC181902.1 | 108 \| 108 \| 108 \| 108 \| 109 | 5 |
| 67 | Philippines/Batangas/-(111, 112, 113) | KC181867.1 | 111 \| 112 \| 113 | 3 |
| 68 | Philippines/Batangas/-(114, S^2^, 52^2^, 92, 53) | KC181866.1 | 114 \| S \| 52 \| 92 \| S \| 52 \| 53 | 7 |
| 69 | Philippines/Batangas/-(Q^2^, R) | KC181868.1 | Q \| Q \| R | 3 |
| 70 | Philippines/Cavite/-(104, 105, 75) | KC181906.1 | 104 \| 105 \| 75 | 3 |
| 71 | Philippines/Cavite/-(106, 40^2^) | KC181904.1 | 106 \| 40 \| 40 \| 101 | 3 |
| 72 | Philippines/Cavite/-(Q, R | KC181897.1 | Q \| R | 2 |
| 73 | Philippines/Iloilo/-(102, 10^2^) | KC181907.1 | 102 \| 10 \| 10 \| 103 | 3 |
| 74 | Philippines/Iloilo/-(110^3^, 19^2^) | KC181898.1 | 110 \| 19 \| 19 \| 110 \| 110 \| 101 | 5 |
| 75 | Philippines/Iloilo/-(95, 54) | KC181914.1 | 95 \| 54 \| 96 | 2 |
| 76 | Philippines/Iloilo/-(Q^2^, R) | KC181915.1 | Q \| Q \| R | 3 |
| 77 | Philippines/Negros Occidental/-(19^2^ | KC181908.1 | 19 \| 19 \| 101 | 2 |
| 78 | Philippines/Negros Occidental/-(98, 97, H) | KC181910.1 | 98 \| 97 \| H | 3 |
| 79 | Philippines/Negros Occidental/-(99, 100) | KC181911.1 | 99 \| 100 | 2 |
| 80 | Philippines/Negros Oriental/-(97, H) | KC181913.1 | 97 \| H | 2 |
| 81 | Russia/Moscow/ - (${147}^{2})$ | MG570150 | 147 \| 147 | 2 |
| 82 | Russia/Moscow/ - (148, 147) | MG570151 | 148 \| 147 | 2 |
| 83 | Russia/Moscow/ - (148, ${147}^{5})$ | MG570152 | 148 \| 147 \| 147 \| 147 \| 147 \| 147 | 6 |
| 84 | Russia/Moscow/ - (149, 150) | MG570154 | 149 \| 150 | 2 |
| 85 | Russia/Moscow/ - (149, ${150}^{2}$) | MG570155 | 149 \| 150 \| 150 | 3 |
| 86 | Russia/Moscow/ - (149, ${150}^{3}$) | MG570156 | 149 \| 150 \| 150 \| 150 | 4 |
| 87 | Russia/Moscow/ - (${150}^{3})$ | MG570153 | 150 \| 150 \| 150 | 3 |
| 88 | Russia/Moscow/ - (151,152) | MG570157 | 151 \| 152 | 2 |
| 89 | Russia/Moscow/ -(${152}^{4}$) | MG570159 | 152 \| 152 \| 152 \| 152 | 4 |
| 90 | Russia/Saratov/-$(154^{5}$) | MG570160.1 | 154 \| 154 \| 154 \| 154 \| 154 | 5 |
| 91 | South Africa/Mpumalanga/-(130, 131, 132, 133, 134) | OQ384906.1 | 130 \| 131 \| 132 \| 133 \| 134 | 5 |
| 92 | South Africa/Mpumalanga/-(135, 136, 132, 133, 136, 132, 133, 137) | OQ384901.1 | 135 \| 136 \| 132 \| 133 \| 136 \| 132 \| 133 \| 137 | 8 |
| 93 | South Africa/Mpumalanga/-(135, 136, 132, 133, 137) | OQ384898.1 | 135 \| 136 \| 132 \| 133 \| 137 | 5 |
| 94 | South Africa/Mpumalanga/-(135, 136, 132, 137) | OQ384904.1 | 135 \| 136 \| 132 \| 137 | 4 |
| 95 | South Africa/Mpumalanga/-(138, 132, 137) | OQ384903.1 | 138 \| 132 \| 137 | 3 |
| 96 | South Africa/Mpumalanga/-(139, 140, 141, 142) | OQ384894.1 | 139 \| 140 \| 141 \| 142 | 4 |
| 97 | South Africa/Mpumalanga/-(143, 144, 145, 143, 146) | OQ384890.1 | 143 \| 144 \| 145 \| 143 \| 146 | 5 |
| 98 | Spain/Southern Spain/-($59^{3}$, 115, 116) | MT664982.1 | 59 \| 59 \| 59 \| 115 \| 116 | 5 |
| 99 | Thailand/-(155,$38^{2}$,157) | ON489300.1 | 155 \| 38 \| 38 \| 157 | 4 |
| 100 | Thailand/-(${156}^{5}$) | ON489292.1 | 156 \| 156 \| 156 \| 156 \| 156 | 5 |
| 101 | Thailand/-($157,38$) | ON489290.1 | 157 \| 38 | 2 |
| 102 | Thailand/-(${157}^{2},38$) | ON489289.1 | 157 \| 157 \| 38 | 3 |
| 103 | Thailand/-(${157}^{3},38$) | ON489288.1 | 157 \| 157 \| 157 \| 38 | 4 |
| 104 | Thailand/-(${157}^{3},{38}^{2}$) | ON489287.1 | 157 \| 157 \| 157 \| 38 \| 38 | 5 |
| 105 | Thailand/-(${157}^{5},38$) | ON489291.1 | 157 \| 157 \| 157 \| 157 \| 157 \| 38 | 6 |
| 106 | Thailand/-($38^{2}$, Q, 69) | ON489301.1 | 38 \| 38 \| Q \| 69 | 4 |
| 107 | Thailand/-(${38}^{3}$) | ON489286.1 | 38 \| 38 \| 38 | 3 |
| 108 | Thailand/-($69^{2}$, 38) | ON489302.1 | 69 \| 69 \| 38 | 3 |
| 109 | Thailand/-($69^{2}$) | ON489299.1 | 69 \| 69 \| 69 \| 69 \| 69 | 5 |
| 110 | Thailand/-(P,$38^{2}$, Q) | ON489296.1 | P \| 38 \| 38 \| Q | 4 |
| 111 | Thailand/-($Q^{4}$, 38) | ON489298.1 | Q \| Q \| Q \| Q \| 38 | 5 |
| 112 | Turkey/Elazig/-(12$0^{4}$, 121, 84) | MF787745.1 | 120 \| 120 \| 120 \| 120 \| 121 \| 84 | 6 |
| 113 | Turkey/Elazig/-(122,123,124) | MF787743.1 | 122 \| 123 \| 124 | 3 |
| 114 | Turkey/Elazig/-(1${22}^{2}$, 8$4^{2}$) | MF787744.1 | 122 \| 122 \| 84 \| 84 | 4 |
| 115 | Turkey/Elazig/-(125, 126) | MF377388.1 | 125 \| 126 | 2 |
| 116 | Turkey/Elazig/-(127,12$8^{2},$,129) | MG983514.1 | 127 \| 128 \| 128 \| 129 | 4 |
| 117 | Turkey/Elazig/-($18$, 4$7^{2}$) | MF787746.1 | 18 \| 47 \| 47 | 3 |
| 118 | Turkey/Elazig/-($78$, 117,118,119) | MF787747.1 | 78 \| 117 \| 118 \| 119 | 4 |
| 119 | Turkey/Elazig/-(8$4^{3},$87, 88) | MF377394.1 | 84 \| 84 \| 84 \| 87 \| 88 | 5 |
| 120 | Turkey/Elazig/-(A, $S^{5},58$) | MF787742.1 | A \| S \| S \| S \| S \| S \| 58 | 7 |
