# Supplementary material for: Nomenclature, classification, and in-silico structural analysis of MSP1a variants of Anaplasma marginale
Source: PLoS One. 2026 Oct 21;21(10):e0360505. doi: 10.1371/journal.pone.0360505 (PMC13686480; doi:10.1371/journal.pone.0360505)
Supplement: Supplementary file 4 [file pone.0360505.s004.docx]

**Occurrence of Structure**

| **Strains** | **Structure of MSP1a Tandem Repeats** | **Number of strains** | **World Occurrence** |
| --- | --- | --- | --- |
| **Most Common** | 125 126 | 8 | 8x Turkey |
| **Second Common** | 48 15 15 15 10 | 5 | 4x Egypt, 1x Cuba |
| **Third Common** | 156 156 156 156 156 | 4 | 4x Thailand |
| **Third Common** | 154 154 154 154 154 | 4 | 4x Russia |
|  | Q R | 3 | 3x Philippines |
|  | 127 128 128 129 | 3 | 3x Turkey |
|  | Q Q R | 2 | 2x Philippines |
|  | 98 97 H | 2 | 2x Philippines |
|  | 40 73 40 74 | 2 | 2x Egypt |
|  | Q Q 57 | 2 | 2x Ecuador |
|  | 135 136 132 133 136 132 133 137 | 2 | 2 x South Africa |
|  | 95 54 96 | 1 | 1x Philippines |
|  | 97 H | 1 | 1x Philippines |
|  | 99 100 | 1 | 1x Philippines |
|  | 19 19 101 | 1 | 1x Philippines |
|  | 102 10 10 103 | 1 | 1x Philippines |
|  | 104 105 75 | 1 | 1x Philippines |
|  | 106 40 40 10 | 1 | 1x Philippines |
|  | 104 10 75 75 10 75 107 10 | 1 | 1x Philippines |
|  | 108 108 108 108 109 | 1 | 1x Philippines |
|  | 110 19 19 110 110 101 | 1 | 1x Philippines |
|  | 111 112 113 | 1 | 1x Philippines |
|  | 114 S 52 92 S 52 53 | 1 | 1x Philippines |
|  | A B C | 1 | 1x Brazil |
|  | E F G H | 1 | 1x Brazil |
|  | J I J J K | 1 | 1x Brazil |
|  | L M N O | 1 | 1x Brazil |
|  | Q P Q Q R | 1 | 1x Brazil |
|  | A S S T U | 1 | 1x Brazil |
|  | A S C V | 1 | 1x Brazil |
|  | Q P Q W | 1 | 1x Brazil |
|  | J α β Γ | 1 | 1x Brazil |
|  | 69 70 71 72 | 1 | 1x Egypt |
|  | 15 15 15 8 | 1 | 1x Egypt |
|  | 10 15 8 | 1 | 1x Egypt |
|  | 75 76 75 77 | 1 | 1x Egypt |
|  | 40 40 40 74 | 1 | 1x Egypt |
|  | 78 79 79 80 | 1 | 1x Egypt |
|  | 81 81 82 83 84 | 1 | 1x Egypt |
|  | 85 81 76 | 1 | 1x Egypt |
|  | 84 86 87 87 87 88 | 1 | 1x Egypt |
|  | 59 59 59 115 | 1 | 1x Egypt |
|  | 91 85 81 76 | 1 | 1x Egypt |
|  | 55 55 56 | 1 | 1x Ecuador |
|  | 99 141 57 | 1 | 1x Ecuador |
|  | L 52 58 | 1 | 1x Ecuador |
|  | Q Q Q 57 | 1 | 1x Ecuador |
|  | Q Q 59 59 | 1 | 1x Ecuador |
|  | Q Q Q | 1 | 1x Ecuador |
|  | 60 60 | 1 | 1x Ecuador |
|  | 61 62 63 64 | 1 | 1x Ecuador |
|  | 65 66 60 60 | 1 | 1x Ecuador |
|  | 67 67 67 67 67 67 68 | 1 | 1x Ecuador |
|  | 61 62 63 62 64 | 1 | 1x Ecuador |
|  | $78$ 117 118 119 | 1 | 1x Turkey |
|  | $18$ 47, 47 | 1 | 1x Turkey |
|  | 120 120 120 120 121 84 | 1 | 1x Turkey |
|  | 122 122 84 84 | 1 | 1x Turkey |
|  | 122 123 124 | 1 | 1x Turkey |
|  | A S S S S S 58 | 1 | 1x Turkey |
|  | 84 84 84 87 88 | 1 | 1x Turkey |
|  | 152 152 152 152 | 1 | 1x Russia |
|  | 153 153 153 | 1 | 1x Russia |
|  | 154 154 154 154 | 1 | 1x Russia |
|  | 151 152 | 1 | 1x Russia |
|  | 149 150 150 150 | 1 | 1x Russia |
|  | 149 150 150 | 1 | 1x Russia |
|  | 149 150 | 1 | 1x Russia |
|  | 150 150 150 | 1 | 1x Russia |
|  | 148 147 147 147 147 147 | 1 | 1x Russia |
|  | 148 147 | 1 | 1x Russia |
|  | 147 147 | 1 | 1x Russia |
|  | 69 38 69 | 1 | 1x Thailand |
|  | 38 38 Q 69 | 1 | 1x Thailand |
|  | 155 38 38 157 | 1 | 1x Thailand |
|  | $69 69$ 69 69 69 | 1 | 1x Thailand |
|  | $Q$Q Q Q 38 | 1 | 1x Thailand |
|  | P $38 38$ Q | 1 | 1x Thailand |
|  | 157 157 157 157 157 38 | 1 | 1x Thailand |
|  | $157 38$ | 1 | 1x Thailand |
|  | ${157}157 38$ | 1 | 1x Thailand |
|  | ${157}157 157 38$ | 1 | 1x Thailand |
|  | ${157}157 157 {38}38$ | 1 | 1x Thailand |
|  | ${38}$38 38 | 1 | 1x Thailand |
|  | 38 38 47 | 1 | 1x Cuba |
|  | 48 48 48 | 1 | 1x Cuba |
|  | 48 15 15 15 | 1 | 1x Cuba |
|  | 15 15 15 49 50 | 1 | 1x Cuba |
|  | 48 15 8 | 1 | 1x Cuba |
|  | A S 52 53 | 1 | 1x Cuba |
|  | 48 15 8 54 | 1 | 1x Cuba |
|  | A A A 92 93 | 1 | 1x Mexico |
|  | A S S 52 53 | 1 | 1x Mexico |
|  | 69 70 71 94 | 1 | 1x Mexico |
|  | A A 52 53 | 1 | 1x Mexico |
|  | 59 59 59 115 116 | 1 | 1x Spain |
|  | 130 131 132 133 134 | 1 | 1 x South Africa |
|  | 135 136 132 137 | 1 | 1 x South Africa |
|  | 138 132 137 | 1 | 1 x South Africa |
|  | 135 136 132 133 137 | 1 | 1 x South Africa |
|  | 139 140 141 142 | 1 | 1 x South Africa |
|  | 143 144 145 143 146 | 1 | 1 x South Africa |
|  | σ μ τ | 1 | 1x Colombia |
|  | Φ 1 2 | 1 | 1x Colombia |
|  | 3 4 5 6 | 1 | 1x Colombia |
|  | 7 8 9 | 1 | 1x Colombia |
|  | 10 11 12 | 1 | 1x Colombia |
|  | 13 14 | 1 | 1x Colombia |
|  | 15 16 17 | 1 | 1x Colombia |
|  | 18 19 20 | 1 | 1x Colombia |
|  | $21 21$R | 1 | 1x Colombia |
|  | 22 23 24 25 | 1 | 1x Colombia |
|  | 26 27 28 | 1 | 1x Colombia |
|  | 29 30 31 | 1 | 1x Colombia |
|  | 32 33 34 | 1 | 1x Colombia |
|  | 35 36 37 | 1 | 1x Colombia |
|  | 18 38 39 | 1 | 1x Colombia |
|  | 40 21 Q R | 1 | 1x Colombia |
|  | 41 42 43 44 | 1 | 1x Colombia |
|  | 45 46 38 | 1 | 1x Colombia |

**Occurrence of each Tandem Repeat**

| **Strains** | **Tandem Repeats** | **No. of times it occurs** | **World Occurrence** |
| --- | --- | --- | --- |
| **Most Common** | Q | 28 | 7x Philippines, 5x Brazil, 6x Thailand, 10x Ecuador |
| **Second Common** | 15 | 26 | 14x Egypt, 11x Cuba, 1x Colombia |
| **Third Common** | 154 | 24 | 24x Russia |
| **Fourth Common** | 38 | 20 | 17x Thailand, 2x Cuba, 1x Colombia |
| **Fourth Common** | 156 | 20 | 20x Thailand |
| **Fifth Common** | 157 | 15 | 15x Thailand |
|  | S | 13 | 2x Philippines, 3x Brazil, 1x Cuba, 2x Mexico, 5x Turkey |
|  | A | 11 | 3x Brazil, 1x Cuba, 6x Mexico, 1x Turkey |
|  | 48 | 11 | 4x Egypt, 7x Cuba |
|  | 10 | 10 | 6x Philippines, 2x Egypt, 1x Cuba, 1x Colombia |
|  | 69 | 10 | 8x Thailand, 1x Egypt, 1x Mexico |
|  | 40 | 9 | 2x Philippines, 6x Egypt, 1x Colombia |
|  | 84 | 9 | 2x Egypt, 7x Turkey |
|  | 150 | 9 | 9x Russia |
|  | R | 8 | 5x Philippines, 1x Brazil, 2x Colombia |
|  | 59 | 8 | 3x Egypt, 3x Spain 2x Ecuador |
|  | 75 | 8 | 4x Philippines, 4x Egypt |
|  | 147 | 8 | 8x Russia |
|  | 52 | 6 | 2x Philippines, 1x Cuba, 2x Mexico 1x Ecuador |
|  | 67 | 6 | 6x Ecuador |
|  | 132 | 6 | 6x South Africa |
|  | 8 | 5 | 2x Egypt, 2x Cuba, 1x Colombia |
|  | 19 | 5 | 4x Philippines, 1x Colombia |
|  | 81 | 5 | 5x Egypt |
|  | 152 | 5 | 5x Russia |
|  | H | 4 | 3x Philippines, 1x Brazil |
|  | J | 4 | 4x Brazil, |
|  | 53 | 4 | 1x Philippines, 1x Cuba, 2x Mexico |
|  | 60 | 4 | 4x Ecuador |
|  | 74 | 4 | 4x Egypt |
|  | 87 | 4 | 3x Egypt, 1x Turkey |
|  | 108 | 4 | 4x Philippines |
|  | 120 | 4 | 4x Turkey |
|  | 133 | 4 | 4x South Africa |
|  | 136 | 4 | 4x South Africa |
|  | 137 | 4 | 4x South Africa |
|  | P | 3 | 2x Brazil, 1x Thailand |
|  | 18 | 3 | 1x Turkey, 2x Colombia |
|  | 47 | 3 | 1x Cuba, 2x Turkey |
|  | 57 | 3 | 3x Ecuador |
|  | 62 | 3 | 3x Ecuador |
|  | 76 | 3 | 3x Egypt |
|  | 97 | 3 | 3x Philippines |
|  | 110 | 3 | 3x Philippines |
|  | 122 | 3 | 3x Turkey |
|  | 135 | 3 | 3x South Africa |
|  | 149 | 3 | 3x Russia |
|  | 153 | 3 | 3x Russia |
|  | C | 2 | 2x Brazil |
|  | L | 2 | 1x Brazil 1x Ecuador |
|  | 21 | 2 | 2x Colombia |
|  | 54 | 2 | 1x Philippines, 1x Cuba |
|  | 55 | 2 | 2x Ecuador |
|  | 58 | 2 | 1x Ecuador, 1x Turkey |
|  | 61 | 2 | 2x Ecuador |
|  | 63 | 2 | 2x Ecuador |
|  | 64 | 2 | 2x Ecuador |
|  | 70 | 2 | 1x Egypt, 1x Mexico |
|  | 71 | 2 | 1x Egypt, 1x Mexico |
|  | 73 | 2 | 2x Egypt |
|  | 78 | 2 | 1x Egypt, 1x Turkey |
|  | 79 | 2 | 2x Egypt |
|  | 85 | 2 | 2x Egypt |
|  | 88 | 2 | 1x Egypt, 1x Turkey |
|  | 92 | 2 | 1x Philippines,1x Mexico |
|  | 98 | 2 | 2x Philippines |
|  | 99 | 2 | 1x Philippines 1x Ecuador |
|  | 101 | 2 | 2x Philippines |
|  | 104 | 2 | 2x Philippines |
|  | 115 | 2 | 1x Egypt, 1x Spain |
|  | 128 | 2 | 2x Turkey |
|  | 141 | 2 | 1x South Africa, 1x Ecuador |
|  | 143 | 2 | 2x South Africa |
|  | 148 | 2 | 2x Russia |
|  | B | 1 | 1x Brazil |
|  | E | 1 | 1x Brazil |
|  | F | 1 | 1x Brazil |
|  | G | 1 | 1x Brazil |
|  | I | 1 | 1x Brazil |
|  | K | 1 | 1x Brazil |
|  | M | 1 | 1x Brazil |
|  | N | 1 | 1x Brazil |
|  | O | 1 | 1x Brazil |
|  | T | 1 | 1x Brazil |
|  | U | 1 | 1x Brazil |
|  | V | 1 | 1x Brazil |
|  | W | 1 | 1x Brazil |
|  | α | 1 | 1x Brazil |
|  | β | 1 | 1x Brazil |
|  | Γ | 1 | 1x Brazil |
|  | μ | 1 | 1x Colombia |
|  | σ | 1 | 1x Colombia |
|  | τ | 1 | 1x Colombia |
|  | Φ | 1 | 1x Colombia |
|  | 1 | 1 | 1x Colombia |
|  | 2 | 1 | 1x Colombia |
|  | 3 | 1 | 1x Colombia |
|  | 4 | 1 | 1x Colombia |
|  | 5 | 1 | 1x Colombia |
|  | 6 | 1 | 1x Colombia |
|  | 7 | 1 | 1x Colombia |
|  | 9 | 1 | 1x Colombia |
|  | 11 | 1 | 1x Colombia |
|  | 12 | 1 | 1x Colombia |
|  | 13 | 1 | 1x Colombia |
|  | 14 | 1 | 1x Colombia |
|  | 16 | 1 | 1x Colombia |
|  | 17 | 1 | 1x Colombia |
|  | 20 | 1 | 1x Colombia |
|  | 22 | 1 | 1x Colombia |
|  | 23 | 1 | 1x Colombia |
|  | 24 | 1 | 1x Colombia |
|  | 25 | 1 | 1x Colombia |
|  | 26 | 1 | 1x Colombia |
|  | 27 | 1 | 1x Colombia |
|  | 28 | 1 | 1x Colombia |
|  | 29 | 1 | 1x Colombia |
|  | 30 | 1 | 1x Colombia |
|  | 31 | 1 | 1x Colombia |
|  | 32 | 1 | 1x Colombia |
|  | 33 | 1 | 1x Colombia |
|  | 34 | 1 | 1x Colombia |
|  | 35 | 1 | 1x Colombia |
|  | 36 | 1 | 1x Colombia |
|  | 37 | 1 | 1x Colombia |
|  | 39 | 1 | 1x Colombia |
|  | 41 | 1 | 1x Colombia |
|  | 42 | 1 | 1x Colombia |
|  | 43 | 1 | 1x Colombia |
|  | 44 | 1 | 1x Colombia |
|  | 45 | 1 | 1x Colombia |
|  | 46 | 1 | 1x Colombia |
|  | 49 | 1 | 1x Cuba |
|  | 50 | 1 | 1x Cuba |
|  | 56 | 1 | 1x Ecuador |
|  | 65 | 1 | 1x Ecuador |
|  | 66 | 1 | 1x Ecuador |
|  | 68 | 1 | 1x Ecuador |
|  | 72 | 1 | 1x Egypt |
|  | 77 | 1 | 1x Egypt |
|  | 80 | 1 | 1x Egypt |
|  | 82 | 1 | 1x Egypt |
|  | 83 | 1 | 1x Egypt |
|  | 86 | 1 | 1x Egypt |
|  | 91 | 1 | 1x Egypt |
|  | 93 | 1 | 1x Mexico |
|  | 94 | 1 | 1x Mexico |
|  | 95 | 1 | 1x Philippines |
|  | 96 | 1 | 1x Philippines |
|  | 100 | 1 | 1x Philippines |
|  | 102 | 1 | 1x Philippines |
|  | 103 | 1 | 1x Philippines |
|  | 105 | 1 | 1x Philippines |
|  | 106 | 1 | 1x Philippines |
|  | 107 | 1 | 1x Philippines |
|  | 109 | 1 | 1x Philippines |
|  | 111 | 1 | 1x Philippines |
|  | 112 | 1 | 1x Philippines |
|  | 113 | 1 | 1x Philippines |
|  | 114 | 1 | 1x Philippines |
|  | 116 | 1 | 1x Spain |
|  | 117 | 1 | 1x Turkey |
|  | 118 | 1 | 1x Turkey |
|  | 119 | 1 | 1x Turkey |
|  | 121 | 1 | 1x Turkey |
|  | 123 | 1 | 1x Turkey |
|  | 124 | 1 | 1x Turkey |
|  | 125 | 1 | 1x Turkey |
|  | 126 | 1 | 1x Turkey |
|  | 127 | 1 | 1x Turkey |
|  | 129 | 1 | 1x Turkey |
|  | 130 | 1 | 1x South Africa |
|  | 131 | 1 | 1x South Africa |
|  | 134 | 1 | 1x South Africa |
|  | 138 | 1 | 1x South Africa |
|  | 139 | 1 | 1x South Africa |
|  | 140 | 1 | 1x South Africa |
|  | 142 | 1 | 1x South Africa |
|  | 144 | 1 | 1x South Africa |
|  | 145 | 1 | 1x South Africa |
|  | 146 | 1 | 1x South Africa |
|  | 151 | 1 | 1x Russia |
|  | 155 | 1 | 1x Thailand |
