# Supplementary material for: Nomenclature, classification, and in-silico structural analysis of MSP1a variants of Anaplasma marginale
Source: PLoS One. 2026 Oct 21;21(10):e0360505. doi: 10.1371/journal.pone.0360505 (PMC13686480; doi:10.1371/journal.pone.0360505)
Supplement: Supplementary file 5 [file pone.0360505.s007.docx]

| **Name of Tandem Repeats** | **2D Structure of Tandem repeats** | **Sequence of The Tandem Repeats** |
| --- | --- | --- |
| A | c-c | -------SGVLSQSGQASTSSQLGTDSSSAGDQQQ------- |
| B | c-c | -------SGVSSQSGQASTSSQLGADSSSAGGQQQ------- |
| C | c-c | -------SSVLSPSGQASTSSQLGADSSSASGQQQ------- |
| D | c-c | -------------------SGVLSQSGQ-AST---------- |
| E | a-c | --------DSSSASGQQQESSVLPQSDQ-ASTSSQLG----- |
| F | a-c | --------DSSPASGQQQESSVLPQSDQ-ASTSSQLG----- |
| G | a-c | --------GSSSASGQQQESSVLPQSGQ-ASTSSQLG----- |
| H | c-c | --------DSSSASGQQQESSVLSQSG--ASTSSQL------ |
| I | a-c | --------DSSSASGQQQESGVLSQSGQ-ASTSSQLG----- |
| J | a-c | --------DSSSASGQQQESSVLSPSGQ-ASTSSQLG----- |
| K | c-c | --------DSSSASGQQQESGVLSQSGQ-ASTSSQL------ |
| L | c-c | -------SGVLSQSGQASTSSQLGTDSSSASGQQQ------- |
| M | c-c | -------SSVLPQSGQASTSSQLGAGSSSASGQQQ------- |
| N | c-c | -------SSVLSQSDQASTSSQLGTDSSSASGQQQ------- |
| O | c-c | -------------------SSVLSQSG--ASTSSQ------- |
| P | c-c | --------DSSSASGQQQESSVLPQSGQ-ASTSSQLG----- |
| Q | a-c | --------DSSSASGQQQESSVLSQSDQ-ASTSSQLG----- |
| R | c-c | --------DSSSASGQQQESSVLSQSDQ-ASTSSQL------ |
| S | c-c | -------SGVSSQSGQASTSSQLGTDSSSAGDQQQ------- |
| T | c-c | -------SGVSSQSDQASTSSQLGTDSSSASGQQQ------- |
| U | a-c | -------------------SSVLSQSDQ-ASTSSQSGTD--- |
| V | c-c | -------------------SGVLSQSGQ-ASTSSQLGTD--- |
| W | c-c | --------DSSSASGQQQESSVSSQSDQ-ASTSSQL------ |
| α | c-c | --------DNSSASGQQPESSVLSPST--SSQLGEWSG---- |
| β | c-c | --------DSSSASGQQPESSVLSPSAQ-ASTSSQLG----- |
| Γ | a-a | -------TDNSSASGQQQESSVSSQSAQ-ASTSSQLG----- |
| μ | c-c | --------DSSSASGQQQESSVSSQSD--EVSTSSQLG---- |
| π | a-a | -----------------STSSQLGTGWRQEMRSKVASDE--- |
| Σ | a-c | -----------------STSSQLGADLRQEVHSKVASVE--- |
| σ | α-α | --------DSSSASDQQQESSVSSQSGQ-ASTSSQLGA---- |
| τ | β-c | --------ITRQSGGQQQESSVSSQSGQ-ASTSSQSGA---- |
| Φ | α-c | -----------SASGQQQESSVSSQSDQASSTSSQLGADSS- |
| 1 | α-c | -----------SASGQQQESSVLSQSDQASSTSSQLGADSS- |
| 2 | α-c | -----------SESGQQQESSVSSQSDQ-ASTSSQSGAD--- |
| 3 | α-α-α | --------SSSASGQQQSESSVLSQSGQ-ASTSSQLGAD--- |
| 4 | α-c | ------TDSSSSASGQQQESSVLSQSDQASSTSSQL------ |
| 5 | α-α | ---------SSSASGQQQESGVLSQSDQ-ASTSSQLGAD--- |
| 6 | α-c | ------TDSSSSASGQQQESSVLSQSDQASSTSSQL------ |
| 7 | c-c | -------ADISSASGQQQESSVSSQSDQ-ASTSSQL------ |
| 8 | c-c | -------ADSSSAGGQQQESSVSSQSGQ-ASTSSQL------ |
| 9 | c-c | -------TDSSSASGQQQESSVSSQSGQ-ASTSSQS------ |
| 10 | c-c | -------ADSSSASGQQQESSVSSQSG--QASTSSQL----- |
| 11 | c-c | -------ADSSSASGQQQESSVSSQSDQ-ASTSSQL------ |
| 12 | c-c | -------TDTSSASGQQQESSVSSQSGQ-ASTSSQS------ |
| 13 | α-c | ------TDSSSSASGQQQESSVLSQSDQASSTSSQL------ |
| 14 | c-c | -------ADSSSASGQQQESSVLSQSDQ-ASTSSQL------ |
| 15 | c-c | -------ADSSSAGGQQQESSVSSQSDQ-ASTSSQL------ |
| 16 | c-c | -------ADSSSAGGQHQESSVSSQSDQ-ASTSSQL------ |
| 17 | c-c | -------PDTSSAGGHHHKSSVSSQSGQ-ASTSSQLG----- |
| 18 | α-c | --------DSSSASGQQQESGVLSQSGQ-ASTSSQLG----- |
| 19 | α-c | --------DSSSAGDQQQESSVSSQSD--ASTSSQLG----- |
| 20 | α-c | -------DSSSAGDQQQESSVSSQSGH-ASTSSQLG----- |
| 21 | α-c | --------DSSSASGQQQESSVLSQSSQ-ASTSSQLG----- |
| 22 | α-c | --------DNSSARGHQQENSVVSPSGQ-ASTSSQLG----- |
| 23 | α-c | --------DSSSASDQQQESSVLSQSNQ-ASTSSQLG----- |
| 24 | α-α | --------DSSSASDQQQESSVLSQSDQ-ASTSSQLG----- |
| 25 | c-c | --------YCSSAGGQQQESSVSSQSGQ-ASTSSQL------ |
| 26 | α-c | --------DSSSAGDQQQESSVLSQSGQASSTSSQLG----- |
| 27 | α-c | --------DSSSASDQQQESSVLSQSNQ-ASTSSLLG----- |
| 28 | α-c | --------NSSSASDQQQDSIVLSQSDQ-ASTSSQL------ |
| 29 | α-α-α | ---------ARQLGGQQQESSVLSQSDQASSTSSQLGAD--- |
| 30 | α-c | -----TDSSSSASGQQQESSVLSQSDQASSTSSQL------ |
| 31 | α-α-α | -----------SSASGQQESSVLSQSDQ-ASTSSQSGTD--- |
| 32 | α-c | -----------SASGQQQESSVLSQSDQASSTSSQLGADSS- |
| 33 | α-c | -----------SASGQQQESSVLSQSDQ-ASTSSQSGTDSS- |
| 34 | c-c | -----------SASGQQQESSVLSQSDQ-ASTSSQLGA---- |
| 35 | α-c | ------TDSSSAGDQQQESSVLSQSDQ-ASTSSQLG----- |
| 36 | α-c | -------ADSSSASGQQQESSVLSQSAQ-ASTSSQLG----- |
| 37 | α-c | --------DNSSASDQQQESSVSSQSGR-ASTSSQLG----- |
| 38 | c-c | --------DSSSASGQQQESSVSSQSGQ-ASTSSQLG----- |
| 39 | α-c | --------DSSSASGQQQESSVLSQSGQ-ASTSSQSG----- |
| 40 | α-c | --------DSSSASGQQQESSVLSQSGQ-ASTSSQLG----- |
| 41 | α-c | ---------SSSASGQQQGSGVSYQSGQ-ASTSINLETD--- |
| 42 | α-α | ---------SSSAGGQQQESSVLSQSGQ-ASTSSQLGAD--- |
| 43 | α-α | ---------SSSAGDQQQESSVSSQSGQ-ASTSSQLGTD--- |
| 44 | α-c | ---------SSSASGQQQESSVLSQSGQ-ASTSSQL------ |
| 45 | α-c | --------DSSSAGDQQQESSVLSQSGQASSTSSQLG----- |
| 46 | α-c | --------DSSSASDQQQERGVLSQSGQ-ASTSSQLG----- |
| 47 | c-c | --------DSSSASGQQQESSVSSQSDQ-ASTSSQLG----- |
| 48 | c-c | -------AGSSSAGGQQQESSVSSQSDQ-ASTSSQL------ |
| 49 | α-c | -------ADSSSASGQQQESSVLSPSGQ-ASTSSQLGV---- |
| 50 | α-c | -------ADSSSASGQQQESSVSSQSD--ASTSSQ------- |
| 51 | c-c | -------ADSSSAGGQQQESSVSSQSDQ-ASTSSQS------ |
| 52 | c-c | -------SGVSSQSGQASTSSQLGTDSSSASGQQQ------- |
| 53 | α-c-α | -------------------SSVSSQSD--ASTSSQLGAD--- |
| 54 | α-c | -------TDSSSASGQQQESSVSSQSGQ-ASTSSQL------ |
| 55 | α-c | -------------SGQQQESSVLSQSDQ-ASTSSQLGTDSSS |
| 56 | c-c | -------------SGQQQESSVLSQSDQ-ASTSSQSGTD--- |
| 57 | α-α | --------DSSSASGQQQESSVLSQSDQ-ASTSSQSG----- |
| 58 | c-c | -------------------SGVSSQSGQ-ASTSSQLGTD--- |
| 59 | α-c | --------DSSSASGQQQESSVSSQSE--ASTSSQLG----- |
| 60 | α-c | ---------SSSASGQQQESSVSSQSE--ASTSSQLGT---- |
| 61 | c-c | -------AGSSSAGGQQQESSVSSQSE--ASTSSQLG----- |
| 62 | c-c | -------ADSSSASGQQQESSVLSQSE--ASTSSQLG----- |
| 63 | c-c | -------ADSSSASGQQQESSVSSQSE--ASTSSQLG----- |
| 64 | c-c | -------ADSSSASGQQQESSVSSQSE--ASTSSQL------ |
| 65 | α-c | ---------SSSASGQQQESSVSSQSE--ASTSSQLGA---- |
| 66 | c-c | ---------SSSASGQQQESSVSSQSDQ-ASTSSQLGT---- |
| 67 | α-c | ---------SSSASGQQQESSVSSQSE--ASTSSQLGGA--- |
| 68 | c-c | ---------SSSASGQQQESSVSSQSE--ASTSSQL------ |
| 69 | c-c | --------DSSSASGQQQESSVLSQSG--ASTSSQL------ |
| 70 | c-c | -------ADSSSASGQQQESSVPSQSG--ASTSSQL------ |
| 71 | c-c | -------ADSSSASGQQQESGVPSQSG--ASTSSQL------ |
| 72 | β-c | -------------------SSVSSQSD--ASTSSQLGAD--- |
| 73 | α-α | --------DSSSAGNQQQESSVSSQSD--ASTSSQLGA---- |
| 74 | α-c | --------DSSSAGNQQQESSVSSQSD--ASTSSQLGT---- |
| 75 | α-c | --------DSSSAGNQQQESSVSSQSD--ASTSSQLG----- |
| 76 | c-c | --------DSSSAGNQQQESSVSSQSD--ASTSSQSGA---- |
| 77 | α-c | --------DSSSAGDQQQESSVSSQSD--ASTSSQLG----- |
| 78 | α-c | ---------SSSASGQQQESSVLSQGGQ-ASTSSQLGT---- |
| 79 | c-c | --------DSSSAGNQQQESSVSSQSG--ASTSSQLGT---- |
| 80 | α-α | ---------SSSASGQQQESSVSSQSE--ASTSSQLGT---- |
| 81 | c-c | ---------SSSASGQQQESSVSSQSE--ASTSSQL------ |
| 82 | c-c | --------DSSSASGQQQESSVSSQSE--ASTSSQLG----- |
| 83 | α-c | -------AGSSSAGGQQQESSVSSQSE--ASTSSQLG----- |
| 84 | c-c | -------ADSSSASGQQQESGVSSQSD--ASTSSQL------ |
| 85 | c-c | -------ADSSSASGQQQESSVSSQSE--ASTSSQLG----- |
| 86 | c-c | -------ADSSSASGQQQESSVSSQSE--ASTSSQL------ |
| 87 | c-c | -------ADSSSASGQQQESGVPSQSG--ASTSSQL------ |
| 88 | c-c | -------TDSSSASGQQQESGVSSQSD--ASTSSQL------ |
| 89 | c-c | -------ADSSSASGQQQESGVPSQSE--ASTSSQL------ |
| 90 | α-c | -------ADSSSASGQQQESGVSSQSE--ASTSSQL------ |
| 91 | α-c | ---------SSSASGQQQGGGVSPQSE--ASTSSQLGT---- |
| 92 | c-c | ----------------SSVSSQSDASTSSQLGTDSSSAGDQQQ-------------- |
| 93 | c-c | -------------------SGVSSQSGQ-ASTSSQLGT---- |
| 94 | α-c | -------TDSSSASGQQQESSVLSQSDQ-ASTSSQS------ |
| 95 | c-c | -------TDSSSASGQQQESSVLPQSGQ-ASTSSQL------ |
| 96 | N/A | -----------------------GTD---------------- |
| 97 | α-α | --------DSSSASGQQQESSVLSQSDQ-ASTLSQLG----- |
| 98 | α-α-α | --------DSSSASGQRQESSVLSQSDQ-ASTLSQLG----- |
| 99 | α-c | -------TDSSSASGQQQESSVLSQSDQ-ASTSSQLG----- |
| 100 | α-c | -------TDSSSASGQQQESSVLSQSG--ASTSSQLGT---- |
| 101 | N/A | ------------------------TD---------------- |
| 102 | α-c | -------ADSSSAGDQQQESSVLSQSGQ-ASTSSQL------ |
| 103 | N/A | -----------------------GAD---------------- |
| 104 | α-α | -------ADSSSASDQQQESGVPSQSE--ASTSSQL------ |
| 105 | c-c | -------ADSSSASGQQQESGVPSQSE--ASTSSQL------ |
| 106 | α-c | --------DSSSASDQQQESSVLSQSGQ-ASTSSQLG----- |
| 107 | c-c | -------TDSSSASGQQQESSVLSQSGQ-ASTSSQL------ |
| 108 | α-c | -------ADSSSASGQQQESSVSSQL---------------- |
| 109 | α-c | -------ADSSSASGQQQESSVSSQ----------------- |
| 110 | α-c | --------DSSSAGDQQQESSVSSQSG--ASTSSQLG----- |
| 111 | c-c | -------SGVLSKSDQASTSSQLGADSSSASGQQQ------- |
| 112 | α-c | -------SSVLSQSDQASTSSQLGADSSSASGQQQ------- |
| 113 | α-c | -------------------SSVLSQSDQ-ASTSSQL------ |
| 114 | α-c | -------SGVLSKSDQASTSSQLGTDSSSAGDQQQ------- |
| 115 | α-c | --------DSSSASGQQQESSVSSQSE--ASTSSHLG----- |
| 116 | c-c | --------DSSSASGQHQESTVSSQSD--ASTSAQLD----- |
| 117 | c-c | ---------SSSASGQQQGGGVSPQSE--ASTSSQLGT---- |
| 118 | α-c | ---------SSSASGQQQESGVSPQSE--ASTSSQLGT--- |
| 119 | α-c | ---------SSSASGQQQESGVSPQSE--ASTSSQL------ |
| 120 | α-c | --------DSSSASGQQQESSVSSQSD--ASTSSQLG----- |
| 121 | α-c | --------DSSSASGQQQESGVSSQSD--ASTSSQL------ |
| 122 | c-c | -------AGSSSASGQQQESGVSSQSD--ASTSSQL------ |
| 123 | α-α | ---------SSSASGQQQESGVPSQSGQ-ASTSSQLGA---- |
| 124 | α-c | ---------SSSASGQQQESGVSSQSD--ASTSSQLGA---- |
| 125 | α-α | --------DSSSAGDQQQESSVLSQSDQ-ASTSSQLG----- |
| 126 | α-c | --------DSSSAGDQQQESGVSSQSGQ-ASTSSQLG----- |
| 127 | c-c | --------GVLSQSGQASTSSQLGTDSSSAGDQQQE------ |
| 128 | c-c | --------GVSSQSGQASTSSQLGTDSSSAGDQQQE------ |
| 129 | c-c | --------GVSSQSGQASTSSQLGTDSSSAGDQQQE------ |
| 130 | α-c | --------DSSSAGNQQQESSVSSQSG--ASTSSQLGT---- |
| 131 | α-c | --------DSSSAGDQQQESSVLSPSGQ-ASTSSQSGT---- |
| 132 | α-α | --------DSSSASGQQQESSVLSQSSQ-ASTSSQLGA---- |
| 133 | α-c | --------DSSSAGNQQQESSVSSQSD--ASTSSQLGA---- |
| 134 | α-α | --------DSSSAGNQQQESSVSSQSD--ASTSSQLGT---- |
| 135 | α-c | --------DSSSASGQQQESSVLPQSD--ASTSSQLGA---- |
| 136 | α-c | --------DSSSAGNQQQESSVSSQSD--ASTSSQSGA---- |
| 137 | α-c | --------DSSSAGNQQQESSVSSQSD--ASTSSQLG----- |
| 138 | α-c | --------DSSSASGQQQESSVLPQSGQ-ASTSSQSGA---- |
| 139 | β-c | -------TDSSSASGQQQESSVLSPSGQ-ASTSSQLG----- |
| 140 | α-c | -------ADSSSASGQQQESSVLSPSGQ-ASTSSQSG----- |
| 141 | α-c | ------TDSSSASGQQQESSVLSQSDQ-ASTSSQLG----- |
| 142 | α-c | -------TDSSSASGQQQESSVLSQSDQ-ASTSSQ------- |
| 143 | c-c | -------------------SSQSGADSS-------------- |
| 144 | c-c | ----------------------SSQSGQ-AST---------- |
| 145 | c-c | -----------SASGQQQESSV-------------------- |
| 146 | N/A | -------------------SSQ-------------------- |
| 147 | α - c | --------DSSSASGQQQESGLSSQSE--ASTSSQLG----- |
| 148 | α -α | --------DSSSASDQQQESGLSSQSE--ASTSSQLG----- |
| 149 | α - c | ---------SSSASDQQQGSGVSSQSE--ASTSSQLGT---- |
| 150 | α - c | ---------SSSASGQQQESGVSSQSE--ASTSSQLGT---- |
| 151 | c-c | -------ADSSSASGQQQESGVSSQSE--ASTSSQL------ |
| 152 | c-c | -------ADSLSAGNQQQESDVSSQSE--ASTSSQL------ |
| 153 | α - c | -------ADSSSASGQQQESGVSSQSE--ASTSSQLGADS-- |
| 154 | α-c | DSSSASGQQQESGVSSQSEASTSSQLG |
| 155 | α-α-α | --------DSSSASSQQQESSVLSQSDQ-ASTSSQLG----- |
| 156 | c-c | STSSQLGTDSSSASGQQQESSVSSQSG--Q------------ |
| 157 | c-c | --------DGSSASGQQQESSVLSQSGQ-ASTSSQLG----- |
