# Supplementary material for: Nomenclature, classification, and in-silico structural analysis of MSP1a variants of Anaplasma marginale
Source: PLoS One. 2026 Oct 21;21(10):e0360505. doi: 10.1371/journal.pone.0360505 (PMC13686480; doi:10.1371/journal.pone.0360505)
Supplement: Supplementary file 6 [file pone.0360505.s008.docx]

| **Strains** | **MSP1a tandem repeats 2D** | **Transmission by ticks** | | |
| --- | --- | --- | --- | --- |
|  | | **Dermacentor spp.** | **R. annulatus or R. microplus** | **H. excavatum** |
| Brazil/Maranhao/-(A,B,C) | (c-c, c-c, c-c) | ND | Yes, R. microplus  (Queiroz et al., 2021) | ND |
| Brazil/Maranhao/-(**E,F**,G,H) | (**a-c**, **a-c**, a-c, c-c) | ND | Yes, R. microplus  (Queiroz et al., 2021) | ND |
| Brazil/Maranhao/-(J,I,J²,K) | (a-c, a-c, a-c, a-c, c-c) | ND | Yes, R. microplus  (Queiroz et al., 2021) | ND |
| Brazil/Maranhao/-(L,M,**N**,O) | (c-c, c-c, **c-c**, c-c) | ND | Yes, R. microplus  (Queiroz et al., 2021) | ND |
| Brazil/Maranhao/-(**Q**,P,**Q²**,**R**) | (**a-c**, c-c, **a-c**, **a-c**, **c-c)** | ND | Yes, R. microplus  (Queiroz et al., 2021) | ND |
| Brazil/Maranhao/-(A,S²,**T**,**U**) | (c-c, c-c, c-c, **c-c**, **a-c**) | ND | Yes, R. microplus  (Queiroz et al., 2021) | ND |
| Brazil/Maranhao/-(A,S,C,V) | (c-c, c-c, c-c, c-c) | ND | Yes, R. microplus  (Queiroz et al., 2021) | ND |
| Brazil/Maranhao/-(**Q**,P,Q,**W**) | (**a-c**, c-c, a-c, **c-c**) | ND | Yes, R. microplus  (Queiroz et al., 2021) | ND |
| Brazil/Maranhao/-(J,α,β,Γ) | (a-c, c-c, c-c, a-a) | ND | Yes, R. microplus  (Queiroz et al., 2021) | ND |
| Colombia/-(σ,**μ**,τ) | (α-α, **c-c**, β-c) | ND | yes(R. microplus) [1] | ND |
| Colombia/-(**Φ**,**1,2**) | (**α-c**, **α-c, α-c**) | ND | yes(R. microplus) [1] | ND |
| Colombia/-(3,**4**,5,**6**) | (α-α-α, **α-c**, α-α, **α-c**) | ND | yes(R. microplus) [1] | ND |
| Colombia/-(**7**,8,9) | (**c-c**, c-c, c-c) | ND | yes(R. microplus) [1] | ND |
| Colombia/-(10,**11**,12) | (c-c, **c-c**, c-c) | ND | yes(R. microplus) [1] | ND |
| Colombia/-(**13**,**14**) | (**α-c**, **c-c**) | ND | yes(R. microplus) [1] | ND |
| Colombia/-(**15,16**,17) | (**c-c, c-c**, c-c) | ND | yes(R. microplus) [1] | ND |
| Colombia/-(**18,19**,20) | (**α-c, α-c**, α-c) | ND | yes(R. microplus) [1] | ND |
| Colombia/-(${21}^{2}$,**R**) | (α-c,α-c, **c-c**) | ND | yes(R. microplus) [1] | ND |
| Colombia/-(22,23,**24**,25) | (α-c, α-c, **α-α**, c-c) | ND | yes(R. microplus) [1] | ND |
| Colombia/-(26,27,**28**) | (α-c, α-c, **α-c**) | ND | yes(R. microplus) [1] | ND |
| Colombia/-(**29**,**30**,31) | (**α-α-α**, **α-c**, α-α-α) | ND | yes(R. microplus) [1] | ND |
| Colombia/-(**32**,**33**,**34**) | (**α-c**, **α-c, c-c**) | ND | yes(R. microplus) [1] | ND |
| Colombia/-(**35**,36,37) | **(α-c**, α-c, α-c) | ND | yes(R. microplus) [1] | ND |
| Colombia/-(**18**,38,39) | (**α-c**, c-c, α-c) | ND | yes(R. microplus) [1] | ND |
| Colombia/-(40,21,**Q,R**) | (α-c, α-c, **α-c**, **c-c** ) | ND | yes(R. microplus) [1] | ND |
| Colombia/-(41,42,43,44) | (α-c, α-α, α-α, α-c) | ND | yes(R. microplus) [1] | ND |
| Colombia/-(45,46,38) | (α-c, α-c, c-c) | ND | Yes (R. microplus) [1] | ND |
| Cuba/Western Cuba/-($38^{2}$, **47**) | (c-c, c-c, **c-c)** | Yes | Yes (R. microplus) | ND |
| Cuba/Western Cuba/-(48^3^) | (**c-c, c-c, c-c**) | Yes | Yes (R. microplus) | ND |
| Cuba/Western Cuba/-(48,**15^3^**) | (**c-c**, **c-c, c-c, c-c**) | Yes | Yes (R. microplus) | ND |
| Cuba/Western Cuba/-(**15^3^**, 49, **50)** | _(_**c-c, c-c, c-c_,_** α-c, **α-c)** | Yes | Yes (R. microplus) | ND |
| Cuba/Western Cuba/-(**48,15**,8) | (**c-c**, **c-c**, c-c) | Yes | Yes (R. microplus) | ND |
| Cuba/Western Cuba/-(**48,15,51**) | (**c-c,** **c-c**, **c-c**) | Yes | Yes (R. microplus) | ND |
| Cuba/Western Cuba/-(48, **15^3^**, 10) | (**c-c**, **c-c, c-c, c-c**, c-c) | Yes | Yes (R. microplus) | ND |
| Cuba/Western Cuba/-(A, S, 52, **53)** | (c-c, c-c, α-c**, c-c**) | Yes | Yes (R. microplus) | ND |
| Cuba/Western Cuba/-(48, **15**, 8, 54) | (c-c**, c-c**, c-c, α-c) | Yes | Yes (R. microplus) | ND |
| Ecuador/Zamora-Chinchipe/-(**55^2^**,**56**) | (**α-c, α-c**, **c-c**) | Yes [1] | Yes (Rhipicephalus microplus) [1] | ND |
| Ecuador/Zamora-Chinchipe/-(**99**,141,**57**) | ( **α-c**, α-c, **α-α**) | Yes [1] | Yes (Rhipicephalus microplus) [1] | ND |
| Ecuador/Zamora-Chinchipe/-(**Q^2^**, **57**) | (**α-c, α-c**, **α-α**) | Yes [1] | Yes (Rhipicephalus microplus) [1] | ND |
| Ecuador/Zamora-Chinchipe/-(L, 52, 58) | ( c-c, α-c, c-c) | Yes [1] | Yes (Rhipicephalus microplus) [1] | ND |
| Ecuador/Zamora-Chinchipe/-($Q^{3}$**,** **57)** | ( **α-c**, **α-c**, **α-c**, **α-α**) | Yes [1] | Yes (Rhipicephalus microplus) [1] | ND |
| Ecuador/Zamora-Chinchipe/-($Q^{2}$, **57**) | (**α-c**, **α-c**, **α-α**) | Yes [1] | Yes (Rhipicephalus microplus) [1] | ND |
| Ecuador/Zamora-Chinchipe/-($Q^{2}$, ${59}^{2})$ | ( **α-c**, **α-c**, α-c, **α-c**) | Yes [1] | Yes (Rhipicephalus microplus) [1] | ND |
| Ecuador/Loja/-($Q^{3}$) | ( **α-c**, **α-c**, **α-c** ) | ND | Yes (Rhipicephalus microplus) [2] | ND |
| Ecuador/Loja/-(${60}^{2}$) | ( **α-c, α-c**) | ND | Yes (Rhipicephalus microplus) [2] | ND |
| Ecuador/Loja/-(**61**, **62, 63**, 64) | ( **c-c**, **c-c, c-c**, c-c) | ND | Yes (Rhipicephalus microplus) [2] | ND |
| Ecuador/Loja/-(**65**, **66,** ${60}^{2}$**)** | **( α-c, c-c,** **α-c, α-c**) | ND | Yes (Rhipicephalus microplus) [2] | ND |
| Ecuador/Loja/-( ${67}^{6}$**,** **68**) | ( **α-c, α-c, α-c, α-c, α-c, α-c,** **c-c)** | ND | Yes (Rhipicephalus microplus) [2] | ND |
| Ecuador/Loja/-(**61**, **62, 63**, 62, **64**) | ( **c-c**, **c-c, c-c**, c-c, **c-c**) | ND | Yes (Rhipicephalus microplus) [2] | ND |
| Egypt/El-Wady El-Geded/-  (69,70,**71,72**) | (c-c, c-c, **c-c,** **β-c**) | ND | Yes (Rhipicephalus microplus & Rhipicephalus annulatus) | Yes |
| Egypt/El-Wady El-Geded/-$\left( \boldsymbol{15}^{\boldsymbol{3}},8 \right)$ | (**α-α-α, α-α-α, α-α-α**,α-c) | ND | Yes (Rhipicephalus microplus & Rhipicephalus annulatus) | Yes |
| Egypt/El-Wady El-Geded/-($48$,**15^3^**,10) | (c-c, **α-α-α, α-α-α, α-α-α**, α-c) | ND | Yes (Rhipicephalus microplus & Rhipicephalus annulatus) | Yes |
| Egypt/El-Wady El-Geded/-(48,**15^3^**,10) | (c-c, **α-c, α-c, α-c**, α-c) | ND | Yes (Rhipicephalus microplus & Rhipicephalus annulatus) | Yes |
| Egypt/El-Wady El-Geded/-(48,**15^3^,**10) | (c-c, **α-c, α-c, α-c**, α-c) | ND | Yes (Rhipicephalus microplus & Rhipicephalus annulatus) | Yes |
| Egypt/El-Wady El-Geded/-(48,**15^3^**,10) | (c-c, **α-c, α-c, α-c,** α-c) | ND | Yes (Rhipicephalus microplus & Rhipicephalus annulatus) | Yes |
| Egypt/El-Wady El-Geded/-(10,**15**,8) | (α-c, **α-c**, α-c) | ND | Yes (Rhipicephalus microplus & Rhipicephalus annulatus) | Yes |
| Egypt/-(40,**73**,40,74) | (α-c, **α-α,** α-c, α-c) | ND | Yes (Rhipicephalus microplus & Rhipicephalus annulatus) | Yes |
| Egypt/-(75,**76**,75,77 ) | (α-c**, c-c**, α-c, α-c) | ND | Yes (Rhipicephalus microplus & Rhipicephalus annulatus) | Yes |
| Egypt/-(40,**73**,40,74) | (α-c, **α-α,** α-c, α-c) | ND | Yes (Rhipicephalus microplus & Rhipicephalus annulatus) | Yes |
| Egypt/-$\left( {40}^{3}, 74 \right)$ | (α-c, α-c, α-c, α-c) | ND | Yes (Rhipicephalus microplus & Rhipicephalus annulatus) | Yes |
| Egypt/-$\left( 78, {79}^{2}, 80 \right)$ | (α-c, c-c, c-c, α-α) | ND | Yes (Rhipicephalus microplus & Rhipicephalus annulatus) | Yes |
| Egypt/-(81^2^,82,83,**84**) | (c-c, c-c, c-c, α-c, **c-c)** | ND | Yes (Rhipicephalus microplus & Rhipicephalus annulatus) | Yes |
| Egypt/-(**85**,81,76 ) | (**c-c**, c-c, c-c) | ND | Yes (Rhipicephalus microplus & Rhipicephalus annulatus) | Yes |
| Egypt/ElMinia/-(**84**,86,87^3^,**88** ) | (**c-c**, c-c, c-c, c-c, c-c**, c-c**) | ND | Yes (Rhipicephalus microplus & Rhipicephalus annulatus) | Yes |
| Egypt/ElMinia/-(**85**,89,**90**,85) | (**c-c,** c-c, **α-c**, c-c) | ND | Yes (Rhipicephalus microplus & Rhipicephalus annulatus) | Yes |
| Egypt/ElMinia/-(91,**85**,81,76 ) | (α-c, **c-c**, c-c, c-c) | ND | Yes (Rhipicephalus microplus & Rhipicephalus annulatus) | Yes |
| Mexico/Chiapas/-($A^{3}$, **92**, 93) | (c-c, c-c, c-c, **α-c**, α-c) | Yes | ND | ND |
| Mexico/Chiapas/-(A, S^2^, 52, **53**) | (c-c, c-c, c-c, α-c, **c-c**) | ND | ND | ND |
| Mexico/Hidalgo/-(69, 70, **71**, **94**) | (c-c, c-c, **c-c, α-c)** | ND | ND | ND |
| Mexico/Veracruz/-($A^{2}$, 52, **53**) | (c-c, c-c, α-c, **c-c**) | Yes | Yes (Rhipicephalus microplus) | ND |
| Philippines/Iloilo/-(**Q^2^**, **R**) | (**a-c**, **a-c**, **c-c**) | ND | Yes (Rhipicephalus microplus) (YBAÑEZ, 2014) | ND |
| Philippines/Iloilo/-(95, 54) | (c-c, c-c) | ND | Yes (Rhipicephalus microplus) (YBAÑEZ, 2014) | ND |
| Philippines/Negros Oriental/-(**97**, H) | (**α-α**, c-c) | ND | Yes (Rhipicephalus microplus) (YBAÑEZ, 2014) | ND |
| Philippines/Negros Occidental/-(**98, 97**, H) | (**α-α-α, α-α,** c-c) | ND | Yes (Rhipicephalus microplus) (YBAÑEZ, 2014) | ND |
| Philippines/Negros Occidental/-(**99**, 100) | **(α-c,** α-c) | ND | Yes (Rhipicephalus microplus) (YBAÑEZ, 2014) | ND |
| Philippines/Negros Occidental/-(**98, 97**, H) | (**α-α-α, α-α**, c-c) | ND | Yes (Rhipicephalus microplus) (YBAÑEZ, 2014) | ND |
| Philippines/Negros Occidental/-(**19^2^**,101) | (**α-c, α-c**, N/A) | ND | Yes (Rhipicephalus microplus) (YBAÑEZ, 2014) | ND |
| Philippines/Iloilo/-(102, 10^2^) | (α-c, c-c, c-c) | ND | Yes (Rhipicephalus microplus) (YBAÑEZ, 2014) | ND |
| Philippines/Cavite/-(**104, 105**, 75) | **(α-α, c-c,** α-c) | ND | Yes (Rhipicephalus microplus) (YBAÑEZ, 2014) | ND |
| Philippines/Cavite/-(**Q**, **R**) | **(a-c**, **c-c**) | ND | Yes (Rhipicephalus microplus) (YBAÑEZ, 2014) | ND |
| Philippines/Cavite/-(106, 40^2^) | (α-c, α-c, α-c) | ND | Yes (Rhipicephalus microplus) (YBAÑEZ, 2014) | ND |
| Philippines/Batangas/-(**104**, 10^3^, 75^3^, 107) | (**α-α**, c-c, c-c, c-c, α-c, α-c, α-c, c-c) | ND | Yes (Rhipicephalus microplus) (YBAÑEZ, 2014) | ND |
| Philippines/Batangas/-(108^4^, 109) | (α-c, α-c, α-c, α-c, α-c) | ND | Yes (Rhipicephalus microplus) (YBAÑEZ, 2014) | ND |
| Philippines/Negros Occidental  /-(**Q**, **R**) | (**a-c**, **c-c**) | ND | Yes (Rhipicephalus microplus) (YBAÑEZ, 2014) | ND |
| Philippines/Iloilo/-(110^3^, 19^2^) | (α-c, α-c, α-c, α-c, α-c) | ND | Yes (Rhipicephalus microplus) (YBAÑEZ, 2014) | ND |
| Philippines/Cavite/-(**Q,** **R**) | (**a-c**, **c-c**) | ND | Yes (Rhipicephalus microplus) (YBAÑEZ, 2014) | ND |
| Philippines/Batangas/-(**Q^2^**, **R**) | (**a-c, a-c**, **c-c**) | ND | Yes (Rhipicephalus microplus) (YBAÑEZ, 2014) | ND |
| Philippines/Batangas/-(**111, 112, 113**) | (**c-c, a-c, a-c**) | ND | Yes (Rhipicephalus microplus) (YBAÑEZ, 2014) | ND |
| Philippines/Batangas/-(**114**, S^2^, 52^2^**, 92**, **53**) | (**α-c**, c-c, c-c, c-c, c-c, **c-c**, **α-c-α)** | ND | Yes (Rhipicephalus microplus) (YBAÑEZ, 2014) | ND |
| Russia/Moscow/ -(${152}^{4}$) | **(c - c, c - c, c - c, c - c**) | ND | **ND** | ND |
| Russia/Moscow/ -( ${153}^{3}$) | (**α - c, α - c, α - c**) | ND | **ND** | ND |
| Russia/Moscow/ - (**151,152**) | **(** **c - c, c-c** ) | ND | **ND** | ND |
| Russia/Moscow/ - (**149**, ${150}^{3}$) | **(α - c, c - c, c - c, c - c**) | ND | **ND** | ND |
| Russia/Moscow/ - (**149**, ${150}^{2}$) | (**α - c, α - c, α - c**) | ND | **ND** | ND |
| Russia/Moscow/ - (**149, 150**) | (**α - c, α - c**) | ND | **ND** | ND |
| Russia/Moscow/ - (${150}^{3})$ | **(α - c, α - c, α - c**) | ND | **ND** | ND |
| Russia/Moscow/ - (**148,** ${147}^{5})$ | **(α-α, α - c, α - c, α - c,**  **α - c, α - c**) | **ND** | **ND** | **ND** |
| Russia/Moscow/ - (**148, 147**) | **(α-α, α - c**) | **ND** | **ND** | **ND** |
| Russia/Moscow/ - (${147}^{2}$) | **( α - c, α - c)** | **ND** | **ND** | **ND** |
| Russia/Saratov/- ($154^{5}$) | **(α-c,α-c,α-c,α-c,α-c)** | **ND** | **ND** | **ND** |
| Russia/Saratov/- ($154^{5}$) | **(α-c,α-c,α-c,α-c,α-c)** | **ND** | **ND** | **ND** |
| Russia/Saratov/- ( $154^{5}$) | **(α-c,α-c,α-c,α-c,α-c)** | **ND** | **ND** | **ND** |
| Russia/Saratov/- ($154^{5}$) | **(α-c,α-c,α-c,α-c,α-c)** | **ND** | **ND** | **ND** |
| Russia/Saratov/- ($154^{4}$) | **(α-c,α-c,α-c,α-c)** | **ND** | **ND** | **ND** |
| South Africa/Mpumalanga/-(130, 131, 132, **133, 134)** | (α-α, α-c, c-c, **β-c, α-c**) | Yes | Yes (R. microplus) [1] | No |
| South Africa/Mpumalanga/-(**135, 136**, 132, **133, 136**, 132, **133, 137**) | (**α-c, c-c**, c-c, **β-c**, **c-c**, c-c, **β-c, α-c)** | Yes | Yes (R. microplus) [1] | No |
| South Africa/Mpumalanga/-(**135, 136**, 132, **137**) | (**α-c, c-c**, c-c, **α-c**) | Yes | Yes (R. microplus) [1] | No |
| South Africa/Mpumalanga/-(138, 132, **137**) | (α-c, c-c, **α-c**) | Yes | Yes (R. microplus) [1] | No |
| South Africa/Mpumalanga/-(**135, 136**, 132, **133**, **136**, 132, **133, 137**) | (**α-c, c-c**, c-c, **β-c**, **c-c**, c-c, **β-c, α-c)** | Yes | Yes (R. microplus) [1] | No |
| South Africa/Mpumalanga/-(**135**, **136**, 132, **133**, **137**) | (**α-c, c-c**, c-c, **β-c, α-c**) | Yes | Yes (R. microplus) [1] | No |
| South Africa/Mpumalanga/-(139, 140, **141**, 142) | (α-α, c-c, **α-c**, c-c) | Yes | Yes (R. microplus) [1] | No |
| South Africa/Mpumalanga/-(143, 144, 145, 143, **146**) | (c-c, c-c, α-α, c-c, **α-α-α**) | Yes | Yes (R. microplus) [1] | No |
| Spain/Southern Spain/-($59^{3}$, **115**, **116)** | (**α-c, α-c, α-c**, **α-c**, **c-c**) | ND | ND | ND |
| Thailand/-($69^{2}$, 38) | (c-c, c-c, c-c) | ND | ND | ND |
| Thailand/-($38^{2}$, **Q**,69) | (c-c,c-c, **a-c**, c-c) | ND | ND | ND |
| Thailand/-(155,$38^{2}$,157) | (α-α-α, c-c, c-c, c-c) | ND | ND | ND |
| Thailand/-($69^{2}$) | (c-c, c-c) | ND | ND | ND |
| Thailand/-($Q^{4}$,38) | (**a-c, a-c, a-c, a-c, c-c**) | ND | ND | ND |
| Thailand/-(P,$38^{2}$,**Q**) | (c-c, c-c, c-c,**a-c**) | ND | ND | ND |
| Thailand/-(${156}^{5}$) | (c-c, c-c, c-c, c-c, c-c) | ND | ND | ND |
| Thailand/-(${156}^{5}$) | (c-c, c-c, c-c, c-c, c-c) | ND | ND | ND |
| Thailand/-(${156}^{5}$) | (c-c, c-c, c-c, c-c, c-c) | ND | ND | ND |
| Thailand/-(${156}^{5}$) | (c-c, c-c, c-c, c-c, c-c) | ND | ND | ND |
| Thailand/-(${157}^{5},38$) | (c-c, c-c, c-c, c-c, c-c, c-c) | ND | ND | ND |
| Thailand/-($157,38$) | (c-c, c-c) | ND | ND | ND |
| Thailand/-(${157}^{2},38$) | (c-c, c-c, c-c) | ND | ND | ND |
| Thailand/-(${157}^{3},38$) | (c-c, c-c, c-c, c-c) | ND | ND | ND |
| Thailand/-(${157}^{3},{38}^{2}$) | (c-c, c-c, c-c, c-c, c-c) | ND | ND | ND |
| Thailand/-(${38}^{3}$) | (c-c, c-c, c-c) | ND | ND | ND |
| Turkey/Elazig/-(**12**$0^{4}$, **121**, **84**) | (**α-c, α-c, α-c, α-c**, **α-c,** **c-c**) | Yes [1 | Yes (Rhipicephalus microplus) [1] | ND |
| Turkey/Elazig/-(**122,**123,**124**) | (**c-c**, α-α, **α-c**) | Yes [1] | Yes (*Rhipicephalus microplus*) [1] | ND |
| Turkey/Elazig/-(1${22}^{2}$, **8**$4^{2}$) | (c-c, c-c, **c-c, c-c)** | Yes [1] | Yes (*Rhipicephalus sanguineus*) [1] | ND |
| Turkey/Elazig/-(**125**, $126$) | (**α-α**, α-c) | Yes [1] | Yes (Rhipicephalus microplus) [1] | ND |
| Turkey/Elazig/-(**125**, 126) | (**α-α**, α-c) | Yes [1] | Yes (*Rhipicephalus microplus*) [1] | ND |
| Turkey/Elazig/-(**125**, 126) | (**α-α**, α-c) | Yes [1] | Yes (*Rhipicephalus sanguineus*) [1] | ND |
| Turkey/Elazig/-(**125**, 126) | (**α-α**, α-c) | Yes [1] | Yes (Rhipicephalus microplus) [1] | ND |
| Turkey/Elazig/-(**125**, 126) | (**α-α**, α-c) | Yes [1] | Yes (*Rhipicephalus microplus*) [1] | ND |
| Turkey/Elazig/-(**125**, 126) | (**α-α**, α-c) | Yes [1] | Yes (*Rhipicephalus sanguineus*) [1] | ND |
| Turkey/Elazig/-(**125**, 126) | (**α-α**, α-c) | Yes [1] | Yes (Rhipicephalus microplus) [1] | ND |
| Turkey/Elazig/-(127, 12$8^{2},$129) | (c-c, c-c, c-c, c-c) | Yes [1] | Yes (*Rhipicephalus microplus*) [1] | ND |
| Turkey/Elazig/-(127,12$8^{2}$,129) | (c-c, c-c, c-c, c-c) | Yes [1] | Yes (*Rhipicephalus sanguineus*) [1] | ND |
| Turkey/Elazig/-(127,12$8^{2},$129) | (c-c, c-c, c-c, c-c) | Yes [1] | Yes (Rhipicephalus microplus) [1] | ND |
| Turkey/Elazig/-(**125**,126) | (**α-α**, α-c) | Yes [1] | Yes (*Rhipicephalus microplus*) [1] | ND |
| Turkey/Elazig/-($18$, 4$7^{2}$) | (α-c, α-c, c-c, c-c) | Yes [1] | Yes (*Rhipicephalus sanguineus*) [1] | ND |
| Turkey/Elazig/-($78$, **117,118,119**) | (α-c, **c-c, α-c, α-c**) | Yes [1] | Yes (Rhipicephalus microplus) [1] | ND |
| Turkey/Elazig/-(**8**$4^{3},$87, **88**) | (**c-c, c-c, c-c,** c-c, **c-c**) | Yes [1] | Yes (*Rhipicephalus microplus*) [1] | ND |
| Turkey/Elazig/-(A, $S^{5},58$) | (c-c, c-c, c-c, c-c, c-c, c-c, c-c) | Yes [1] | Yes (*Rhipicephalus sanguineus*) [1] | ND |

1. All the tandem repeats which are written in bold and underlined are the TCE-binding tandem repeats.
2. All the tandem repeats which are highlighted as blue are the tandem repeats with D as the 20^th^ amino acid.
3. The MSP1a strains which are highlighted as orange are the MSP1a strains which do not contain any TCE-binding tandem repeats or a- helix structure but still are transmissible through ticks.

Reference

1. <https://doi.org/10.1590/S1984-29612021097>
2. <https://www.sciencedirect.com/science/article/abs/pii/S1877959X17304405>
3. Cuba: <https://doi.org/10.1016/j.ttbdis.2019.101356>
4. Ecuador: <https://doi.org/10.1016/j.ttbdis.2020.101380>
5. Ecuador: <https://doi.org/10.3390/pathogens9110901>
6. Egypt: <https://www.ncbi.nlm.nih.gov/pmc/articles/PMC7526245/>
7. Mexico: Jimenez-Ocampo, Rafael & Rodríguez, Sergio & Rosario-Cruz, Rodrigo & Vega, Laura & Fuentec, Jose. (2008). Anaplasma marginale: analysis of variable fragment sequences in msp1 alpha and msp4 genes in four new Mexican strains. Técnica Pecuaria en México. 46. 69-78.
8. Mexico: <https://doi.org/10.1079/AHRR200132>
9. Philippines: <https://www.ncbi.nlm.nih.gov/pmc/articles/PMC4143641/>
10. South Africa: <https://www.mdpi.com/2076-2607/11/2/465>
11. Turkey: <https://doi.org/10.1016/j.meegid.2020.104608>
