# Supplementary material for: Nomenclature, classification, and in-silico structural analysis of MSP1a variants of Anaplasma marginale
Source: PLoS One. 2026 Oct 21;21(10):e0360505. doi: 10.1371/journal.pone.0360505 (PMC13686480; doi:10.1371/journal.pone.0360505)
Supplement: Supplementary file 7 [file pone.0360505.s023.docx]

| S.I. | Main Cluster | Subcluster | AA20 |
| --- | --- | --- | --- |
| 1. | α-1 | 1. | Aspartic acid (D) |
| 2. | α-1 | 2. | Glycine (G) |
| 3. | α-1 | 3. | Aspartic acid (D) |
| 4. | α-1 | 4. | --------------------- |
| 5. | α-1 | 5. | Glutamic Acid (E) |
| 6. | α-1 | 6. | Aspartic acid (D) |
| 7. | α-1 | 7. | Glycine (G) |
| 8. | α-1 | 8. | Aspartic acid (D) |
| 9. | α-1 | 9. | Glycine (G) |
| 10. | α-1 | 10. | Serine |
| 11. | α-1 | 11. | Glycine (G) |
| 12. | α-1 | 12. | Aspartic acid (D) |
| 13. | α-1 | 13. | Glutamic Acid (E) |
| 14. | α-1 | 14. | Glycine (G) |
| 15. | α-1 | 15. | Glutamic acid (E) |
| 16. | α-1 | 16. | Aspartic acid (D) |
| 17. | c | 1. | Serine (S) |
| 18. | c | 2. | Glycine (G) |
| 19. | C | 3. | Aspartic acid (D) |
| 20. | C | 4. | Glycine (G) |
| 21. | C | 5. | Glycine (G) |
| 22. | C | 6. | Glycine (G) |
| 23. | α-2 | 1. | Aspartic acid (D) |
| 24. | α-2 | 2. | Aspartic acid (D) |
| 25. | α-2 | 3. | Glycine (G) |
| 26. | b-a-c | 1. | Glycine (G) |

Compute observed statistic

| **Cluster** | **Aspartic Acid (D)** | **Glycine (G)** | **Serine (S)** | **Glutamic acid (E)** |
| --- | --- | --- | --- | --- |
| **α-1** | 6 | 5 | 1 | 3 |
| **α-2** | 2 | 1 | 0 | 0 |
| **c** | 1 | 4 | 1 | 0 |
| **b-a-c** | 0 | 1 | 0 | 0 |
