# Supplementary material for: Nomenclature, classification, and in-silico structural analysis of MSP1a variants of Anaplasma marginale
Source: PLoS One. 2026 Oct 21;21(10):e0360505. doi: 10.1371/journal.pone.0360505 (PMC13686480; doi:10.1371/journal.pone.0360505)
Supplement: Supplementary file 8 [file pone.0360505.s024.docx]

| **Tandem repeats** | **Secondary structure** | **Amino acid at the 20^th^ position** | **Cluster** |
| --- | --- | --- | --- |
| 133 | Alpha-helix | Aspartic acid (D) | **α-1** |
| 136 | Alpha-helix | Aspartic acid (D) |  |
| 137 | Alpha-helix | Aspartic acid (D) |  |
| 53 | Alpha-helix | Aspartic acid (D) |  |
| 92 | Alpha-helix | Serine (S) | **c** |
| 110 | Alpha-helix | Glycine (G) | **α-1** |
| 130 | Alpha-helix | Glycine (G) |  |
| 120 | Alpha-helix | Aspartic acid (D) | **α-1** |
| 88 | Coils | Aspartic acid (D) |  |
| 84 | Coils | Aspartic acid (D) |  |
| 122 | Coils | Aspartic acid (D) |  |
| 108 | Alpha-helix | ------------ | **α-1** |
| 109 | Alpha-helix | ------------ |  |
| 65 | Alpha-helix | Glutamic Acid (E) | **α-1** |
| 59 | Alpha-helix | Glutamic Acid (E) |  |
| 60 | Alpha-helix | Glutamic Acid (E) |  |
| 68 | Coils | Glutamic Acid (E) |  |
| 61 | Coils | Glutamic Acid (E) |  |
| 67 | Alpha-helix | Glutamic Acid (E) |  |
| 47 | Coils | Aspartic acid | **α-1** |
| W | Coils | Aspartic acid |  |
| 11 | Coils | Aspartic acid |  |
| Phi | Alpha-helix | Aspartic acid |  |
| 7 | Coils | Aspartic acid (D) |  |
| 10 | Coils | Glycine (G) | **c** |
| 8 | Coils | Glycine (G) |  |
| 48 | Coils | Aspartic acid (D) | **c** |
| 51 | Coils | Aspartic acid (D) |  |
| 16 | Coils | Aspartic acid (D) |  |
| 15 | Coils | Aspartic acid (D) |  |
| 38 | Coils | Glycine (G) | **c** |
| 54 | Alpha-helix | Glycine (G) |  |
| Sigma | Alpha-helix | Glycine (G) | **α-1** |
| 106 | Alpha-helix | Glycine (G) |  |
| 35 | Alpha-helix | Aspartic acid (D) | **α-1** |
| 125 | Alpha-helix | Aspartic acid (D) |  |
| 45 | Alpha-helix | Aspartic acid (D) | **α-1** |
| 26 | Alpha helix | Glycine (G) |  |
| 102 | Alpha-helix | Glycine (G) |  |
| Alpha | Coils | Glycine (G) | **B-a-c** |
| 22 | Alpha-helix | Glycine (G) |  |
| 69 | Coils | Glycine (G) |  |
| 139 | Beta-strand | Glycine (G) |  |
| 49 | Alpha-helix | Glycine (G) |  |
| 70 | Coils | Glycine (G) |  |
| 40 | Alpha-helix | Glycine (G) |  |
| H | Coils | Glycine (G) |  |
| 75 | Alpha-helix | Glycine (G) |  |
| 78 | Alpha-helix | Glycine (G) |  |
| 107 | Coils | Serine (S) |  |
| 21 | Alpha helix | Serine (S) | **α-1** |
| 132 | Alpha helix | Serine (S) |  |
| 138 | Alpha-helix | Glycine (G) | **α-1** |
| 95 | Coils | Glycine (G) |  |
| P | Coils | Glycine (G) |  |
| E | Alpha-helix | Aspartic acid (D) | **α-1** |
| 135 | Alpha-helix | Aspartic acid (D) |  |
| 57 | Alpha-helix | Aspartic acid (D) | **α-2** |
| 98 | Alpha-helix | Aspartic acid (D) |  |
| 99 | Alpha-helix | Aspartic acid (D) |  |
| Q  4 | Alpha-helix  Alpha-helix | Aspartic acid (D)  Aspartic acid (D) |  |
| R | Coils | Aspartic acid (D) |  |
| 32 | Alpha-helix | Aspartic acid (D) |  |
| 13 | Alpha-helix | Aspartic acid (D) |  |
| 55 | Alpha-helix | Aspartic acid (D) |  |
| 104 | Alpha-helix | Glutamic acid € | **α-1** |
| 105 | Coils | Glutamic acid € |  |
| 86 | Coils | Glycine (G) | **c** |
| 81 | Coils | Glycine (G) |  |
| 18 | Alpha-helix | Aspartic acid (D) | **α-1** |
| 74 | Alpha-helix | Glycine (G) |  |
| I | Alpha-helix | Glycine (G) |  |
| 41 | Alpha-helix | Glycine (G) | **α-1** |
| 117 | Coils | Glutamic acid € |  |
| 91 | Coils | Glutamic acid € |  |
| 89 | Coils | Serine (S) |  |
| 85 | Coils | Aspartic acid (D) | **α-1** |
| 73 | Alpha-helix | Aspartic acid (D) |  |
| 128 | Coils | Glycine (G) | **c** |
| 129 | Coils | Glycine (G) |  |
| S | Coils | Glycine (G) |  |
| 58 | Coils | Glycine (G) |  |
| 126 | Alpha-helix | Glycine (G) |  |
| 52 | Coils | Glycine (G) |  |
| T | Coils | Aspartic acid (D) |  |
| 29 | Alpha-helix | Aspartic acid (D) | **α-2** |
| 111 | Coils | Aspartic acid (D) |  |
| 114 | Alpha-helix | Aspartic acid (D) |  |
| L | Coils | Glycine (G) | **α-2** |
| V | Coils | Glycine (G) |  |
| A | Coils | Glycine (G) |  |
| 3 | Alpha-helix | Serine (S) |  |
| 127 | Coils | Glycine (G) |  |
