# Supplementary material for: Nomenclature, classification, and in-silico structural analysis of MSP1a variants of Anaplasma marginale
Source: PLoS One. 2026 Oct 21;21(10):e0360505. doi: 10.1371/journal.pone.0360505 (PMC13686480; doi:10.1371/journal.pone.0360505)
Supplement: Supplementary file 9 [file pone.0360505.s025.docx]

| **Name of tandem repeats** | **Tandem Repeats Sequences** | **Position of B-cell Epitopes** | **B cell epitopes** | **BCPRED Scores** | **Antigenic Scores** | **Location** |
| --- | --- | --- | --- | --- | --- | --- |
| A | SGVLSQSGQASTSSQLGTDSSSAGDQQQ | 11 | STSSQLGTDSSS | 0.467 | 1.1090 | outside |
| B | SGVSSQSGQASTSSQLGADSSSAGGQQQ | 11 | STSSQLGADSSS | 0.715 | 1.0510 | outside |
| C | SSVLSPSGQASTSSQLGADSSSASGQQQ | 11 | STSSQLGADSSS | 0.715 | 1.0510 | outside |
| D | SGVLSQSGQAST | N/A | N/A | N/A | N/A | N/A |
| E | DSSSASGQQQESSVLPQSDQASTSSQLG | 3 | SSASGQQQESSV | 0.946 | 1.2768 | inside |
|  |  | 16 | PQSDQASTSSQL | 0.565 | 0.9305 | outside |
| F | DSSPASGQQQESSVLPQSDQASTSSQLG | 3 | SPASGQQQESSV | 0.986 | 0.8510 | outside |
|  |  | 16 | PQSDQASTSSQL | 0.565 | 0.9305 | outside |
| G | GSSSASGQQQESSVLPQSGQASTSSQLG | 3 | SSASGQQQESSV | 0.946 | 1.2768 | inside |
|  |  | 16 | PQSGQASTSSQL | 0.374 | 0.8794 | outside |
| H | DSSSASGQQQESSVLSQSGASTSSQL | 3 | SSASGQQQESSV | 0.946 | 1.2768 | inside |
| I | DSSSASGQQQESGVLSQSGQASTSSQLG | 3 | SSASGQQQESGV | 0.756 | 1.2743 | inside |
| J | DSSSASGQQQESSVLSPSGQASTSSQLG | 3 | SSASGQQQESSV | 0.946 | 1.2768 | inside |
|  |  | 16 | SPSGQASTSSQL | 0.584 | 1.1794 | outside |
| K | DSSSASGQQQESGVLSQSGQASTSSQL | 3 | SSASGQQQESGV | 0.756 | 1.2743 | inside |
| L | SGVLSQSGQASTSSQLGTDSSSASGQQQ | 11 | STSSQLGTDSSS | 0.467 | 1.1090 | outside |
| M | SSVLPQSGQASTSSQLGAGSSSASGQQQ | 5 | PQSGQASTSSQL | 0.374 | 0.8794 | outside |
| N | SSVLSQSDQASTSSQLGTDSSSASGQQQ | 5 | SQSDQASTSSQL | 0.501 | 0.9748 | outside |
| O | SSVLSQSGASTSSQ | N/A | N/A | N/A | N/A | N/A |
| P | DSSSASGQQQESSVLPQSGQASTSSQLG | 3 | SSASGQQQESSV | 0.946 | 1.2768 | inside |
|  |  | 16 | PQSGQASTSSQL | 0.374 | 0.8794 | outside |
| Q | DSSSASGQQQESSVLSQSDQASTSSQLG | 3 | SSASGQQQESSV | 0.946 | 1.2768 | inside |
|  |  | 16 | SQSDQASTSSQL | 0.501 | 0.9748 | outside |
| R | DSSSASGQQQESSVLSQSDQASTSSQL | 3 | SSASGQQQESSV | 0.946 | 1.2768 | inside |
|  |  | 16 | SQSDQASTSSQL | 0.501 | 0.9748 | outside |
| S | SGVSSQSGQASTSSQLGTDSSSAGDQQQ | 11 | STSSQLGTDSSS | 0.467 | 1.1090 | outside |
| T | SGVSSQSDQASTSSQLGTDSSSASGQQQ | 2 | GVSSQSDQASTS | 0.881 | 1.3194 | outside |
| U | SSVLSQSDQASTSSQSGTD | N/A | N/A | N/A | N/A | N/A |
| V | SGVLSQSGQASTSSQLGTD | 7 | SGQASTSSQLGT | 0.327 | 1.1992 | outside |
| W | DSSSASGQQQESSVSSQSDQASTSSQL | 4 | SASGQQQESSVS | 0.965 | 1.2575 | inside |
| α | DNSSASGQQPESSVLSPSTSSQLGEWSG | 16 | SPSTSSQLGEWS | 0.978 | 0.6015 | outside |
|  |  | 3 | SSASGQQPESSV | 0.746 | 1.3888 | outside |
| β | DSSSASGQQPESSVLSPSAQASTSSQLG | 5 | ASGQQPESSVLS | 0.856 | 1.3537 | outside |
| Γ | TDNSSASGQQQESSVSSQSAQASTSSQLG | 5 | SASGQQQESSVS | 0.965 | 1.2575 | inside |
|  |  | 18 | QSAQASTSSQLG | 0.339 | 0.9825 | inside |
| π | STSSQLGTGWRQEMRSKVASDE | 1 | STSSQLGTGWRQ | 0.967 | 0.0582 | outside |
| Σ | STSSQLGADLRQEVHSKVASVE | 9 | DLRQEVHSKVAS | 0.418 | 1.6003 | inside |
| σ | DSSSASDQQQESSVSSQSGQASTSSQLGA | 4 | SASDQQQESSVS | 0.914 | 1.1573 | inside |
| μ | DSSSASGQQQESSVSSQSDEVSTSSQLG | 4 | SASGQQQESSVS | 0.965 | 1.2575 | inside |
|  |  | 17 | QSDEVSTSSQLG | 0.749 | 0.9446 | outside |
| τ | ITRQSGGQQQESSVSSQSGQASTSSQSGA | 4 | QSGGQQQESSVS | 0.974 | 1.1283 | inside |
| Φ | SASGQQQESSVSSQSDQASSTSSQLGADSS | 1 | SASGQQQESSVS | 0.965 | 1.2575 | inside |
|  |  | 19 | SSTSSQLGADSS | 0.292 | 1.0580 | outside |
| 1 | SASGQQQESSVLSQSDQASSTSSQLGADSS | 2 | ASGQQQESSVLS | 0.909 | 1.1787 | inside |
|  |  | 19 | SSTSSQLGADSS | 0.292 | 1.0580 | outside |
| 2 | SESGQQQESSVSSQSDQASTSSQSGAD | 1 | SESGQQQESSVS | 0.999 | 1.2790 | outside |
| 3 | SSSASGQQQSESSVLSQSGQASTSSQLGAD | 3 | SASGQQQSESSV | 0.855 | 1.2136 | inside |
| 4 | TDSSSSASGQQQESSVLSQSDQASSTSSQL | 5 | SSASGQQQESSV | 0.946 | 1.2768 | inside |
| 5 | SSSASGQQQESGVLSQSDQASTSSQLGAD | 2 | SSASGQQQESGV | 0.756 | 1.2743 | inside |
|  |  | 15 | SQSDQASTSSQL | 0.501 | 0.9748 | outside |
| 6 | TDSSSSASGQQQESSVLSQSDQASSTSSQL | 5 | SSASGQQQESSV | 0.946 | 1.2768 | inside |
| 7 | ADISSASGQQQESSVSSQSDQASTSSQL | 5 | SASGQQQESSVS | 0.965 | 1.2575 | inside |
| 8 | ADSSSAGGQQQESSVSSQSGQASTSSQL | 5 | SAGGQQQESSVS | 0.894 | 1.1696 | outside |
| 9 | TDSSSASGQQQESSVSSQSGQASTSSQS | 5 | SASGQQQESSVS | 0.965 | 1.2575 | inside |
| 10 | ADSSSASGQQQESSVSSQSGQASTSSQL | 5 | SASGQQQESSVS | 0.965 | 1.2575 | inside |
| 11 | ADSSSASGQQQESSVSSQSDQASTSSQL | 5 | SASGQQQESSVS | 0.965 | 1.2575 | inside |
| 12 | TDTSSASGQQQESSVSSQSGQASTSSQS | 5 | SASGQQQESSVS | 0.965 | 1.2575 | inside |
| 13 | TDSSSSASGQQQESSVLSQSDQASSTSSQL | 5 | SSASGQQQESSV | 0.946 | 1.2768 | inside |
| 14 | ADSSSASGQQQESSVLSQSDQASTSSQL | 4 | SSASGQQQESSV | 0.946 | 1.2768 | inside |
|  |  | 17 | SQSDQASTSSQL | 0.501 | 0.9748 | outside |
| 15 | ADSSSAGGQQQESSVSSQSDQASTSSQL | 5 | SAGGQQQESSVS | 0.894 | 1.1696 | outside |
| 16 | ADSSSAGGQHQESSVSSQSDQASTSSQL | 16 | SSQSDQASTSSQ | 0.724 | 1.4025 | outside |
| 17 | PDTSSAGGHHHKSSVSSQSGQASTSSQLG | 5 | SAGGHHHKSSVS | 0.611 | 1.4055 | outside |
| 18 | DSSSASGQQQESGVLSQSGQASTSSQLG | 3 | SSASGQQQESGV | 0.756 | 1.2743 | inside |
| 19 | DSSSAGDQQQESSVSSQSDASTSSQLG | 4 | SAGDQQQESSVS | 0.928 | 1.1617 | outside |
| 20 | DSSSAGDQQQESSVSSQSGHASTSSQLG | 4 | SAGDQQQESSVS | 0.928 | 1.1617 | outside |
|  |  | 17 | QSGHASTSSQLG | 0.682 | 1.0947 | outside |
| 21 | DSSSASGQQQESSVLSQSSQASTSSQLG | 3 | SSASGQQQESSV | 0.946 | 1.2768 | inside |
| 22 | DNSSARGHQQENSVVSPSGQASTSSQLG | 7 | GHQQENSVVSPS | 0.608 | 1.3097 | outside |
| 23 | DSSSASDQQQESSVLSQSNQASTSSQLG | 3 | SSASDQQQESSV | 0.904 | 1.3886 | inside |
| 24 | DSSSASDQQQESSVLSQSDQASTSSQLG | 3 | SSASDQQQESSV | 0.904 | 1.3886 | inside |
|  |  | 16 | SQSDQASTSSQL | 0.501 | 0.9748 | outside |
| 25 | YCSSAGGQQQESSVSSQSGQASTSSQL | 4 | SAGGQQQESSVS | 0.894 | 1.1696 | outside |
| 26 | DSSSAGDQQQESSVLSQSGQASSTSSQLG | 3 | SSAGDQQQESSV | 0.887 | 1.4895 | outside |
| 27 | DSSSASDQQQESSVLSQSNQASTSSLLG | 3 | SSASDQQQESSV | 0.904 | 1.3886 | inside |
| 28 | NSSSASDQQQDSIVLSQSDQASTSSQL | 16 | SQSDQASTSSQL | 0.501 | 0.9748 | outside |
|  |  | 3 | SSASDQQQDSIV | 0.364 | 1.2631 | inside |
| 29 | ARQLGGQQQESSVLSQSDQASSTSSQLGAD | 2 | RQLGGQQQESSV | 0.853 | 1.0031 | inside |
| 30 | TDSSSSASGQQQESSVLSQSDQASSTSSQL | 5 | SSASGQQQESSV | 0.946 | 1.2768 | inside |
| 31 | SSASGQQESSVLSQSDQASTSSQSGTD | 3 | ASGQQESSVLSQ | 0.756 | 1.1203 | outside |
| 32 | SASGQQQESSVLSQSDQASSTSSQLGADSS | 2 | ASGQQQESSVLS | 0.909 | 1.1787 | inside |
|  |  | 19 | SSTSSQLGADSS | 0.292 | 1.0580 | outside |
| 33 | SASGQQQESSVLSQSDQASTSSQSGTDSS | 2 | ASGQQQESSVLS | 0.909 | 1.1787 | inside |
| 34 | SASGQQQESSVLSQSDQASTSSQLGA | 2 | ASGQQQESSVLS | 0.909 | 1.1787 | inside |
| 35 | TDSSSAGDQQQESSVLSQSDQASTSSQLG | 4 | SSAGDQQQESSV | 0.887 | 1.4895 | outside |
|  |  | 17 | SQSDQASTSSQL | 0.501 | 0.9748 | outside |
| 36 | ADSSSASGQQQESSVLSQSAQASTSSQLG | 4 | SSASGQQQESSV | 0.946 | 1.2768 | inside |
|  |  | 18 | QSAQASTSSQLG | 0.339 | 0.9825 | inside |
| 37 | DNSSASDQQQESSVSSQSGRASTSSQLG | 4 | SASDQQQESSVS | 0.914 | 1.1573 | inside |
| 38 | DSSSASGQQQESSVSSQSGQASTSSQLG | 4 | SASGQQQESSVS | 0.965 | 1.2575 | inside |
| 39 | DSSSASGQQQESSVLSQSGQASTSSQSG | 3 | SSASGQQQESSV | 0.946 | 1.2768 | inside |
| 40 | DSSSASGQQQESSVLSQSGQASTSSQLG | 3 | SSASGQQQESSV | 0.946 | 1.2768 | inside |
| 41 | SSSASGQQQGSGVSYQSGQASTSINLETD | 5 | SGQQQGSGVSYQ | 0.654 | 0.9623 | outside |
| 42 | SSSAGGQQQESSVLSQSGQASTSSQLGAD | 5 | GGQQQESSVLSQ | 0.728 | 1.0972 | outside |
| 43 | SSSAGDQQQESSVSSQSGQASTSSQLGTD | 3 | SAGDQQQESSVS | 0.928 | 1.1617 | outside |
|  |  | 17 | SGQASTSSQLGT | 0.327 | 1.1992 | outside |
| 44 | SSSASGQQQESSVLSQSGQASTSSQL | 2 | SSASGQQQESSV | 0.946 | 1.2768 | inside |
| 45 | DSSSAGDQQQESSVLSQSGQASSTSSQLG | 3 | SSAGDQQQESSV | 0.887 | 1.4895 | outside |
| 46 | DSSSASDQQQERGVLSQSGQASTSSQLG | 3 | SSASDQQQERGV | 0.923 | 1.1040 | inside |
|  |  |  |  |  |  |  |
| 47 | DSSSASGQQQESSVSSQSDQASTSSQLG | 4 | SASGQQQESSVS | 0.965 | 1.2575 | inside |
|  |  | 17 | QSDQASTSSQLG | 0.432 | 1.0110 | inside |
| 48 | AGSSSAGGQQQESSVSSQSDQASTSSQL | 5 | SAGGQQQESSVS | 0.894 | 1.1696 | outside |
| 49 | ADSSSASGQQQESSVLSPSGQASTSSQLGV | 4 | SSASGQQQESSV | 0.946 | 1.2768 | inside |
|  |  | 17 | SPSGQASTSSQL | 0.584 | 1.1794 | outside |
| 50 | ADSSSASGQQQESSVSSQSDASTSSQ | 5 | SASGQQQESSVS | 0.965 | 1.2575 | inside |
| 51 | ADSSSAGGQQQESSVSSQSDQASTSSQS | 5 | SAGGQQQESSVS | 0.894 | 1.1696 | outside |
| 52 | SGVSSQSGQASTSSQLGTDSSSASGQQQ | 11 | STSSQLGTDSSS | 0.467 | 1.1090 | outside |
| 53 | SSVSSQSDASTSSQLGAD | 3 | VSSQSDASTSSQ | 0.569 | 1.4745 | outside |
| 54 | TDSSSASGQQQESSVSSQSGQASTSSQL | 5 | SASGQQQESSVS | 0.965 | 1.2575 | inside |
| 55 | SGQQQESSVLSQSDQASTSSQLGTDSSS | 1 | SGQQQESSVLSQ | 0.871 | 1.1364 | outside |
|  |  | 17 | STSSQLGTDSSS | 0.467 | 1.1090 | outside |
| 56 | SGQQQESSVLSQSDQASTSSQSGTD | 1 | SGQQQESSVLSQ | 0.871 | 1.1364 | outside |
| 57 | DSSSASGQQQESSVLSQSDQASTSSQSG | 3 | SSASGQQQESSV | 0.946 | 1.2768 | inside |
| 58 | SGVSSQSGQASTSSQLGTD | 4 | SSQSGQASTSSQ | 0.441 | 1.4877 | outside |
| 59 | DSSSASGQQQESSVSSQSEASTSSQLG | 4 | SASGQQQESSVS | 0.965 | 1.2575 | inside |
| 60 | SSSASGQQQESSVSSQSEASTSSQLGT | 3 | SASGQQQESSVS | 0.965 | 1.2575 | inside |
| 61 | AGSSSAGGQQQESSVSSQSEASTSSQLG | 5 | SAGGQQQESSVS | 0.894 | 1.1696 | outside |
| 62 | ADSSSASGQQQESSVLSQSEASTSSQLG | 4 | SSASGQQQESSV | 0.946 | 1.2768 | inside |
| 63 | ADSSSASGQQQESSVSSQSEASTSSQLG | 5 | SASGQQQESSVS | 0.965 | 1.2575 | inside |
| 64 | ADSSSASGQQQESSVSSQSEASTSSQL | 5 | SASGQQQESSVS | 0.965 | 1.2575 | inside |
| 65 | SSSASGQQQESSVSSQSEASTSSQLGA | 3 | SASGQQQESSVS | 0.965 | 1.2575 | inside |
| 66 | SSSASGQQQESSVSSQSDQASTSSQLGT | 3 | SASGQQQESSVS | 0.965 | 1.2575 | inside |
|  |  | 16 | QSDQASTSSQLG | 0.432 | 1.0110 | inside |
| 67 | SSSASGQQQESSVSSQSEASTSSQLGGA | 3 | SASGQQQESSVS | 0.965 | 1.2575 | inside |
| 68 | SSSASGQQQESSVSSQSEASTSSQL | 3 | SASGQQQESSVS | 0.965 | 1.2575 | inside |
| 69 | TDSSSASGQQQESSVLSPSGQASTSSQL | 4 | SSASGQQQESSV | 0.946 | 1.2768 | inside |
|  |  | 17 | SPSGQASTSSQL | 0.584 | 1.1794 | outside |
| 70 | ADSSSASGQQQESSVLSPSGQ-ASTSSQS | 4 | SSASGQQQESSV | 0.946 | 1.2768 | inside |
| 71 | TDSSSASGQQQESSVLSQSDQ-ASTSSQL | 4 | SSASGQQQESSV | 0.946 | 1.2768 | inside |
|  |  | 17 | SQSDQASTSSQL | 0.501 | 0.9748 | outside |
| 72 | TDSSSTSGQQQESSVLSQSDQ-ASTSSQS | 8 | QQQESGVSSQSD | 0.909 | 1.6348 | inside |
| 73 | DSSSASGQQQESGVSSQSDQASTSSQLG | 8 | QQQESGVSSQSD | 0.909 | 1.6348 | inside |
| 74 | DSSSASGQQQESGVLSQSGQASTSSQSG | 4 | SAGNQQQESSVS | 0.989 | 1.0628 | inside |
| 75 | ADSSSASGQQQESGVSSQSDQASTSSQL | 3 | SSASGQQQESGV | 0.756 | 1.2743 | inside |
| 76 | TDSSSASGQQQESGVSSQSDQASTSSQL | 9 | QQQESGVSSQSD | 0.909 | 1.6348 | inside |
| 77 | ADSSSASGQQQESGVLSQSGQASTSSQL | 4 | SSASGQQQESGV | 0.756 | 1.2743 | inside |
| 78 | SSSASGQQQESSVLSQGGQASTSSQLGT | 2 | SSASGQQQESSV | 0.946 | 1.2768 | inside |
|  |  | 15 | SQGGQASTSSQL | 0.543 | 1.2885 | outside |
| 79 | SSSASGQQQESSVSSQSGQASTSSQLGT | 3 | SASGQQQESSVS | 0.965 | 1.2575 | inside |
|  |  | 17 | SGQASTSSQLGT | 0.327 | 1.1992 | outside |
| 80 | SSSASGQQQESSVSSQSGQASTSSQLGA | 3 | SASGQQQESSVS | 0.965 | 1.2575 | inside |
| 81 | ADSSSASGQQQESGVPSQSGQASTSSQL | 7 | SGQQQESGVPSQ | 0.852 | 1.3488 | outside |
| 82 | TDSSSASGQQQESGVPSQSGQASTSSQL | 7 | SGQQQESGVPSQ | 0.852 | 1.3488 | outside |
| 83 | TDSSSASGQQQESGVPSQSGQASTSSQLGS | 7 | SGQQQESGVPSQ | 0.852 | 1.3488 | outside |
| 84 | ADSSSASGQQQESGVSSQSDASTSSQL | 9 | QQQESGVSSQSD | 0.909 | 1.6348 | inside |
| 85 | ADSSSASGQQQESGVSSQSDQASTSSQL | 9 | QQQESGVSSQSD | 0.909 | 1.6348 | inside |
| 86 | ADSSSASGQQQESSVPSQSGASTSSQL | 7 | SGQQQESSVPSQ | 0.983 | 1.1848 | outside |
| 87 | ADSSSASGQQQESGVPSQSGASTSSQL | 13 | SGVPSQSGASTS | 0.867 | 1.0209 | outside |
| 88 | TDSSSASGQQQESGVSSQSDASTSSQL | 9 | QQQESGVSSQSD | 0.909 | 1.6348 | inside |
| 89 | ADSSSASGQQQESGVSSQSSHASTSSQL | 7 | SGQQQESGVSSQ | 0.906 | 1.4769 | outside |
| 90 | ADSSSASGQQQESGVPSQSDQASTSSQL | 9 | QQQESGVPSQSD | 0.892 | 1.1920 | outside |
| 91 | AGSSSASGQQQESGVSSQSEQASTSSQL | 10 | QQESGVSSQSEQ | 0.993 | 1.3242 | inside |
| 92 | SSVSSQSDASTSSQLGTDSSSAGDQQQ | 3 | VSSQSDASTSSQ | 0.569 | 1.4745 | outside |
|  |  | 16 | GTDSSSAGDQQQ | 0.371 | 1.7299 | outside |
| 93 | SGVSSQSGQASTSSQLGT | 4 | SSQSGQASTSSQ | 0.441 | 1.4877 | outside |
| 94 | TDSSSASGQQQESSVLSQSDQASTSSQS | 4 | SSASGQQQESSV | 0.946 | 1.2768 | inside |
| 95 | TDSSSASGQQQESSVLPQSGQASTSSQL | 4 | SSASGQQQESSV | 0.946 | 1.2768 | inside |
|  |  | 17 | PQSGQASTSSQL | 0.374 | 0.8794 | outside |
| 96 | GTD |  |  |  |  |  |
| 97 | DSSSASGQQQESSVLSQSDQASTLSQLG | 3 | SSASGQQQESSV | 0.946 | 1.2768 | inside |
|  |  | 16 | SQSDQASTLSQL | 0.531 | 0.3748 | outside |
| 98 | DSSSASGQRQESSVLSQSDQASTLSQLG | 3 | SSASGQRQESSV | 0.889 | 1.0568 | inside |
|  |  | 16 | SQSDQASTLSQL | 0.531 | 0.3748 | outside |
| 99 | TDSSSASGQQQESSVLSQSDQASTSSQLG | 4 | SSASGQQQESSV | 0.946 | 1.2768 | inside |
|  |  |  |  |  |  |  |
|  |  |  |  |  |  |  |
|  |  | 17 | SQSDQASTSSQL | 0.501 | 0.9748 | outside |
|  |  |  |  |  |  |  |
|  |  |  |  |  |  |  |
| 100 | TDSSSASGQQQESSVLSQSGASTSSQLGT | 4 | SSASGQQQESSV | 0.946 | 1.2768 | inside |
|  |  | 18 | QSGASTSSQLGT | 0.531 | 1.1174 | outside |
| 101 | TD |  |  |  |  |  |
| 102 | ADSSSAGDQQQESSVLSQSGQASTSSQL | 4 | SSAGDQQQESSV | 0.887 | 1.4895 | outside |
| 103 | GAD |  |  |  |  |  |
| 104 | ADSSSASDQQQESGVPSQSEASTSSQL | 9 | QQQESGVPSQSE | 0.897 | 1.1560 | inside |
| 105 | ADSSSASGQQQESGVPSQSEASTSSQL | 9 | QQQESGVPSQSE | 0.897 | 1.1560 | inside |
| 106 | DSSSASDQQQESSVLSQSGQASTSSQLG | 3 | SSASDQQQESSV | 0.904 | 1.3886 | inside |
| 107 | TDSSSASGQQQESSVLSQSGQASTSSQL | 4 | SSASGQQQESSV | 0.946 | 1.2768 | inside |
| 108 | ADSSSASGQQQESSVSSQL | 5 | SASGQQQESSVS | 0.965 | 1.2575 | insinde |
| 109 | ADSSSASGQQQESSVSSQ | 5 | SASGQQQESSVS | 0.965 | 1.2575 | insinde |
| 110 | DSSSAGDQQQESSVSSQSGASTSSQLG | 4 | SAGDQQQESSVS | 0.928 | 1.1617 | outside |
| 111 | SGVLSKSDQASTSSQLGADSSSASGQQQ | 11 | STSSQLGADSSS | 0.715 | 1.0510 | outside |
| 112 | SSVLSQSDQASTSSQLGADSSSASGQQQ | 11 | STSSQLGADSSS | 0.715 | 1.0510 | outside |
| 113 | SSVLSQSDQASTSSQL | 5 | SQSDQASTSSQL | 0.501 | 0.9748 | outside |
| 114 | SGVLSKSDQASTSSQLGTDSSSAGDQQQ | 11 | STSSQLGTDSSS | 0.467 | 1.1090 | outside |
| 115 | DSSSASGQQQESSVSSQSEASTSSHLG | 4 | SASGQQQESSVS | 0.965 | 1.2575 | inside |
| 116 | DSSSASGQHQESTVSSQSDASTSAQLD | 11 | ESTVSSQSDAST | 0.902 | 1.4949 | outside |
| 117 | SSSASGQQQGGGVSPQSEASTSSQLGT | 5 | SGQQQGGGVSPQ | 1 | 1.6891 | outside |
| 118 | SSSASGQQQESGVSPQSEASTSSQLGT | 5 | SGQQQESGVSPQ | 0.898 | 1.1532 | inside |
| 119 | SSSASGQQQESGVSPQSEASTSSQL | 5 | SGQQQESGVSPQ | 0.898 | 1.1532 | inside |
| 120 | DSSSASGQQQESSVSSQSDASTSSQLG | 4 | SASGQQQESSVS | 0.965 | 1.2575 | inside |
| 121 | DSSSASGQQQESGVSSQSDASTSSQL | 8 | QQQESGVSSQSD | 0.909 | 1.6348 | inside |
| 122 | AGSSSASGQQQESGVSSQSDASTSSQL | 9 | QQQESGVSSQSD | 0.909 | 1.6348 | inside |
| 123 | SSSASGQQQESGVPSQSGQASTSSQLGA | 5 | SGQQQESGVPSQ | 0.852 | 1.3488 | outside |
| 124 | SSSASGQQQESGVSSQSDASTSSQLGA | 7 | QQQESGVSSQSD | 0.909 | 1.6348 | inside |
| 125 | DSSSAGDQQQESSVLSQSDQASTSSQLG | 3 | SSAGDQQQESSV | 0.887 | 1.4895 | outside |
|  |  | 16 | SQSDQASTSSQL | 0.501 | 0.9748 | inside |
| 126 | DSSSAGDQQQESGVSSQSGQASTSSQLG | 7 | DQQQESGVSSQS | 0.833 | 1.6185 | inside |
| 127 | GVLSQSGQASTSSQLGTDSSSAGDQQQE | 17 | TDSSSAGDQQQE | 0.648 | 1.7397 | outside |
| 128 | GVSSQSGQASTSSQLGTDSSSAGDQQQE | 17 | TDSSSAGDQQQE | 0.648 | 1.7397 | outside |
|  |  | 3 | SSQSGQASTSSQ | 0.441 | 1.4877 | inside |
| 129 | GVSSQSGQASTSSQLGTDSSSAGDQQQE | 3 | SSQSGQASTSSQ | 0.441 | 1.4877 | inside |
| 130 | DSSSAGNQQQESSVSSQSGASTSSQLGT | 4 | SAGNQQQESSVS | 0.989 | 1.0628 | inside |
|  |  | 17 | QSGASTSSQLGT | 0.531 | 1.1174 | outside |
| 131 | DSSSAGDQQQESSVLSPSGQASTSSQSGT | 3 | SSAGDQQQESSV | 0.887 | 1.4895 | outside |
| 132 | DSSSASGQQQESSVLSQSSQASTSSQLGA | 3 | SSASGQQQESSV | 0.946 | 1.2768 | inside |
| 133 | DSSSAGNQQQESSVSSQSDASTSSQLGA | 4 | SAGNQQQESSVS | 0.989 | 1.0628 | inside |
| 134 | DSSSAGNQQQESSVSSQSDASTSSQLGT | 4 | SAGNQQQESSVS | 0.989 | 1.0628 | inside |
|  |  | 17 | QSDASTSSQLGT | 0.419 | 1.1514 | outside |
| 135 | DSSSASGQQQESSVLPQSDASTSSQLGA | 3 | SSASGQQQESSV | 0.946 | 1.2768 | inside |
|  |  | 16 | PQSDASTSSQLG | 0.702 | 1.0478 | outside |
| 136 | DSSSAGNQQQESSVSSQSDASTSSQSGA | 4 | SAGNQQQESSVS | 0.989 | 1.0628 | inside |
| 137 | DSSSAGNQQQESSVSSQSDASTSSQLG | 4 | SAGNQQQESSVS | 0.989 | 1.0628 | inside |
| 138 | DSSSASGQQQESSVLPQSGQASTSSQSGA | 3 | SSASGQQQESSV | 0.946 | 1.2768 | inside |
| 139 | TDSSSASGQQQESSVLSPSGQASTSSQLG | 4 | SSASGQQQESSV | 0.946 | 1.2768 | inside |
|  |  | 17 | SPSGQASTSSQL | 0.584 | 1.1794 | outside |
| 140 | ADSSSASGQQQESSVLSPSGQASTSSQSG | 4 | SSASGQQQESSV | 0.946 | 1.2768 | inside |
| 141 | TDSSSASGQQQESSVLSQSDQASTSSQLG | 4 | SSASGQQQESSV | 0.946 | 1.2768 | inside |
|  |  | 17 | SQSDQASTSSQL | 0.501 |  | outside |
| 142 | TDSSSASGQQQESSVLSQSDQASTSSQ | 4 | SSASGQQQESSV | 0.946 | 1.2768 | inside |
| 143 | SSQSGADSS | No Epitopes Found as Sequence is Short | | | N/A | |
| 144 | SSQSGQAST |  |  |  |  |  |
| 145 | SASGQQQESSV |  |  |  |  |  |
| 146 | SSQ |  |  |  |  |  |
| 147 | DSSSASGQQQESGLSSQSEASTSSQLG | 3 | SSASGQQQESGL | 0.604 | 1.1579 | outside |
| 148 | DSSSASDQQQESGLSSQSEASTSSQLG | 3 | SSASDQQQESGL | 0.67 | 1.2698 | outside |
| 149 | SSSASDQQQGSGVSSQSEASTSSQLGT | 7 | QQQGSGVSSQSE | 0.908 | 1.7441 | inside |
| 150 | SSSASGQQQESGVSSQSEASTSSQLGT | 7 | QQQESGVSSQSE | 0.957 | 1.4868 | inside |
| 151 | ADSSSASGQQQESGVSSQSEASTSSQL | 9 | QQQESGVSSQSE | 0.957 | 1.4868 | inside |
| 152 | ADSLSAGNQQQESDVSSQSEASTSSQL | 9 | QQQESDVSSQSE | 0.97 | 1.2944 | inside |
| 153 | ADSSSASGQQQESGVSSQSEASTSSQLGADS | 9 | QQQESGVSSQSE | 0.957 | 1.4868 | inside |
| 154 | DSSSASGQQQESGVSSQSEASTSSQLG | 8 | QQQESGVSSQSE | 0.957 | 1.4868 | inside |
| 155 | DSSSASSQQQESSVLSQSDQASTSSQLG | 6 | SSQQQESSVLSQ | 0.967 | 1.0919 | inside |
| 156 | STSSQLGTDSSSASGQQQESSVSSQSGQ | 12 | SASGQQQESSVS | 0.965 | 1.2575 | inside |
| 157 | DGSSASGQQQESSVLSQSGQASTSSQLG | 3 | SSASGQQQESSV | 0.946 | 1.2768 | inside |
