# Supplementary material for: Nomenclature, classification, and in-silico structural analysis of MSP1a variants of Anaplasma marginale
Source: PLoS One. 2026 Oct 21;21(10):e0360505. doi: 10.1371/journal.pone.0360505 (PMC13686480; doi:10.1371/journal.pone.0360505)
Supplement: Supplementary file 10 [file pone.0360505.s026.docx]

| **Name of tandem repeats** | **Tandem Repeats Sequences** | **Position of B-cell Epitopes** | **B-cell epitopes** | **Scores** | **Antigenic Scores** | **Location** | **No. of times the specific B-cell epitopes were found** | **Countries where the B-cell epitopes were found** |
| --- | --- | --- | --- | --- | --- | --- | --- | --- |
| A | SGVLSQSGQASTSSQLGTDSSSAGDQQQ | 11 | STSSQLGTDSSS | 0.467 | 1.1090 | outside | 6 | Philippines, Ecuador, Grenada, Brazil |
| B | SGVSSQSGQASTSSQLGADSSSAGGQQQ | 11 | STSSQLGADSSS | 0.715 | 1.0510 | outside | 4 | Philippines, Brazil |
| E | DSSSASGQQQESSVLPQSDQASTSSQLG | 3 | SSASGQQQESSV  (Type 1) | 0.946 | 1.2768 | inside | 38 | Colombia, Philippines, Grenada, Brazil, Ecuador, Egypt, Thailand, South Africa |
| E | DSSSASGQQQESSVLPQSDQASTSSQLG | 16 | PQSDQASTSSQL | 0.565 | 0.9305 | outside | 2 | Brazil |
| F | DSSPASGQQQESSVLPQSDQASTSSQLG | 3 | SPASGQQQESSV | 0.986 | 0.8510 | outside | 1 | Brazil |
| F | DSSPASGQQQESSVLPQSDQASTSSQLG | 16 | PQSGQASTSSQL | 0.374 | 0.8794 | outside | 4 | Philippines, Brazil |
| I | DSSSASGQQQESGVLSQSGQASTSSQLG | 3 | SSASGQQQESGV  (Type 3) | 0.756 | 1.2743 | inside | 6 | Colombia, Egypt, Brazil |
| J | DSSSASGQQQESSVLSPSGQASTSSQLG | 16 | SPSGQASTSSQL | 0.584 | 1.1794 | outside | 4 | Grenada, Egypt, South Africa, Brazil |
| N | SSVLSQSDQASTSSQLGTDSSSASGQQQ | 5 | SQSDQASTSSQL | 0.501 | 0.9748 | outside | 12 | Colombia, Turkey, Philippines,  Egypt,  South Africa, Brazil |
| T | SGVSSQSDQASTSSQLGTDSSSASGQQQ | 2 | GVSSQSDQASTS | 0.881 | 1.3194 | outside | 1 | Brazil |
| V | SGVLSQSGQASTSSQLGTD | 7 | SGQASTSSQLGT | 0.327 | 1.1992 | outside | 3 | Colombia, Egypt, Brazil |
| W | DSSSASGQQQESSVSSQSDQASTSSQL | 4 | SASGQQQESSVS | 0.965 | 1.2575 | inside | 28 | Colombia, Turkey, Philippines, Grenada, Equador, Egypt, Thailand, Brazil |
| α | DNSSASGQQPESSVLSPSTSSQLGEWSG | 16 | SPSTSSQLGEWS | 0.978 | 0.6015 | outside | 1 | Brazil |
| α | DNSSASGQQPESSVLSPSTSSQLGEWSG | 3 | SSASGQQPESSV | 0.746 | 1.3888 | outside | 1 | Brazil |
| β | DSSSASGQQPESSVLSPSAQASTSSQLG | 5 | ASGQQPESSVLS | 0.856 | 1.3537 | outside | 1 | Brazil |
| Γ | TDNSSASGQQQESSVSSQSAQASTSSQLG | 18 | QSAQASTSSQLG | 0.339 | 0.9825 | inside | 2 | Colombia, Brazil |
| π | STSSQLGTGWRQEMRSKVASDE | 1 | STSSQLGTGWRQ | 0.967 | 0.0582 | outside | 1 | Brazil |
| Σ | STSSQLGADLRQEVHSKVASVE | 9 | DLRQEVHSKVAS | 0.418 | 1.6003 | inside | 1 | Brazil |
| σ | DSSSASDQQQESSVSSQSGQASTSSQLGA | 4 | SASDQQQESSVS | 0.914 | 1.1573 | inside | 2 | Colombia |
| μ | DSSSASGQQQESSVSSQSDEVSTSSQLG | 17 | QSDEVSTSSQLG | 0.749 | 0.9446 | outside | 1 | Colombia |
| τ | ITRQSGGQQQESSVSSQSGQASTSSQSGA | 4 | QSGGQQQESSVS | 0.974 | 1.1283 | inside | 1 | Colombia |
| Φ | SASGQQQESSVSSQSDQASSTSSQLGADSS | 19 | SSTSSQLGADSS | 0.292 | 1.0580 | outside | 3 | Colombia |
| 1 | SASGQQQESSVLSQSDQASSTSSQLGADSS | 2 | ASGQQQESSVLS | 0.909 | 1.1787 | inside | 4 | Colombia |
| 2 | SESGQQQESSVSSQSDQASTSSQSGAD | 1 | SESGQQQESSVS | 0.999 | 1.2790 | outside | 1 | Colombia |
| 3 | SSSASGQQQSESSVLSQSGQASTSSQLGAD | 3 | SASGQQQSESSV | 0.855 | 1.2136 | inside | 1 | Colombia |
| 8 | ADSSSAGGQQQESSVSSQSGQASTSSQL | 5 | SAGGQQQESSVS | 0.894 | 1.1696 | outside | 6 | Colombia-, Grenada, Ecuador, |
| 16 | ADSSSAGGQHQESSVSSQSDQASTSSQL | 16 | SSQSDQASTSSQ | 0.724 | 1.4025 | outside | 1 | Colombia |
| 17 | PDTSSAGGHHHKSSVSSQSGQASTSSQLG | 5 | SAGGHHHKSSVS | 0.611 | 1.4055 | outside | 1 | Colombia |
| 19 | DSSSAGDQQQESSVSSQSDASTSSQLG | 4 | SAGDQQQESSVS | 0.928 | 1.1617 | outside | 4 | Colombia, Philippines |
| 20 | DSSSAGDQQQESSVSSQSGHASTSSQLG | 17 | QSGHASTSSQLG | 0.682 | 1.0947 | outside | 1 | Colombia |
| 22 | DNSSARGHQQENSVVSPSGQASTSSQLG | 7 | GHQQENSVVSPS | 0.608 | 1.3097 | outside | 1 | Colombia |
| 23 | DSSSASDQQQESSVLSQSNQASTSSQLG | 3 | SSASDQQQESSV  (Type 4) | 0.904 | 1.3886 | inside | 4 | Colombia, Philippines |
| 26 | DSSSAGDQQQESSVLSQSGQASSTSSQLG | 3 | SSAGDQQQESSV  (Type 2) | 0.887 | 1.4895 | outside | 6 | Colombia, Turkey, Philippines, South Africa, |
|  |  | 3 | SSASDQQQDSIV | 0.364 | 1.2631 | inside | 1 | Colombia |
| 29 | ARQLGGQQQESSVLSQSDQASSTSSQLGAD | 2 | RQLGGQQQESSV | 0.853 | 1.0031 | inside | 1 | Colombia |
| 31 | SSASGQQESSVLSQSDQASTSSQSGTD | 3 | ASGQQESSVLSQ | 0.756 | 1.1203 | outside | 1 | Colombia |
| 41 | SSSASGQQQGSGVSYQSGQASTSINLETD | 5 | SGQQQGSGVSYQ | 0.654 | 0.9623 | outside | 1 | Colombia |
| 42 | SSSAGGQQQESSVLSQSGQASTSSQLGAD | 5 | GGQQQESSVLSQ | 0.728 | 1.0972 | outside | 1 | Colombia |
| 46 | DSSSASDQQQERGVLSQSGQASTSSQLG | 3 | SSASDQQQERGV | 0.923 | 1.1040 | inside | 1 | Colombia |
| 47 | DSSSASGQQQESSVSSQSDQASTSSQLG | 17 | QSDQASTSSQLG | 0.432 | 1.0110 | inside | 2 | Grenada, Ecuador |
| 53 | SSVSSQSDASTSSQLGAD | 3 | VSSQSDASTSSQ | 0.569 | 1.4745 | outside | 2 | Grenada |
| 55 | SGQQQESSVLSQSDQASTSSQLGTDSSS | 1 | SGQQQESSVLSQ | 0.871 | 1.1364 | outside | 2 | Ecuador |
| 58 | SGVSSQSGQASTSSQLGTD | 4 | SSQSGQASTSSQ | 0.441 | 1.4877 | outside | 4 | Turkey, Grenada, Ecuador |
| 72 | TDSSSTSGQQQESSVLSQSDQ-ASTSSQS | 8 | QQQESGVSSQSD | 0.909 | 1.6348 | inside | 9 | Turkey, Egypt |
| 74 | DSSSASGQQQESGVLSQSGQASTSSQSG | 4 | SAGNQQQESSVS | 0.989 | 1.0628 | inside | 6 | Egypt, South Africa |
| 78 | SSSASGQQQESSVLSQGGQASTSSQLGT | 15 | SQGGQASTSSQL | 0.543 | 1.2885 | outside | 1 | Egypt |
| 81 | ADSSSASGQQQESGVPSQSGQASTSSQL | 7 | SGQQQESGVPSQ | 0.852 | 1.3488 | outside | 3 | Turkey, Egypt |
| 86 | ADSSSASGQQQESSVPSQSGASTSSQL | 7 | SGQQQESSVPSQ | 0.983 | 1.1848 | outside | 1 | Egypt |
| 87 | ADSSSASGQQQESGVPSQSGASTSSQL | 13 | SGVPSQSGASTS | 0.867 | 1.0209 | outside | 1 | Egypt |
| 89 | ADSSSASGQQQESGVSSQSSHASTSSQL | 7 | SGQQQESGVSSQ | 0.906 | 1.4769 | outside | 1 | Egypt |
| 90 | ADSSSASGQQQESGVPSQSDQASTSSQL | 9 | QQQESGVPSQSD | 0.892 | 1.1920 | outside | 1 | Egypt |
| 91 | AGSSSASGQQQESGVSSQSEQASTSSQL | 10 | QQESGVSSQSEQ | 0.993 | 1.3242 | inside | 1 | Egypt |
| 92 | SSVSSQSDASTSSQLGTDSSSAGDQQQ | 16 | GTDSSSAGDQQQ | 0.371 | 1.7299 | outside | 1 | Grenada |
| 97 | DSSSASGQQQESSVLSQSDQASTLSQLG | 16 | SQSDQASTLSQL | 0.531 | 0.3748 | outside | 2 | Philippines |
| 98 | DSSSASGQRQESSVLSQSDQASTLSQLG | 3 | SSASGQRQESSV | 0.889 | 1.0568 | inside | 1 | Philippines |
| 100 | TDSSSASGQQQESSVLSQSGASTSSQLGT | 18 | QSGASTSSQLGT | 0.531 | 1.1174 | outside | 2 | Philippines, South Africa |
| 104 | ADSSSASDQQQESGVPSQSEASTSSQL | 9 | QQQESGVPSQSE | 0.897 | 1.1560 | inside | 2 | Philippines |
| 116 | DSSSASGQHQESTVSSQSDASTSAQLD | 11 | ESTVSSQSDAST | 0.902 | 1.4949 | outside | 1 | Grenada |
| 117 | SSSASGQQQGGGVSPQSEASTSSQLGT | 5 | SGQQQGGGVSPQ | 1 | 1.6891 | outside | 1 | Turkey |
| 118 | SSSASGQQQESGVSPQSEASTSSQLGT | 5 | SGQQQESGVSPQ | 0.898 | 1.1532 | inside | 2 | Turkey |
| 123 | SSSASGQQQESGVPSQSGQASTSSQLGA | 5 | SGQQQESGVPSQ | 0.852 | 1.3488 | outside | 1 | Turkey, Egypt |
| 126 | DSSSAGDQQQESGVSSQSGQASTSSQLG | 7 | DQQQESGVSSQS | 0.833 | 1.6185 | inside | 1 | Turkey |
| 127 | GVLSQSGQASTSSQLGTDSSSAGDQQQE | 17 | TDSSSAGDQQQE | 0.648 | 1.7397 | outside | 2 | Turkey |
| 130 | DSSSAGNQQQESSVSSQSGASTSSQLGT | 17 | QSGASTSSQLGT | 0.531 | 1.1174 | outside | 2 | Philippines, South Africa |
| 134 | DSSSAGNQQQESSVSSQSDASTSSQLGT | 17 | QSDASTSSQLGT | 0.419 | 1.1514 | outside | 1 | South Africa |
| 135 | DSSSASGQQQESSVLPQSDASTSSQLGA | 16 | PQSDASTSSQLG | 0.702 | 1.0478 | outside | 1 | South Africa |
| 147 | DSSSASGQQQESGLSSQSEASTSSQLG | 3 | SSASGQQQESGL | 0.604 | 1.1579 | outside | 1 | Russia |
| 148 | DSSSASDQQQESGLSSQSEASTSSQLG | 3 | SSASDQQQESGL | 0.67 | 1.2698 | outside | 1 | Russia |
| 149 | SSSASDQQQGSGVSSQSEASTSSQLGT | 7 | QQQGSGVSSQSE | 0.908 | 1.7441 | inside | 1 | Russia |
| 150 | SSSASGQQQESGVSSQSEASTSSQLGT | 7 | QQQESGVSSQSE | 0.957 | 1.4868 | inside | 4 | Russia |
| 152 | ADSLSAGNQQQESDVSSQSEASTSSQL | 9 | QQQESDVSSQSE | 0.97 | 1.2944 | inside | 1 | Russia |
| 155 | DSSSASSQQQESSVLSQSDQASTSSQLG | 6 | SSQQQESSVLSQ | 0.967 | 1.0919 | inside | 1 | Thailand |

MODEL SEQUENCE SSAGGQQQESS VAXIJEN SCORE- **1.3498**
