# Supplementary material for: Nomenclature, classification, and in-silico structural analysis of MSP1a variants of Anaplasma marginale
Source: PLoS One. 2026 Oct 21;21(10):e0360505. doi: 10.1371/journal.pone.0360505 (PMC13686480; doi:10.1371/journal.pone.0360505)
Supplement: Supplementary file 11 [file pone.0360505.s027.docx]

| **Name of tandem repeats** | **Tandem Repeats** | **B-cell epitopes** | **Blastp score** |
| --- | --- | --- | --- |
| A | SGVLSQSGQASTSSQLGTDSSSAGDQQQ | STSSQLGTDSSS | -3.0 |
| B | SGVSSQSGQASTSSQLGADSSSAGGQQQ | STSSQLGADSSS | -3.0 |
| C | SSVLSPSGQASTSSQLGADSSSASGQQQ | STSSQLGADSSS | -3.0 |
| D | SGVLSQSGQAST | N/A | N/A |
| E | DSSSASGQQQESSVLPQSDQASTSSQLG | SSASGQQQESSV | 34.0 |
|  |  | PQSDQASTSSQL | -13.00 |
| F | DSSPASGQQQESSVLPQSDQASTSSQLG | SPASGQQQESSV | 29.0 |
|  |  | PQSDQASTSSQL | -13.0 |
| G | GSSSASGQQQESSVLPQSGQASTSSQLG | SSASGQQQESSV | 34.0 |
|  |  | PQSGQASTSSQL | -6.0 |
| H | DSSSASGQQQESSVLSQSGASTSSQL | SSASGQQQESSV | 34.0 |
| I | DSSSASGQQQESGVLSQSGQASTSSQLG | SSASGQQQESGV | 30.0 |
| J | DSSSASGQQQESSVLSPSGQASTSSQLG | SSASGQQQESSV | 34.0 |
|  |  | SPSGQASTSSQL | -2.0 |
| K | DSSSASGQQQESGVLSQSGQASTSSQL | SSASGQQQESGV | 30.0 |
| L | SGVLSQSGQASTSSQLGTDSSSASGQQQ | STSSQLGTDSSS | -3.0 |
| M | SSVLPQSGQASTSSQLGAGSSSASGQQQ | PQSGQASTSSQL | -6.0 |
| N | SSVLSQSDQASTSSQLGTDSSSASGQQQ | SQSDQASTSSQL | -8.0 |
| O | SSVLSQSGASTSSQ | N/A |  |
| P | DSSSASGQQQESSVLPQSGQASTSSQLG | SSASGQQQESSV | 34.0 |
|  |  | PQSGQASTSSQL | -6.0 |
| Q | DSSSASGQQQESSVLSQSDQASTSSQLG | SSASGQQQESSV | 34.0 |
|  |  | SQSDQASTSSQL | -8.0 |
| R | DSSSASGQQQESSVLSQSDQASTSSQL | SSASGQQQESSV | 34.0 |
|  |  | SQSDQASTSSQL | -8.0 |
| S | SGVSSQSGQASTSSQLGTDSSSAGDQQQ | STSSQLGTDSSS | -3.0 |
| T | SGVSSQSDQASTSSQLGTDSSSASGQQQ | GVSSQSDQASTS | 0.0 |
| U | SGVSSQSDQASTSSQLGTDSSSASGQQQ | N/A | N/A |
| V | SGVLSQSGQASTSSQLGTD | SGQASTSSQLGT | -3.0 |
| W | DSSSASGQQQESSVSSQSDQASTSSQL | SASGQQQESSVS | 18.0 |
| α | DNSSASGQQPESSVLSPSTSSQLGEWSG | SPSTSSQLGEWS | 3.0 |
|  |  | SSASGQQPESSV | 28.0 |
| β | DSSSASGQQPESSVLSPSAQASTSSQLG | ASGQQPESSVLS | 6.0 |
| Γ | TDNSSASGQQQESSVSSQSAQASTSSQLG | SASGQQQESSVS | 18.0 |
|  |  | QSAQASTSSQLG | -5.0 |
| π | STSSQLGTGWRQEMRSKVASDE | STSSQLGTGWRQ | -13.0 |
| Σ | STSSQLGADLRQEVHSKVASVE | DLRQEVHSKVAS | -15.0 |
| σ | DSSSASDQQQESSVSSQSGQASTSSQLGA | SASDQQQESSVS | -21.0 |
| μ | DSSSASGQQQESSVSSQSDEVSTSSQLG | SASGQQQESSVS | -18.0 |
|  |  | QSDEVSTSSQLG | -21.0 |
| τ | ITRQSGGQQQESSVSSQSGQASTSSQSGA | QSGGQQQESSVS | -14.0 |
| Φ | SASGQQQESSVSSQSDQASSTSSQLGADSS | SASGQQQESSVS | -18.0 |
|  |  | SSTSSQLGADSS | -25.0 |
| 1 | SASGQQQESSVLSQSDQASSTSSQLGADSS | ASGQQQESSVLS | -14.0 |
|  |  | SSTSSQLGADSS | -25.0 |
| 2 | SESGQQQESSVSSQSDQASTSSQSGAD | SESGQQQESSVS | -14.0 |
| 3 | SSSASGQQQSESSVLSQSGQASTSSQLGAD | SASGQQQSESSV | -18.0 |
| 4 | TDSSSSASGQQQESSVLSQSDQASSTSSQL | SSASGQQQESSV | -15.0 |
| 5 | SSSASGQQQESGVLSQSDQASTSSQLGAD | SSASGQQQESGV | -15.0 |
|  |  | SQSDQASTSSQL | -18.0 |
| 6 | TDSSSSASGQQQESSVLSQSDQASSTSSQL | SSASGQQQESSV | -15.0 |
| 7 | ADISSASGQQQESSVSSQSDQASTSSQL | SASGQQQESSVS | -18.0 |
| 8 | ADSSSAGGQQQESSVSSQSGQASTSSQL | SAGGQQQESSVS | -18.0 |
| 9 | TDSSSASGQQQESSVSSQSGQASTSSQS | SASGQQQESSVS | -18.0 |
| 10 | ADSSSASGQQQESSVSSQSGQASTSSQL | SASGQQQESSVS | -18.0 |
| 11 | ADSSSASGQQQESSVSSQSDQASTSSQL | SASGQQQESSVS | -18.0 |
| 12 | TDTSSASGQQQESSVSSQSGQASTSSQS | SASGQQQESSVS | -18.0 |
| 13 | TDSSSSASGQQQESSVLSQSDQASSTSSQL | SSASGQQQESSV | -15.0 |
| 14 | ADSSSASGQQQESSVLSQSDQASTSSQL | SSASGQQQESSV | -15.0 |
|  |  | SQSDQASTSSQL | -18.0 |
| 15 | ADSSSAGGQQQESSVSSQSDQASTSSQL | SAGGQQQESSVS | -18.0 |
| 16 | ADSSSAGGQHQESSVSSQSDQASTSSQL | SSQSDQASTSSQ | -20.0 |
| 17 | PDTSSAGGHHHKSSVSSQSGQASTSSQLG | SAGGHHHKSSVS | -26.0 |
| 18 | DSSSASGQQQESGVLSQSGQASTSSQLG | SSASGQQQESGV | -15.0 |
| 19 | DSSSAGDQQQESSVSSQSDASTSSQLG | SAGDQQQESSVS | -21.0 |
| 20 | DSSSAGDQQQESSVSSQSGHASTSSQLG | SAGDQQQESSVS | -21.0 |
|  |  | QSGHASTSSQLG | -23.0 |
| 21 | DSSSASGQQQESSVLSQSSQASTSSQLG | SSASGQQQESSV | -15.0 |
| 22 | DNSSARGHQQENSVVSPSGQASTSSQLG | GHQQENSVVSPS | -21.0 |
| 23 | DSSSASDQQQESSVLSQSNQASTSSQLG | SSASDQQQESSV | -18.0 |
| 24 | DSSSASDQQQESSVLSQSDQASTSSQLG | SSASDQQQESSV | -18.0 |
|  |  | SQSDQASTSSQL | -18.0 |
| 25 | YCSSAGGQQQESSVSSQSGQASTSSQL | SAGGQQQESSVS | -18.0 |
| 26 | DSSSAGDQQQESSVLSQSGQASSTSSQLG | SSAGDQQQESSV | -15.0 |
| 27 | DSSSASDQQQESSVLSQSNQASTSSLLG | SSASDQQQESSV | -18.0 |
| 28 | NSSSASDQQQDSIVLSQSDQASTSSQL | SQSDQASTSSQL | -18.0 |
|  |  | SSASDQQQDSIV | -18.0 |
| 29 | ARQLGGQQQESSVLSQSDQASSTSSQLGAD | RQLGGQQQESSV | -16.0 |
| 30 | TDSSSSASGQQQESSVLSQSDQASSTSSQL | SSASGQQQESSV | -15.0 |
| 31 | SSASGQQESSVLSQSDQASTSSQSGTD | ASGQQESSVLSQ | -14.0 |
| 32 | SASGQQQESSVLSQSDQASSTSSQLGADSS | ASGQQQESSVLS | -14.0 |
|  |  | SSTSSQLGADSS | -25.0 |
| 33 | SASGQQQESSVLSQSDQASTSSQSGTDSS | ASGQQQESSVLS | -14.0 |
| 34 | SASGQQQESSVLSQSDQASTSSQLGA | ASGQQQESSVLS | -14.0 |
| 35 | TDSSSAGDQQQESSVLSQSDQASTSSQLG | SSAGDQQQESSV | -15.0 |
|  |  | SQSDQASTSSQL | -18.0 |
| 36 | ADSSSASGQQQESSVLSQSAQASTSSQLG | SSASGQQQESSV | -15.0 |
|  |  | QSAQASTSSQLG | -18.0 |
| 37 | DNSSASDQQQESSVSSQSGRASTSSQLG | SASDQQQESSVS | -18.0 |
| 38 | DSSSASGQQQESSVSSQSGQASTSSQLG | SASGQQQESSVS | -18.0 |
| 39 | DSSSASGQQQESSVLSQSGQASTSSQSG | SSASGQQQESSV | -15.0 |
| 40 | DSSSASGQQQESSVLSQSGQASTSSQLG | SSASGQQQESSV | -15.0 |
| 41 | SSSASGQQQGSGVSYQSGQASTSINLETD | SGQQQGSGVSYQ | -18.0 |
| 42 | SSSAGGQQQESSVLSQSGQASTSSQLGAD | GGQQQESSVLSQ | -19.0 |
| 43 | SSSAGDQQQESSVSSQSGQASTSSQLGTD | SAGDQQQESSVS | -21.0 |
|  |  | SGQASTSSQLGT | -20.0 |
| 44 | SSSASGQQQESSVLSQSGQASTSSQL | SSASGQQQESSV | -15.0 |
| 45 | DSSSAGDQQQESSVLSQSGQASSTSSQLG | SSAGDQQQESSV | -15.0 |
| 46 | DSSSASDQQQERGVLSQSGQASTSSQLG | SSASDQQQERGV | -18.0 |
|  |  |  |  |
| 47 | DSSSASGQQQESSVSSQSDQASTSSQLG | SASGQQQESSVS | 18 |
|  |  | QSDQASTSSQLG | -10 |
| 48 | AGSSSAGGQQQESSVSSQSDQASTSSQL | SAGGQQQESSVS | 23 |
| 49 | ADSSSASGQQQESSVLSPSGQASTSSQLGV | SSASGQQQESSV | 34 |
|  |  | SPSGQASTSSQL | -2 |
| 50 | ADSSSASGQQQESSVSSQSDASTSSQ | SASGQQQESSVS | 18 |
| 51 | ADSSSAGGQQQESSVSSQSDQASTSSQS | SAGGQQQESSVS | 23 |
| 52 | SGVSSQSGQASTSSQLGTDSSSASGQQQ | STSSQLGTDSSS | -3 |
| 53 | SSVSSQSDASTSSQLGAD | VSSQSDASTSSQ | -4 |
| 54 | TDSSSASGQQQESSVSSQSGQASTSSQL | SASGQQQESSVS | 18 |
| 55 | SGQQQESSVLSQSDQASTSSQLGTDSSS | SGQQQESSVLSQ | 9.0 |
|  |  | STSSQLGTDSSS | -3.0 |
| 56 | SGQQQESSVLSQSDQASTSSQSGTD | SGQQQESSVLSQ | 9.0 |
| 57 | DSSSASGQQQESSVLSQSDQASTSSQSG | SSASGQQQESSV | 34.0 |
| 58 | SGVSSQSGQASTSSQLGTD | SSQSGQASTSSQ | 12.0 |
| 59 | DSSSASGQQQESSVSSQSEASTSSQLG | SASGQQQESSVS | 18.0 |
| 60 | SSSASGQQQESSVSSQSEASTSSQLGT | SASGQQQESSVS | 18.0 |
| 61 | AGSSSAGGQQQESSVSSQSEASTSSQLG | SAGGQQQESSVS | 23.0 |
| 62 | ADSSSASGQQQESSVLSQSEASTSSQLG | SSASGQQQESSV | 34.0 |
| 63 | ADSSSASGQQQESSVSSQSEASTSSQLG | SASGQQQESSVS | 18.0 |
| 64 | ADSSSASGQQQESSVSSQSEASTSSQL | SASGQQQESSVS | 18.0 |
| 65 | SSSASGQQQESSVSSQSEASTSSQLGA | SASGQQQESSVS | 18.0 |
| 66 | SSSASGQQQESSVSSQSDQASTSSQLGT | SASGQQQESSVS | 18.0 |
|  |  | QSDQASTSSQLG | -10.0 |
| 67 | SSSASGQQQESSVSSQSEASTSSQLGGA | SASGQQQESSVS | 18.0 |
| 68 | SSSASGQQQESSVSSQSEASTSSQL | SASGQQQESSVS | 18.0 |
| 69 | TDSSSASGQQQESSVLSPSGQASTSSQL | SSASGQQQESSV | 31.2 |
|  |  | SPSGQASTSSQL | 8.7 |
| 70 | ADSSSASGQQQESSVLSPSGQ-ASTSSQS | SSASGQQQESSV | 31.2 |
| 71 | TDSSSASGQQQESSVLSQSDQ-ASTSSQL | SSASGQQQESSV | 31.2 |
|  |  | SQSDQASTSSQL | No significant similarity found |
| 72 | TDSSSTSGQQQESSVLSQSDQ-ASTSSQS | QQQESGVSSQSD | 19.3 |
| 73 | DSSSASGQQQESGVSSQSDQASTSSQLG | QQQESGVSSQSD | 19.3 |
| 74 | DSSSASGQQQESGVLSQSGQASTSSQSG | SAGNQQQESSVS | 28.2 |
| 75 | ADSSSASGQQQESGVSSQSDQASTSSQL | SSASGQQQESGV | 28.6 |
| 76 | TDSSSASGQQQESGVSSQSDQASTSSQL | QQQESGVSSQSD | 19.3 |
| 77 | ADSSSASGQQQESGVLSQSGQASTSSQL | SSASGQQQESGV | 28.6 |
| 78 | SSSASGQQQESSVLSQGGQASTSSQLGT | SSASGQQQESSV | 31.2 |
|  |  | SQGGQASTSSQL | 11.2 |
| 79 | SSSASGQQQESSVSSQSGQASTSSQLGT | SASGQQQESSVS | 28.6 |
|  |  | SGQASTSSQLGT | 8.3 |
| 80 | SSSASGQQQESSVSSQSGQASTSSQLGA | SASGQQQESSVS | 28.6 |
| 81 | ADSSSASGQQQESGVPSQSGQASTSSQL | SGQQQESGVPSQ | 21.8 |
| 82 | TDSSSASGQQQESGVPSQSGQASTSSQL | SGQQQESGVPSQ | 21.8 |
| 83 | TDSSSASGQQQESGVPSQSGQASTSSQLGS | SGQQQESGVPSQ | 21.8 |
| 84 | ADSSSASGQQQESGVSSQSDASTSSQL | QQQESGVSSQSD | 19.3 |
| 85 | ADSSSASGQQQESGVSSQSDQASTSSQL | QQQESGVSSQSD | 19.3 |
| 86 | ADSSSASGQQQESSVPSQSGASTSSQL | SGQQQESSVPSQ | 24.4 |
| 87 | ADSSSASGQQQESGVPSQSGASTSSQL | SGVPSQSGASTS | No significant similarity found |
| 88 | TDSSSASGQQQESGVSSQSDASTSSQL | QQQESGVSSQSD | 19.3 |
| 89 | ADSSSASGQQQESGVSSQSSHASTSSQL | SGQQQESGVSSQ | 21.8 |
| 90 | ADSSSASGQQQESGVPSQSDQASTSSQL | QQQESGVPSQSD | 19.3 |
| 91 | AGSSSASGQQQESGVSSQSEQASTSSQL | QQESGVSSQSEQ | 15.9 |
| 92 | SSVSSQSDASTSSQLGTDSSSAGDQQQ | VSSQSDASTSSQ | -4 |
|  |  | GTDSSSAGDQQQ | 3 |
| 93 | SGVSSQSGQASTSSQLGT | SSQSGQASTSSQ | 12 |
| 94 | TDSSSASGQQQESSVLSQSDQASTSSQS | SSASGQQQESSV | 34 |
| 95 | TDSSSASGQQQESSVLPQSGQASTSSQL | SSASGQQQESSV | 34.0 |
|  |  | PQSGQASTSSQL | -6.0 |
| 96 | GTD |  |  |
| 97 | DSSSASGQQQESSVLSQSDQASTLSQLG | SSASGQQQESSV | 34.0 |
|  |  | SQSDQASTLSQL | -11.0 |
| 98 | DSSSASGQRQESSVLSQSDQASTLSQLG | SSASGQRQESSV | 30.0 |
|  |  | SQSDQASTLSQL | -11.0 |
| 99 | TDSSSASGQQQESSVLSQSDQASTSSQLG | SSASGQQQESSV | 34.0 |
|  |  |  |  |
|  |  |  |  |
|  |  | SQSDQASTSSQL | -8.0 |
|  |  |  |  |
|  |  |  |  |
| 100 | TDSSSASGQQQESSVLSQSGASTSSQLGT | SSASGQQQESSV | 34.0 |
|  |  | QSGASTSSQLGT | -3.0 |
| 101 | TD |  |  |
| 102 | ADSSSAGDQQQESSVLSQSGQASTSSQL | SSAGDQQQESSV | 33.0 |
| 103 | GAD |  |  |
| 104 | ADSSSASDQQQESGVPSQSEASTSSQL | QQQESGVPSQSE | -4.0 |
| 105 | ADSSSASGQQQESGVPSQSEASTSSQL | QQQESGVPSQSE | -4.0 |
| 106 | DSSSASDQQQESSVLSQSGQASTSSQLG | SSASDQQQESSV | 27.0 |
| 107 | TDSSSASGQQQESSVLSQSGQASTSSQL | SSASGQQQESSV | 34.0 |
| 108 | ADSSSASGQQQESSVSSQL | SASGQQQESSVS | 18.0 |
| 109 | ADSSSASGQQQESSVSSQ | SASGQQQESSVS | 18.0 |
| 110 | DSSSAGDQQQESSVSSQSGASTSSQLG | SAGDQQQESSVS | 16.0 |
| 111 | SGVLSKSDQASTSSQLGADSSSASGQQQ | STSSQLGADSSS | -3.0 |
| 112 | SSVLSQSDQASTSSQLGADSSSASGQQQ | STSSQLGADSSS | -3.0 |
| 113 | SSVLSQSDQASTSSQL | SQSDQASTSSQL | -8.0 |
| 114 | SGVLSKSDQASTSSQLGTDSSSAGDQQQ | STSSQLGTDSSS | -3.0 |
| 115 | DSSSASGQQQESSVSSQSEASTSSHLG | SASGQQQESSVS | 18 |
| 116 | DSSSASGQHQESTVSSQSDASTSAQLD | ESTVSSQSDAST | 2 |
| 117 | SSSASGQQQGGGVSPQSEASTSSQLGT | SGQQQGGGVSPQ | -5 |
| 118 | SSSASGQQQESGVSPQSEASTSSQLGT | SGQQQESGVSPQ | 5 |
| 119 | SSSASGQQQESGVSPQSEASTSSQL | SGQQQESGVSPQ | 5 |
| 120 | DSSSASGQQQESSVSSQSDASTSSQLG | SASGQQQESSVS | 18 |
| 121 | DSSSASGQQQESGVSSQSDASTSSQL | QQQESGVSSQSD | -3 |
| 122 | AGSSSASGQQQESGVSSQSDASTSSQL | QQQESGVSSQSD | -3 |
| 123 | SSSASGQQQESGVPSQSGQASTSSQLGA | SGQQQESGVPSQ | 5 |
| 124 | SSSASGQQQESGVSSQSDASTSSQLGA | QQQESGVSSQSD | -3 |
| 125 | DSSSAGDQQQESSVLSQSDQASTSSQLG | SSAGDQQQESSV | 33 |
|  |  | SQSDQASTSSQL | -8 |
| 126 | DSSSAGDQQQESGVSSQSGQASTSSQLG | DQQQESGVSSQS | -3 |
| 127 | GVLSQSGQASTSSQLGTDSSSAGDQQQE | TDSSSAGDQQQE | 10 |
| 128 | GVSSQSGQASTSSQLGTDSSSAGDQQQE | TDSSSAGDQQQE | 10 |
|  |  | SSQSGQASTSSQ | 12 |
| 129 | GVSSQSGQASTSSQLGTDSSSAGDQQQE | SSQSGQASTSSQ | 12 |
| 130 | DSSSAGNQQQESSVSSQSGASTSSQLGT | SAGNQQQESSVS | 17 |
|  |  | QSGASTSSQLGT | -3 |
| 131 | DSSSAGDQQQESSVLSPSGQASTSSQSGT | SSAGDQQQESSV | 33 |
| 132 | DSSSASGQQQESSVLSQSSQASTSSQLGA | SSASGQQQESSV | 34 |
| 133 | DSSSAGNQQQESSVSSQSDASTSSQLGA | SAGNQQQESSVS | 17 |
| 134 | DSSSAGNQQQESSVSSQSDASTSSQLGT | SAGNQQQESSVS | 17 |
|  |  | QSDASTSSQLGT | -3 |
| 135 | DSSSASGQQQESSVLPQSDASTSSQLGA | SSASGQQQESSV | 34 |
|  |  | PQSDASTSSQLG | -11 |
| 136 | DSSSAGNQQQESSVSSQSDASTSSQSGA | SAGNQQQESSVS | 17 |
| 137 | DSSSAGNQQQESSVSSQSDASTSSQLG | SAGNQQQESSVS | 17 |
| 138 | DSSSASGQQQESSVLPQSGQASTSSQSGA | SSASGQQQESSV | 34 |
| 139 | TDSSSASGQQQESSVLSPSGQASTSSQLG | SSASGQQQESSV | 34 |
|  |  | SPSGQASTSSQL | -2 |
| 140 | ADSSSASGQQQESSVLSPSGQASTSSQSG | SSASGQQQESSV | 34 |
| 141 | TDSSSASGQQQESSVLSQSDQASTSSQLG | SSASGQQQESSV | 34 |
|  |  | SQSDQASTSSQL | -8 |
| 142 | TDSSSASGQQQESSVLSQSDQASTSSQ | SSASGQQQESSV | 34 |
| 143 | SSQSGADSS | N/A | N/A |
| 144 | SSQSGQAST |  |  |
| 145 | SASGQQQESSV |  |  |
| 146 | SSQ |  |  |
| 147 | DSSSASGQQQESGLSSQSEASTSSQLG | SSASGQQQESGL | 30.0 |
| 148 | DSSSASDQQQESGLSSQSEASTSSQLG | SSASDQQQESGL | 23.0 |
| 149 | SSSASDQQQGSGVSSQSEASTSSQLGT | QQQGSGVSSQSE | 3.0 |
| 150 | SSSASGQQQESGVSSQSEASTSSQLGT | QQQESGVSSQSE | -3.0 |
| 151 | ADSSSASGQQQESGVSSQSEASTSSQL | QQQESGVSSQSE | -3.0 |
| 152 | ADSLSAGNQQQESDVSSQSEASTSSQL | QQQESDVSSQSE | -9.0 |
| 153 | ADSSSASGQQQESGVSSQSEASTSSQLGADS | QQQESGVSSQSE | -3.0 |
| 154 | DSSSASGQQQESGVSSQSEASTSSQLG | QQQESGVSSQSE | 0.957 |
| 155 | DSSSASSQQQESSVLSQSDQASTSSQLG | SSQQQESSVLSQ | -18.0 |
| 156 | STSSQLGTDSSSASGQQQESSVSSQSGQ | SASGQQQESSVS | -14.0 |
| 157 | DGSSASGQQQESSVLSQSGQASTSSQLG | SSASGQQQESSV | -14.0 |
