# Supplementary material for: Nomenclature, classification, and in-silico structural analysis of MSP1a variants of Anaplasma marginale
Source: PLoS One. 2026 Oct 21;21(10):e0360505. doi: 10.1371/journal.pone.0360505 (PMC13686480; doi:10.1371/journal.pone.0360505)
Supplement: Supplementary file 12 [file pone.0360505.s028.docx]

| S.I. | Name of Tandem repeats | Tandem repeats | 2D structure |
| --- | --- | --- | --- |
| 1. | E | DSSSASGQQQESSVLPQSDQASTSSQLG | a-c |
| 2. | G | GSSSASGQQQESSVLPQSGQASTSSQLG | a-c |
| 3. | H | DSSSASGQQQESSVLSQSGASTSSQL | c-c |
| 4. | L | DSSSASGQQQESSVLSPSGQASTSSQLG | c-c |
| 5. | P | DSSSASGQQQESSVLPQSGQASTSSQLG | c-c |
| 6. | Q | DSSSASGQQQESSVLSQSDQASTSSQLG | a-c |
| 7. | R | DSSSASGQQQESSVLSQSDQASTSSQL | c-c |
| 8. | 4 | TDSSSSASGQQQESSVLSQSDQASSTSSQL | a-c |
| 9. | 6 | TDSSSSASGQQQESSVLSQSDQASSTSSQL | a-c |
| 10. | 13 | TDSSSSASGQQQESSVLSQSDQASSTSSQL | a-c |
| 11. | 14 | ADSSSASGQQQESSVLSQSDQASTSSQL | c-c |
| 12. | 21 | DSSSASGQQQESSVLSQSSQASTSSQLG | a-c |
| 13. | 30 | TDSSSSASGQQQESSVLSQSDQASSTSSQL | a-c |
| 14. | 36 | ADSSSASGQQQESSVLSQSAQASTSSQLG | a-c |
| 15. | 39 | DSSSASGQQQESSVLSQSGQASTSSQSG | a-c |
| 16. | 40 | DSSSASGQQQESSVLSQSGQASTSSQLG | a-c |
| 17. | 44 | SSSASGQQQESSVLSQSGQASTSSQL | a-c |
| 18. | 49 | ADSSSASGQQQESSVLSPSGQASTSSQLGV | a-c |
| 19. | 57 | DSSSASGQQQESSVLSQSDQASTSSQSG | a-a |
| 20. | 62 | ADSSSASGQQQESSVLSQSEASTSSQLG | c-c |
| 21. | 69 | TDSSSASGQQQESSVLSPSGQASTSSQL | c-c |
| 22. | 70 | ADSSSASGQQQESSVLSPSGQ-ASTSSQS | c-c |
| 23. | 71 | TDSSSASGQQQESSVLSQSDQ-ASTSSQL | c-c |
| 24. | 78 | SSSASGQQQESSVLSQGGQASTSSQLGT | a-c |
| 25. | 94 | TDSSSASGQQQESSVLSQSDQASTSSQS | a-c |
| 26. | 95 | TDSSSASGQQQESSVLPQSGQASTSSQL | c-c |
| 27. | 97 | DSSSASGQQQESSVLSQSDQASTLSQLG | a-a |
| 28. | 99 | TDSSSASGQQQESSVLSQSDQASTSSQLG | a-c |
| 29. | 100 | TDSSSASGQQQESSVLSQSGASTSSQLGT | a-c |
| 30. | 107 | TDSSSASGQQQESSVLSQSGQASTSSQL | c-c |
| 31. | 132 | DSSSASGQQQESSVLSQSSQASTSSQLGA | a-a |
| 32. | 135 | DSSSASGQQQESSVLPQSDASTSSQLGA | a-c |
| 33. | 138 | DSSSASGQQQESSVLPQSGQASTSSQSGA | a-c |
| 34. | 139 | TDSSSASGQQQESSVLSPSGQASTSSQLG | b-c |
| 35. | 140 | ADSSSASGQQQESSVLSPSGQASTSSQSG | a-c |
| 36. | 141 | TDSSSASGQQQESSVLSQSDQASTSSQLG | a-c |
| 37. | 142 | TDSSSASGQQQESSVLSQSDQASTSSQ | a-c |
| 38. | 157 | DGSSASGQQQESSVLSQSGQASTSSQLG | c-c |
|  |  |  |  |
|  |  |  |  |

25 a-helixes
