# Supplementary material for: Nomenclature, classification, and in-silico structural analysis of MSP1a variants of Anaplasma marginale
Source: PLoS One. 2026 Oct 21;21(10):e0360505. doi: 10.1371/journal.pone.0360505 (PMC13686480; doi:10.1371/journal.pone.0360505)
Supplement: Supplementary file 13 [file pone.0360505.s029.docx]

| Name of Tandem repeats | Tandem repeats | 2d structure |
| --- | --- | --- |
| 26 | DSSSAGDQQQESSVLSQSGQASSTSSQLG | a-c |
| 35 | TDSSSAGDQQQESSVLSQSDQASTSSQLG | a-c |
| 45 | DSSSAGDQQQESSVLSQSGQASSTSSQLG | a-c |
| 102 | ADSSSAGDQQQESSVLSQSGQASTSSQL | a-c |
| 125 | DSSSAGDQQQESSVLSQSDQASTSSQLG | a-a |
| 131 | DSSSAGDQQQESSVLSPSGQASTSSQSGT | a-c |
| 77 | ADSSSASGQQQESGVLSQSGQASTSSQL | a-c |
|  |  |  |
|  |  |  |
|  |  |  |
|  |  |  |
|  |  |  |
|  |  |  |
|  |  |  |
|  |  |  |
|  |  |  |
|  |  |  |
|  |  |  |
|  |  |  |
|  |  |  |

Type 2 b-cell epitope, SSAGDQQQESSV

100% a-helixes
