# Supplementary material for: Nomenclature, classification, and in-silico structural analysis of MSP1a variants of Anaplasma marginale
Source: PLoS One. 2026 Oct 21;21(10):e0360505. doi: 10.1371/journal.pone.0360505 (PMC13686480; doi:10.1371/journal.pone.0360505)
Supplement: Supplementary file 14 [file pone.0360505.s030.docx]

Type 3 b-cell epitope, SSASGQQQESGV

| Name of Tandem repeats | Tandem repeats | 2d structure of Tandem repeats |
| --- | --- | --- |
| I | DSSSASGQQQESGVLSQSGQASTSSQLG | a-c |
| K | DSSSASGQQQESGVLSQSGQASTSSQL | c-c |
| 5 | SSSASGQQQESGVLSQSDQASTSSQLGAD | a-a |
| 18 | DSSSASGQQQESGVLSQSGQASTSSQLG | a-c |
| 75 | ADSSSASGQQQESGVSSQSDQASTSSQL | a-c |
| 77 | ADSSSASGQQQESGVLSQSGQASTSSQL | a-c |
|  |  |  |

83.33% a-helixes
