# Supplementary material for: Nomenclature, classification, and in-silico structural analysis of MSP1a variants of Anaplasma marginale
Source: PLoS One. 2026 Oct 21;21(10):e0360505. doi: 10.1371/journal.pone.0360505 (PMC13686480; doi:10.1371/journal.pone.0360505)
Supplement: Supplementary file 15 [file pone.0360505.s031.docx]

Type 4 b-cell epitope, SSASDQQQESSV

| Name of Tandem repeat | Tandem repeat | 2d structure |
| --- | --- | --- |
| 23 | DSSSASDQQQESSVLSQSNQASTSSQLG | a-c |
| 24 | DSSSASDQQQESSVLSQSDQASTSSQLG | a-a |
| 27 | DSSSASDQQQESSVLSQSNQASTSSLLG | a-c |
| 106 | DSSSASDQQQESSVLSQSGQASTSSQLG | a-c |
|  |  |  |
|  |  |  |
|  |  |  |

100% a-helixes
